# Enriching Alkene *syn*-Dihalogenation: Aryl Alkene, Bromine, Regio-Reversion, and Stereoconvergency

**DOI:** 10.1021/acs.orglett.6c00241

**Published:** 2026-02-25

**Authors:** Hyeon Moon, Juyeon Hong, Won-jin Chung

**Affiliations:** Department of Chemistry, 65419 Gwangju Institute of Science and Technology, Gwangju 61005, Republic of Korea

## Abstract

Alkene dihalogenation is widely utilized for organohalide synthesis as an indispensable synthetic tool. Ironically, the nearly perfect *anti*-diastereospecificity becomes a formidable hurdle when the other *syn*-diastereochemical course is desired. Thus, accessing the uncharted half of the stereochemical space has been an intriguing synthetic challenge. Despite the recent advancements in *syn*-dihalogenation, critical problems still remain to be resolved, such as the notorious unsuitability of aryl alkenes and low stereospecificity of dibromination. Herein, both of these issues are successfully addressed via our vicinal double electrophilic activation strategy to enable the development of a highly stereoselective *syn*-dibromination of aryl alkenes for the first time. Moreover, the use of two different halogens leads to unusual site-selectivity that is inaccessible via traditional methods. Furthermore, the complementary behavior of the alkene geometrical isomers allows for a rare type of stereoconvergent dibromination from *E*/*Z* mixtures. As a result, our work provides a number of enriching features to this promising research field.

## Graphical abstract

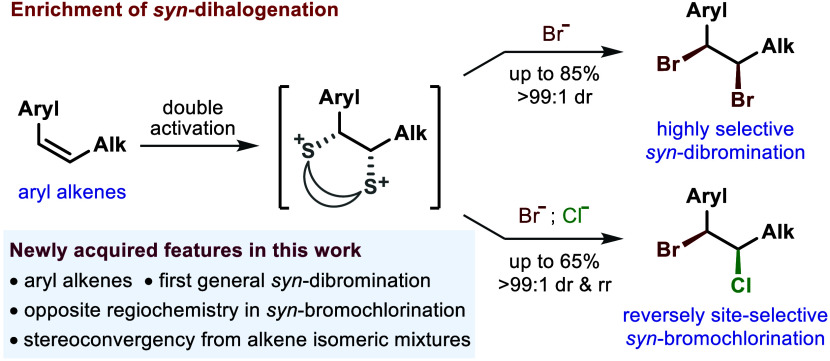

Alkene dihalogenation is a widely utilized classic organic transformation that is highly predictable in terms of stereocontrol, and thus the well-defined *anti*-stereospecificity serves as an indispensable synthetic tool.^1^ On the other hand, infiltration into the other half of the diastereochemical space has been a longstanding challenge in organic synthesis,^2–7^ and many issues still remain unresolved despite the impressive recent progress in this intriguing *syn*-dihalogenation field by Denmark, Jacobsen, Gilmour, Chung, and Lennox.^8–15^ One of the most conspicuous limitations is the unsuitability of aryl alkenes. Because of the pronounced benzylic stabilization, it is difficult to form a configurationally stable species even for the conventional process, and thus the stereochemical information is easily lost via an acyclic carbocationic intermediate (Figure 1A).^16,17^ Probably for this reason, all of the substrates are aliphatic alkenes in Denmark’s seminal study on *syn*-dichlorination (Figure 1B, top).^8,9^ A partial solution was provided by our group through concerted internal trapping at the benzylic position without generating a discrete carbocation (Figure 1B, bottom).^15^ Although the stereochemical integrity could be successfully preserved, the necessity of the pendant stereodirecting group diminished the practicality by restricting the substrate structure. Our group also developed a vicinal double electrophilic alkene activation strategy utilizing thianthrene dication (TT^2+^)^18–23^ and expanded the halogen scope, particularly by enabling controlled additions of two different halogens (Figure 1C).^13^ However, we focused only on the aliphatic substrates in that initial study on the basis of the assumption that aryl substrates would be hard to handle. Then, as a follow-up investigation, we applied our reaction conditions to aryl alkenes **1** such as styrene derivatives, and the vicinal dichlorination was outcompeted by a large degree of elimination, affording arene-conjugated chloroalkenes **2** as the major product (Figure 1D). Fortunately, bromination-initiated sequences proceeded surprisingly well despite the risk of anchimeric stereoscrambling poised by the intrinsically nucleophilic adjacent bromine substituent (Figure 1E).^24^ An excellent level of diastereoselectivity was acquired for *syn*-dibromination (**3**) that had been considered a formidable challenge.^25^ In fact, under similar reaction conditions in our previous report, a structurally analogous aliphatic substrate gave a much-attenuated outcome, which was nonetheless the highest stereospecificity approachable at that time.^13^ Moreover, interhalogenation with bromine and chlorine produced the preferred isomer **4** predominantly out of four possible combinations. It is notable that bromine is incorporated at the benzylic position as opposed to the conventional electrophilic protocol via a bromiranium intermediate using BrCl-type reagents, highlighting the mechanistically distinctive feature of our method. Furthermore, the alkene isomers exhibited unusual stereoconvergent behavior for dibromination (Figure 1F). It appeared that two complementary reaction pathways are operative to merge into the identical diastereo-structure. Whereas *Z*-alkenes undergo two intermolecular brominations as designed, the stereochemical course of *E*-alkenes is inverted by intramolecular bromine participation, thereby allowing for an alkene-geometry-independent, stereoselective dibromination. Here, we describe our successful enrichment of alkene *syn*-dihalogenation chemistry regarding substrate, halogen, regio-reversion, and stereoconvergency.

**Figure 1:**
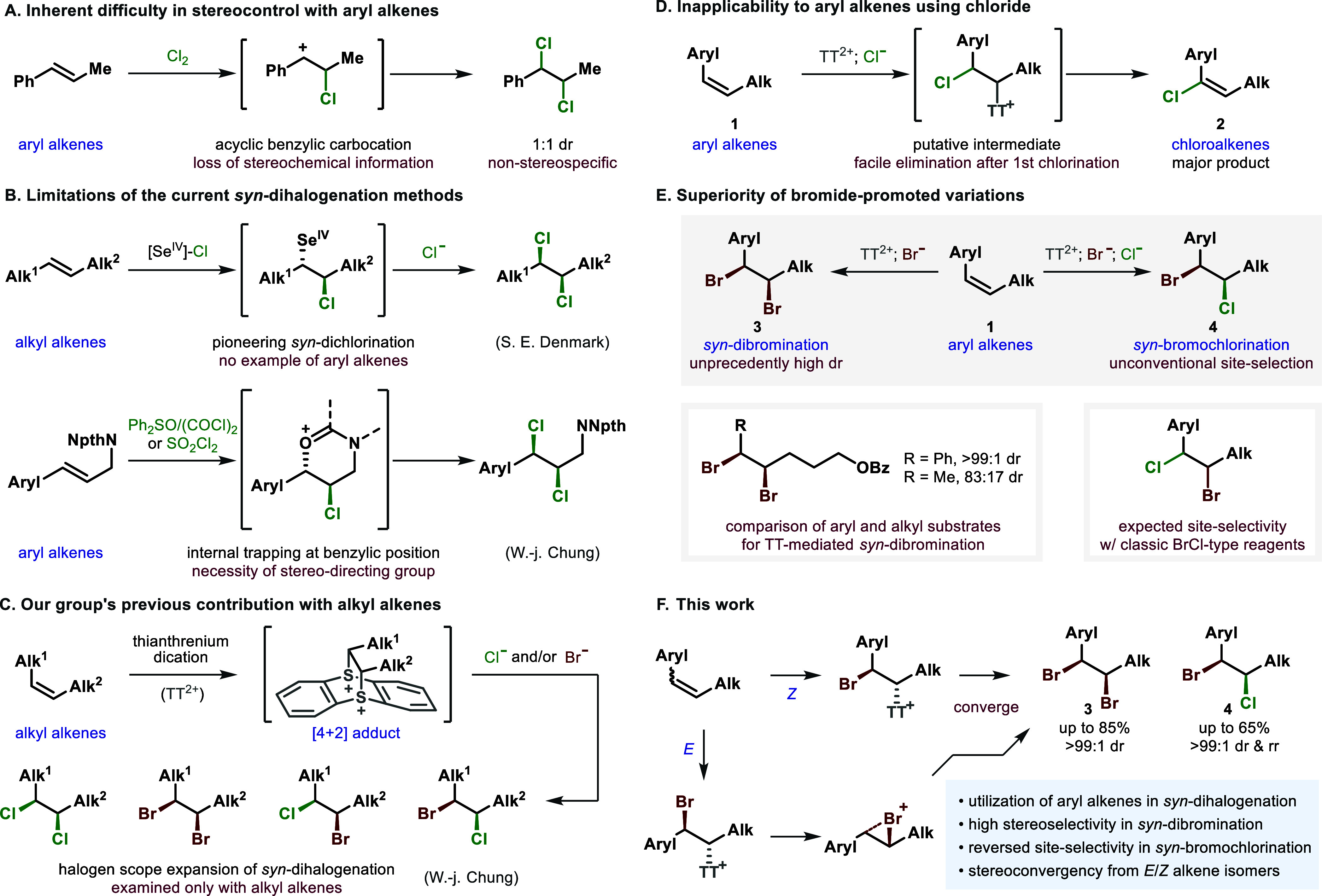
***syn*** **-Dihalogenation of aryl alkenes**. Coverage of uncharted dihalogenation chemical space: highly stereoselective *syn*-dibromination and reversely site-selective *syn*-bromochlorination of aryl alkenes.

The initial reaction condition screening was conducted using a disubstituted *Z*-aryl alkene **1a** (Table 1). With thianthrene-*S*-oxide (TTO, 1.0 equiv), triflic anhydride (Tf_2_O, 1.0 equiv), and tetra-*n*-butylammonium bromide (3.0 equiv) in CH_2_Cl_2_ at room temperature, the *syn*-dibrominated product (**3a**) was afforded in 63% yield with a moderate 90:10 dr (entry 1), which was probably caused by a partial dissociation of the labile benzylic C–S^+^ bond in the alkene-TT adduct. To suppress such stereochemical erosion, the cycloaddition was performed at 0 °C, resulting in a notably improved 95:5 dr (entry 2). A higher loading of TTO had a negligible influence on the reaction outcome (entry 3), and an extended reaction time only provided a more chance for the stereoscrambling (entry 4). Gratifyingly, an electron-rich TTO derivative, 4,7-dimethoxythianthrene-*S*-oxide (DMTTO) appeared to be beneficial for maintaining the stereochemical integrity likely by attenuating the nucleofugality of the sulfonium moiety (entry 5), and complete diastereoselectivity was obtained upon the use of a slight excess. As a side note, a small amount of the starting alkene was regenerated as an isomerized *E*-form, which could be completely removed by recrystallization. Nonetheless, for the purpose of prompt optimization, the yields were simply calibrated spectroscopically after column chromatography.

**Table 1:**
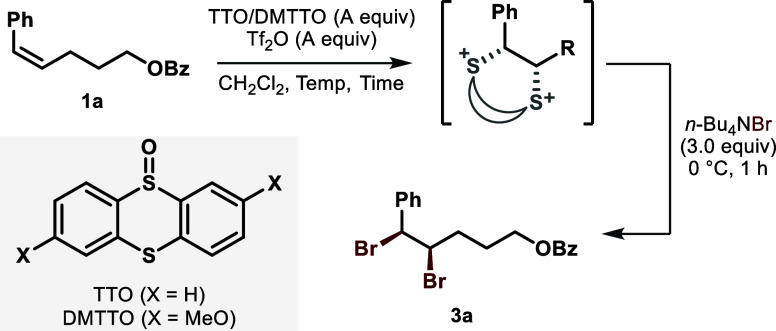
Optimization for *syn*-Dibromination^a^

Entry	(DM)TTO (A)	Temp	Time	Yield^b^	dr^c^
1	TTO (1.0)	rt	1 h	63%	90:10
2	TTO (1.0)	0 °C	1 h	68%	95:5
3	TTO (1.2)	0 °C	1 h	65%	94:6
4	TTO (1.0)	0 °C	2 h	69%	92:8
5	DMTTO (1.0)	0 °C	1 h	65%	96:4
6	DMTTO (1.2)	0 °C	1 h	66%	>99:1

a Standard conditions: 1.0 mmol scale at 0.25 M concentration.

b Calibrated yields based on ^1^H NMR integration after column chromatography.

c Determined by ^1^H NMR analysis of the purified materials. Bz: benzoyl, TTO: thianthrene-*S*-oxide, DMTTO: 4,7-dimethoxythianthrene-*S*-oxide, Tf: trifluoromethanesulfonyl, dr: diastereomeric ratio.

With the optimized reaction conditions in hand, various aryl alkenes were examined for *syn*-dibromination (Figure 2). The presence of an electron-donating methyl group was tolerated (**3b** and **3c**) even at the *ortho*-position, indicating that our reaction is insensitive to the steric hindrance of the aryl moiety. However, the oxidative C–H thianthrenation became problematic for highly electron-rich aryl substrates.^26^ In the cases with electron-withdrawing trifluoromethyl (**3d**), trifluoromethoxy (**3e**), or chlorine (**3g**) substitution, the excellent level of diastereoselectivity was maintained although sluggish cycloaddition or pronounced elimination to the haloalkene led to moderate yields. Functional group compatibility was further demonstrated through the modification of the distal ester part. Electronically altered arenes (**3h** and **3i**) as well as heterocycles including furoyl (**3j**) and thienoyl (**3k**) were all accommodated well to give the corresponding *syn*-dibromides with high efficiency. Surprisingly, the reactive tosyl group (**3l**) remained intact, showcasing the mildness of our reaction conditions. Furthermore, exclusive chemoselectivity was observed (**3m**) in the presence of a less electron-rich alkene. For the aliphatic side of the alkene, a bulkier secondary alkyl group (**3n**) was amenable to give a reasonable yield. Unfortunately, alkenes bearing a tertiary alkyl group were unsuitable substrates because of the difficulty in S_N_2 displacement at the neopentylic carbon. The stereochemistry of our *syn*-dibromination was confirmed by the single crystallographic X-ray diffraction analysis of **3f** (see Section 3.1 of the SI).

**Figure 2:**
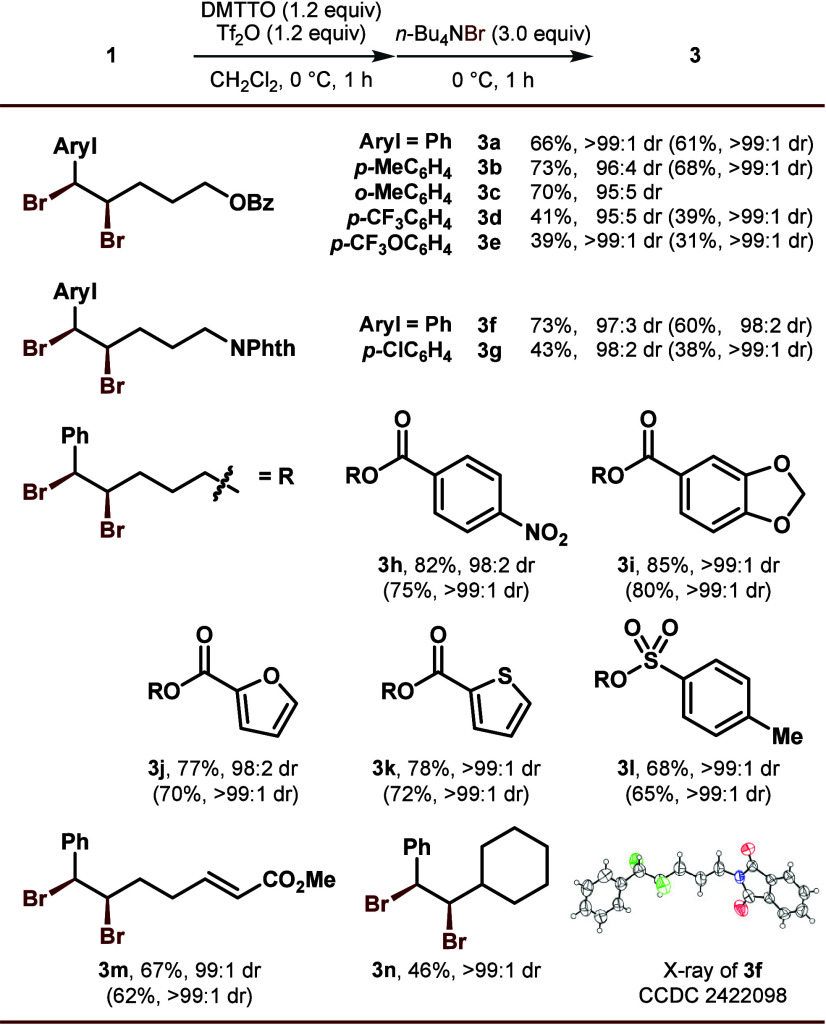
**Substrate scope of** ***syn*** **-dibromination of aryl alkenes**. All reactions were performed on a 1.0 mmol scale at 0.25 M concentration. Calibrated yields based on ^1^H NMR integration after column chromatography are given. Diastereomeric ratios (dr) were determined by ^1^H NMR analysis. The data of the pure materials after recrystallization are given in parentheses. Phth = phthaloyl.

Following the successful development of a general *syn*-dibromination of aryl alkenes, we investigated a more advanced variant involving two different halides,^27–29^ which had been shown to be amenable to dialkyl alkenes in our previous work (Figure 1C).^13^ For the controlled sequential reactions of bromine and chlorine with the alkene-TT adduct, the first halogenation must take place monoselectively and site-selectively with high conversion. Otherwise, the homodihalides and the constitutional isomer would be produced as inseparable side products. To that end, it was critical to conduct the reaction at a low temperature while ensuring complete consumption of the cycloadduct. Fortunately, in the current aryl alkene system, the benzylic activation would accelerate the first halogenation even at low temperatures and also provide an intrinsically preferred reacting site. Consequently, nearly exclusive site-selectivity was obtained for *syn*-bromochlorination of **1a** at 0 °C within 1 h (see Section 2.7 of the SI). Again, trace amounts of the aforementioned dihalide side products and the regenerated *E*-alkene could be removed by recrystallization, but spectroscopically calibrated yields were used to facilitate the optimization process. The moderate chemical yield could be improved to a useful level below −30 °C, probably through the stabilization of reactive intermediates. Eventually, −78 °C was selected because it was the most convenient temperature to achieve.

Under the optimized reaction conditions, the scope of the site-selective *syn*-bromochlorination was then explored (Figure 3). Similarly to the *syn*-dibromination, excellent diastereo- and site-selectivity were preserved through electronic and steric variations of the phenyl substituent (**4b**–**4g**). The tethered benzoate part could also be modified without impacting the outcome (**4h**–**4l**). The labile tosyl group again survived without deteriorating the reaction efficiency, which underscores the notable functional group tolerance of our protocol. Furthermore, as alluded to previously (Figure 1E), the current method tweaks the traditional approach in terms of both stereo- and regiochemical aspects, expanding the chemical pool of vicinal bromochloride.

**Figure 3:**
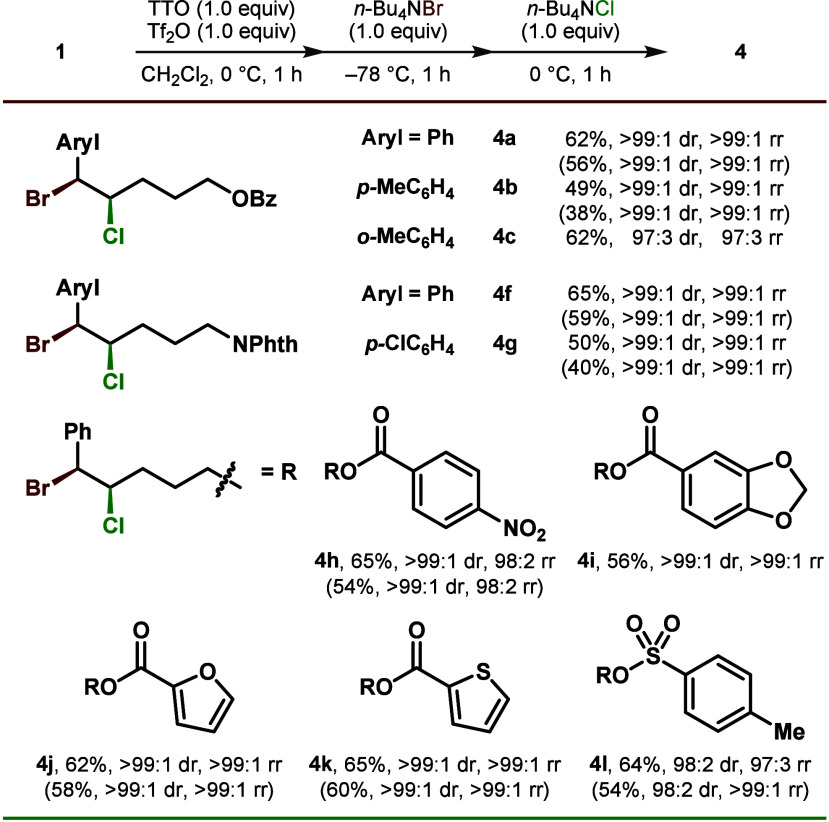
**Substrate scope of** ***syn*** **-bromochlorination of aryl alkenes**. All reactions were performed on a 1.0 mmol scale at 0.25 M concentration. Calibrated yields based on ^1^H NMR integration after column chromatography are given. Diastereomeric ratios (dr) and regioisomeric ratios (rr) were determined by ^1^H NMR analysis. The data of the pure materials after recrystallization are given in parentheses.

While the effect of alkene geometry was examined, an unexpected diastereochemical behavior was observed (Figure 4A). Although the *E*-isomer of **1a** underwent highly selective dibromination, the *anti*-addition process appeared to be operative. As a result, the same 1,2-dibromide diastereomer was produced from both the *E*- and *Z*-alkenes. Accordingly, a rare type of diastereoconvergency could be acquired from an isomeric mixture of **1a**, which can be of high synthetic utility when geometrically pure alkene is difficult to access. This result also demonstrates the superiority of aryl alkenes as substrates because aliphatic *E*-alkenes were poorly stereoselective for dibromination under the essentially identical reaction conditions.^13^ The enhanced Br nucleophilicity from the more polarized benzylic C–Br bond is probably responsible for promoting the bromiranium formation.^30^ Unfortunately, stereoconvergency is intrinsically unattainable for bromochlorination because the *E/Z*-alkene isomers would provide the opposite site-selectivity.

**Figure 4:**
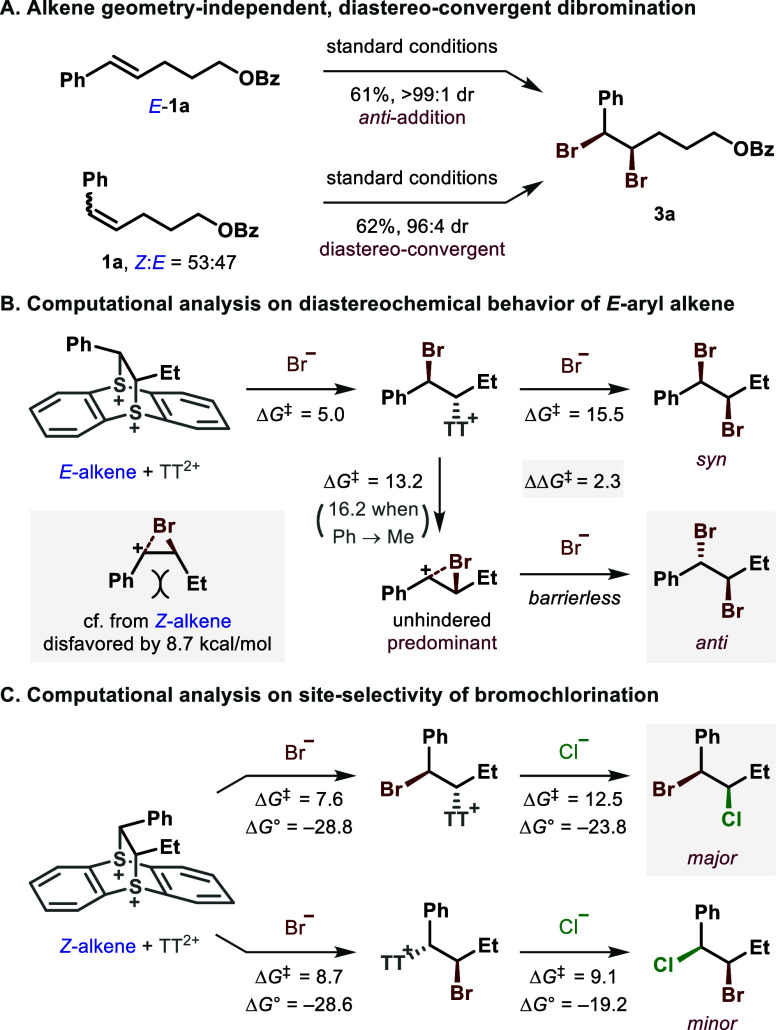
**Mechanistic discussion**. Computation at *w*B97X-D/6–31+G­(d,p), SMD­(CH_2_Cl_2_), free energies in kcal/mol.

To gain insight into the observed stereochemical dichotomy, the sequential nucleophilic brominations of a simplified *E*-alkene + TT^2+^ adduct were analyzed by the density functional theory (DFT) calculation (Figure 4B).^31–33^ After the facile first bromide addition at the benzylic site of the bis-sulfonium species, two competing pathways are encountered. Whereas the intermolecular substitution affords the *syn*-addition product, the intramolecular displacement leads to the generation of an activated cyclic bromiranium-like species, the intermediate for classic *anti*-dihalogenation. In the case of *E*-alkene, the formation of sterically unhindered *trans*-bromiranium is favored by 2.3 kcal/mol, resulting in the predominant *anti*-addition. Furthermore, the nontraditional site-selectivity of our *syn*-bromochlorination was examined (Figure 4C). As one can expect, substitution at the benzylic site is favored by 1.1 kcal/mol in the irreversible, regiodetermining first halogenation step. This computed value is in good agreement with the experimental result, which generally showed a site-selectivity of 96:4 or higher.

In conclusion, we have tackled conspicuous problems in alkene *syn*-dihalogenation chemistry and successfully enriched this intriguing field by acquiring several features that were previously inaccessible. Our reaction process enables the use of notoriously difficult aryl alkene substrates that are known to lose the stereochemical integrity easily through the formation of benzylic cation, which is suppressed by the intermediacy of an expanded cycle that is less strained than the classic haliranium ion. In addition, a highly selective *syn*-dibromination with wide substrate generality is developed for the first time. The steric encumbrance exerted by the aryl substituent hampers the well-known anchimeric participation of the bromine substituent, which would form an unstable *cis*-bromiranium. Moreover, the sequential additions of two different halogens, bromine and chlorine, lead to a highly stereospecific *syn*-bromochlorination with exceptional site-selectivity that is opposite to those from the classic BrCl-type system employing an electrophilic bromine. Furthermore, the complementary behavior of *E*-alkenes through facile formation of stable *trans*-bromiranium-like species and the consequent *anti*-addition allows for a practical stereoconvergent alkene dibromination that is independent of the alkene geometry. We believe that our work unlocks the potential utility of *syn*-dihalogenation to reach a synthetically attractive level.

## Supplementary Material





## Data Availability

The data underlying this study are available in the published article and its

## Notes

Computation output files (ZIP)
Experimental details, characterization data, and copies of NMR spectra for all new compounds (PDF)
