# Supplementary material for: Enriching Alkene syn-Dihalogenation: Aryl Alkene, Bromine, Regio-Reversion, and Stereoconvergency
Source: Org Lett. 2026 Feb 25;28(9):3002–7. doi: 10.1021/acs.orglett.6c00241 (PMC12973299; doi:10.1021/acs.orglett.6c00241)
Supplement: Supplementary file 2 [file ol6c00241_si_002.pdf]

Enriching Alkene *syn*-Dihalogenation:

Aryl Alkene, Bromine, Regio-reversion, and Stereoconvergency

Hyeon Moon, Juyeon Hong, and Won-jin Chung*

Department of Chemistry, Gwangju Institute of Science and Technology, Gwangju 61005, Republic of Korea.

e-mail: wjchung@gist.ac.kr

Supporting Information

1. General Experimental ········· *S2*

2. Synthetic Procedures ········· *S3*

2.1. Literature Preparations ········· *S3*

2.2. Preparation of Substrates ········· *S3*

2.3. Preparation of 4,7-Dimethoxythianthrene-*S*-oxide (**S3**) ········· *S12*

2.4. Synthesis of (4*R**,5*S**)-4-Bromo-5-chloro-5-phenylpentyl Benzoate (**5a**) ········· *S13*

2.5. General Procedure I: *syn*-Dibromination of Alkenes ········· *S13*

2.6. General Procedure II: *syn*-Bromochlorination of Alkenes ········· *S20*

2.7. Reaction Condition Optimization for *syn*-Bromochlorination ········· *S25*

2.8. *anti*-Dibromination of *E*-**1a** ········· *S25*

2.9. Stereoconvergent Dibromination of *Z*/*E* Mixture of **1a** ········· *S25*

3. Characterization Figures ········· *S26*

3.1. X-Ray Crystallographic Data ········· *S26*

3.2. NMR Spectra ········· *S28*

4. References ········· *S113*

1. General Experimental

All reactions were performed in oven-dried (140 °C) or flame-dried glassware under an atmosphere of dry argon unless otherwise noted. Tetrahydrofuran (THF), diethyl ether (Et_2O), dichloromethane (CH_2Cl_2), and acetonitrile (MeCN) were dried by percolation through a column packed with neutral alumina, and a column packed with Q5 reactant, a supported copper catalyst for scavenging oxygen, under a positive pressure of argon. Methanol (MeOH) was dried with Mg/I_2, distilled under Ar atmosphere, and stored over 3 Å molecular sieves prior to use. Dimethyl sulfoxide (DMSO) was dried and stored over 3 Å molecular sieves prior to use. Heptane and toluene were dried with CaH_2, distilled under reduced pressure, and stored over 3 Å molecular sieves prior to use. 3,4-Dihydropyran was distilled under argon prior to use. The following reagents were distilled from the indicated drying agents under argon prior to use: acetic acid (Ac_2O), triethylamine (CaH_2), and triflic anhydride (P_2O_5). The following reagents were distilled under reduced pressure prior to use: cyclohexene, and benzoyl chloride (BzCl). The following reagents were recrystallized from the indicated solvents prior to use: thianthrene (acetone), triphenylphosphine (hexanes), phthalimide (CH_2Cl_2/hexanes), 4-(dimethylamino)pyridine (CH_2Cl_2/hexanes), and methyl (triphenylphosphoranylidene)acetate (CH_2Cl_2/hexanes). Iodine (Duksan, Extra pure), $MoCl_5$ (Alfa, 99.6%), $Fe(NO_3)_3$•$9H_2O$ (Alfa, >98%), sodium bromide (Aldrich, 98%), tetra-*n*-butylammonium chloride (Aldrich, 97%), tetra-*n*-butylammonium bromide (Alfa, 98%), dichlorobis(triphenylphosphine)nickel(II) (ACROS, 98%), oxalyl chloride (Alfa, 98%), 2-thenoyl chloride (TCI, 99%), 2-furoyl chloride (TCI, 98%), piperonylic acid (Aldrich, 99%), 4-nitrobenzoyl chloride (Alfa, 98%), BH_3•SMe_3 (2M in THF, alfa), *N*-bromosuccinimide (NBS, Alfa, 99%), thionyl chloride ($SOCl_2$, Daejung, 99%), and diisopropyl azodicarboxylate (DIAD, Alfa, 94%) were used without further purification. Solvents and reagents for workup and chromatography were hexanes (Duksan, Extra Pure), ethyl acetate (EtOAc, Duksan, Extra Pure), CH_2Cl_2 (Duksan, Extra Pure), Et_2O (Daejung, Extra Pure), and pentane (Daejung, Extra Pure). Quenching solutions and drying reagents for workup were magnesium sulphate (Duksan, Extra pure), sodium bicarbonate (Daejung, Extra Pure), potassium carbonate (Duksan, Extra pure), sodium sulfite (Samchun, 85%), and sodium chloride (Daejung, Extra pure). ^1H and ^{13}C NMR spectra were recorded on a JEOL JCX-400 spectrometer (400 MHz, ^1H; 100 MHz, ^{13}C; 376 MHz, ^{19}F). Spectra were referenced to residual chloroform (7.26 ppm, ^1H; 77.23 ppm, ^{13}C), benzene (128.06 ppm, ^{13}C) hexafluorobenzene (–164.9 ppm, ^{19}F). Chemical shifts were reported in ppm, and multiplicities are indicated by s (singlet), d (doublet), t (triplet), q (quartet), pent (pentet), and m (multiplet). Coupling constants, *J*, are reported in Hertz. Kugelrohr distillation was carried out using a Büchi B585 glass oven with Büchi bulb-to-bulb distillation apparatus, and air bath temperatures (ABT) are reported. Filtration and column chromatography were performed using Merck 230-400 mesh silica gel. Analytical thin layer chromatography (TLC) was conducted on Merck silica gel 60 F_{254} TLC plates. Visualization was accomplished with UV (254 nm) as well as potassium permanganate ($KMnO_4$) and ceric ammonium molybdate (CAM) staining solutions. ESI-HRMS was performed on a Bruker Impact II quadrupole-time-of-flight (Q-TOF) spectrometer at GIST Advanced Institute of Instrumental Analysis (GAIA). FAB-HRMS and EI-HRMS were performed on a JEOL JMS-700 MStation mass spectrometer at Korea Basic Science Institute (KBSI), Daegu Center. Data are reported in the form of *m/z*. Elemental analysis was conducted on an Elementar UNICUBE at GIST Advanced Institute of Instrumental Analysis (GAIA). X-ray crystallographic data for **3k** was collected using a Rigaku Oxford Diffraction XtaLAB Synergy-S diffractometer using $Cu_{K\alpha}$ radiation (λ = 1.54184 Å) at GIST Advanced Institute of Instrumental Analysis (GAIA). Melting point was determined using a Büchi M-560 melting point apparatus.

2. Synthetic Procedures

2.1. Literature Preparations

The following compounds were prepared according to literature procedures: (*Z*)-5-phenylpent-4-en-1-ol,[1] (*E*)-5-phenylpent-4-en-1-ol,[1] (*Z*)-5-(*o*-tolyl)pent-4-en-1-ol[1], (*Z*)-5-(*p*-tolyl)pent-4-en-1-ol,[1] 5-[4-(Trifluoromethyl)phenyl]-4-pentyn-1-ol,[2] (*Z*)-5-(4-chlorophenyl)pent-4-en-1-ol,[1] (*Z*)-5-(4-(trifluoromethoxy)phenyl)pent-4-en-1-ol,[1] (*Z*)-5-phenylpent-4-en-1-yl 4-methylbenzenesulfonate,[1] (cyclohexylethynyl)benzene,[3] (*E*)-5-phenylpent-4-en-1-yl benzoate (*E*-**1a**),[4] thianthrene-*S*-oxide.[5]

2.2. Preparation of Substrates

(*Z*)-5-phenylpent-4-en-1-yl benzoate (**1a**)

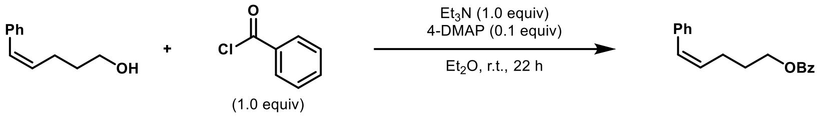

To a stirred solution of 4-DMAP (96 mg, 0.79 mmol, 0.1 equiv), (*Z*)-5-phenylpent-4-en-1-ol (1.28 g, 7.87 mmol, 1.0 equiv, *Z* only), and Et_3N (1.1 mL, 7.9 mmol, 1.0 equiv) in Et_2O (8.0 mL) was added benzoyl chloride (0.91 mL, 7.9 mmol, 1.0 equiv) at room temperature. After 22 hours (unoptimized), H_2O (20 mL) was added, and the organic layer was separated. The aqueous layer was extracted with EtOAc (20 mL × 2), and the combined organic extracts were dried over $MgSO_4$ (1.81 g), filtered through a glass frit, and concentrated in vacuo. The crude material was purified by flash column chromatography (SiO_2, ϕ = 3.0 cm, l = 11 cm) eluting with CH_2Cl_2/hexanes = 1/3 (R_f = 0.35 [254 nm/$KMnO_4$]) to afford a colorless oil (2.02 g, 96%). Further purification by Kugelrohr distillation (P = 0.5 mmHg, ABT = 265 °C) provided a colorless oil (1.91 g, 91%, *Z*/*E* > 99/1).

Data for **1a**: JYH-01-035

^{1}H NMR: (400 MHz, $CDCl_3$)
δ 8.00–7.98 (m, 2H), 7.58–7.53 (m, 1H), 7.46–7.40 (m, 2H), 7.33–7.24 (m, 4H), 7.23–7.19 (m, 1H), 6.50 (d, *J* = 11.6, 1H), 5.71 (dt, *J* = 11.6, 7.6, 1H), 4.35 (t, *J* = 6.4, 2H), 2.52 (appr. q, *J* = 7.4, 2H), 1.93 (appr. pent, *J* = 6.9, 2H).

^{13}C NMR: (100 MHz, $CDCl_3$)
δ 166.8, 137.6, 133.4, 131.5, 130.5, 130.2, 129.7, 128.9, 128.5, 128.4, 126.8, 64.5, 29.1, 25.3.

HRMS (EI): $[M]^+$ calcd for $C_{18}H_{18}O_2$: 266.1307; found: 266.1309.

(*Z*)-5-(*p*-tolyl)pent-4-en-1-yl benzoate (**1b**)

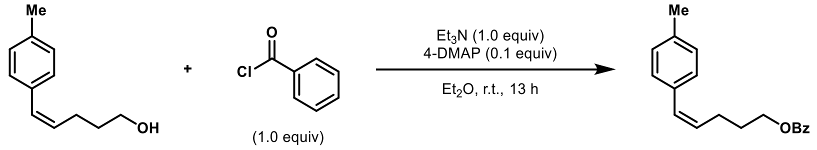

To a stirred solution of 4-DMAP (62 mg, 0.51 mmol, 0.1 equiv), (*Z*)-5-(*p*-tolyl)pent-4-en-1-ol (895 mg, 5.08 mmol, 1.0 equiv), and Et_3N (0.71 mL, 5.1 mmol, 1.0 equiv) in Et_2O (5.1 mL) was added benzoyl chloride (0.59 mL, 5.1 mmol, 1.0 equiv) at room temperature. After 18 hours (unoptimized), H_2O (20 mL) was added, and the organic layer was separated. The aqueous layer was extracted with EtOAc (30 mL × 3), and the combined organic extracts were dried over $MgSO_4$ (5 g), filtered through a glass frit, and concentrated in vacuo. The crude material was purified by flash column chromatography (SiO_2, ϕ = 3.5 cm, l = 7.5 cm) eluting with EtOAc/hexanes = 1/20 (R_f = 0.35 [254 nm/$KMnO_4$]) to give a pale-yellow oil (1.36 g, 96%). Further purification by Kugelrohr distillation (P = 0.15 mmHg, ABT = 260 °C) provided a colorless oil (1.34 g, 95%).

Data for **1b**: JYH-01-057

^{1}H NMR: (400 MHz, $CDCl_3$)

δ 8.00–7.97 (m, 2H), 7.57–7.52 (m, 1H), 7.42 (t, *J* = 7.8, 2H), 7.17 (d, *J* = 8.2, 2H), 7.10 (d, *J* = 8.2, 2H), 6.46 (d, *J* = 11.6, 1H), 5.68–5.62 (m, 1H), 4.35 (t, *J* = 6.7, 2H), 2.54–2.48 (m, 2H), 2.32 (s, 3H), 1.98–1.89 (m, 2H).

^{13}C NMR: (100 MHz, $CDCl_3$)

δ 166.8, 136.5, 134.7, 133.0, 130.8, 130.6, 130.1, 129.8, 129.1, 128.8, 128.5, 64.5, 29.1, 25.3, 21.4.

HRMS (EI): $[M]^+$ calcd for $C_{19}H_{20}O_2$: 280.1463; found: 280.1461.

(*Z*)-5-(*o*-tolyl)pent-4-en-1-yl benzoate (**1c**)

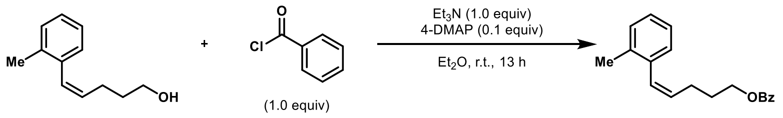

To a stirred solution of 4-DMAP (68 mg, 0.56 mmol, 0.1 equiv), (*Z*)-5-(*o*-tolyl)pent-4-en-1-ol (985 mg, 5.59 mmol, 1.0 equiv, *Z* only), and Et_3N (0.78 mL, 5.6 mmol, 1.0 equiv) in Et_2O (5.6 mL) was added benzoyl chloride (0.65 mL, 5.6 mmol, 1.0 equiv) at room temperature. After 13 hours (unoptimized), H_2O (10 mL) was added, and the organic layer was separated. The aqueous layer was extracted with EtOAc (10 mL × 3), and the combined organic extracts were dried over $MgSO_4$ (4.74 g), filtered through a glass frit, and concentrated in vacuo. The crude material was purified by flash column chromatography (SiO_2, ϕ = 5.0 cm, l = 10 cm) eluting with CH_2Cl_2/hexanes = 1/4 (R_f = 0.20 [254 nm/$KMnO_4$]) to afford a colorless oil (1.50 g, 96%). Further purification by Kugelrohr distillation (P = 0.5 mmHg, ABT = 230 °C) provided a colorless oil (1.44 g, 92%, *Z/E* > 99/1).

Data for **1c**: HM-11-012

^{1}H NMR: (400 MHz, $CDCl_3$)

δ 7.94–7.92 (m, 2H), 7.54–7.52 (m, 1H), 7.42–7.38 (m, 2H), 7.17–7.09 (m, 4H), 6.52 (d, *J* = 11.6, 1H), 5.75 (dt, *J* = 11.6, 7.3, 1H), 4.30 (t, *J* = 6.4, 2H), 2.39–2.31 (m, 2H), 2.24 (s, 3H), 1.87 (appr. pent, *J* = 6.9, 2H).

^{13}C NMR: (100 MHz, $CDCl_3$)

δ 166.7, 136.6, 136.3, 132.9, 131.2, 130.4, 129.9, 129.6, 129.4, 129.0, 128.4, 127.0, 125.4, 64.3, 28.8, 24.8, 20.0.

HRMS (EI): $[M]^+$ calcd for $C_{19}H_{20}O_2$: 280.1463; found: 280.1461.

5-(4-(trifluoromethyl)phenyl)pent-4-yn-1-yl benzoate (**S1**)

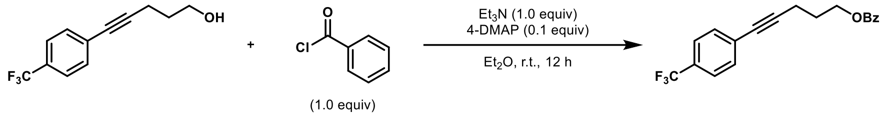

To a stirred solution of 4-DMAP (64 mg, 0.53 mmol, 0.1 equiv), 5-(4-(trifluoromethyl)phenyl)pent-4-yn-1-ol (1.20 g, 5.27 mmol, 1.0 equiv), and Et_3N (0.73 mL, 5.3 mmol, 1.0 equiv) in Et_2O (5.6 mL) was added benzoyl chloride (0.61 mL, 5.3 mmol, 1.0 equiv) at room temperature. After 12 hours (unoptimized), H_2O (10 mL) was added, and the organic layer was separated. The aqueous layer was extracted with EtOAc (10 mL × 3), and the combined organic extracts were dried over $MgSO_4$ (1.74 g), filtered through a glass frit, and concentrated in vacuo. The crude material was purified by flash column chromatography (SiO_2, ϕ = 4.0 cm, l = 12 cm) eluting with CH_2Cl_2/hexanes = 1/3 (R_f = 0.70 [254 nm/CAM(blue)]) to afford **S1** as a colorless oil (1.72 g, 98%).

Data for **S1**: HM-11-017

^{1}H NMR: (400 MHz, $CDCl_3$)
δ 8.07–8.05 (m, 2H), 7.58–7.52 (m, 3H), 7.48–7.41 (m, 4H), 4.50 (t, J = 6.4, 2H), 2.64 (t, J = 7.0, 2H), 2.10 (appr. pent, J = 6.6, 2H).

^{13}C NMR: (100 MHz, $CDCl_3$)
δ 166.7, 133.1, 132.0, 130.4, 129.7, 129.6 (q, J_{CF3} = 32.4), 128.5, 127.7, 125.3 (d, J_{CF3} = 3.5), 124.2 (q, J_{CF3} = 271.2), 91.7, 80.4, 63.8, 27.9, 16.6.

^{19}F NMR: (376 MHz, $CDCl_3$)
δ –66.0.

HRMS (EI): $[M]^+$ calcd for $C_{19}H_{15}F_3O_2$: 332.1027; found: 332.1024.

(*Z*)-5-(4-(trifluoromethyl)phenyl)pent-4-en-1-yl benzoate (**1d**)

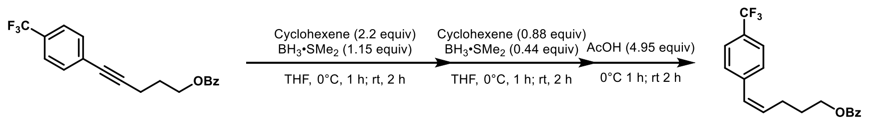

To a stirred solution of cyclohexene (1.0 mL, 9.9 mmol, 2.2 equiv) in THF (5 mL) was added a solution of BH_3•SMe_2 (2.59 mL, 5.18 mmol, 1.15 equiv) at 0 °C. After 1 hour, a solution of 5-(4-(trifluoromethyl)phenyl)pent-4-yn-1-yl benzoate (1.5 g, 4.5 mmol, 1.0 equiv) in THF (6.3 mL) was added to the reaction mixture. After 1 hour, the reaction mixture was heated to room temperature. After 2 hours, to another stirred solution of cyclohexene (0.40 mL, 4.0 mmol, 0.88

equiv) in THF (1 mL) was added a solution of BH_3•SMe_2 (1.00 mL, 2.28 mmol, 0.44 equiv) at 0 °C. After 1 hour, the reaction mixture was transferred to Cy_2BH solution via cannular. After 1 hour, the reaction mixture was heated to room temperature. After 2 hours, AcOH (1.28 mL, 22.3 mmol, 4.95 equiv) was added at 0 °C. After 1 hour, the reaction mixture was heated to room temperature and $NaHCO_3$ (10 mL) was added, and the organic layer was separated. The aqueous layer was extracted with EtOAc (10 mL × 3), and the combined organic extracts were washed with brine (20 mL), H_2O (20 mL), dried over $MgSO_4$ (4.74 g), filtered through a glass frit, and concentrated in vacuo. The crude material was purified by flash column chromatography (SiO_2, ϕ = 4.0 cm, l = 10 cm) eluting with CH_2Cl_2/hexanes = 1/3 (R_f = 0.30 [254 nm/CAM(grey)]) to afford a colorless oil (1.70 g, 113%). Further purification by Kugelrohr distillation (P = 0.5 mmHg, ABT = 260 °C) provided a colorless oil (1.34 g, 89%, *Z/E* > 99/1, + 6% alkyne).

Data for **1d**: HM-11-043

^{1}H NMR: (400 MHz, $CDCl_3$)

δ 7.96–7.94 (m, 2H), 7.55–7.51 (m, 3H), 7.43–7.39 (m, 2H), 7.35 (d, J = 7.9, 2H), 6.51 (d, J = 11.6, 1H), 5.81 (dt, J = 11.6, 7.6, 1H), 4.34 (t, J = 6.1, 2H), 2.52–2.47 (m, 2H), 1.99–1.90 (m, 2H).

^{13}C NMR: (100 MHz, $CDCl_3$)

δ 166.6, 141.1, 133.5, 133.1, 130.3, 129.6, 129.0, 128.7 (q, J_{CF3} = 32.6), 128.5, 127.1 (q, J_{CF3} = 275.1), 125.2 (d, J_{CF3} = 3.8), 64.1, 28.8, 25.1.

^{19}F NMR: (376 MHz, $CDCl_3$)

δ –65.6.

HRMS (FAB): $[M+H]^+$ calcd for $C_{19}H_{18}F_3O_2$: 335.1259; found: 335.1265.

(*Z*)-5-(4-(trifluoromethoxy)phenyl)pent-4-en-1-yl benzoate (**1e**)

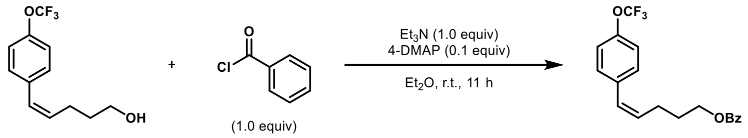

To a stirred solution of 4-DMAP (46 mg, 0.36 mmol, 0.1 equiv), (*Z*)-5-(4-(trifluoromethoxy)phenyl)pent-4-en-1-ol (897 mg, 3.64 mmol, 1.0 equiv, *Z* only), and Et_3N (0.50 mL, 3.6 mmol, 1.0 equiv) in Et_2O (4.0 mL) was added benzoyl chloride (0.42 mL, 3.6 mmol, 1.0 equiv) at room temperature. After 11 hours (unoptimized), H_2O (20 mL) and CH_2Cl_2 (20 mL) were added, and the organic layer was separated. The aqueous layer was extracted with CH_2Cl_2 (20 mL × 3), and the combined organic extracts were dried over $MgSO_4$ (3 g), filtered through a glass frit, and concentrated in vacuo. The crude material was purified by flash column chromatography (SiO_2, ϕ = 3.5 cm, l = 16 cm) eluting with EtOAc/hexanes = 1/10 (R_f = 0.45 [254 nm/CAM (blue)]) to afford a colorless oil (1.20 g, 96%). Further purification by Kugelrohr distillation (P = 0.15 mmHg, ABT = 260 °C) provided a colorless oil (1.15 g, 90%, *Z/E* > 99/1).

Data for **1e**: JYH-02-065

^{1}H NMR: (400 MHz, $CDCl_3$)

δ 7.98 (d, *J* = 7.0, 2H), 7.55 (t, *J* = 7.3, 1H), 7.44–7.40 (m, 2H), 7.28–7.26 (m, 2H), 7.12 (d, *J* = 8.2, 2H), 6.46 (d, *J* = 11.6, 1H), 5.74 (dt, *J* = 11.6, 7.3, 1H), 4.35 (t, *J* = 6.4, 2H), 2.52–2.46 (m, 2H), 1.97–1.90 (m, 2H).

^{13}C NMR: (100 MHz, $CDCl_3$)
δ 166.7, 147.9, 136.3, 133.1, 132.4, 130.5, 130.2, 129.7, 128.8, 128.5, 120.8, 120.7 (q, J_{CF3} = 256.9), 64.3, 29.0, 25.2.

^{19}F NMR: (376 MHz, $CDCl_3$)
δ –60.8.

HRMS (ESI): $[M+Na]^+$ calcd for $C_{19}H_{17}F_3NaO_3$: 373.1027; found: 373.1022.

(*Z*)-2-(5-phenylpent-4-en-1-yl)isoindoline-1,3-dione (**1f**)

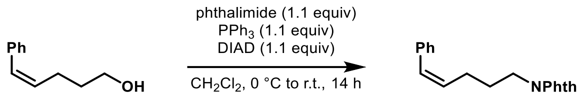

To a stirred solution of phthalimide (563 mg, 3.85 mmol, 1.1 equiv), triphenylphosphine (1.01 g, 3.85 mmol, 1.1 equiv), and (*Z*)-5-phenylpent-4-en-1-ol (568 mg, 3.50 mmol, 1.0 equiv, *Z*/*E* > 99/1) in CH_2Cl_2 (18 mL) was added DIAD (0.76 mL, 3.5 mmol, 1.1 equiv) at 0 °C. Then, the reaction mixture was warmed up to room temperature. After 14 hours (unoptimized), H_2O (20 mL) was added, and the organic layer was separated. The aqueous layer was extracted with CH_2Cl_2 (20 mL × 3), and the combined organic extracts were dried over $MgSO_4$ (5.84 g), filtered through a glass frit, and concentrated in vacuo. The crude material was purified by flash column chromatography (SiO_2, ϕ = 4.0 cm, l = 9 cm) eluting with EtOAc/hexanes = 1/10 (R_f = 0.25 [254 nm/CAM(grey)) to afford a pale-yellow oil (870 mg, 85%). Further purification by recrystallization from CH_2Cl_2/hexanes provided a white solid (805 mg, 79%, *Z*/*E* > 99/1).

Data for **1f**: HM-11-055

^{1}H NMR: (400 MHz, $CDCl_3$)
δ 7.83–7.81 (m, 2H), 7.70–7.68 (m, 2H), 7.28–7.18 (m, 5H), 6.43 (d, *J* = 11.6, 1H), 5.66 (dt, *J* = 11.9, 7.3, 1H), 3.69 (t, *J* = 7.3, 2H), 2.41–2.38 (m, 2H), 1.86–1.81 (m, 2H).

^{13}C NMR: (100 MHz, $CDCl_3$)
δ 168.6, 137.5, 134.1, 132.3, 131.4, 130.0, 128.9, 128.4, 126.8, 123.4, 37.8, 28.9, 26.2.

HRMS (EI): $[M]^+$ calcd for $C_{19}H_{17}NO_2$: 291.1259; found: 291.1259.

(*Z*)-2-(5-(4-chlorophenyl)pent-4-en-1-yl)isoindoline-1,3-dione (**1g**)

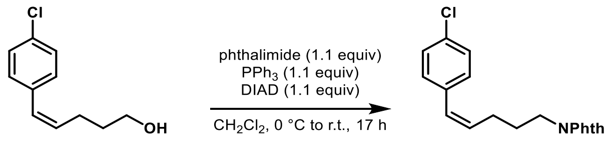

To a stirred solution of phthalimide (1.09 g, 7.43 mmol, 1.1 equiv), triphenylphosphine (1.95 g, 7.43 mmol, 1.1 equiv), and (*Z*)-5-(4-chlorophenyl)pent-4-en-1-ol (1.33 g, 7.43 mmol, 1.0 equiv, *Z*/*E* = >99/1) in CH_2Cl_2 (34 mL) was added DIAD (1.46 mL, 7.43 mmol, 1.1 equiv) at 0 °C. Then, the reaction mixture was warmed up to room temperature. After 17 hours (unoptimized), H_2O (30 mL) was added, and the organic layer was separated. The aqueous layer was extracted with CH_2Cl_2 (20 mL × 3), and the combined organic extracts were dried over $MgSO_4$ (1.71 g), filtered through a glass frit, and concentrated in vacuo. The crude material was purified by flash column chromatography (SiO_2, ϕ = 4.5 cm, l = 12 cm) eluting with CH_2Cl_2/hexanes = 1/1 (R_f = 0.25 [254 nm/CAM(grey)) to afford a pale-yellow oil (1.70 g, 77%). Further purification by recrystallization from CH_2Cl_2/hexanes provided a white solid (1.27 g, 57%, *Z*/*E* > 99/1).

Data for **1g**: HM-11-071

^{1}H NMR: (400 MHz, $CDCl_3$)
δ 7.84–7.79 (m, 2H), 7.72–7.65 (m, 2H), 7.25–7.22 (m, 2H), 7.15–7.12 (m, 2H), 6.37 (d, *J* = 11.9, 1H), 5.68 (dt, *J* = 11.5, 7.3, 1H), 3.69 (t, *J* = 7.3, 2H), 2.37–2.32 (m, 2H), 1.83 (appr. pent, *J* = 7.5, 2H).

^{13}C NMR: (100 MHz, $CDCl_3$)
δ 168.5, 135.9, 134.1, 132.5, 132.3, 132.1, 130.1, 128.9, 128.5, 123.4, 37.7, 28.7, 26.1.

HRMS (EI): $[M]^+$ calcd for $C_{19}H_{16}ClNO_2$: 325.0870; found: 325.0868.

(*Z*)-5-phenylpent-4-en-1-yl 4-nitrobenzoate (**1h**)

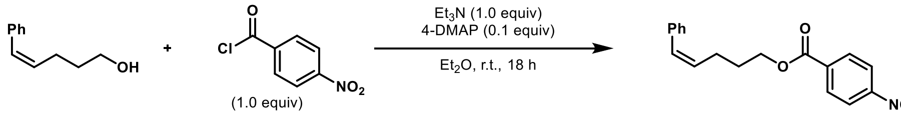

To a stirred solution of 4-DMAP (43 mg, 0.35 mmol, 0.1 equiv), (*Z*)-5-phenylpent-4-en-1-ol (568 mg, 3.50 mmol, 1.0 equiv, *Z* only), and 4-nitrobenzoyl chloride (694 mg, 7.87 mmol, 1.0 equiv) in Et_2O (3.5 mL) was added Et_3N (0.49 mL, 3.5 mmol, 1.0 equiv) at room temperature. After 18 hours (unoptimized), H_2O (10 mL) was added, and the organic layer was separated. The aqueous layer was extracted with CH_2Cl_2 (10 mL × 3), and the combined organic extracts were dried over $MgSO_4$ (3.72 g), filtered through a glass frit, and concentrated in vacuo. The crude material was purified by flash column chromatography (SiO_2, ϕ = 3.5 cm, l = 11 cm) eluting with CH_2Cl_2/hexanes = 1/2 (R_f = 0.20 [254 nm/CAM(grey)]) to afford a pale-yellow oil (1.04 g, 95%). Further purification by Kugelrohr distillation (P = 0.5 mmHg, ABT = 290 °C) provided a pale-yellow oil (1.03 g, 94%, *Z*/*E* > 99/1).

Data for **1h**: HM-11-030

^{1}H NMR: (400 MHz, $CDCl_3$)
δ 8.26–8.23 (m, 2H), 8.10–8.08 (m, 2H), 7.31–7.24 (m, 4H), 7.22–7.18 (m, 1H), 6.52 (d, *J* = 11.6, 1H) 5.69 (dt, *J* = 11.6, 7.3, 1H), 4.38 (t, *J* = 6.4, 2H), 2.52 (qd, *J* = 7.3, 1.5, 2H), 1.94 (appr. pent, *J* = 6.9, 2H).

^{13}C NMR: (100 MHz, $CDCl_3$)
δ 164.8, 150.6, 137.5, 135.9, 131.1, 130.8, 130.5, 128.9, 128.4, 126.9, 123.7, 65.3, 28.8, 25.0.

HRMS (EI): [M]+ calcd for $C_{18}H_{17}NO_4$: 311.1158; found: 311.1154.

(*Z*)-5-phenylpent-4-en-1-yl benzo[d][1,3]dioxole-5-carboxylate (**1i**)

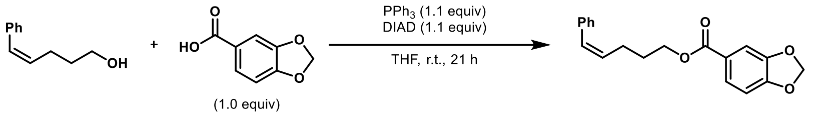

To a stirred solution of piperonylic acid (640 mg, 3.85 mmol, 1.1 equiv), PPh_3 (1.00 g, 3.85 mmol, 1.1 equiv), and (*Z*)-5-phenylpent-4-en-1-ol (568 mg, 3.50 mmol, 1.0 equiv) in THF (16 mL) was added DIAD (0.76 mL, 3.5 mmol, 1.0 equiv) at 0 °C. Then, the reaction mixture was warmed at room temperature. After 13 hours (unoptimized), H_2O (30 mL) was added, and the organic layer was separated. The aqueous layer was extracted with Et_2O (50 mL × 3), and the combined organic extracts were dried over $MgSO_4$ (7 g), filtered through a glass frit, and concentrated in vacuo. The crude material was purified by flash column chromatography (SiO_2, ϕ = 4 cm, l = 13 cm) eluting with EtOAc/hexanes = 1/10 (R_f = 0.30 [254 nm/$KMnO_4$]) to afford a pale-yellow oil (1.06 g, 97%). Further purification by Kugelrohr distillation (P = 0.15 mmHg, ABT = 270 °C) provided a colorless oil (1.05 g, 96%).

Data for **1i**: JYH-01-077

^{1}H NMR: (400 MHz, $CDCl_3$)

δ 7.58 (dd, *J* = 8.1, 1.7, 1H), 7.41 (d, *J* = 1.8, 1H), 7.32−7.26 (m, 4H), 7.23−7.19 (m, 1H), 6.81 (d, *J* = 8.2, 1H), 6.49 (d, *J* = 11.6, 1H), 6.04 (d, *J* = 2.7, 2H), 5.72−5.66 (m, 1H), 4.35−4.28 (m, 2H), 2.52−2.46 (m, 2H), 1.94−1.87 (m, 2H).

^{13}C NMR: (100 MHz, $CDCl_3$)

δ 166.1, 151.7, 147.9, 137.6, 131.5, 130.1, 128.9, 128.4, 126.9, 125.5, 124.6, 109.7, 108.1, 102.0, 64.5, 29.1, 25.3.

HRMS (EI): [M]+ calcd for $C_{19}H_{18}O_4$: 310.1205; found: 310.1202.

(*Z*)-5-phenylpent-4-en-1-yl furan-2-carboxylate (**1j**)

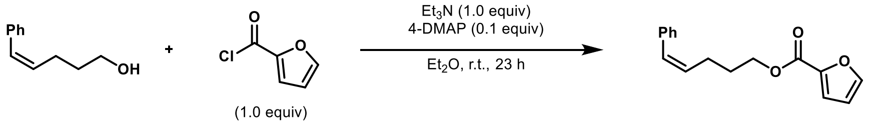

To a stirred solution of 4-DMAP (43 mg, 0.35 mmol, 0.1 equiv), (*Z*)-5-phenylpent-4-en-1-ol (568 mg, 3.50 mmol, 1.0 equiv, *Z* only), and Et_3N (0.49 mL, 3.5 mmol, 1.0 equiv) in Et_2O (3.5 mL) was added 2-furoyl chloride (0.35 mL, 3.5 mmol, 1.0 equiv) at room temperature. After 23 hours (unoptimized), H_2O (5 mL) was added, and the organic layer was separated. The aqueous layer was extracted with CH_2Cl_2 (5 mL × 3), and the combined organic extracts were dried over $MgSO_4$ (5.2 g), filtered through a glass frit, and concentrated in vacuo. The crude material was purified by flash column chromatography (SiO_2, ϕ = 4.0 cm, l = 11 cm) eluting with CH_2Cl_2/hexanes = 1/3 (R_f = 0.30 [254 nm/CAM(grey)) to afford

a colorless oil (820 mg, 91%). Further purification by Kugelrohr distillation (P = 0.5 mmHg, ABT = 270 °C) provided a colorless oil (797 mg, 89%, *Z/E* > 99/1).

Data for **1j**: HM-11-052

^{1}H NMR: (400 MHz, $CDCl_3$)
δ 7.57 (m, 1H), 7.33–7.25 (m, 4H), 7.22–7.18 (m, 1H), 7.10 (d, *J* = 3.7, 1H), 6.50–6.47 (m, 2H), 5.68 (dt, *J* = 11.6, 7.3, 1H), 4.32 (t, *J* = 6.4, 2H), 2.48 (m, 2H), 1.91 (appr. pent, *J* = 7.0, 2H).

^{13}C NMR: (100 MHz, $CDCl_3$)
δ 158.9, 146.4, 144.9, 137.5, 131.3, 130.2, 128.9, 128.4, 126.8, 118.0, 112.0, 64.5, 29.0, 25.1.

HRMS (EI): $[M]^+$ calcd for $C_{16}H_{16}O_3$: 256.1099; found: 256.1101.

(*Z*)-5-phenylpent-4-en-1-yl thiophene-2-carboxylate (**1k**)

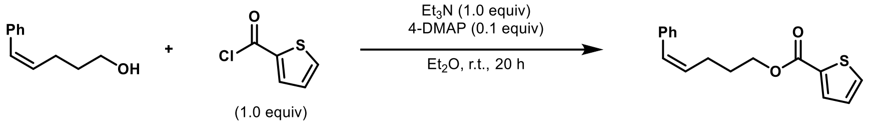

To a stirred solution of 4-DMAP (31 mg, 0.25 mmol, 0.1 equiv), (*Z*)-5-phenylpent-4-en-1-ol (406 mg, 2.50 mmol, 1.0 equiv, *Z* only), and Et_3N (0.35 mL, 2.5 mmol, 1.0 equiv) in Et_2O (2.5 mL) was added 2-thenoyl chloride (0.27 mL, 2.5 mmol, 1.0 equiv) at room temperature. After 20 hours (unoptimized), H_2O (5 mL) was added, and the organic layer was separated. The aqueous layer was extracted with CH_2Cl_2 (5 mL × 3), and the combined organic extracts were dried over $MgSO_4$ (2.87 g), filtered through a glass frit, and concentrated in vacuo. The crude material was purified by flash column chromatography (SiO_2, ϕ = 3.5 cm, l = 9.0 cm) eluting with CH_2Cl_2/hexanes = 1/3 (R_f = 0.20 [254 nm/CAM(grey)) to afford a colorless oil (705 mg, 103%). Further purification by Kugelrohr distillation (P = 0.1 mmHg, ABT = 250 °C) provided a colorless oil (648 mg, 95%, *Z/E* = 97/3).

Data for **1k**: HM-11-063

^{1}H NMR: (400 MHz, $CDCl_3$)
δ 7.73 (d, *J* = 3.7, 1H), 7.53 (d, *J* = 4.9, 1H), 7.31–7.27 (m, 4H), 7.22–7.19 (m, 1H), 7.10–7.08 (m, 1H), 6.49 (d, *J* = 11.6, 1H), 5.69 (dt, *J* = 11.6, 7.3, 1H), 4.32 (t, *J* = 6.4, 2H), 2.52–2.47 (m, 2H), 1.91 (appr. pent, *J* = 7.0, 2H).

^{13}C NMR: (100 MHz, $CDCl_3$)
δ 162.5, 137.6, 134.1, 133.5, 132.5, 131.4, 130.1, 128.9, 128.4, 127.9, 126.8, 64.7, 29.1, 25.2.

HRMS (EI): $[M]^+$ calcd for $C_{16}H_{16}O_2S$: 272.0871; found: 272.0873.

methyl (2*E*,6*Z*)-7-phenylhepta-2,6-dienoate (**1m**)

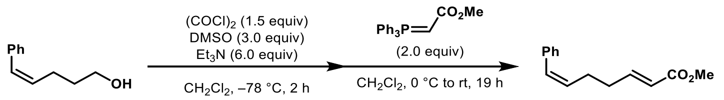

To a stirred solution of oxalylchloride (0.39 mL, 4.5 mmol, 1.5 equiv) in CH_2Cl_2 (13 mL) was added DMSO (0.64 mL, 9.0 mmol, 3.0 equiv) at −78 ˚C. To the reaction mixture, a solution of (*Z*)-5-phenylpent-4-en-1-ol (487 mg, 3.00 mmol, 1.0 equiv) in CH_2Cl_2 (1.5 mL), and Et_3N (2.5 mL, 18 mmol, 6.0 equiv) dropwisely. After 2 hours, the reaction mixture was warmed at 0 ˚C, then a solution of methyl(triphenylphosphoranylidene) acetate (2.01 g, 6.00 mmol, 2.0 equiv) in CH_2Cl_2 (6 mL) was added. After 19 hours (unoptimised), H_2O (20 mL) was added, and the organic layer was separated. The aqueous layer was extracted with Et_2O (30 mL × 3), and the combined organic extracts were dried over $MgSO_4$ (7 g), filtered through a glass frit, and concentrated in vacuo. The crude material was purified by flash column chromatography (SiO_2, ϕ = 3.5 cm, l = 13 cm) eluting with CH_2Cl_2/hexanes = 1/2 (R_f = 0.25 [254 nm/CAM (blue)]) to afford a pale-yellow oil (464 mg, 63%). Further purification by Kugelrohr distillation (P = 0.15 mmHg, ABT = 170 °C) provided a colorless oil (434 mg, 58%).

Data for **1m**: JYH-01-086

^{1}H NMR: (400 MHz, $CDCl_3$)
δ 7.38−7.18 (m, 5H), 6.97 (dt, *J* = 15.6, 6.8, 1H), 6.43 (d, *J* = 11.6, 1H), 5.86 (dt, *J* = 15.6, 1.6, 1H), 5.66−5.59 (m, 1H), 3.70 (s, 3H), 2.53−2.27 (m, 4H).

^{13}C NMR: (100 MHz, $CDCl_3$)
δ 167.2, 148.6, 137.5, 131.0, 130.2, 128.9, 128.4, 127.0, 121.6, 51.7, 32.6, 27.2.

HRMS (EI): $[M]^+$ calcd for $C_{14}H_{16}O_2$: 216.1150; found: 216.1147.

(*Z*)-2-(5-phenylpent-4-en-1-yl)isoindoline-1,3-dione (**1n**)

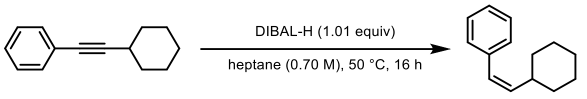

To a stirred solution of (cyclohexylethynyl)benzene (751 mg, 4.07 mmol, 1.0 equiv) in heptane (5.8 mL) was added DIBAL-H (1.0 M in hexane, 4.12 mL, 4.12 mmol, 1.01 equiv). Then, the reaction mixture was heated to 50 °C. After 16 hours (unoptimized), the reaction mixture was cooled at room temperature. sat. Rochelle salt solution (60 mL) was added, and the organic layer was separated. The aqueous layer was extracted with hexanes (50 mL × 3), and the combined organic extracts were dried over $MgSO_4$ (5 g), filtered through a glass frit, and concentrated in vacuo. The crude material was purified by flash column chromatography (SiO_2, ϕ = 4 cm, l = 17 cm) eluting hexanes (R_f = 0.70 [254 nm/CAM(blue)]) to give a colorless oil (530 mg, 70%). Further purification by Kugelrohr distillation (P = 0.15 mmHg, ABT = 150 °C) provided a colorless oil (506 mg, 67%).

Data for **1n**:[6] JYH-01-096

^{1}H NMR: (400 MHz, $CDCl_3$)
δ 7.35–7.31 (m, 2H), 7.27–7.20 (m, 4H), 6.31 (d, *J* = 11.4, 1H), 5.49 (dd, *J* = 11.4, 10.1, 1H), 2.62–2.53 (m, 1H), 1.82–1.71 (m, 4H), 1.66 (dd, *J* = 10.8, 1.6, 1H).

2.3. Preparation of 4,7-Dimethoxythianthrene-*S*-oxide (**S3**)

4,7-dimethoxythianthrene (**S2**)

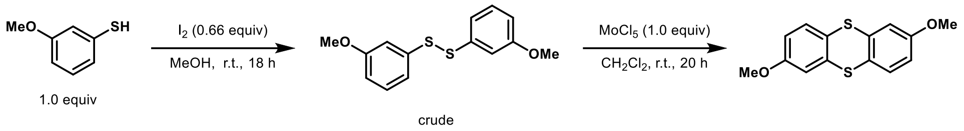

To a stirred solution of iodine (2.02 g, 7.97 mmol, 0.66 equiv) in MeOH (15 mL) was added 3-methoxyphenylmercaptan (1.50 mL, 12.1 mmol, 1.0 equiv) at room temperature. After 18 hours (unoptimized), sat. aq. Na_2SO_3 (10 mL) was added, and the separated organic layer was washed with Na_2SO_3 (10 mL × 2), H_2O (10 mL), dried over $MgSO_4$ (1.7 g), filtered through a glass frit, and concentrated in vacuo. To a solution of the crude material in CH_2Cl_2 (60 mL) was added $MoCl_5$ (3.31 g, 12.1 mmol, 1.0 equiv) at room temperature. After 20 hours (unoptimized), ice-cold water (50 mL) was added to the mixture. The separated aqueous layer was extracted with CH_2Cl_2 (30 mL × 3), and the combined organic layer was dried over $MgSO_4$ (7.54 g), filtered, and concentrated in vacuo. The crude material was purified by flash column chromatography (5.17g SiO_2, dry load, SiO_2, ϕ = 5.0 cm, l = 14.5 cm) eluting with CH_2Cl_2/hexanes = 1/1 (R_f = 0.55 [254 nm/CAM(blue)]) to afford an off-white solid (914 mg, 55%) and further recrystallization from CH_2Cl_2/hexanes gave **S2** as colorless needles (664 mg, 40%).

<u>Data for **S2**:[7]</u> HM-08-018

<u>^{1}H NMR:</u> (400 MHz, $CDCl_3$)

δ 7.36 (d, J = 8.5, 2H), 7.05 (d, J = 2.8, 2H), 6.79 (d, J = 8.2, 2H), 3.79 (s, 6H).

4,7-dimethoxythianthrene-*S*-oxide (**S3**)

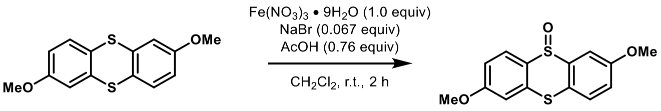

To a stirred solution of 4,7-methoxythianthrene (**S2**, 650 mg, 2.35 mmol, 1.0 equiv), $Fe(NO_3)_3$•$9H_2O$ (950 mg, 2.35 mmol, 1.0 equiv), and NaBr (16 mg, 0.16 mmol, 0.067 equiv) in CH_2Cl_2 (5.2 mL) was added acetic acid (0.10 mL, 1.8 mmol, 0.76 equiv) under air. After 2 hours, H_2O (50 mL) was added, and the organic layer was separated. The aqueous layer was extracted with CH_2Cl_2 (10 mL × 3), and the combined organic extracts were dried over $MgSO_4$ (2.7 g), filtered through a glass frit, and concentrated in vacuo. The crude material was purified by Kugelrohr distillation (P = 0.15 mmHg, ABT = 300 °C) to give a yellow solid (612 mg, 89%). Further purification by recrystallization from CH_2Cl_2/hexanes provided pale yellow needles (544 mg, 79%).

<u>Data for **S3**:</u> HM-10-064

<u>^{1}H NMR:</u> (400 MHz, $CDCl_3$)

δ 7.77 (d, J = 8.5, 1H), 7.47 (d, J = 8.5, 1H), 7.43 (d, J = 2.8, 1H), 7.14 (d, J = 2.1, 1H), 7.04 (dd, J = 8.5, 2.4, 1H), 6.94 (dd, J = 8.5, 2.8, 1H), 3.87 (s, 3H), 3.83 (s, 3H).

<u>^{13}C NMR:</u> (100 MHz, $CDCl_3$)
δ 161.1, 160.6, 143.9, 132.6, 131.0, 130.4, 125.9, 119.3, 117.4, 114.6, 114.4, 108.7, 56.1, 56.0.
<u>HRMS (EI):</u> $[M]^+$ calcd for $C_{14}H_{12}O_3S_2$: 292.0228; found: 292.0231.

2.4. Synthesis of (4*R**,5*S**)-4-Bromo-5-chloro-5-phenylpentyl Benzoate (**5a**)

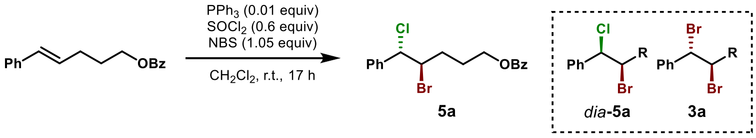

To a stirred solution of alkene (*E*-**1a**, 266 mg, 1.00 mmol, 1.0 equiv), PPh_3 (3 mg, 0.01 mmol, 0.01 equiv), and (187 mg, 1.05 mmol, 1.05 equiv) in CH_2Cl_2 (2 mL) was added $SOCl_2$ (44 µL, 0.60 mmol, 0.6 equiv) at room temperature. After 17 hours (unoptimized), H_2O (5 mL) was added, and the organic layer was separated. The aqueous layer was extracted with CH_2Cl_2 (5 mL × 3), and the combined organic extracts were dried over $MgSO_4$ (2.25 g), filtered through a glass frit, and concentrated in vacuo. The crude material was purified by flash column chromatography (SiO_2, ϕ = 3.0 cm, l = 11 cm) eluting with CH_2Cl_2/hexanes = 1/2 (R_f = 0.25 [254 nm/$KMnO_4$]) to afford **5a** (334 mg, 87%, 92:8 dr, >99:1 rr) as white solid.

<u>Data for (4*R**,5*S**)-4-bromo-5-chloro-5-phenylpentyl benzoate (**5a**):</u> HM-12-026
<u>^{1}H NMR:</u> (400 MHz, $CDCl_3$)
δ 8.08–8.03 (m, 2H), 7.59–7.55 (m, 1H), 7.47–7.33 (m, 7H), 5.05 (d, J = 9.2, 1H), 4.48–4.43 (m, 1H), 4.41–4.38 (m, 2H), 2.52–2.45 (m, 1H), 2.24–2.08 (m, 2H), 2.02–1.95 (m, 1H).
5.15 (d, J = 5.8, 0.085H, the *dia*-**5a**).
5.10 (d, J = 10.4, 0.081H, the dibromide **3a**).
<u>^{13}C NMR:</u> (100 MHz, $CDCl_3$)
δ 166.7, 139.5, 133.2, 130.3, 129.8, 129.1, 128.8, 128.6, 127.8, 66.0, 64.1, 58.5. 32.1, 26.5.
<u>HRMS (FAB):</u> $[M+H]^+$ calcd for $C_{18}H_{19}{}^{79}Br^{35}ClO_2$, $[C_{18}H_{19}{}^{81}Br^{35}ClO_2 + C_{18}H_{19}{}^{79}Br^{37}ClO_2]$: 381.0257 (76.0%), 383.0236 (100.0%); found: 381.0253 (80.6%), 383.0245 (100.0%).

2.5. General Procedure I: *syn*-Dibromination of Alkenes

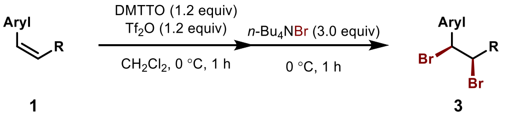

To a stirred solution of alkene (**1**, 1.0 mmol, 1.0 equiv) and 4,7-dimethoxythianthrene-*S*-oxide (351 mg, 1.20 mmol, 1.2 equiv) in CH_2Cl_2 (4 mL) was added Tf_2O (200 µL, 1.2 mmol, 1.2 equiv) at 0 °C. After 1 hour, a solution of *n*-Bu_4NBr (967 mg, 3.00 mmol, 3.0 equiv) in CH_2Cl_2 (8 mL) was added. After 1 hour, the reaction mixture was concentrated in vacuo and the residue was diluted with Et_2O (5 mL), filtered through a pad of SiO_2 (ϕ = 1.5 cm, l = 3.0 cm, Et_2O, 150 mL), and

concentrated in vacuo. The crude material was purified by flash column chromatography on SiO_2 to afford dibromide. Chromatographed products were further purified by recrystallization.

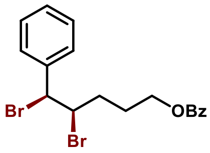

Flash column chromatography (SiO_2, ϕ = 5.0 cm, l = 12 cm, CH_2Cl_2/hexanes = 1/2, R_f = 0.25 [254 nm/CAM (grey)], 281 mg, 66%, >99:1 dr) and recrystallization (hexanes) afforded **3a** (262 mg, 61%, >99:1 dr) as colorless needles.

Data for (4*R**,5*S**)-4,5-dibromo-5-phenylpentyl benzoate (**3a**): HM-10-094

m.p.: 113.6 – 114.2 °C

^{1}H NMR: (400 MHz, $CDCl_3$)
δ 8.07–8.05 (m, 2H), 7.61–7.56 (m, 1H), 7.47–7.43 (m, 2H), 7.39–7.31 (m, 5H), 5.10 (d, J = 10.4, 1H), 4.63–4.57 (m, 1H), 4.47–4.41 (m, 2H), 2.70–2.63 (m, 1H), 2.24–2.14 (m, 2H), 2.08–1.98 (m, 1H).

^{13}C NMR: (100 MHz, $CDCl_3$)
δ 166.8, 140.5, 133.2, 130.4, 129.8, 129.1, 128.9, 128.6, 128.0, 64.2, 57.5, 57.2, 34.1, 26.4.

HRMS (FAB): $[M+H]^+$ calcd for $C_{18}H_{19}{}^{79}Br^{79}BrO_2$, $C_{18}H_{19}{}^{79}Br^{81}BrO_2$, $C_{18}H_{19}{}^{81}Br^{81}BrO_2$: 424.9752 (50.8%), 426.9732 (100.0%), 428.9715 (50.4%); found: 424.9756 (58.2%), 426.9710 (100.0%), 428.9688 (44.8%).

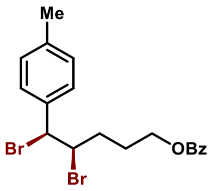

Flash column chromatography (SiO_2, ϕ = 5.0 cm, l = 13 cm, CH_2Cl_2/hexanes = 1/2, R_f = 0.35 [254 nm/CAM (grey)], 320 mg, 73%, 96:4 dr) and recrystallization (hexanes) afforded **3b** (301 mg, 68%, >99:1 dr) as colorless needles.

Data for (4*R**,5*S**)-4,5-dibromo-5-(*p*-tolyl)pentyl benzoate (**3b**): HM-10-096

m.p.: 117.6 – 118.1 °C

^{1}H NMR: (400 MHz, $CDCl_3$)
δ 8.07–8.05 (m, 2H), 7.57 (t, J = 7.5, 1H), 7.45 (t, J = 7.6, 2H), 7.28–7.26 (m, 2H), 7.17 (d, J = 7.9, 2H), 5.09 (d, J = 10.4, 1H), 4.62–4.57 (m, 1H), 4.42 (t, J = 6.1, 2H), 2.70–2.63 (m, 1H), 2.35 (s, 3H), 2.22–2.16 (m, 2H), 2.08–1.98 (m, 1H).

^{13}C NMR: (100 MHz, $CDCl_3$)
δ 166.8, 139.1, 137.7, 133.2, 130.4, 129.8, 129.6, 128.6, 127.8, 64.2, 57.6, 57.4, 34.2, 26.4, 21.5.

HRMS (ESI): $[M+Na]^+$ calcd for $C_{19}H_{20}{}^{79}Br^{79}BrNaO_2$, $C_{19}H_{20}{}^{79}Br^{81}BrNaO_2$, $C_{19}H_{20}{}^{81}Br^{81}BrNaO_2$: 460.9728 (51.3%), 462.9707 (100.0%), 464.9687 (48.8%); found: 460.9720 (49.6%), 462.9700 (100.0%), 464.9680 (46.2%).

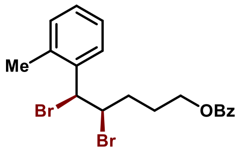

Flash column chromatography three times (SiO_2, ϕ = 5.0 cm, l = 11 cm, CH_2Cl_2/hexanes = 1/3, R_f = 0.20 [254 nm/CAM(grey)], ϕ = 4.0 cm, l = 13 cm, CH_2Cl_2/hexanes = 1/3, then ϕ = 3.0 cm, l = 13 cm, CH_2Cl_2/hexanes = 1/4) afforded **3c** (309 mg, 70%, 95:5 dr) as a colorless oil.

Data for (4*R**,5*S**)-4,5-dibromo-5-(*o*-tolyl)pentyl benzoate (**3c**): HM-11-022

^{1}H NMR: (400 MHz, $CDCl_3$)
δ 8.09–8.07 (m, 2H), 7.59–7.55 (m, 1H), 7.48–7.39 (m, 3H), 7.27–7.13 (m, 3H), 5.38 (d, J = 11.0, 1H), 4.68 (ddd, J = 11.3, 8.9, 2.8, 1H), 4.45 (t, J = 6.0, 2H), 2.79–2.70 (m, 1H), 2.41 (s, 3H), 2.28–2.16 (m, 2H), 2.10–2.00 (m, 1H).
5.47 (d, J = 7.6, 0.0561H, the *dia*-**3c**).

^{13}C NMR: (100 MHz, $CDCl_3$)
δ 166.8, 138.9, 135.6, 133.2, 130.9, 130.5, 129.8, 128.8, 128.6, 127.3, 127.0, 64.2, 57.0, 52.9, 34.3, 26.5, 19.6.

HRMS (ESI): $[M+Na]^+$ calcd for $C_{19}H_{20}{}^{79}Br^{79}BrNaO_2$, $C_{19}H_{20}{}^{79}Br^{81}BrNaO_2$, $C_{19}H_{20}{}^{81}Br^{81}BrNaO_2$: 460.9728 (51.3%), 462.9707 (100.0%), 464.9687 (48.8%); found: 460.9720 (50.8%), 462.9701 (100.0%), 464.9682 (48.9%).

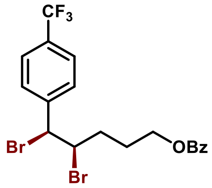

Flash column chromatography twice (SiO_2, ϕ = 3.5 cm, l = 15 cm, CH_2Cl_2/hexanes = 1/2, R_f = 0.20 [254 nm/CAM(grey)], then ϕ = 4.0 cm, l = 17 cm, CH_2Cl_2/hexanes = 1/2, 203 mg, 41%, 95:5 dr) and recrystallization (hexanes) afforded **3d** (193 mg, 39%, >99:1 dr) as white solid.

Data for (4*R**,5*S**)-4,5-dibromo-5-(4-(trifluoromethyl)phenyl)pentyl benzoate (**3d**): HM-11-062

^{1}H NMR: (400 MHz, $CDCl_3$)
δ 8.07 (d, J = 7.3, 2H), 7.63–7.60 (m, 2H), 7.58–7.56 (m, 1H), 7.50–7.44 (m, 4H), 5.09 (d, J = 11.0, 1H), 4.57 (ddd, J = 11.0, 8.5, 2.4, 1H), 4.48–4.39 (m, 2H), 2.72–2.63 (m, 1H), 2.26–2.13 (m, 2H), 2.09–2.00 (m, 1H).

^{13}C NMR: (100 MHz, $CDCl_3$)
δ 166.7, 144.3, 133.2, 131.0 (q, J_{CF3} = 32.6), 130.3, 129.8, 128.6, 128.4, 125.9 (d, J_{CF3} = 3.8), 124.0 (q, J_{CF3} = 272.2), 64.0, 56.6, 55.4, 34.0, 26.3.

^{19}F NMR: (376 MHz, $CDCl_3$)
δ –65.9.

HRMS (FAB): $[M+H]^+$ calcd for $C_{19}H_{18}{}^{79}Br^{79}BrF_3O_2$, $C_{19}H_{18}{}^{81}Br^{79}BrF_3O_2$, $C_{19}H_{18}{}^{81}Br^{81}BrF_3O_2$: 492.9626 (50.7%), 494.9606 (100.0%), 496.9589 (50.5%); found: 492.9625 (57.8%), 494.9593 (100.0%), 496.9700 (44.6%).

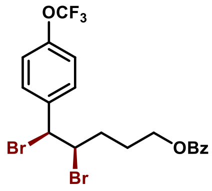

Flash column chromatography (SiO_2, ϕ = 3 cm, l = 12 cm, CH_2Cl_2/hexanes = 1/4 to 1/2, R_f = 0.30 [254 nm/CAM(grey)], 199 mg, 39%, >99:1 dr) and recrystallization (CH_2Cl_2/hexanes) afforded **3e** (159 mg, 31%, >99:1 dr) as white solid.

Data for (4*R**,5*S**)-4,5-dibromo-5-(4-(trifluoromethoxy)phenyl)pentyl benzoate (**3e**): HM-13-044

^{1}H NMR: (400 MHz, $CDCl_3$)

δ 8.06 (d, J = 7.9, 2H), 7.58 (t, J = 7.2, 1H), 7.46 (t, J = 7.6, 2H), 7.41 (d, J = 8.9, 2H), 7.20, (d, J = 8.5, 2H), 5.07 (d, J = 10.4, 1H), 4.47–4.52 (m, 1H), 4.48–4.38 (m, 2H), 2.70–2.62 (m, 1H), 2.25–2.12 (m, 2H), 2.08–2.02 (m, 1H).

^{13}C NMR: (100 MHz, $CDCl_3$)

δ 166.8, 149.4, 139.2, 133.3, 130.4, 129.8, 129.6, 128.6, 121.1, 120.6 (q, J_{OCF3} = 257.8), 64.1, 57.2, 55.7, 34.1, 26.4.

^{19}F NMR: (376 MHz, $CDCl_3$)

δ –61.0.

HRMS (ESI): $[M+Na]^+$ calcd for $C_{19}H_{17}{}^{79}Br{}^{79}BrF_3NaO_3$, $C_{19}H_{17}{}^{81}Br{}^{79}BrF_3NaO_3$, $C_{19}H_{17}{}^{81}Br{}^{81}BrF_3NaO_3$: 530.9394 (51.4%), 532.9374 (100.0%), 534.9353 (48.6%); found: 530.9384 (51.1%), 532.9367 (100.0%), 534.9344 (46.5%).

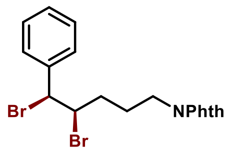

Flash column chromatography (SiO_2, ϕ = 4.0 cm, l = 11 cm, CH_2Cl_2/hexanes = 1/1, R_f = 0.20 [254 nm/CAM(grey)], 429 mg, 95% (73% **3f**), 97:3 dr, +29% *E* alkene) and recrystallization (CH_2Cl_2/hexanes) afforded **3f** (280 mg, 62% (60% **3f**), 98:2 dr, +3% *E* alkene) as white solid.

Data for 2-((4*R**,5*S**)-4,5-dibromo-5-phenylpentyl)isoindoline-1,3-dione (**3f**): HM-11-067

^{1}H NMR: (400 MHz, $CDCl_3$)

δ 7.88–7.81 (m, 2H), 7.74–7.67 (m, 2H), 7.38–7.28 (m, 5H), 5.04 (d, J = 10.4, 1H), 4.61–4.55 (m, 1H), 3.85–3.74 (m, 2H), 2.54–2.43 (m, 1H), 2.18–2.02 (m, 2H), 2.00–1.87 (m, 1H).

5.23 (d, J = 6.1, 0.020H, the *dia*-**3f**)

^{13}C NMR: (100 MHz, $CDCl_3$)

δ 168.6, 140.6, 134.2, 132.4, 129.0, 128.8, 128.0, 123.5, 57.2, 57.1, 37.2, 34.5, 26.2.

HRMS (ESI): $[M+Na]^+$ calcd for $C_{19}H_{17}{}^{79}Br{}^{79}BrNNaO_2$, $C_{19}H_{17}{}^{79}Br{}^{81}BrNNaO_2$, $C_{19}H_{17}{}^{81}Br{}^{81}BrNNaO_2$: 471.9524 (51.3%), 473.9503 (100.0%), 475.9483 (48.8%); found: 471.9518 (49.4%), 473.9500 (100.0%), 475.9483 (48.6%).

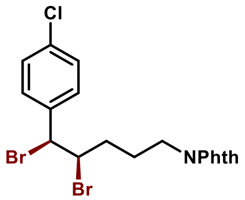

Flash column chromatography three times (SiO_2, ϕ = 5.0 cm, l = 10 cm, CH_2Cl_2/hexanes = 1/1, R_f = 0.25 [254 nm/CAM(grey)], 359 mg, 74% (43% **3g**), 98:2 dr, +42% *E* alkene) and recrystallization (CH_2Cl_2/hexanes) afforded **3g** (232 mg, 48% (38% **3g**), >99:1 dr, +14% *E* alkene) as white solid.

Data for 2-((4*R**,5*S**)-4,5-dibromo-5-(4-chlorophenyl)pentyl)isoindoline-1,3-dione (**3g**): HM-11-074

^{1}H NMR: (400 MHz, $CDCl_3$)

δ 7.88–7.82 (m, 2H), 7.73–7.69 (m, 2H), 7.33–7.28 (m, 4H), 4.99 (d, *J* = 10.5, 1H), 4.52 (ddd, *J* = 11.0, 9.2, 2.8, 1H), 3.80–3.75 (m, 2H), 2.49–2.42 (m, 1H), 2.16–1.84 (m, 3H).

^{13}C NMR: (100 MHz, $CDCl_3$)

δ 168.5, 139.1, 134.6, 134.2, 132.1, 129.3, 129.0, 123.4, 56.8, 55.9, 37.0, 34.2, 26.0.

HRMS (ESI): $[M+Na]^+$ calcd for $C_{19}H_{16}{}^{79}Br^{79}Br^{35}ClNNaO_2$, [$C_{19}H_{16}{}^{81}Br^{79}Br^{35}ClNNaO_2$ + $C_{19}H_{16}{}^{79}Br^{79}Br^{37}ClNNaO_2$], [$C_{19}H_{16}{}^{81}Br^{81}Br^{35}ClNNaO_2$ + $C_{19}H_{16}{}^{81}Br^{79}Br^{37}ClNNaO_2$], $C_{19}H_{16}{}^{81}Br^{81}Br^{37}ClNNaO_2$: 505.9134 (51.3%), 507.9114 (100.0%), [509.9093 (48.6%) + 509.9084 (32.0%)], 511.9064 (15.6%); found: 505.9126 (42.6%), 507.9108 (100.0%), 509.9085 (67.0%), 511.9061 (12.6%).

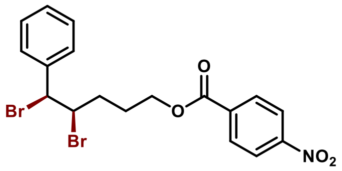

Flash column chromatography (SiO_2, ϕ = 4.0 cm, l = 13 cm, CH_2Cl_2/hexanes = 1/2, R_f = 0.15 [254 nm/CAM(grey)], 430 mg, 91% (82% **3h**), 98:2 dr, +14% *E* alkene) and recrystallization (CH_2Cl_2/hexanes) afforded **3h** (354 mg, 75%, >99:1 dr) as colorless crystals.

Data for (4*R**,5*S**)-4,5-dibromo-5-phenylpentyl 4-nitrobenzoate (**3h**): HM-11-032

m.p.: 117.7 – 118.2 °C

^{1}H NMR: (400 MHz, $CDCl_3$)

δ 8.30 (d, *J* = 8.9, 2H), 8.23 (d, *J* = 9.2, 2H), 7.38–7.32 (m, 5H), 5.10 (d, *J* = 10.7, 1H), 4.61–4.56 (m, 1H), 4.48 (t, *J* = 6.1, 2H), 2.70–2.63 (m, 1H), 2.28–2.16 (m, 2H), 2.11–2.02 (m, 1H).

^{13}C NMR: (100 MHz, $CDCl_3$)

δ 164.9, 150.8, 140.4, 135.8, 130.9, 129.1, 128.9, 128.0, 123.8, 65.1, 57.24, 57.15, 34.0, 26.3.

HRMS (ESI): $[M+Na]^+$ calcd for $C_{18}H_{17}{}^{79}Br^{79}BrNNaO_4$, $C_{18}H_{17}{}^{81}Br^{81}BrNNaO_4$, $C_{18}H_{17}{}^{81}Br^{81}BrNNaO_4$: 491.9422 (51.3%), 493.9402 (100.0%), 495.9381 (48.8%); found: 491.9420 (48.1%), 493.9400 (100.0%), 495.9380 (50.9%).

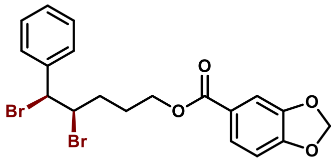

Flash column chromatography (SiO_2, ϕ = 3.5 cm, l = 11 cm, CH_2Cl_2/hexanes = 1/3 to 1/1, R_f = 0.40 (1/1) [254 nm/CAM(grey)], 438 mg, 93% (85% **3i**), >99:1 dr, +9% *E* alkene) and recrystallization (hexanes) afforded **3i** (378 mg, 80%, >99:1 dr) as white solid.

Data for (4*R**,5*S**)-4,5-dibromo-5-phenylpentyl benzo[*d*][1,3]dioxole-5-carboxylate (**3i**): HM-11-037

^{1}H NMR: (400 MHz, $CDCl_3$)

δ 7.67 (dd, J = 8.2, 1.5, 1H), 7.48 (d, J = 1.8, 1H), 7.39–7.31 (m, 5H), 6.85 (d, J = 7.9, 1H), 6.05 (s, 2H), 5.09 (d, J = 10.7, 1H), 4.59 (ddd, J = 10.7, 8.9, 2.8, 1H), 4.38 (t, J = 6.1, 2H), 2.68–2.60 (m, 1H), 2.23–2.12 (m, 2H), 2.07–1.95 (m, 1H).

^{13}C NMR: (100 MHz, $CDCl_3$)

δ 166.1, 151.9, 148.0, 140.5, 129.1, 128.9, 128.0, 125.6, 124.4, 109.7, 108.2, 102.0, 64.1, 57.4, 57.2, 34.1, 26.4.

HRMS (FAB): $[M]^+$ calcd for $C_{19}H_{18}{}^{79}Br^{79}BrO_4$, $C_{19}H_{18}{}^{81}Br^{79}BrO_4$, $C_{19}H_{18}{}^{81}Br^{81}BrO_4$: 467.9572 (51.3%), 469.9552 (100.0%), 471.9531 (48.8%); found: 467.9574 (48.6%), 469.9507 (100.0%), 471.9499 (54.1%).

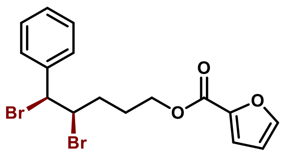

Flash column chromatography (SiO_2, ϕ = 4.0 cm, l = 13 cm, CH_2Cl_2/hexanes = 1/3, R_f = 0.20 [254 nm/CAM(grey)], 366 mg, 88% (77% **3j**), 98:2 dr, +19% *E* alkene) and recrystallization (hexanes) afforded **3j** (292 mg, 70%, >99:1 dr) as white solid.

Data for (4*R**,5*S**)-4,5-dibromo-5-phenylpentyl furan-2-carboxylate (**3j**): HM-11-057

^{1}H NMR: (400 MHz, $CDCl_3$)

δ 7.60–7.59 (m, 1H), 7.39–7.30 (m, 5H), 7.20 (dd, J = 3.4, 0.6, 1H), 6.52 (dd, J = 3.7, 1.8, 1H), 5.08 (d, J = 10.4, 1H), 4.59 (ddd, J = 10.7, 8.5, 2.4, 1H), 4.45–4.35 (m, 2H), 2.65–2.59 (m, 1H), 2.20–2.10 (m, 2H), 2.06–1.95 (m, 1H).

^{13}C NMR: (100 MHz, $CDCl_3$)

δ 158.9, 146.6, 144.8, 140.5, 129.0, 128.9, 128.0, 118.2, 112.1, 64.1, 57.4, 57.2, 33.9, 26.4.

HRMS (FAB): $[M+H]^+$ calcd for $C_{16}H_{17}{}^{79}Br^{79}BrO_3$, $C_{16}H_{17}{}^{81}Br^{79}BrO_3$, $C_{16}H_{17}{}^{81}Br^{81}BrO_3$: 414.9544 (50.8%), 416.9525 (100.0%), 418.9507 (50.2%); found: 414.9545 (57.0%), 416.9509 (100.0%), 418.9468 (47.8%).

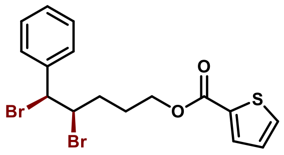

Flash column chromatography twice (SiO_2, ϕ = 4.0 cm, l = 11 cm, CH_2Cl_2/hexanes = 1/3, R_f = 0.15 [254 nm/CAM(grey)], then ϕ = 3.5 cm, l = 9 cm, CH_2Cl_2/hexanes = 1/3, 382 mg, 88% (78% **3k**), >99:1 dr, +17% *E* alkene) and recrystallization (hexanes) afforded **3k** (311 mg, 72%, >99:1 dr) as white solid.

Data for (4*R**,5*S**)-4,5-dibromo-5-phenylpentyl thiophene-2-carboxylate (**3k**): HM-11-064

^{1}H NMR: (400 MHz, $CDCl_3$)

δ 7.82 (dd, J = 3.7, 1.2, 1H), 7.57 (dd, J = 5.5, 1.2, 1H), 7.40–7.29 (m, 5H), 7.11 (dd, J = 4.9, 3.7, 1H), 5.09 (d, J = 10.4, 1H), 4.59 (ddd, J = 11.0, 8.5, 2.4, 1H), 4.45–4.34 (m, 2H), 2.69–2.61 (m, 1H), 2.21–2.12 (m, 2H), 2.06–1.94 (m, 1H).

^{13}C NMR: (100 MHz, $CDCl_3$)

δ 162.4, 140.6, 134.1, 133.7, 132.6, 129.0, 128.9, 128.1, 128.0, 64.3, 57.4, 57.3, 34.1, 26.5.

HRMS (FAB): $[M+H]^+$ calcd for $C_{16}H_{17}{}^{79}Br^{79}BrO_2S$, $C_{16}H_{17}{}^{81}Br^{79}BrO_2S$, $C_{16}H_{17}{}^{81}Br^{81}BrO_2S$: 430.9316 (49.7%), 432.9296 (100.0%), 434.9276 (53.4%); found: 430.9318 (52.3%), 432.9225 (100.0%), 434.9174 (56.8%).

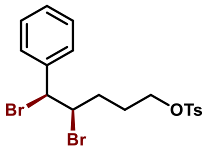

Flash column chromatography (SiO_2, ϕ = 3.5 cm, l = 12 cm, CH_2Cl_2/hexanes = 1/1, R_f = 0.25 [254 nm/CAM(blue)], 425 mg, 89% (68% **3l**), >99:1 dr, +31% *E* alkene) and recrystallization (hexanes) afforded **3l** (311 mg, 65%, >99:1 dr) as white solid.

Data for (4*R**,5*S**)-4,5-dibromo-5-phenylpentyl 4-methylbenzenesulfonate (**3l**): HM-13-025

^{1}H NMR: (400 MHz, $CDCl_3$)
δ 7.83–7.81 (m, 2H), 7.38–7.32 (m, 7H), 4.99 (d, *J* = 10.7, 1H), 4.46–4.04 (m, 1H), 4.17–4.10 (m, 2H), 2.57–2.45 (m, 4H), 2.12–1.81 (m, 3H).

^{13}C NMR: (100 MHz, C_6D_6)
δ 145.1, 140.3, 133.2, 130.2, 129.1, 128.9, 128.2, 127.9, 69.6, 57.0, 56.95, 33.5, 26.8, 21.9.

HRMS (ESI): $[M+Na]^+$ calcd for $C_{18}H_{20}{}^{79}Br^{79}BrNaO_3S$, $C_{18}H_{20}{}^{81}Br^{79}BrNaO_3S$, $C_{18}H_{20}{}^{81}Br^{81}BrNaO_3S$: 496.9398 (51.4%), 498.9377 (100.0%), 500.9357 (48.6%); found: 496.9395 (48.3%), 498.9376 (100.0%), 500.9355 (50.0%).

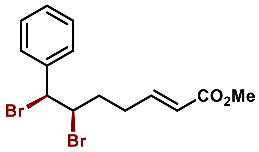

Flash column chromatography (SiO_2, ϕ = 4.0 cm, l = 12 cm, CH_2Cl_2/hexanes = 1/2, R_f = 0.10 [254 nm/CAM(grey)], 288 mg, 77% (67% **3m**). 67%, 99:1 dr, +16% *E* alkene) and recrystallization (hexanes) afforded **3m** (232 mg, 62%, >99:1 dr) as white solid.

Data for methyl (6*R**,7*S**,*E*)-6,7-dibromo-7-phenylhept-2-enoate (**3m**): HM-11-079

^{1}H NMR: (400 MHz, $CDCl_3$)
δ 7.38–7.30 (m, 5H), 7.00 (ddd, *J* = 15.6, 7.3, 5.5, 1H), 5.96–5.92 (m, 1H), 5.07 (d, *J* = 10.1, 1H), 4.50–4.45 (m, 1H), 3.75 (s, 3H), 2.69–2.59 (m, 2H), 2.53–2.45 (m, 1H), 2.17–2.09 (m, 1H).

^{13}C NMR: (100 MHz, $CDCl_3$)
δ 167.0, 147.1, 140.4, 129.1, 128.9, 128.0, 122.5, 57.2, 57.1, 51.8, 35.6, 29.8.

HRMS (FAB): $[M+H]^+$ calcd for $C_{14}H_{17}{}^{79}Br^{79}BrO_2$, $C_{14}H_{17}{}^{81}Br^{79}O_2$, $C_{14}H_{17}{}^{81}Br^{81}O_2$: 374.9595 (51.0%), 376.9575 (100.0%), 378.9557 (49.8%); found: 374.9597 (55.3%), 376.9597 (100.0%), 378.9575 (46.4%).

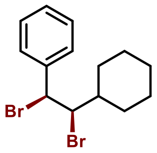

Flash column chromatography (SiO_2, ϕ = 4.0 cm, l = 14 cm, *n*-pentane, R_f = 0.30 [254 nm/CAM(blue)]) afforded **3n** (159 mg, 46%, >99:1 dr) as white solid.

Data for ((1*S**,2*R**)-1,2-dibromo-2-cyclohexylethyl)benzene (**3n**): HM-11-097

^{1}H NMR: (400 MHz, $CDCl_3$)
δ 7.40–7.30 (m, 5H), 5.16 (d, *J* = 11.9, 1H), 4.54 (dd, *J* = 11.5, 2.1, 1H), 2.32–2.28 (m, 1H), 1.90–1.16 (m, 10H).

^{13}C NMR: (100 MHz, C_6D_6)
δ 141.5, 128.8, 128.7, 128.2, 65.4, 55.2, 41.5, 33.1, 26.5, 26.4, 25.95, 25.91.

EA: Anal. Calcd for $C_{14}H_{18}Br_2$: C, 48.58; H, 5.24. Found: C, 48.54; H, 5.36.

2.6. General Procedure II: *syn*-Bromochlorination of Alkenes

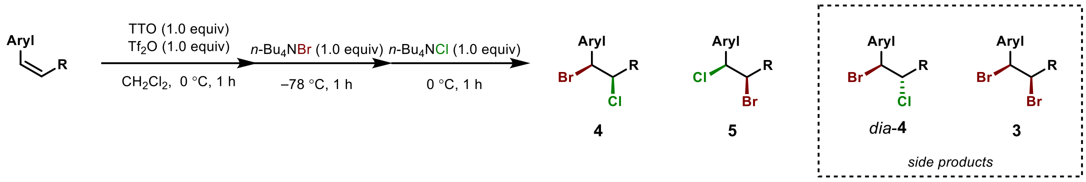

To a stirred solution of alkene (**1a**, 1.0 mmol, 1.0 equiv) and thianthrene-*S*-oxide (232 mg, 1.00 mmol, 1.0 equiv) in CH_2Cl_2 (4 mL) was added Tf_2O (170 µL, 1.0 mmol, 1.0 equiv) at 0 °C. After 1 hour, a solution of *n*-Bu_4NBr (322 mg, 1.00 mmol, 1.0 equiv) in CH_2Cl_2 (2.5 mL) was added at –78 ºC. After 1 hour, a solution of *n*-Bu_4NCl (278 mg, 1.00 mmol, 1.0 equiv) in CH_2Cl_2 (2.5 mL) was added at 0 ºC. After 1 hour, the reaction mixture was concentrated in vacuo and the residue was diluted with Et_2O (5 mL), filtered through a pad of SiO_2 (ϕ = 2.0 cm, l = 3.0 cm, Et_2O, 150 mL), and concentrated in vacuo. The crude material was purified by flash column chromatography on SiO_2 to afford bromochloride. Chromatographed products were further purified by recrystallization.

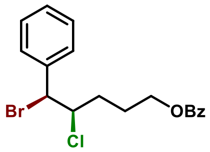

Flash column chromatography (SiO_2, ϕ = 3.0 cm, l = 12 cm, CH_2Cl_2/hexanes = 1/2, R_f = 0.30 [254 nm/CAM(blue)], 334 mg, 88% (62% **4a**), >99:1 dr, >99:1 rr +4% *E* alkene) and recrystallization (hexanes) afforded **4a** (290 mg, 76% (56% **4a**), >99:1 dr, >99:1 rr) as white solid.

Data for (4*R**,5*S**)-5-bromo-4-chloro-5-phenylpentyl benzoate (**4a**): JYH-02-011

^{1}H NMR: (400 MHz, $CDCl_3$)
δ 8.08–8.05 (m, 2H), 7.58 (t, *J* = 7.3, 1H), 7.48–7.31 (m, 7H), 5.07 (d, *J* = 9.2, 1H), 4.65–4.27 (m, 3H), 2.71–2.38 (m, 1H), 2.24–1.80 (m, 3H).
5.12 (d, *J* = 10.5, 0.12H, the dibromide **3a**).

^{13}C NMR: (100 MHz, $CDCl_3$)
δ 166.6, 139.4, 133.1, 130.3, 129.7, 129.0, 128.7, 128.5, 127.8, 66.0, 64.1, 58.0, 32.1, 26.4.

HRMS (FAB): $[M–Cl]^+$ calcd for $C_{18}H_{18}{}^{79}BrO_2$, $C_{18}H_{19}{}^{81}BrO_2$: 345.0490 (100.0%), 347.0470 (97.5%); found: 345.0493 (100.0%), 347.0478 (97.8%).
$[M+H]^+$ calcd for $C_{18}H_{19}{}^{79}Br^{35}ClO_2$, $[C_{18}H_{19}{}^{81}Br^{35}ClO_2 + C_{18}H_{19}{}^{79}Br^{37}ClO_2]$: 381.0257 (76.0%), 383.0236 (100.0%); found: 381.0253 (76.2%), 383.0238 (100.0%).

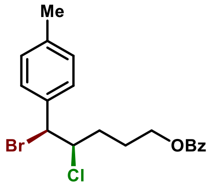

Flash column chromatography (SiO_2, ϕ = 3.0 cm, l = 12 cm, CH_2Cl_2/hexanes = 1/2, R_f = 0.30 [254 nm/CAM(blue)], 283 mg, 72% (49% **4b**), >99:1 dr, >99:1 rr) and recrystallization (CH_2Cl_2/hexanes) afforded **4b** (188 mg, 48% (38% **4b**), >99:1 dr, >99:1 rr) as white solid.

Data for (4*R**,5*S**)-5-bromo-4-chloro-5-(*p*-tolyl)pentyl benzoate (**4b**): JYH-02-027

^{1}H NMR: (400 MHz, $CDCl_3$)

δ 8.07–8.03 (m, 2H), 7.57 (t, *J* = 7.3, 1H), 7.45 (t, *J* = 7.8, 2H), 7.27 (d, *J* = 9.2, 2H), 7.17 (d, *J* = 7.9, 2H), 5.02 (d, *J* = 8.9, 1H), 4.47–4.35 (m, 3H), 2.53–2.45 (m, 1H), 2.35 (s, 3H), 2.23–1.92 (m, 3H).

5.09 (d, *J* = 10.7, 0.21H, the dibromide **3b**).

^{13}C NMR: (100 MHz, $CDCl_3$)

δ 166.7, 139.0, 136.6, 133.2, 129.8, 129.6, 129.5, 128.6, 127.7, 66.0, 64.2, 58.2, 32.2, 26.5, 21.4.

HRMS (FAB): $[M–Cl]^+$ calcd for $C_{19}H_{20}{}^{79}BrO_2$, $C_{19}H_{20}{}^{81}BrO_2$: 359.0647 (100.0%), 361.0628 (99.8%); found: 359.0650 (100.0%), 361.0624 (99.8%).

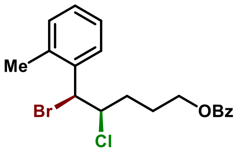

Flash column chromatography (SiO_2, ϕ = 3.5 cm, *l* = 11 cm, CH_2Cl_2/hexanes = 1/3, R_f = 0.20 [254 nm/CAM(blue)], 314 mg, 79% (62% **4c**), 97:3 dr, 97:3 rr +2% *E* alkene) afforded **4c** as a yellow oil.

Data for (4*R**,5*S**)-5-bromo-4-chloro-5-(*o*-tolyl)pentyl benzoate (**4c**): JYH-02-028

^{1}H NMR: (400 MHz, $CDCl_3$)

δ 8.06 (d, *J* = 7.0, 2H), 7.58 (t, *J* = 7.3, 1H), 7.46 (t, *J* = 7.5, 4H), 7.26–7.19 (m, 2H), 5.35 (d, *J* = 9.8, 1H), 4.54–4.41 (m, 3H), 2.63–2.56 (m, 1H), 2.43 (s, 3H), 2.23–1.99 (m, 3H).

5.48 (d, *J* = 7.9, 0.027H, the *dia*-**4c**).

5.39 (d, *J* = 10.7, 0.14H, the dibromide **3c**).

5.25 (d, *J* = 9.2, 0.034H, the constitutional isomer **5c**).

^{13}C NMR: (100 MHz, $CDCl_3$)

δ 166.7, 138.1, 135.8, 133.2, 130.8, 130.4, 129.8, 128.8, 128.6, 126.91, 126.87, 64.2, 61.5, 57.4, 32.4, 26.5, 19.7.

HRMS (FAB): $[M–Cl]^+$ calcd for $C_{19}H_{20}{}^{79}BrO_2$, $C_{19}H_{20}{}^{81}BrO_2$: 359.0647 (100.0%), 361.0628 (99.8%); found: 359.0649 (97.7%), 361.0671 (100.0%).

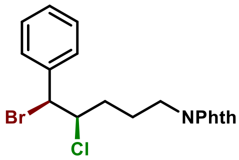

Flash column chromatography (SiO_2, ϕ = 3.5 cm, *l* = 14 cm, CH_2Cl_2/hexanes = 1/1, R_f = 0.20 [254 nm/CAM(blue)], 347 mg, 85% (65% **4f**), >99:1 dr, >99:1 rr, +7% *E* alkene) and recrystallization (CH_2Cl_2/hexanes) afforded a **4f** (290 mg, 71% (59% **4f**), >99:1 dr, >99:1 rr) as white solid.

Data for 2-((4*R**,5*S**)-5-bromo-4-chloro-5-phenylpentyl)isoindoline-1,3-dione (**4f**): HM-14-050-2

^{1}H NMR: (400 MHz, $CDCl_3$)

δ 7.87–7.82 (m, 2H), 7.74–7.70 (m, 2H), 7.38–7.29 (m, 5H), 4.98 (d, *J* = 9.2, 1H), 4.43 (td, *J* = 9.2, 2.8, 1H), 3.79–3.73 (m, 2H), 2.37–2.29 (m, 1H), 2.16–1.86 (m, 3H).

5.03 (d, *J* = 10.5, 0.12H, the dibromide **3f**).

^{13}C NMR: (100 MHz, $CDCl_3$)

δ 168.6, 139.5, 134.2, 132.2, 129.0, 128.7, 127.9, 123.5, 65.9, 57.7, 37.2, 32.6, 26.3.

HRMS (ESI): [M+Na]$^+$ calcd for $C_{19}H_{17}{}^{79}Br^{35}ClNNaO_2$, [$C_{19}H_{17}{}^{81}Br^{35}ClNNaO_2$ + $C_{19}H_{17}{}^{79}Br^{37}ClNNaO_2$], $C_{19}H_{17}{}^{81}Br^{37}ClNNaO_2$]: 428.0023 (77.3%), 430.0006 (100.0%), 431.9979 (24.1%); found: 428.0027 (75.4%), 430.0002 (100.0%), 431.9978 (23.8%).

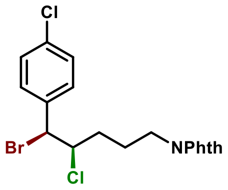

Flash column chromatography (SiO_2, ϕ = 3.5 cm, l = 14 cm, CH_2Cl_2/hexanes = 1/1, R_f = 0.30 [254 nm/CAM(blue)], 402 mg, 91% (50% **4g**), >99:1 dr, >99:1 rr +16% *E* alkene) and recrystallization (CH_2Cl_2/hexanes) afforded **4g** (255 mg, 58% (40% **4g**), >99:1 dr, >99:1 rr + 4% *E* alkene) as white solid.

Data for 2-((4*R**,5*S**)-5-bromo-4-chloro-5-(4-chlorophenyl)pentyl)isoindoline-1,3-dione (**4g**): JYH-02-024

^{1}H NMR: (400 MHz, $CDCl_3$)

δ 7.91–7.79 (m, 2H), 7.74–7.67 (m, 2H), 7.39–7.28 (m, 4H), 4.93 (d, J = 9.2, 1H), 4.55–4.35 (m, 1H), 3.84–3.61 (m, 2H), 2.51–2.25 (m, 1H), 2.17–1.63 (m, 3H).

4.99 (d, J = 10.4, 0.17H, the dibromide **3g**).

^{13}C NMR: (100 MHz, $CDCl_3$)

δ 168.6, 138.1, 134.8, 134.2, 132.2, 129.3, 129.0, 123.5, 65.0, 57.4, 37.1, 32.5, 26.2.

HRMS (ESI): [M+Na]$^+$ calcd for $C_{19}H_{16}{}^{79}Br^{35}Cl^{35}ClNNaO_2$, [$C_{19}H_{16}{}^{81}Br^{35}Cl^{35}ClNNaO_2$ + $C_{19}H_{16}{}^{79}Br^{37}Cl^{35}ClNNaO_2$], [$C_{19}H_{16}{}^{81}Br^{37}Cl^{35}ClNNaO_2$ + $C_{19}H_{16}{}^{79}Br^{37}Cl^{37}ClNNaO_2$], $C_{19}H_{16}{}^{81}Br^{37}Cl^{37}ClNNaO_2$: 461.9639 (62.0%), 463.9615 (100.0%), 465.9589 (38.6%), 467.9560 (6.2%); found: 461.9632 (60.6%), 463.9609 (100.0%), 465.9583 (45.1%), 467.9563 (6.6%).

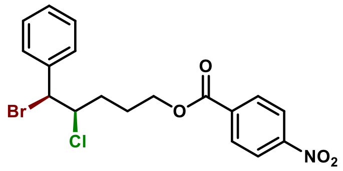

Flash column chromatography (SiO_2, ϕ = 3.5 cm, l = 12 cm, CH_2Cl_2/hexanes = 1/2, R_f = 0.20 [254 nm/CAM(blue)], 363 mg, 85% (65% **4h**), >99:1 dr, 98:2 rr +5% *E* alkene) and recrystallization (hexanes) afforded **4h** (290 mg, 68% (55% **4h**), >99:1 dr, 98:2 rr) as white solid.

Data for (4*R**,5*S**)-5-bromo-4-chloro-5-phenylpentyl 4-nitrobenzoate (**4h**): JYH-02-036

^{1}H NMR: (400 MHz, $CDCl_3$)

δ 8.29–8.17 (m, 4H), 7.43–7.18 (m, 5H), 5.05 (d, J = 9.2, 1H), 4.63–4.24 (m, 3H), 2.56–2.38 (m, 1H), 2.32–1.88 (m, 3H).

5.10 (d, J = 10.7, 0.12H, the dibromide **3h**).

4.96 (d, J = 8.2, 0.016H, the constitutional isomer **5h**).

^{13}C NMR: (100 MHz, $CDCl_3$)

δ 164.8, 150.8, 139.4, 135.7, 130.9, 129.2, 128.9, 127.8, 123.8, 66.0, 65.1, 57.7, 32.0, 26.4.

HRMS (FAB): [M–Cl]$^+$ calcd for $C_{18}H_{17}{}^{79}BrNO_4$, $C_{18}H_{17}{}^{81}BrNO_4$: 390.0341 (99.9%), 392.0323 (100.0%); found: 390.0343 (96.7%), 392.0312 (100.0%).

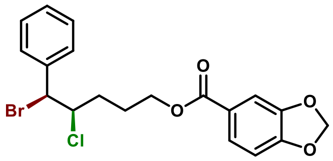

Flash column chromatography (SiO_2, ϕ = 5.0 cm, l = 11 cm, CH_2Cl_2/hexanes = 2/1, R_f = 0.40 [254 nm/CAM(blue)]) afforded **4i** (363 mg, 85% (56% **4i**), >99:1 dr, >99:1 rr, +8% *E* alkene) as a yellow oil.

Data for (4*R**,5*S**)-5-bromo-4-chloro-5-phenylpentyl benzo[*d*][1,3]dioxole-5-carboxylate (**4i**): HM-12-029

^{1}H NMR: (400 MHz, $CDCl_3$)
δ 7.64 (dd, *J* = 8.2, 1.8, 1H), 7.45 (d, *J* = 1.6, 1H), 7.39–7.31 (m, 5H), 6.84 (d, *J* = 7.8, 1H), 6.04 (s, 2H), 5.04 (d, *J* = 9.2, 1H), 4.43 (td, *J* = 9.2, 2.8, 1H), 4.35 (t, *J* = 6.2, 2H), 2.49–2.42 (m, 1H), 2.21–2.06 (m, 2H), 1.99–1.92 (m, 1H).
5.09 (d, *J* = 10.5, 0.14H, the dibromide **3i**).

^{13}C NMR: (100 MHz, $CDCl_3$)
δ 166.1, 151.9, 148.0, 139.5, 129.1, 128.8, 127.9, 125.6, 124.4, 109.7, 108.2, 102.0, 66.1, 64.1, 58.0, 32.2, 26.6.

HRMS (FAB): [M]$^+$ calcd for $C_{19}H_{18}{}^{79}Br^{35}ClO_4$, [$C_{19}H_{18}{}^{81}Br^{35}ClO_4$ + $C_{19}H_{18}{}^{79}Br^{37}ClO_4$], $C_{19}H_{18}{}^{81}Br^{37}ClO_4$: 424.0077 (75.6%), 426.0056 (100.0%), 428.0037 (26.4%); found: 424.0080 (75.7%), 426.0049 (100.0%), 428.0045 (28.3%).

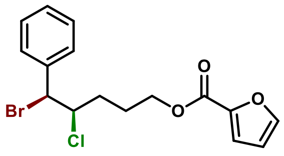

Flash column chromatography (SiO_2, ϕ = 5.0 cm, l = 11 cm, CH_2Cl_2/hexanes = 1/1, R_f = 0.35 [254 nm/CAM(blue)], 297 mg, 80% (62% **4j**), >99:1 dr, >99:1 rr, +6% *E* alkene) and recrystallization (CH_2Cl_2/hexanes) afforded **4j** (264 mg, 71% (58% **4j**), >99:1 dr, >99:1 rr) as white solid.

Data for (4*R**,5*S**)-5-bromo-4-chloro-5-phenylpentyl furan-2-carboxylate (**4j**): HM-12-021

^{1}H NMR: (400 MHz, $CDCl_3$)
δ 7.59–7.59 (m, 1H), 7.40–7.31 (m, 5H), 7.17–7.16 (m, 1H), 6.52 (dd, *J* = 3.7, 1.8, 1H), 5.03 (d, *J* = 9.2, 1H), 4.46–4.34 (m, 3H), 2.49–2.41 (m, 1H), 2.20–1.90 (m, 3H).
5.08 (d, *J* = 10.5, 0.21H, the dibromide **3j**).

^{13}C NMR: (100 MHz, $CDCl_3$)
δ 158.9, 146.6, 144.8, 139.5, 129.1, 128.8, 127.9, 118.2, 112.1, 66.0, 64.1, 58.0, 32.1, 26.5.

HRMS (FAB): [M–Cl]$^+$ calcd for $C_{16}H_{17}{}^{79}BrO_3$, $C_{16}H_{17}{}^{81}BrO_3$: 335.0283 (100.0%), 337.0263 (97.5%); found: 335.0286 (100.0%), 337.0259 (97.3%).
[M+H]$^+$ calcd for $C_{16}H_{17}{}^{79}Br^{35}ClO_3$, [$C_{16}H_{17}{}^{81}Br^{35}ClO_3$ + $C_{16}H_{17}{}^{79}Br^{37}ClO_3$]: 371.0050 (76.1%), 373.0028 (100.0%); found: 371.0052 (83.0%), 373.0024 (100.0%).

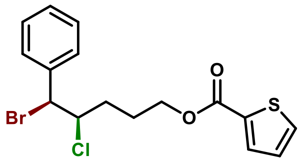

Flash column chromatography (SiO_2, ϕ = 4.0 cm, l = 9 cm, CH_2Cl_2/hexanes = 1/1, R_f = 0.35 [254 nm/CAM(blue)], 322 mg, 83% (65% **4k**), >99:1 dr, >99:1 rr, +6% *E* alkene) and recrystallization (CH_2Cl_2/hexanes) afforded **4k** (278 mg, 72% (60% **4k**), >99:1 dr, >99:1 rr +2% *E* alkene) as white solid.

Data for (4*R**,5*S**)-5-bromo-4-chloro-5-phenylpentyl thiophene-2-carboxylate (**4k**): HM-12-025

^{1}H NMR: (400 MHz, $CDCl_3$)

δ 7.81–7.80 (m, 1H), 7.57 (d, *J* = 5.0, 1H), 7.40–7.33 (m, 5H), 7.12–7.10 (m, 1H), 5.04 (d, *J* = 9.2, 1H), 4.47–4.34 (m, 3H), 2.52–2.44 (m, 1H), 2.21–2.06 (m, 2H), 2.01–1.93 (m, 1H).

5.23 (d, *J* = 9.2, 0.0055H, the *dia*-**4k**).

5.09 (d, *J* = 10.5, 0.16H, the dibromide **3k**).

^{13}C NMR: (100 MHz, $CDCl_3$)

δ 162.3, 139.5, 133.9, 133.7, 132.7, 129.1, 128.8, 128.0, 127.8, 66.0, 64.3, 58.0, 32.1, 26.5.

HRMS (FAB): $[M–Cl]^+$ calcd for $C_{16}H_{16}{}^{79}BrO_2S$, $C_{16}H_{16}{}^{81}BrO_2S$: 351.0055 (73.7%), 353.0034 (100.0%); found: 351.0054 (97.2%), 353.0041 (100.0%).

$[M+H]^+$ calcd for $C_{16}H_{17}{}^{79}Br^{35}ClO_2S$, $[C_{16}H_{17}{}^{81}Br^{35}ClO_2S + C_{16}H_{17}{}^{79}Br^{37}ClO_2S]$: 386.9821 (73.7%), 388.9799 (100.0%); found: 386.9818 (74.9%), 388.9794 (100.0%).

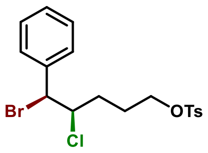

Flash column chromatography (SiO_2, ϕ = 3.0 cm, *l* = 13 cm, CH_2Cl_2/hexanes = 1/1, R_f = 0.25 [254 nm/CAM(blue)], 374 mg, 87% (64% **4l**), 98:2 dr, 97:3 rr, +3% *E* alkene) and recrystallization (CH_2Cl_2/hexanes) afforded a **4l** (293 mg, 68% (54% **4l**), 98:2 dr, >99:1 rr) as white solid.

Data for (4*R**,5*S**)-5-bromo-4-chloro-5-phenylpentyl 4-methylbenzenesulfonate (**4l**): JYH-02-057

^{1}H NMR: (400 MHz, $CDCl_3$)

δ 7.80 (d, *J* = 8.2, 2H), 7.40–7.33 (m, 7H), 4.94 (d, *J* = 8.9, 1H), 4.28 (td, *J* = 9.2, 2.7, 1H), 4.12–4.09 (m, 2H), 2.46 (s, 3H), 2.39–2.31 (m, 1H), 2.06–1.82 (m, 3H).

5.10 (d, *J* = 5.2, 0.016H, the *dia*-**4l**).

4.99 (d, *J* = 10.4, 0.10H, the dibromide **3l**).

^{13}C NMR: (100 MHz, $CDCl_3$)

δ 145.1, 139.3, 133.2, 130.1, 129.1, 128.8, 128.1, 127.8, 69.6, 65.8, 57.7, 31.7, 26.8, 21.9.

HRMS (ESI): $[M+Na]^+$ calcd for $C_{18}H_{20}{}^{79}Br^{35}ClNaO_3S$, $[C_{18}H_{20}{}^{81}Br^{35}ClNaO_3S + C_{18}H_{20}{}^{79}Br^{37}ClNaO_3S]$, $C_{18}H_{20}{}^{79}Br^{35}ClNaO_3S$: 452.9903 (77.3%), 454.9880 (100.0%), 456.9853 (24.1%); found: 452.9899 (72.7%), 454.9878 (100.0%), 456.9851 (27.4%).

2.7. Reaction Condition Optimization for *syn*-Bromochlorination

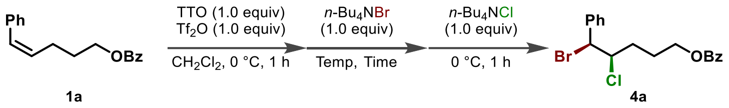

Entry	Temp	Time	Yield[b]	dr[c]	rr[c]
1	0 °C	1 h	49%	>99:1	99:1
2	–30 °C	1 h	61%	>99:1	99:1
3	–50 °C	1 h	58%	>99:1	>99:1
4	–78 °C	1 h	62%	>99:1	>99:1
5	–78 °C	3 h	60%	>99:1	>99:1

[a] Standard conditions: 1.0 mmol scale at 0.25 M concentration. [b] Calibrated yields based on ^{1}H NMR integration after column chromatography. [c] Determined by ^{1}H NMR analysis of the purified materials. (rr: regioisomeric ratio).

2.8. *anti*-Dibromination of *E*-**1a**

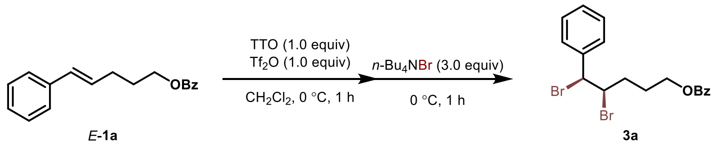

To a stirred solution of alkene (*E*-**1a**, 232 mg, 1.00 mmol, 1.0 equiv) in CH_2Cl_2 (4 mL) was added Tf_2O (170 µL, 1.0 mmol, 1.0 equiv) at 0 °C. After 1 hour, a solution of *n*-Bu_4NBr (967 mg, 3.00 mmol, 3.0 equiv) in CH_2Cl_2 (8 mL) was added. After 1 hour, the reaction mixture was concentrated in vacuo, and the residue was diluted with Et_2O (5 mL), filtered through a pad of SiO_2 (ϕ = 1.5 cm, l = 3.0 cm, Et_2O, 150 mL), and concentrated in vacuo. The crude material was purified by flash column chromatography (SiO_2, ϕ = 3.5 cm, l = 11 cm, CH_2Cl_2/hexanes = 1/2, R_f = 0.25, [254 nm/CAM(blue)]) to afford **3a** (260 mg, 61%, >99:1 dr) as white solid.

2.9. Stereoconvergent Dibromination of *Z*/*E* Mixture of **1a**

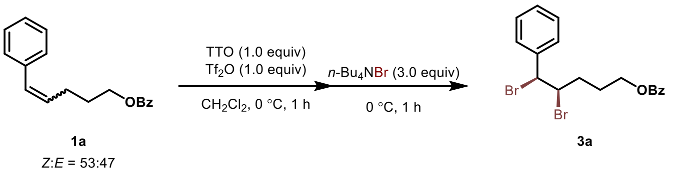

To a stirred solution of alkene (*Z*/*E* = 53:47, 1.0 mmol, 1.0 equiv) and thianthrene-*S*-oxide (232 mg, 1.00 mmol, 1.0 equiv) in CH_2Cl_2 (4 mL) was added Tf_2O (170 µL, 1.0 mmol, 1.0 equiv) at 0 °C. After 1 hour, a solution of *n*-Bu_4NBr (967 mg, 3.00 mmol, 3.0 equiv) in CH_2Cl_2 (8 mL) was added. After 1 hour, the reaction mixture was concentrated in vacuo and the residue was diluted with Et_2O (5 mL), filtered through a pad of SiO_2 (ϕ = 1.5 cm, l = 3.0 cm, Et_2O, 150 mL), and concentrated in vacuo. The crude material was purified by flash column chromatography (SiO_2, ϕ = 3.5 cm, l = 10 cm, CH_2Cl_2/hexanes = 1/2 (R_f = 0.25, [254 nm/CAM(blue)]) to afford **3a** (348 mg, 82% (62% **3a**), 96:4 dr, + 20% *E* alkene) as white solid.

3. Characterization Figures

3.1. X-Ray Crystallographic Data

Crystal (**3f**) mounted on a diffractometer was analyzed at 296 K. X-Ray Crystallographic Data Reflection data were collected using a Rigaku Oxford Diffraction XtaLAB Synergy-S diffractometer with $Cu_{K\alpha}$ radiation (λ = 1.54184 Å). The cell parameters were determined and refined using the CrysAlisPro program.[8] The compound structures were solved by direct methods and refined by full matrix least-squares using the Olex2[9] with anisotropic thermal parameters for all non-hydrogen atoms. The relevant data are summarized in Table S1. CCDC 2422098 contains the supplementary crystallographic data for this study. These data can be obtained free of charge from The Cambridge Crystallographic Data Centre via www.ccdc.cam.ac.uk/data_request/cif.

Table S1. Crystallographic data and parameters for compound **3f**

	**3f**
CCDC #	2422098
Empirical formula	$C_{19}H_{17}Br_2NO_2$
Formula weight	451.16
Temperature/K	296
Crystal system	Monoclinic
Space group	P 1 21/c 1
a/Å	16.3245(5)
b/Å	7.1276(2)
c/Å	15.7563(4)
α/°	90
β/°	99.259(3)
γ/°	90
Volume/Å^3	1809.43(9)
Z	4
ρ_{calc}(g/cm^3)	1.656
μ/mm^{-1}	5.770
F(000)	892.8
Crystal size/mm^3	0.16 × 0.15 × 0.10
Radiation	$Cu_{K\alpha}$ (λ = 1.54184 Å)
2Θ range for data collection/°	10.98 to 155.26
Index ranges	$-20 \le h \le 20$, $-8 \le k \le 8$, $-18 \le l \le 19$
Reflections collected	20490
Independent reflections	3686 [R_{int} = 0.0523, R_{sigma} = 0.0325]
Data/restraints/parameters	3686/0/217
Goodness-of-fit on F2	1.0431
Final R indexes [I>=2σ (I)]	R_1 = 0.0733, wR_2 = 0.1910
Final R indexes [all data]	R_1 = 0.0837, wR_2 = 0.1986
Largest diff. peak/hole / e Å-3	1.7359/–1.0571

$R_1 = \sum||Fo| - |Fc||/\sum|Fo|$. ^{b} $wR_2 = \{[\sum w(Fo^2 - Fc^2)^2]/[\sum w(Fo^2)^2]\}^{1/2}$.

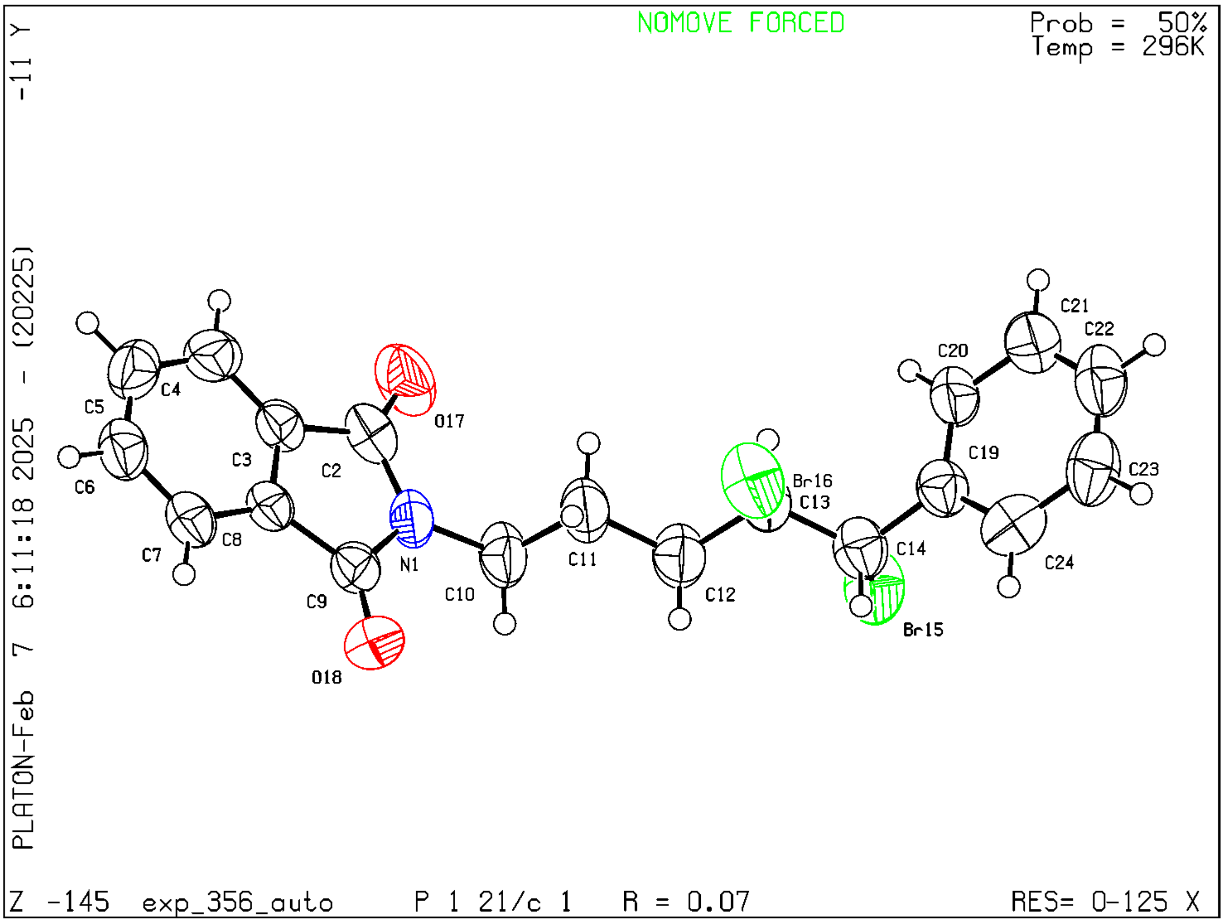

Figure. S1. Thermal ellipsoid plot of **3f** at the 50% probability level, CCDC 2422098.

single_pulse
Filename = OSL_20250111_JYH-01-035 descriptive alkene_Proton-1-2.jdf
Author = OSL
Sample_Id = 20250111_JYH-01-035 descriptive alkene
Creation_Time = 12-JAN-2025 00:46:42
Revision_Time = 15-JUL-2025 15:43:11

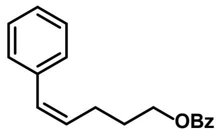

1a

^{1}H NMR (400 MHz, $CDCl_3$)

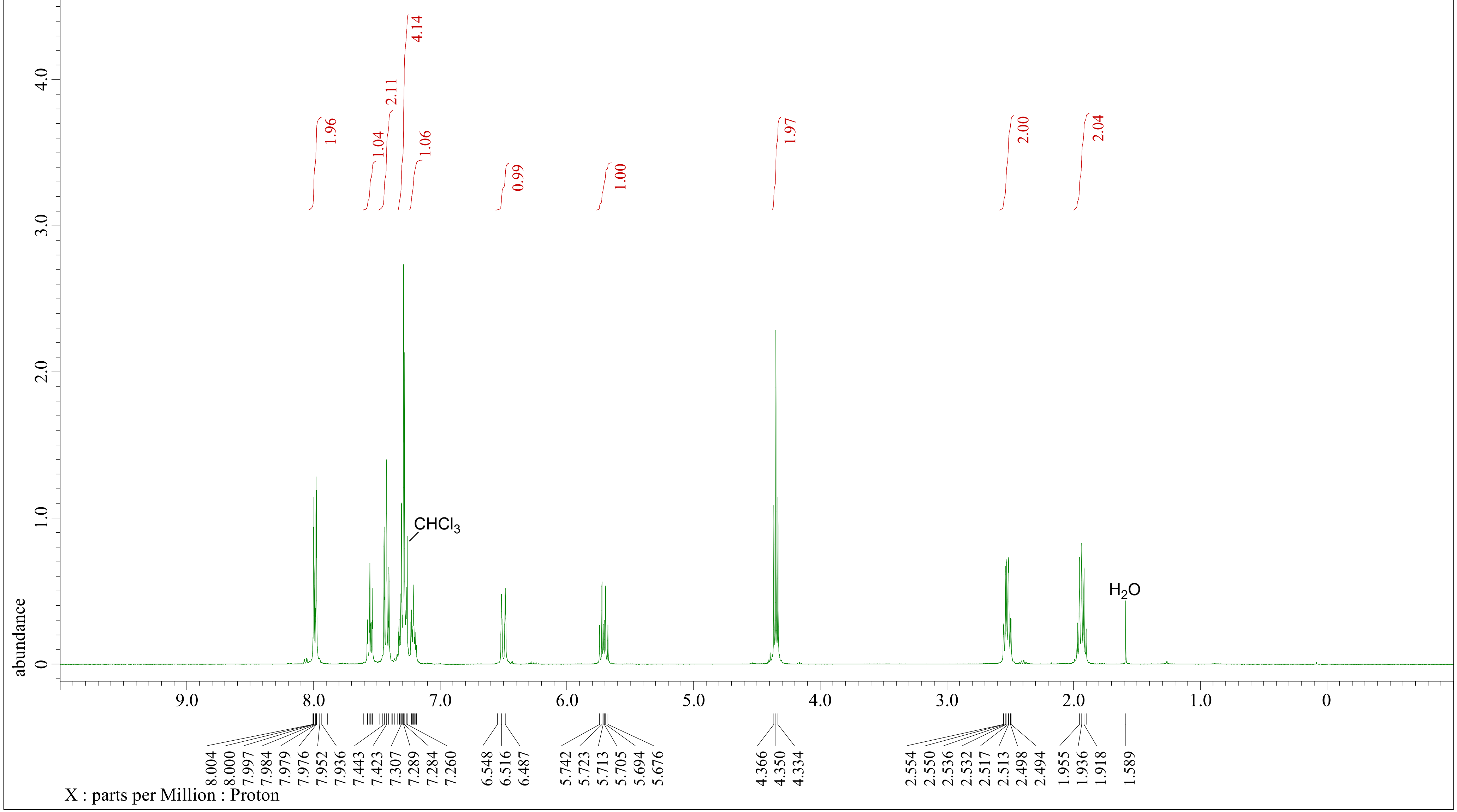

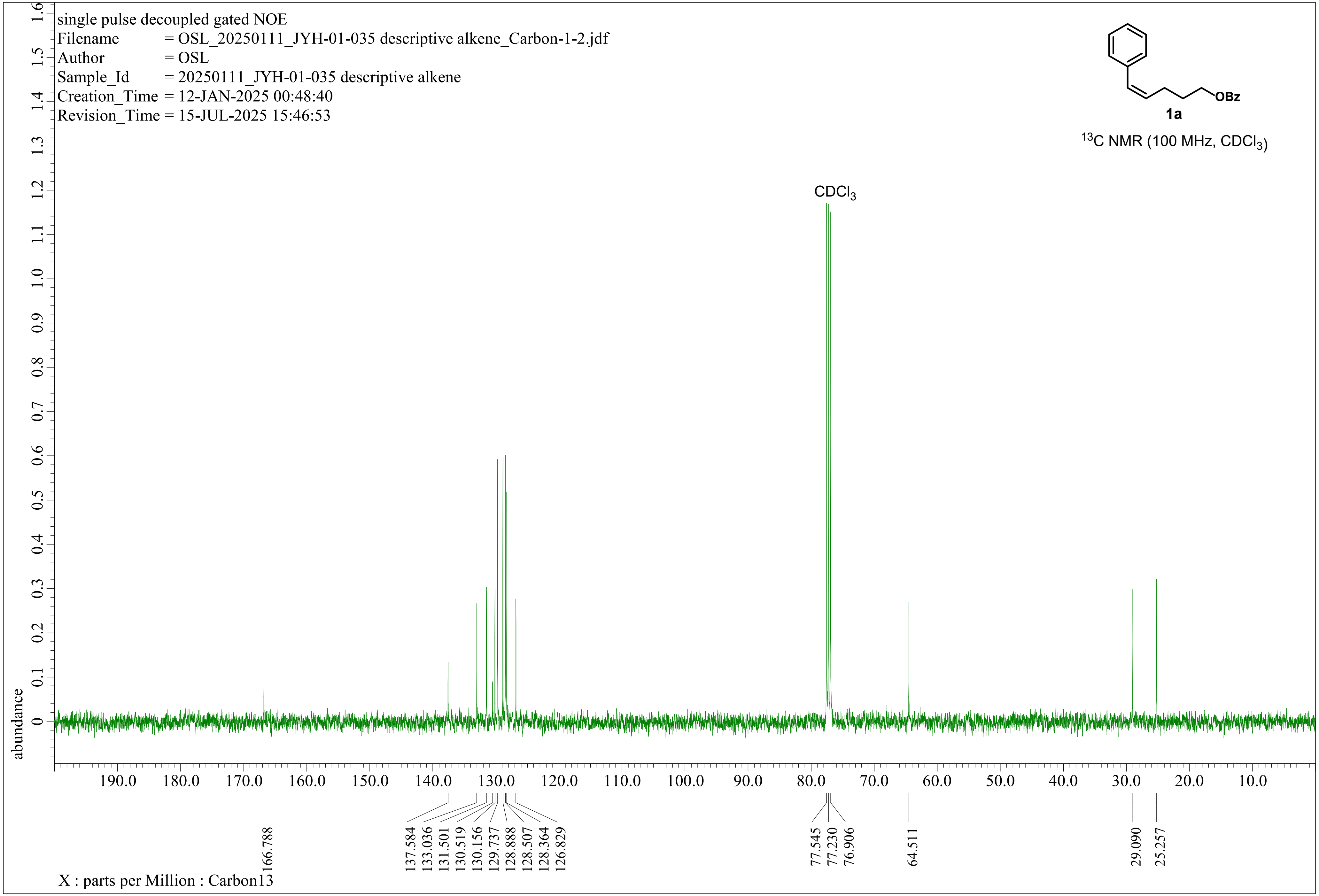
single pulse decoupled gated NOE
Filename = OSL_20250111_JYH-01-035 descriptive alkene_Carbon-1-2.jdf
Author = OSL
Sample_Id = 20250111_JYH-01-035 descriptive alkene
Creation_Time = 12-JAN-2025 00:48:40
Revision_Time = 15-JUL-2025 15:46:53
OBz
1a
13C NMR (100 MHz, CDCl3)
CDCl3
abundance
X : parts per Million : Carbon13
166.788
137.584
133.036
131.501
130.519
130.156
129.737
128.888
128.507
128.364
126.829
77.545
77.230
76.906
64.511
29.090
25.257

single_pulse
Filename = OSL_20250111_JYH-01-057 descriptive alkene_Proton-1-2.jdf
Author = OSL
Sample_Id = 20250111_JYH-01-057 descriptive alkene
Creation_Time = 12-JAN-2025 01:07:00
Revision_Time = 15-JUL-2025 15:38:11

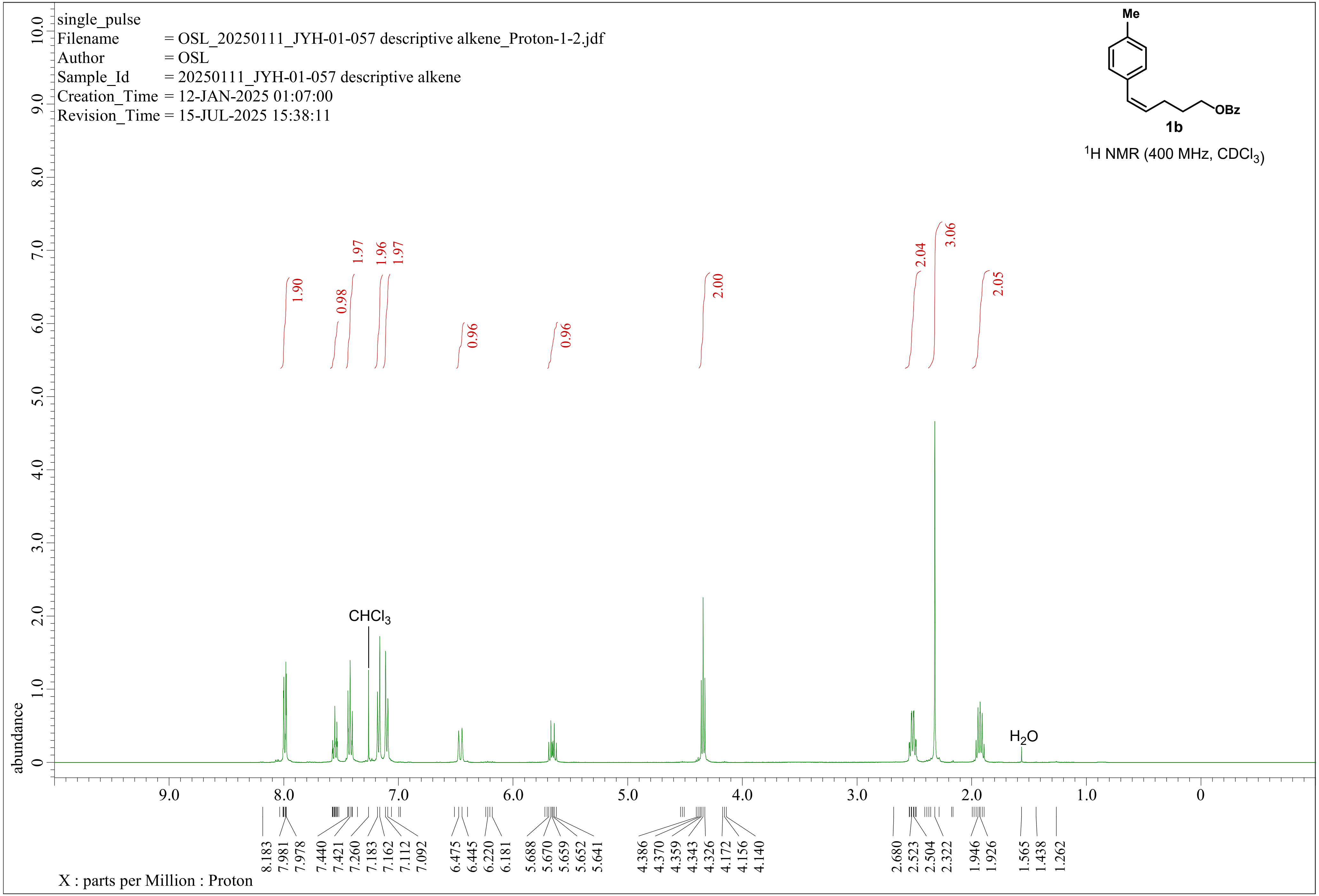

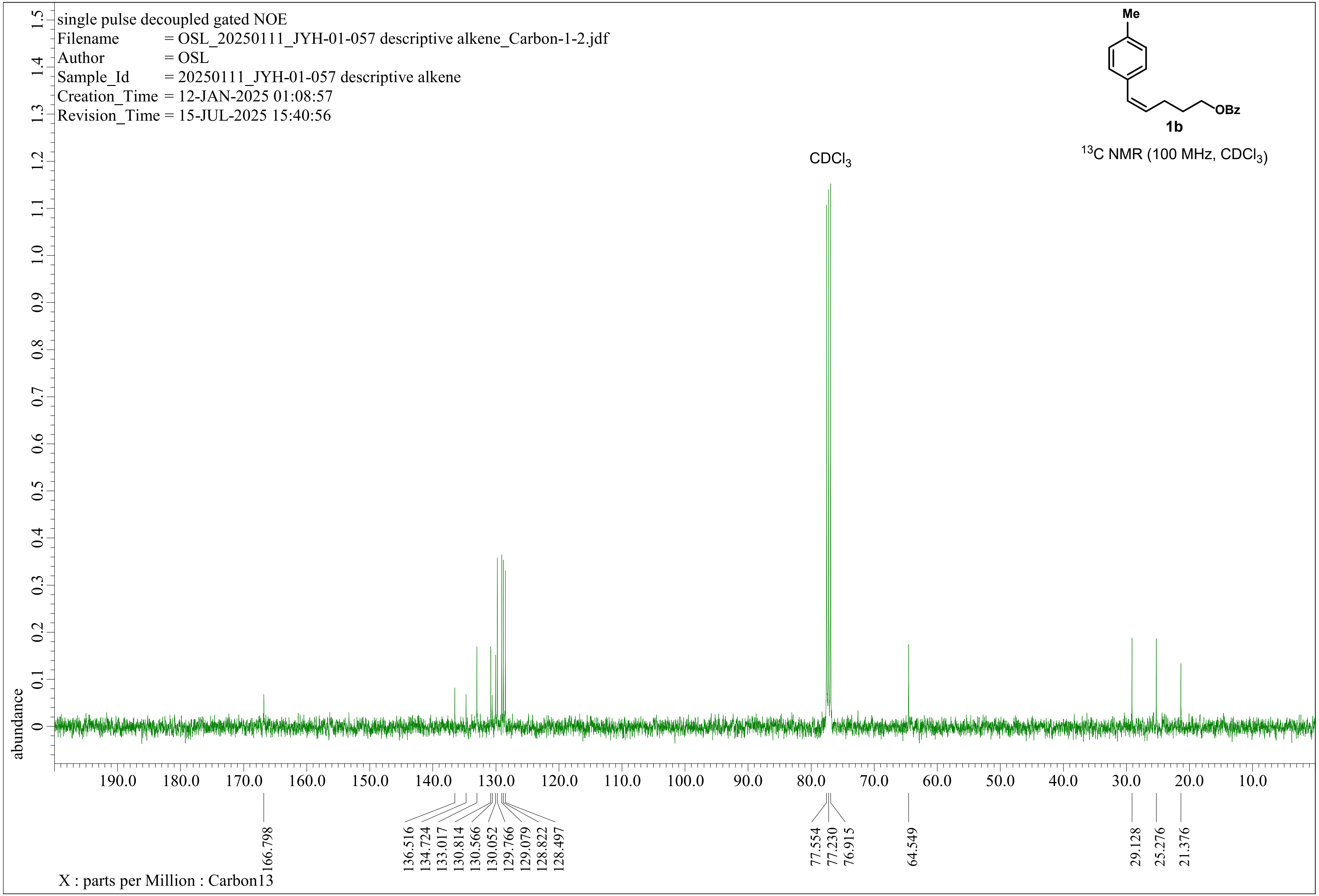
single pulse decoupled gated NOE
Filename = OSL_20250111_JYH-01-057 descriptive alkene_Carbon-1-2.jdf
Author = OSL
Sample_Id = 20250111_JYH-01-057 descriptive alkene
Creation_Time = 12-JAN-2025 01:08:57
Revision_Time = 15-JUL-2025 15:40:56
Me
OBz
1b
^{13}C NMR (100 MHz, $CDCl_3$)
$CDCl_3$
abundance
166.798
136.516
134.724
133.017
130.814
130.566
130.052
129.766
129.079
128.822
128.497
77.554
77.230
76.915
64.549
29.128
25.276
21.376
X : parts per Million : Carbon13

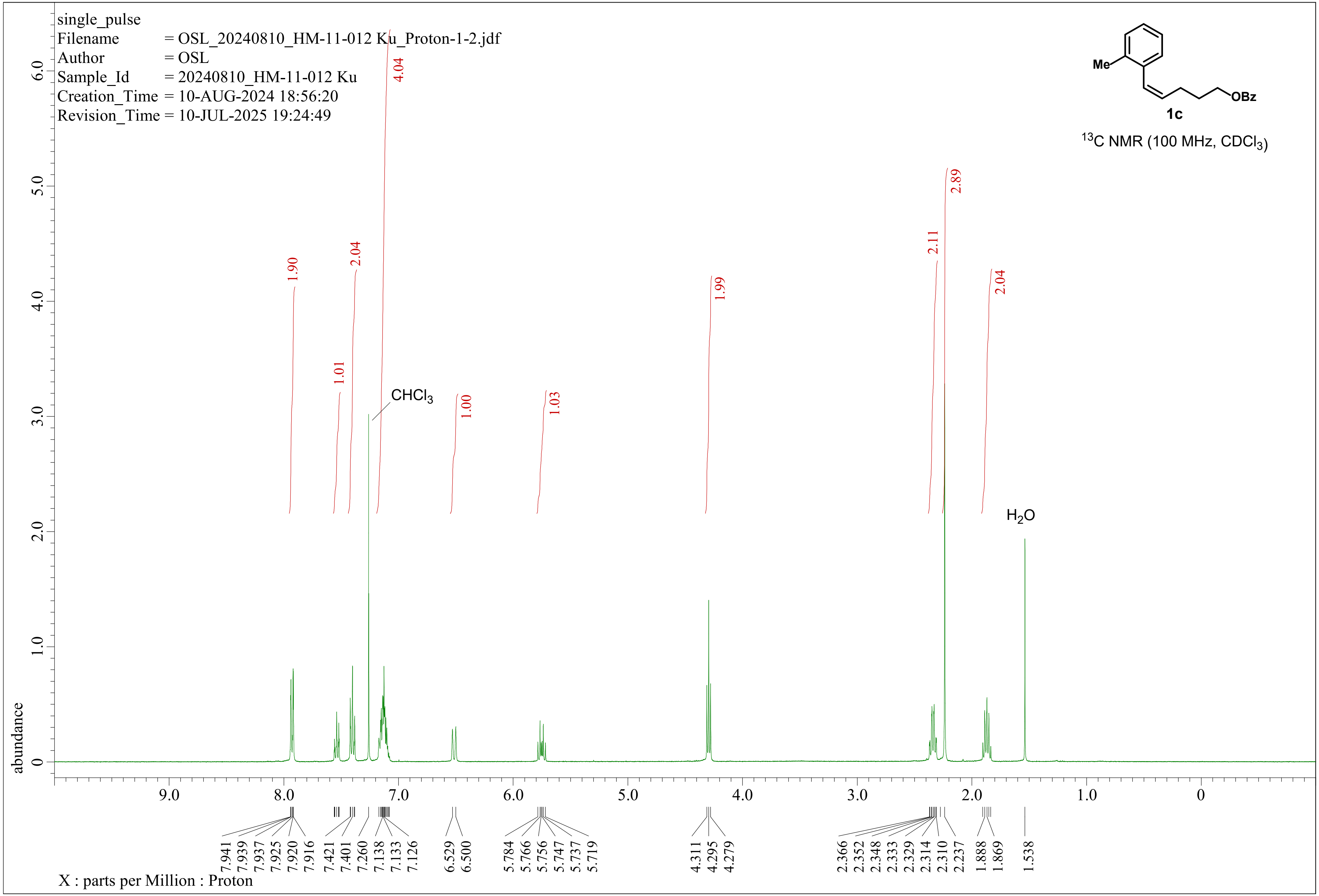
single_pulse
Filename = OSL_20240810_HM-11-012 Ku_Proton-1-2.jdf
Author = OSL
Sample_Id = 20240810_HM-11-012 Ku
Creation_Time = 10-AUG-2024 18:56:20
Revision_Time = 10-JUL-2025 19:24:49
Me
OBz
1c
13C NMR (100 MHz, CDCl3)
CHCl3
H2O
1.90
1.01
2.04
4.04
1.00
1.03
1.99
2.11
2.89
2.04
abundance
X : parts per Million : Proton
7.941 7.939 7.937 7.925 7.920 7.916 7.421 7.401 7.260 7.138 7.133 7.126 6.529 6.500 5.784 5.766 5.756 5.747 5.737 5.719 4.311 4.295 4.279 2.366 2.352 2.348 2.333 2.329 2.314 2.310 2.237 1.888 1.869 1.538

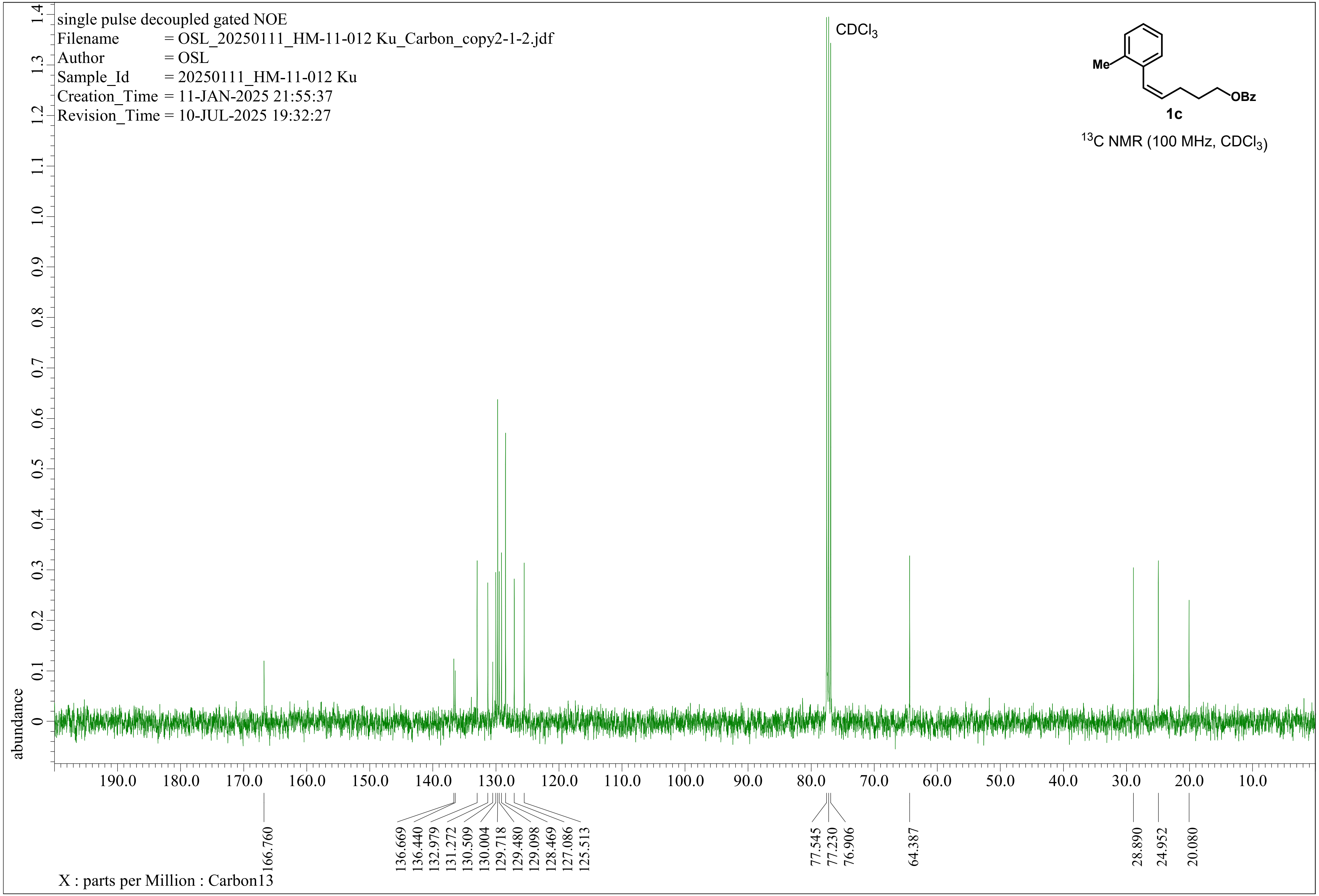
single pulse decoupled gated NOE
Filename = OSL_20250111_HM-11-012 Ku_Carbon_copy2-1-2.jdf
Author = OSL
Sample_Id = 20250111_HM-11-012 Ku
Creation_Time = 11-JAN-2025 21:55:37
Revision_Time = 10-JUL-2025 19:32:27
CDCl3
Me
OBz
1c
13C NMR (100 MHz, CDCl3)
abundance
X : parts per Million : Carbon13
166.760
136.669
136.440
132.979
131.272
130.509
130.004
129.718
129.480
129.098
128.469
127.086
125.513
77.545
77.230
76.906
64.387
28.890
24.952
20.080

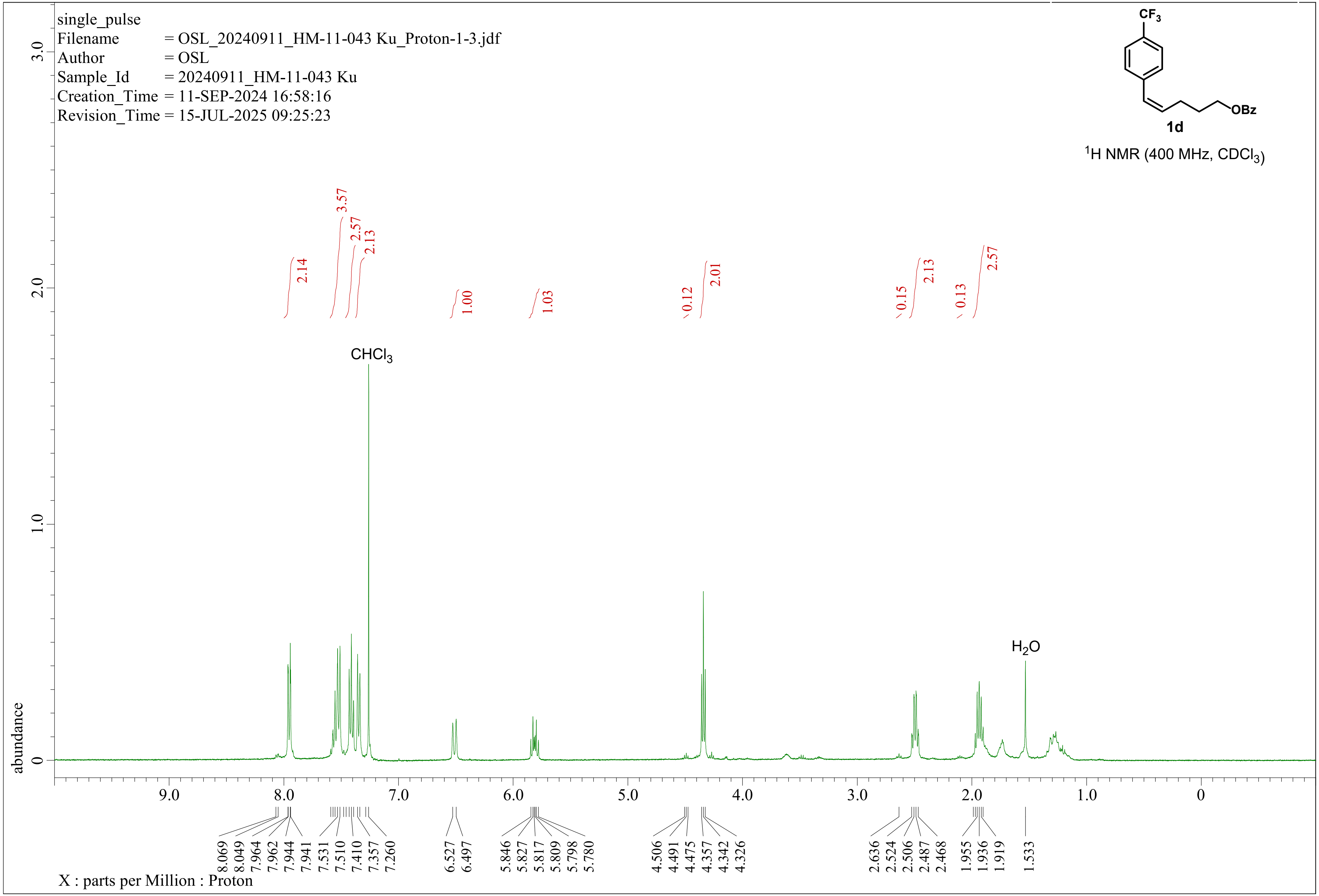

single_pulse
Filename = OSL_20240911_HM-11-043 Ku_Proton-1-3.jdf
Author = OSL
Sample_Id = 20240911_HM-11-043 Ku
Creation_Time = 11-SEP-2024 16:58:16
Revision_Time = 15-JUL-2025 09:25:23
CF3
OBz
1d
1H NMR (400 MHz, CDCl3)
2.14
3.57
2.57
2.13
1.00
1.03
0.12
2.01
0.15
2.13
0.13
2.57
CHCl3
H2O
abundance
3.0
2.0
1.0
0
9.0
8.0
7.0
6.0
5.0
4.0
3.0
2.0
1.0
0
8.069
8.049
7.964
7.962
7.944
7.941
7.531
7.510
7.410
7.357
7.260
6.527
6.497
5.846
5.827
5.817
5.809
5.798
5.780
4.506
4.491
4.475
4.357
4.342
4.326
2.636
2.524
2.506
2.487
2.468
1.955
1.936
1.919
1.533
X : parts per Million : Proton

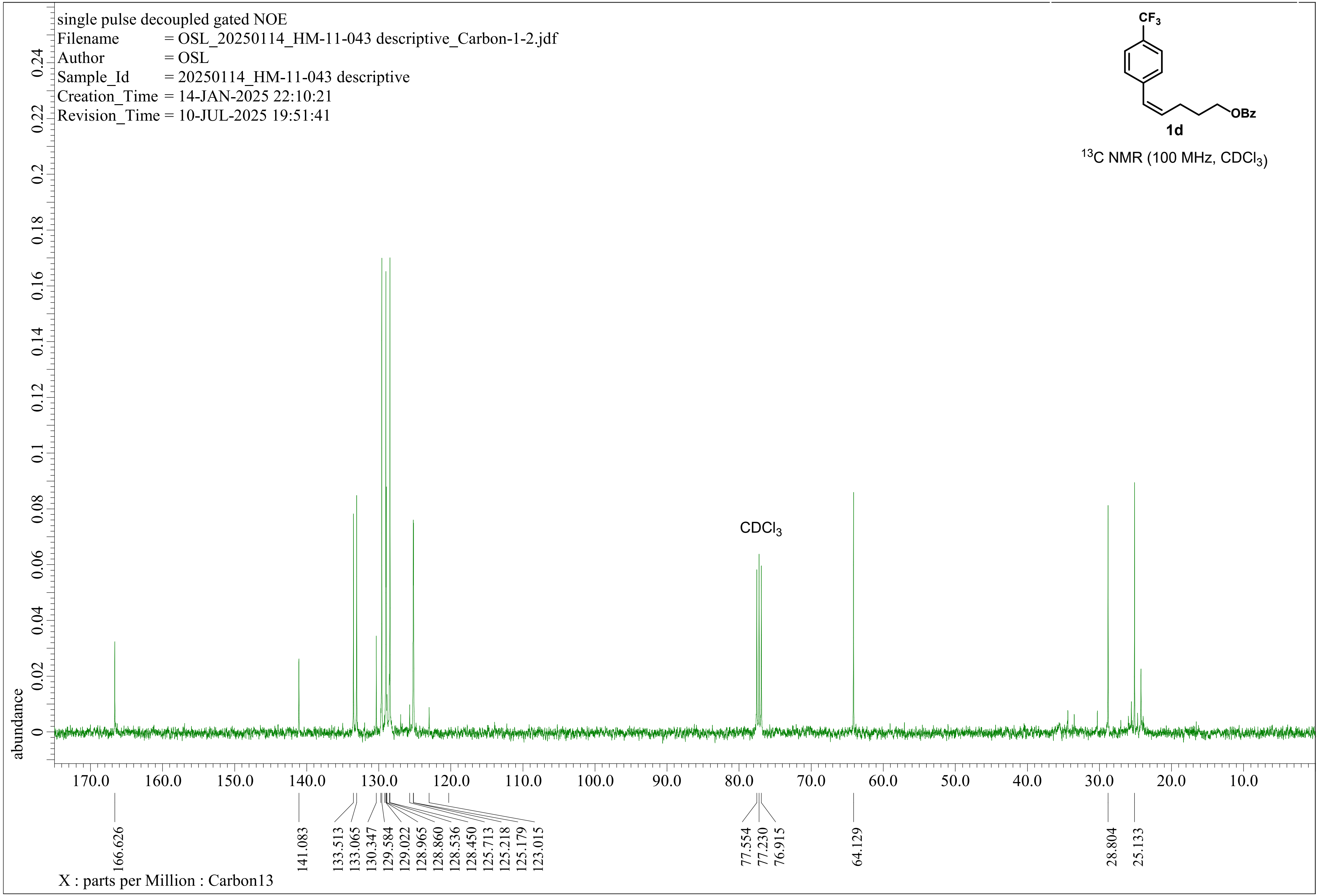

single pulse decoupled gated NOE
Filename = OSL_20250114_HM-11-043 descriptive_Carbon-1-2.jdf
Author = OSL
Sample_Id = 20250114_HM-11-043 descriptive
Creation_Time = 14-JAN-2025 22:10:21
Revision_Time = 10-JUL-2025 19:51:41
CF3
OBz
1d
13C NMR (100 MHz, CDCl3)
CDCl3
abundance
166.626
141.083
133.513
133.065
130.347
129.584
129.022
128.965
128.860
128.536
128.450
125.713
125.218
125.179
123.015
77.554
77.230
76.915
64.129
28.804
25.133
X : parts per Million : Carbon13

single_pulse
Filename = OSL20240911_HM-11-043 Ku_Fluorine-1-3.jdf
Author = OSL
Sample_Id = 20240911_HM-11-043 Ku
Creation_Time = 11-SEP-2024 16:55:55
Revision_Time = 10-JUL-2025 19:59:40

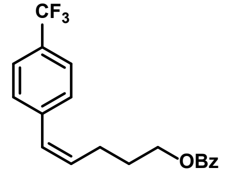

1d

^{19}F NMR (376 MHz, $CDCl_3$)

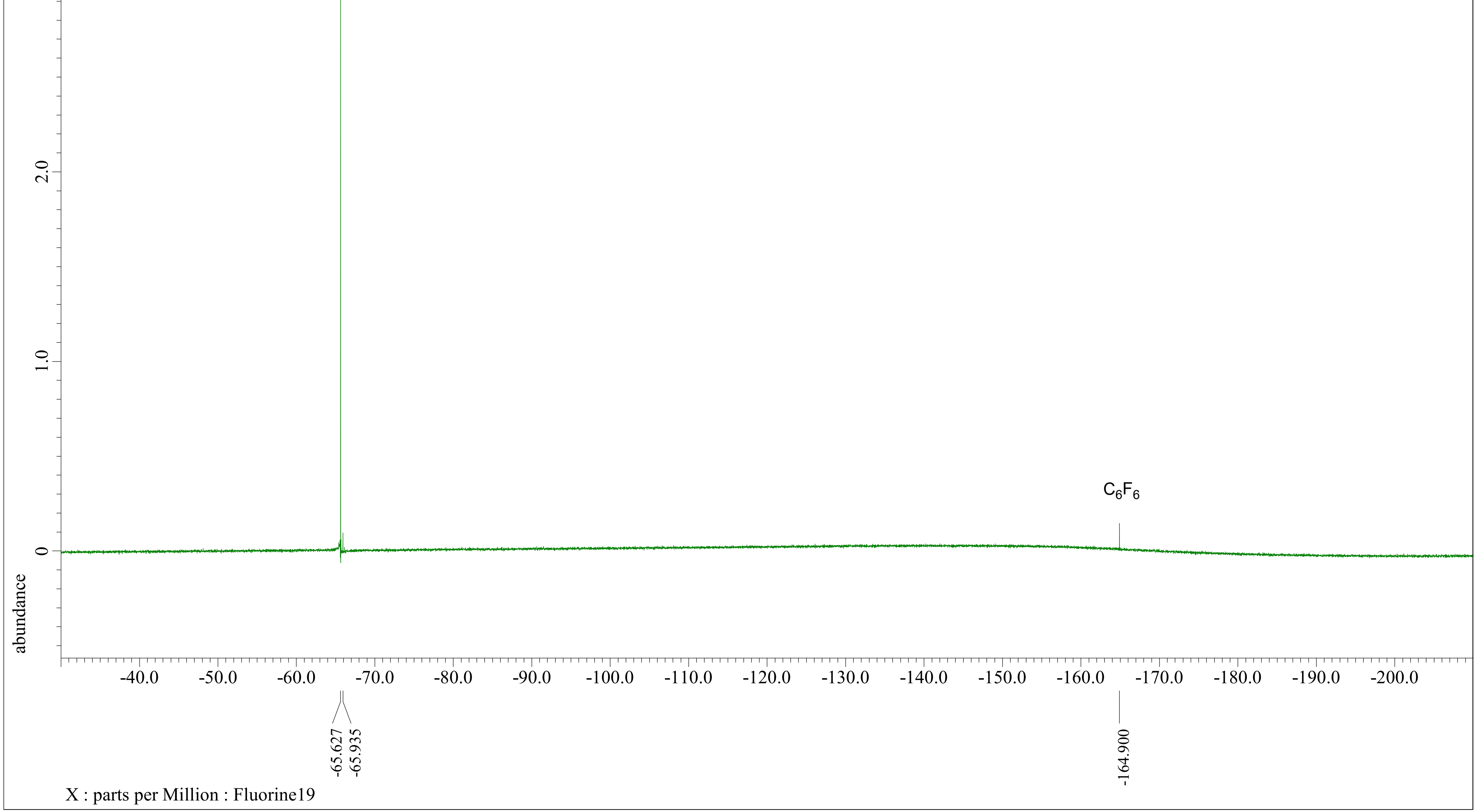

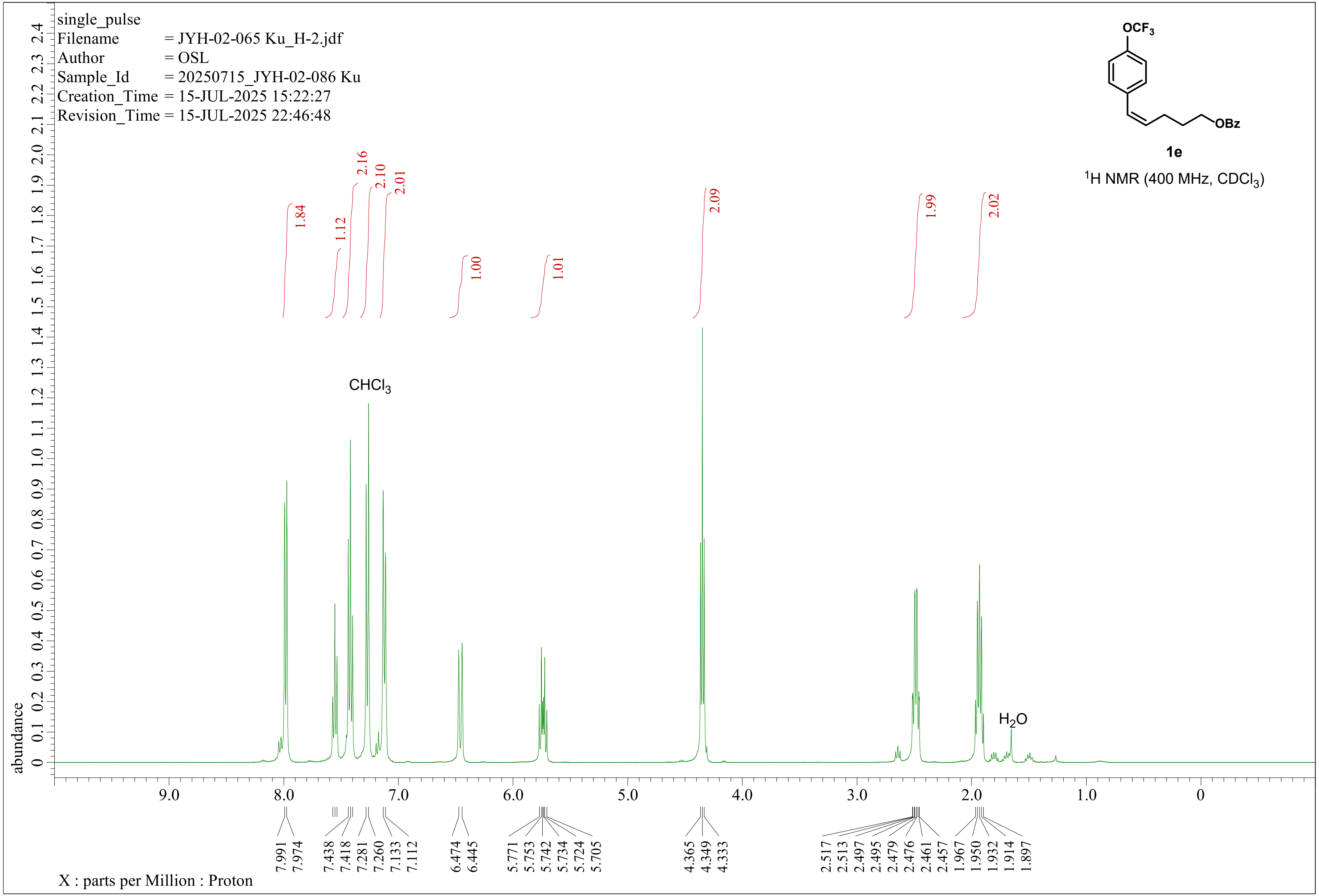

single_pulse
Filename = JYH-02-065 Ku_H-2.jdf
Author = OSL
Sample_Id = 20250715_JYH-02-086 Ku
Creation_Time = 15-JUL-2025 15:22:27
Revision_Time = 15-JUL-2025 22:46:48
OCF3
OBz
1e
1H NMR (400 MHz, CDCl3)
CHCl3
H2O
1.84
1.12
2.16
2.10
2.01
1.00
1.01
2.09
1.99
2.02
7.991
7.974
7.438
7.418
7.281
7.260
7.133
7.112
6.474
6.445
5.771
5.753
5.742
5.734
5.724
5.705
4.365
4.349
4.333
2.517
2.513
2.497
2.495
2.479
2.476
2.461
2.457
1.967
1.950
1.932
1.914
1.897
abundance
X : parts per Million : Proton

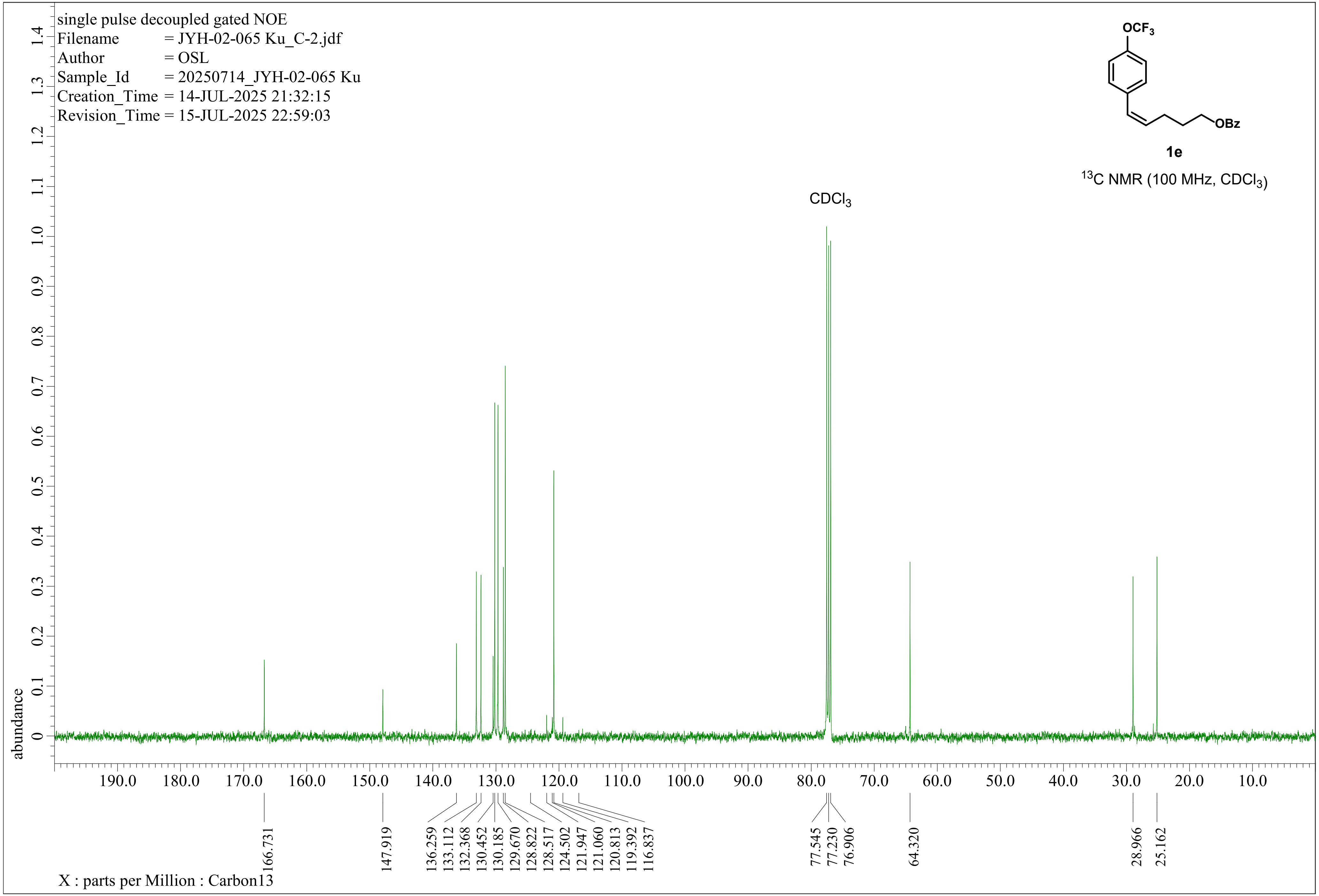

single pulse decoupled gated NOE
Filename = JYH-02-065 Ku_C-2.jdf
Author = OSL
Sample_Id = 20250714_JYH-02-065 Ku
Creation_Time = 14-JUL-2025 21:32:15
Revision_Time = 15-JUL-2025 22:59:03
OCF3
OBz
1e
13C NMR (100 MHz, CDCl3)
CDCl3
abundance
X : parts per Million : Carbon13
166.731
147.919
136.259
133.112
132.368
130.452
130.185
129.670
128.822
128.517
124.502
121.947
121.060
120.813
119.392
116.837
77.545
77.230
76.906
64.320
28.966
25.162

single_pulse
Filename = JYH-02-065 Ku_F-2.jdf
Author = OSL
Sample_Id = 20250710_JYH-02-065 Ku
Creation_Time = 10-JUL-2025 21:42:16
Revision_Time = 15-JUL-2025 22:48:31

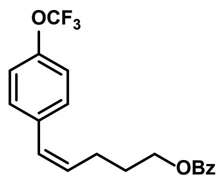

1e

^{19}F NMR (376 MHz, $CDCl_3$)

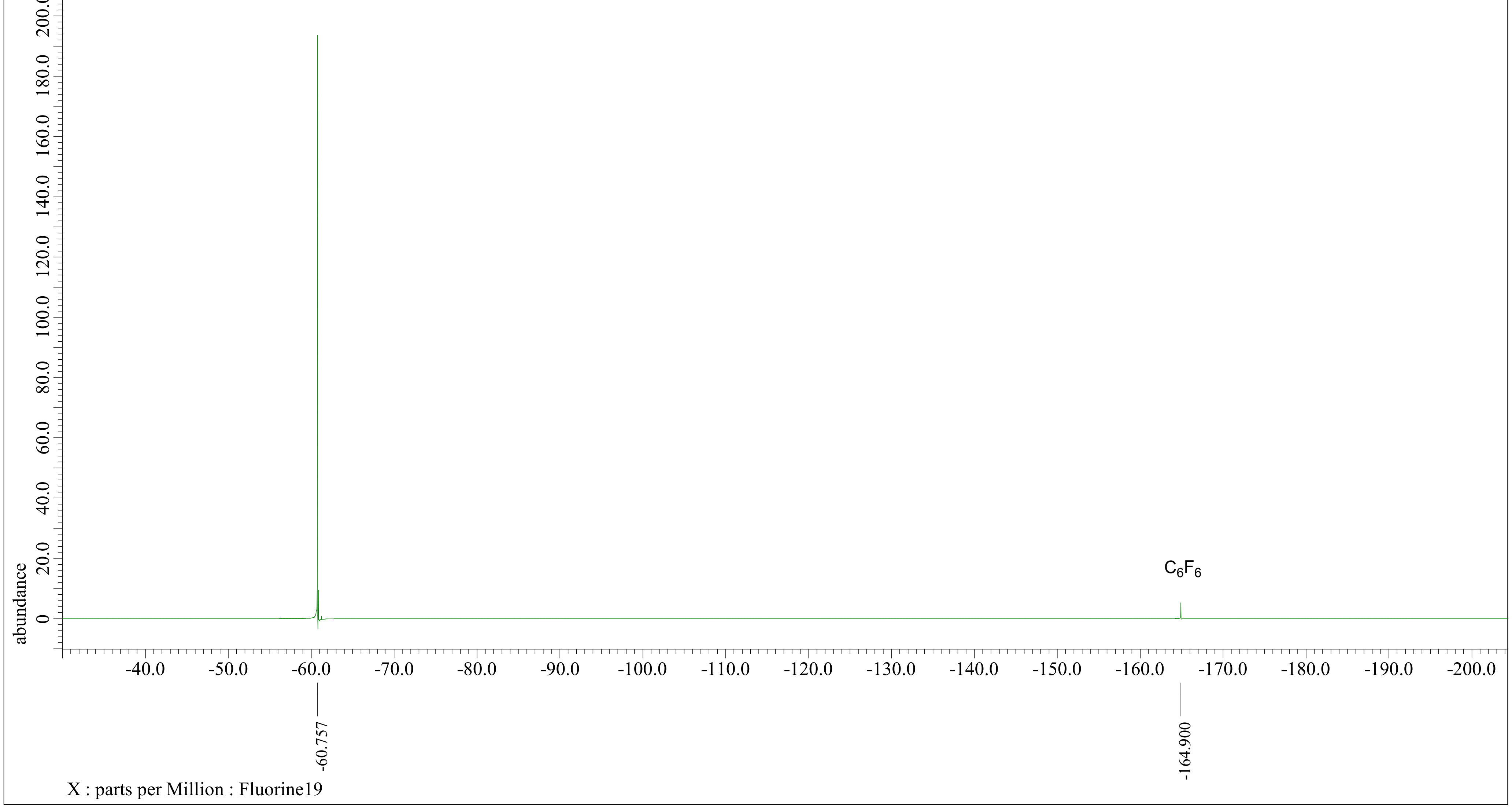

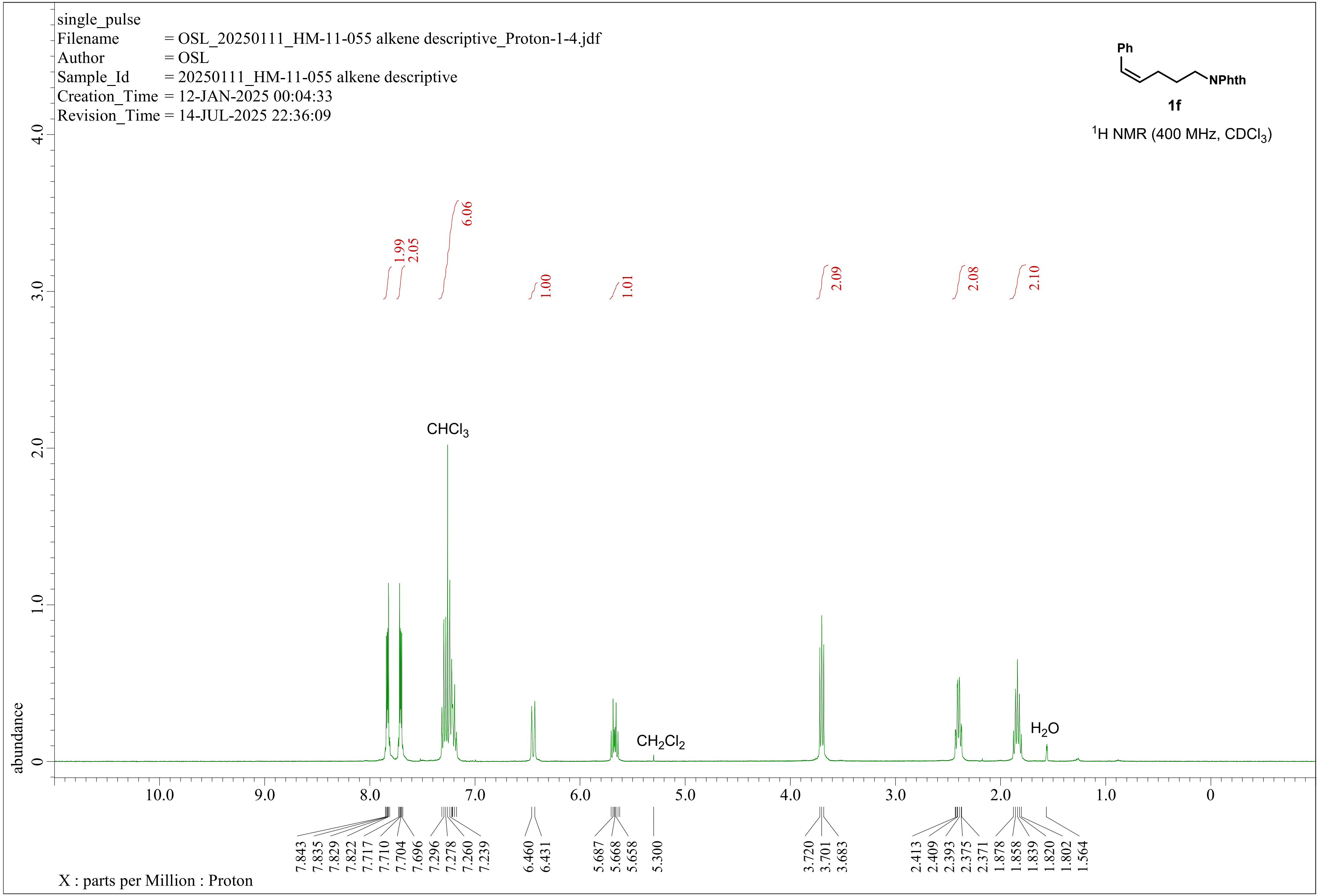
single_pulse
Filename = OSL_20250111_HM-11-055 alkene descriptive_Proton-1-4.jdf
Author = OSL
Sample_Id = 20250111_HM-11-055 alkene descriptive
Creation_Time = 12-JAN-2025 00:04:33
Revision_Time = 14-JUL-2025 22:36:09
Ph
NPhth
1f
1H NMR (400 MHz, CDCl3)
1.99
2.05
6.06
1.00
1.01
2.09
2.08
2.10
CHCl3
CH2Cl2
H2O
abundance
X : parts per Million : Proton
7.843 7.835 7.829 7.822 7.717 7.710 7.704 7.696 7.296 7.278 7.260 7.239
6.460 6.431
5.687 5.668 5.658
5.300
3.720 3.701 3.683
2.413 2.409 2.393 2.375 2.371 1.878 1.858 1.839 1.820 1.802 1.564

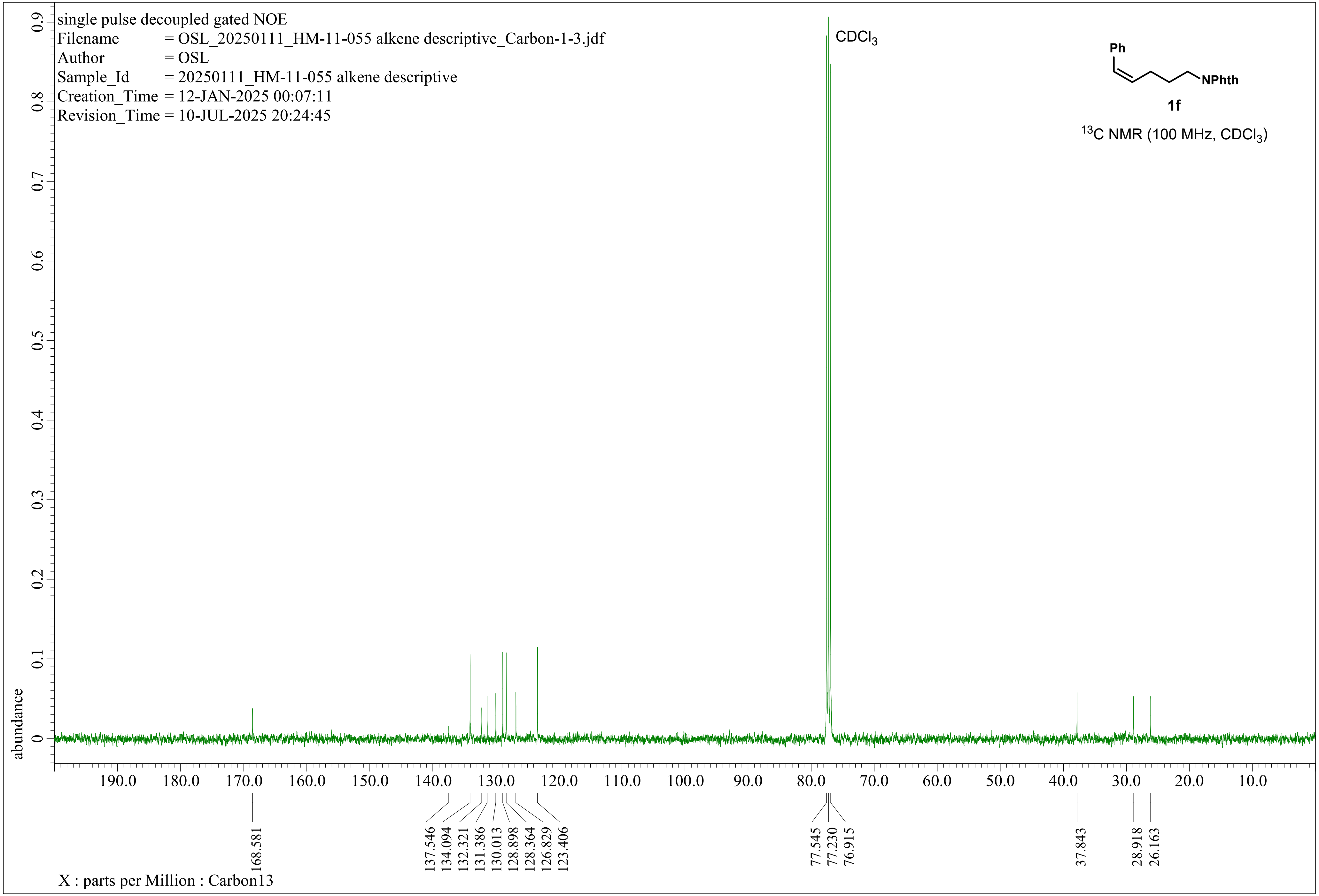
single pulse decoupled gated NOE
Filename = OSL_20250111_HM-11-055 alkene descriptive_Carbon-1-3.jdf
Author = OSL
Sample_Id = 20250111_HM-11-055 alkene descriptive
Creation_Time = 12-JAN-2025 00:07:11
Revision_Time = 10-JUL-2025 20:24:45
CDCl3
Ph
NPhth
1f
13C NMR (100 MHz, CDCl3)
abundance
X : parts per Million : Carbon13
168.581
137.546
134.094
132.321
131.386
130.013
128.898
128.364
126.829
123.406
77.545
77.230
76.915
37.843
28.918
26.163

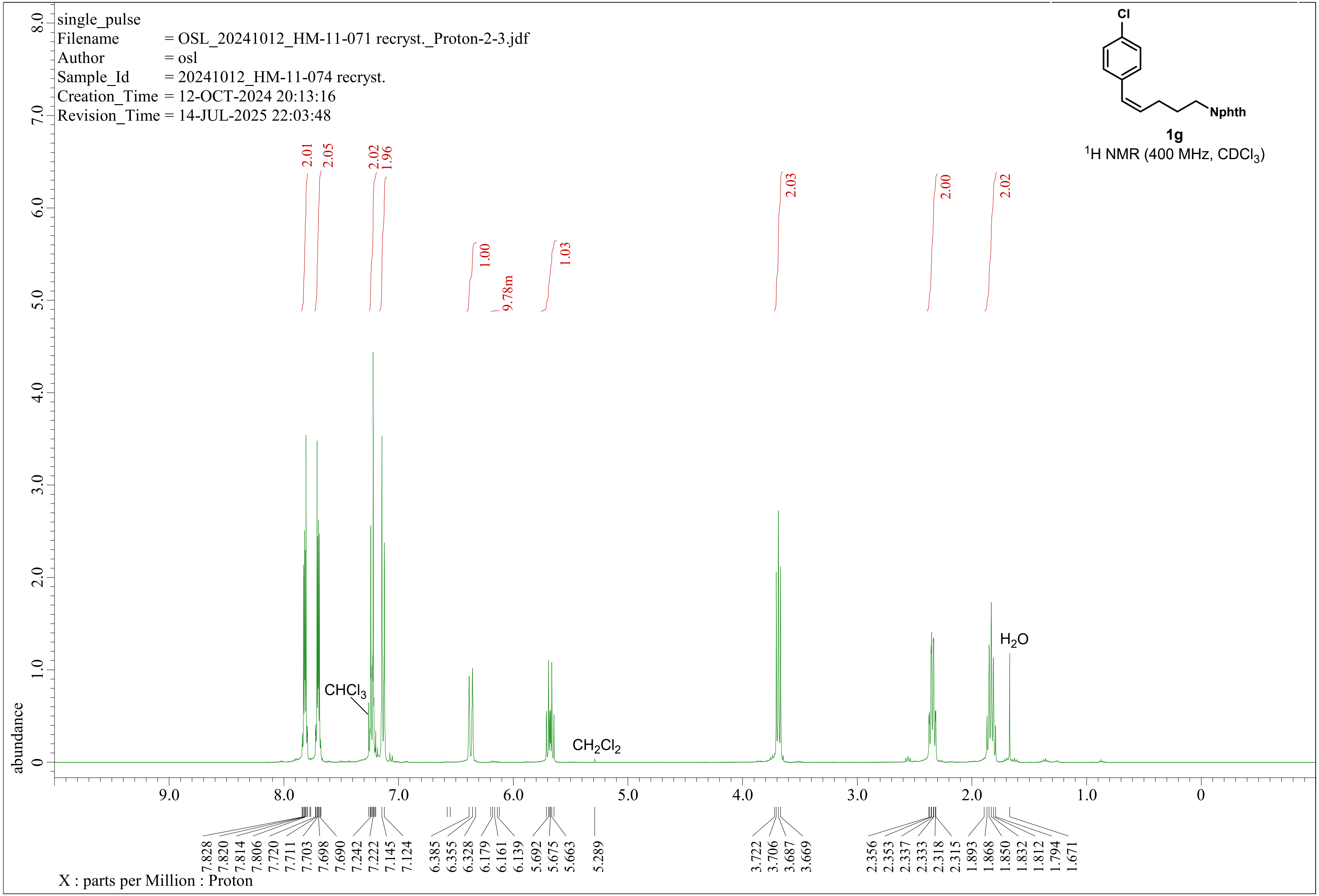
single_pulse
Filename = OSL_20241012_HM-11-071 recryst._Proton-2-3.jdf
Author = osl
Sample_Id = 20241012_HM-11-074 recryst.
Creation_Time = 12-OCT-2024 20:13:16
Revision_Time = 14-JUL-2025 22:03:48
Cl
Nphth
1g
1H NMR (400 MHz, CDCl3)
2.01
2.05
2.02
1.96
1.00
9.78m
1.03
2.03
2.00
2.02
CHCl3
CH2Cl2
H2O
abundance
X : parts per Million : Proton
7.828 7.820 7.814 7.806 7.720 7.711 7.703 7.698 7.690 7.242 7.222 7.145 7.124 6.385 6.355 6.328 6.179 6.161 6.139 5.692 5.675 5.663 5.289 3.722 3.706 3.687 3.669 2.356 2.353 2.337 2.333 2.318 2.315 1.893 1.868 1.850 1.832 1.812 1.794 1.671

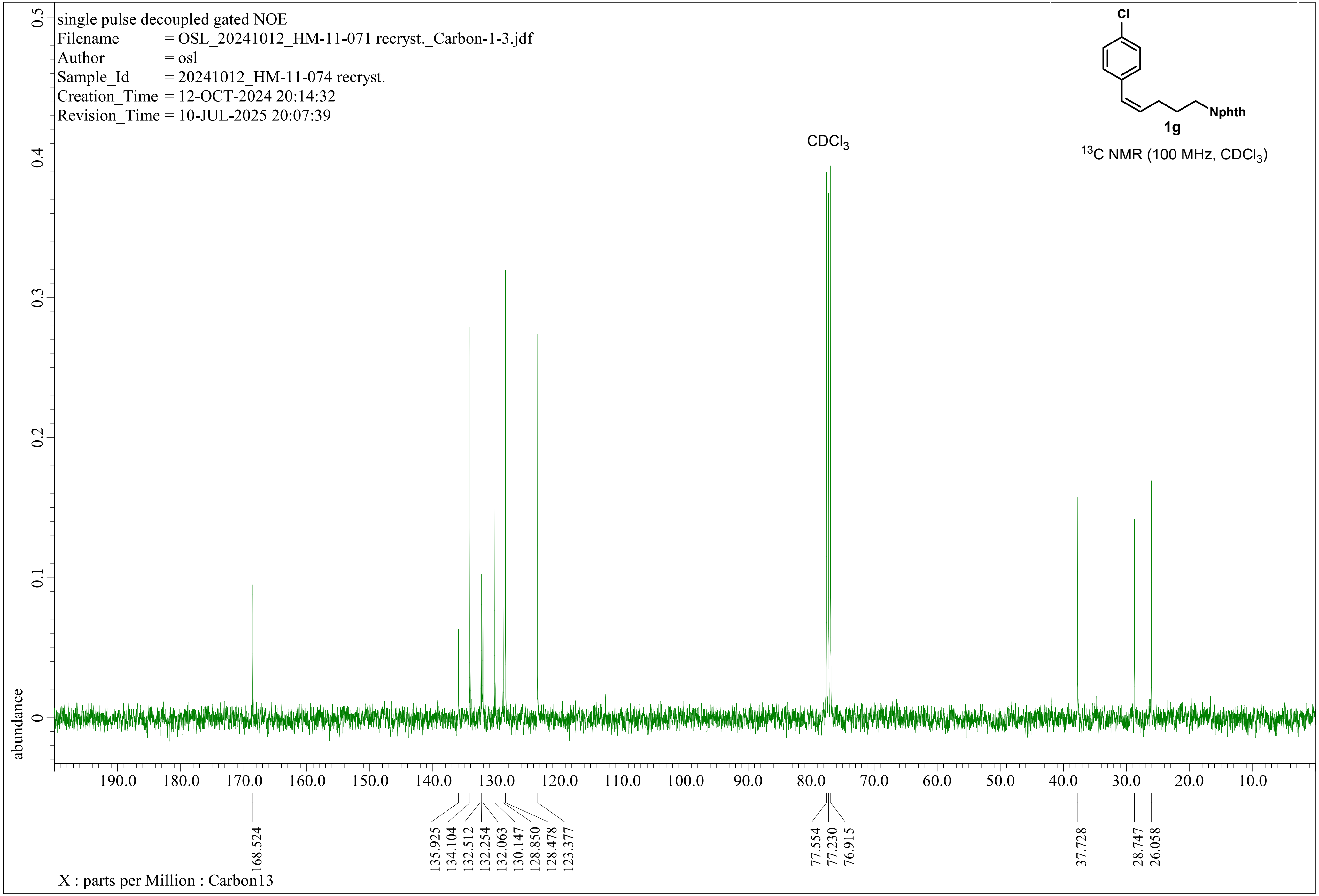
single pulse decoupled gated NOE
Filename = OSL_20241012_HM-11-071 recryst._Carbon-1-3.jdf
Author = osl
Sample_Id = 20241012_HM-11-074 recryst.
Creation_Time = 12-OCT-2024 20:14:32
Revision_Time = 10-JUL-2025 20:07:39
Cl
Nphth
1g
13C NMR (100 MHz, CDCl3)
CDCl3
abundance
0.5
0.4
0.3
0.2
0.1
0
190.0 180.0 170.0 160.0 150.0 140.0 130.0 120.0 110.0 100.0 90.0 80.0 70.0 60.0 50.0 40.0 30.0 20.0 10.0
168.524
135.925
134.104
132.512
132.254
132.063
130.147
128.850
128.478
123.377
77.554
77.230
76.915
37.728
28.747
26.058
X : parts per Million : Carbon13

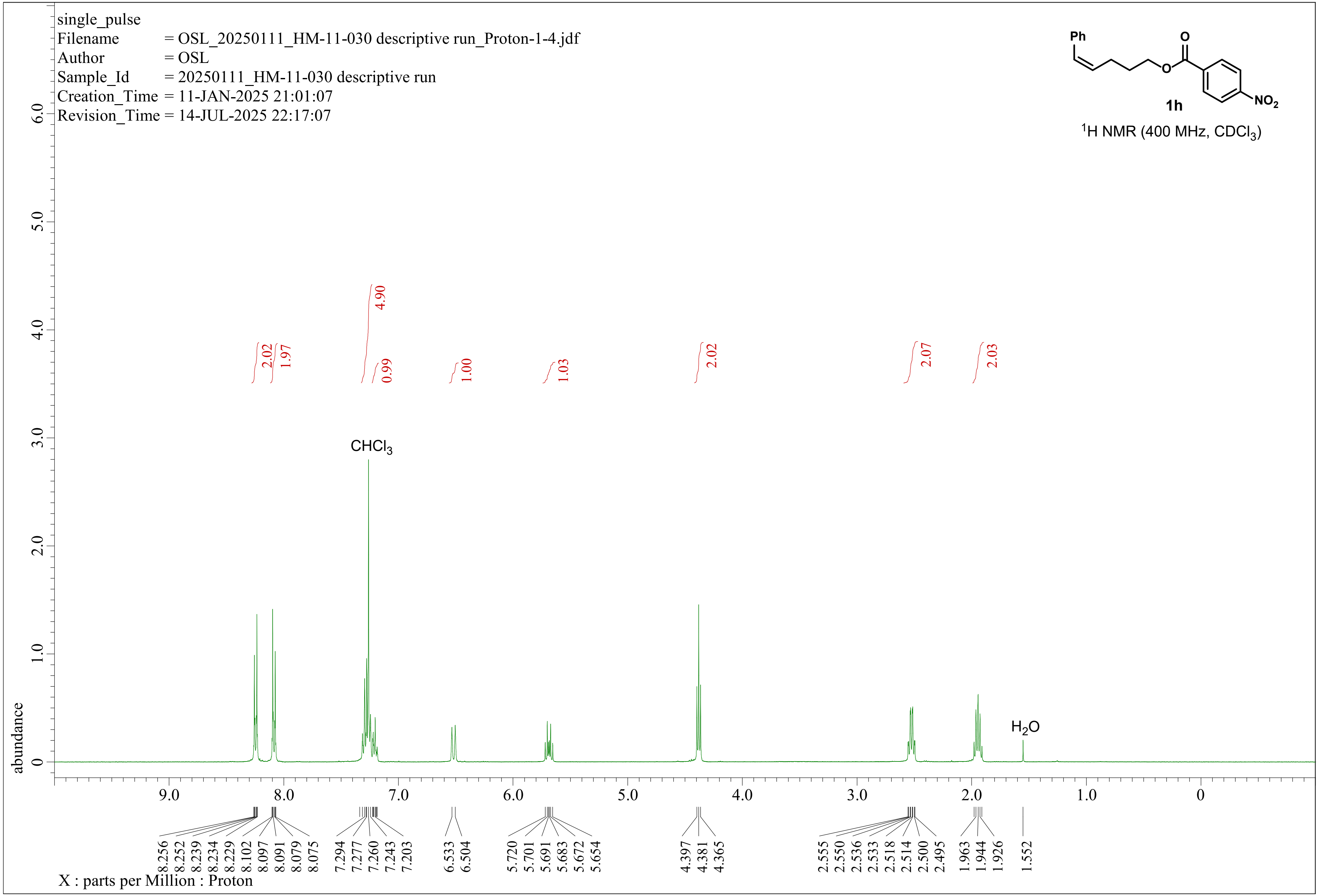

single_pulse
Filename = OSL_20250111_HM-11-030 descriptive run_Proton-1-4.jdf
Author = OSL
Sample_Id = 20250111_HM-11-030 descriptive run
Creation_Time = 11-JAN-2025 21:01:07
Revision_Time = 14-JUL-2025 22:17:07
Ph
O
O
NO2
1h
1H NMR (400 MHz, CDCl3)
CHCl3
H2O
2.02
1.97
4.90
0.99
1.00
1.03
2.02
2.07
2.03
8.256 8.252 8.239 8.234 8.229 8.102 8.097 8.091 8.079 8.075
7.294 7.277 7.260 7.243 7.203
6.533 6.504
5.720 5.701 5.691 5.683 5.672 5.654
4.397 4.381 4.365
2.555 2.550 2.536 2.533 2.518 2.514 2.500 2.495
1.963 1.944 1.926
1.552
abundance
X : parts per Million : Proton

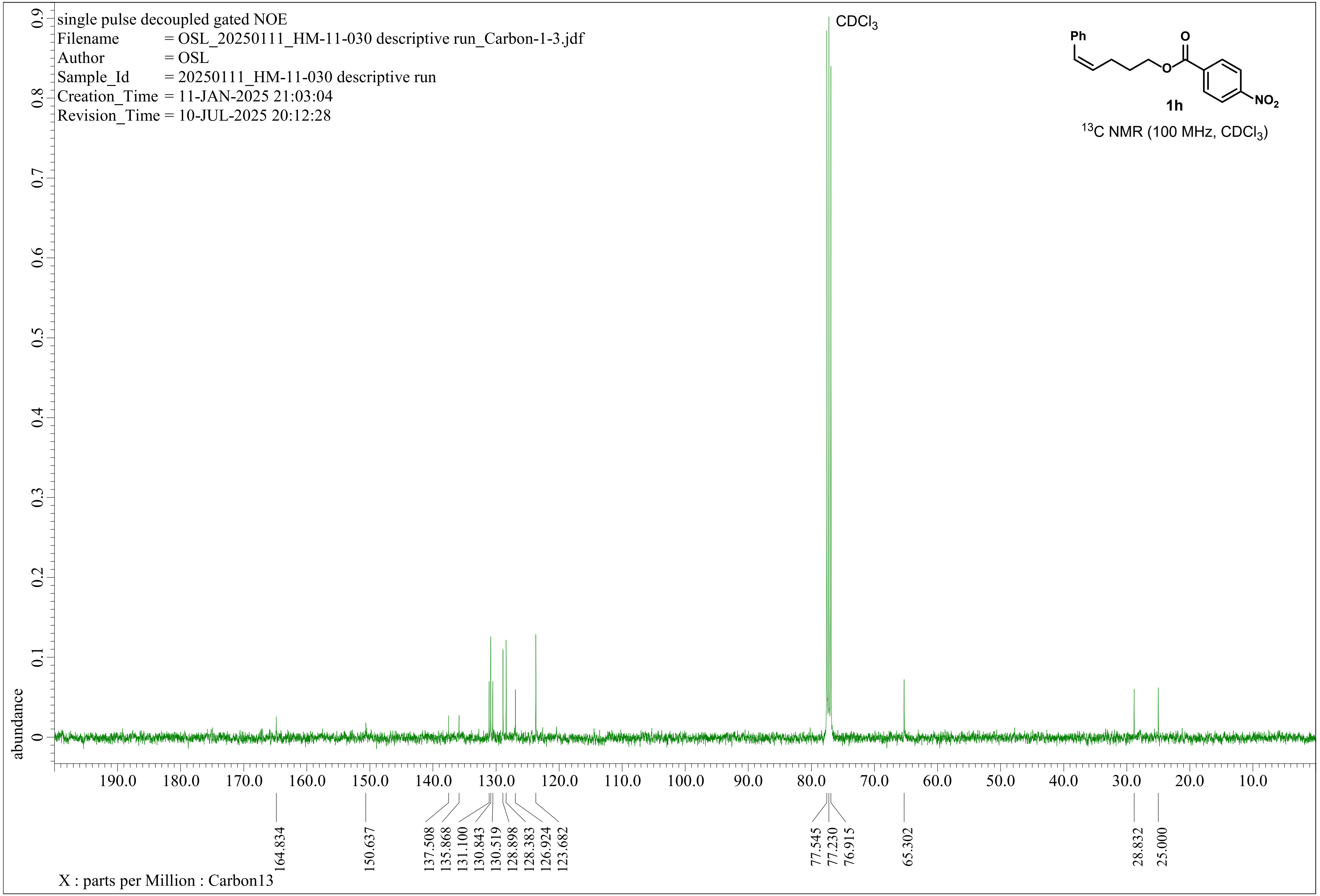

single pulse decoupled gated NOE
Filename = OSL_20250111_HM-11-030 descriptive run_Carbon-1-3.jdf
Author = OSL
Sample_Id = 20250111_HM-11-030 descriptive run
Creation_Time = 11-JAN-2025 21:03:04
Revision_Time = 10-JUL-2025 20:12:28
CDCl3
Ph
O
O
NO2
1h
13C NMR (100 MHz, CDCl3)
abundance
0.9
0.8
0.7
0.6
0.5
0.4
0.3
0.2
0.1
0
190.0
180.0
170.0
160.0
150.0
140.0
130.0
120.0
110.0
100.0
90.0
80.0
70.0
60.0
50.0
40.0
30.0
20.0
10.0
164.834
150.637
137.508
135.868
131.100
130.843
130.519
128.898
128.383
126.924
123.682
77.545
77.230
76.915
65.302
28.832
25.000
X : parts per Million : Carbon13

single_pulse
Filename = OSL_20250111_JYH-01-077 descriptive alkene_Proton-1-2.jdf
Author = OSL
Sample_Id = 20250111_JYH-01-077 descriptive alkene
Creation_Time = 11-JAN-2025 21:33:01
Revision_Time = 15-JUL-2025 15:49:05

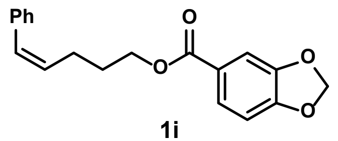

1i

^{1}H NMR (400 MHz, $CDCl_3$)

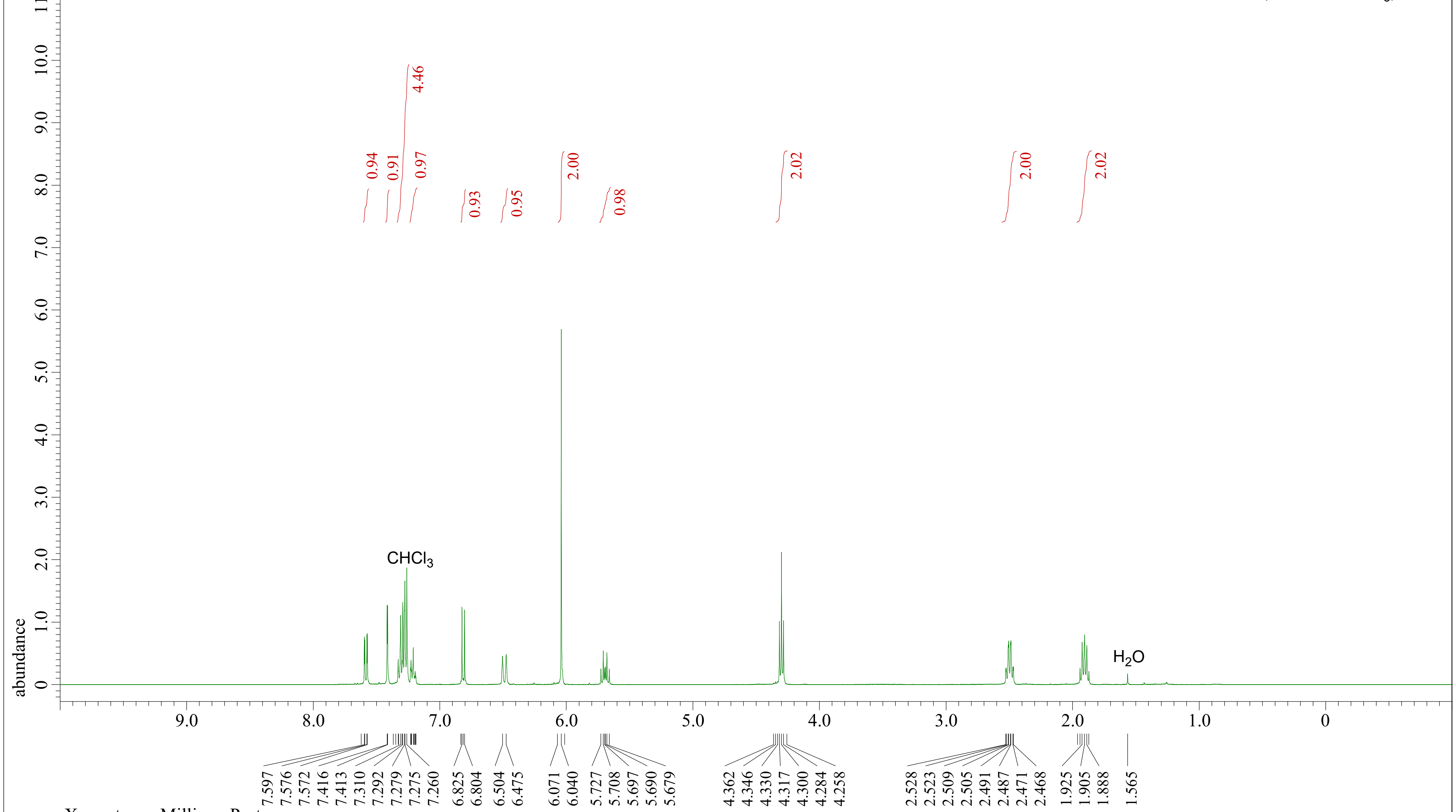

X : parts per Million : Proton

single pulse decoupled gated NOE
Filename = OSL_20250111_JYH-01-077 descriptive alkene_Carbon-1-2.jdf
Author = OSL
Sample_Id = 20250111_JYH-01-077 descriptive alkene
Creation_Time = 11-JAN-2025 21:34:59
Revision_Time = 15-JUL-2025 15:48:07

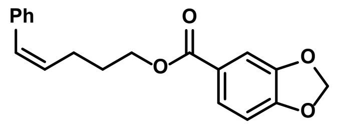

1i

^{13}C NMR (100 MHz, $CDCl_3$)

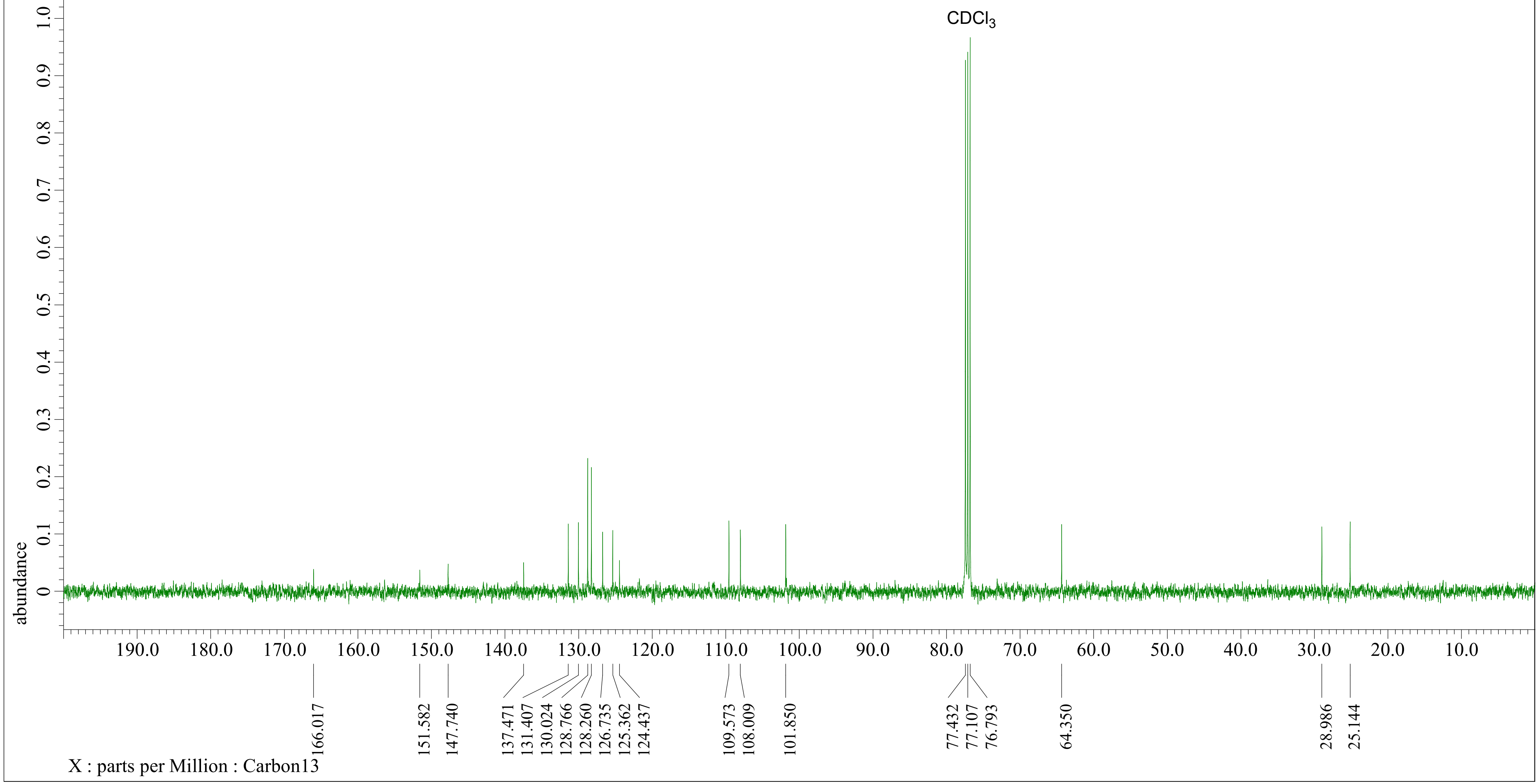

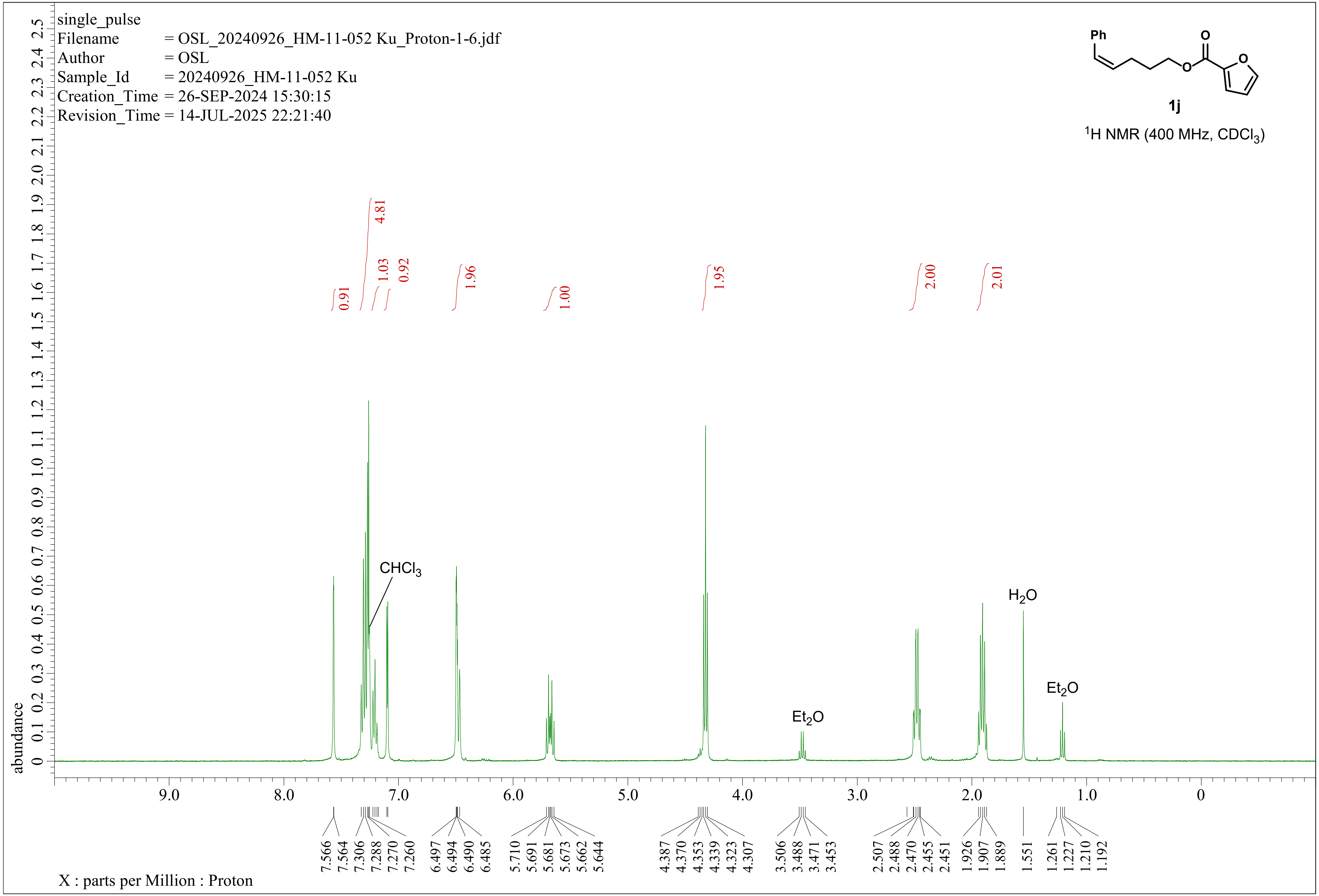
single_pulse
Filename = OSL_20240926_HM-11-052 Ku_Proton-1-6.jdf
Author = OSL
Sample_Id = 20240926_HM-11-052 Ku
Creation_Time = 26-SEP-2024 15:30:15
Revision_Time = 14-JUL-2025 22:21:40
Ph
O
O
O
1j
1H NMR (400 MHz, CDCl3)
CHCl3
H2O
Et2O
Et2O
0.91
4.81
1.03
0.92
1.96
1.00
1.95
2.00
2.01
7.566
7.564
7.306
7.288
7.270
7.260
6.497
6.494
6.490
6.485
5.710
5.691
5.681
5.673
5.662
5.644
4.387
4.370
4.353
4.339
4.323
4.307
3.506
3.488
3.471
3.453
2.507
2.488
2.470
2.455
2.451
1.926
1.907
1.889
1.551
1.261
1.227
1.210
1.192
abundance
X : parts per Million : Proton

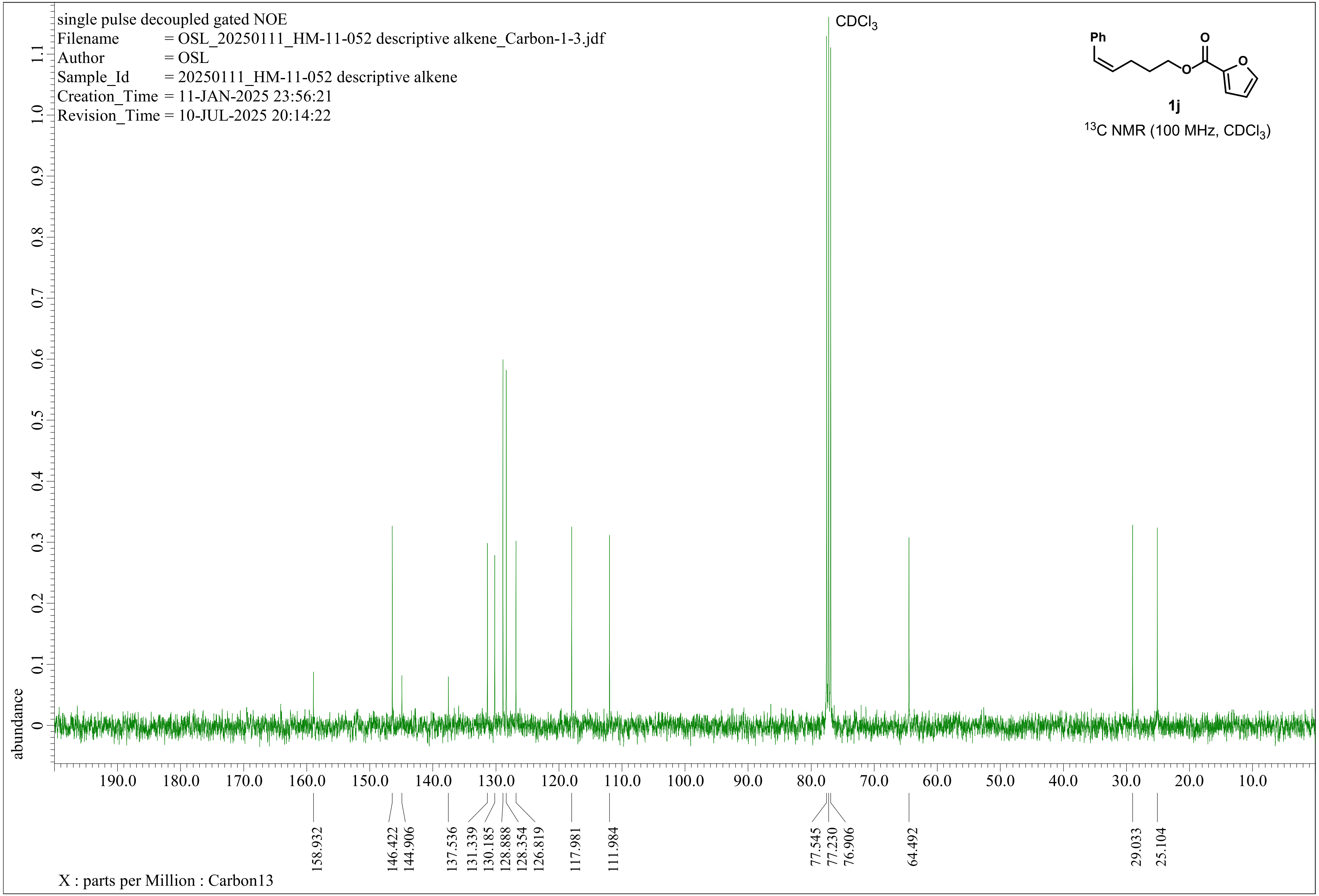
single pulse decoupled gated NOE
Filename = OSL_20250111_HM-11-052 descriptive alkene_Carbon-1-3.jdf
Author = OSL
Sample_Id = 20250111_HM-11-052 descriptive alkene
Creation_Time = 11-JAN-2025 23:56:21
Revision_Time = 10-JUL-2025 20:14:22
CDCl3
Ph
O
O
O
1j
13C NMR (100 MHz, CDCl3)
abundance
158.932
146.422
144.906
137.536
131.339
130.185
128.888
128.354
126.819
117.981
111.984
77.545
77.230
76.906
64.492
29.033
25.104
X : parts per Million : Carbon13

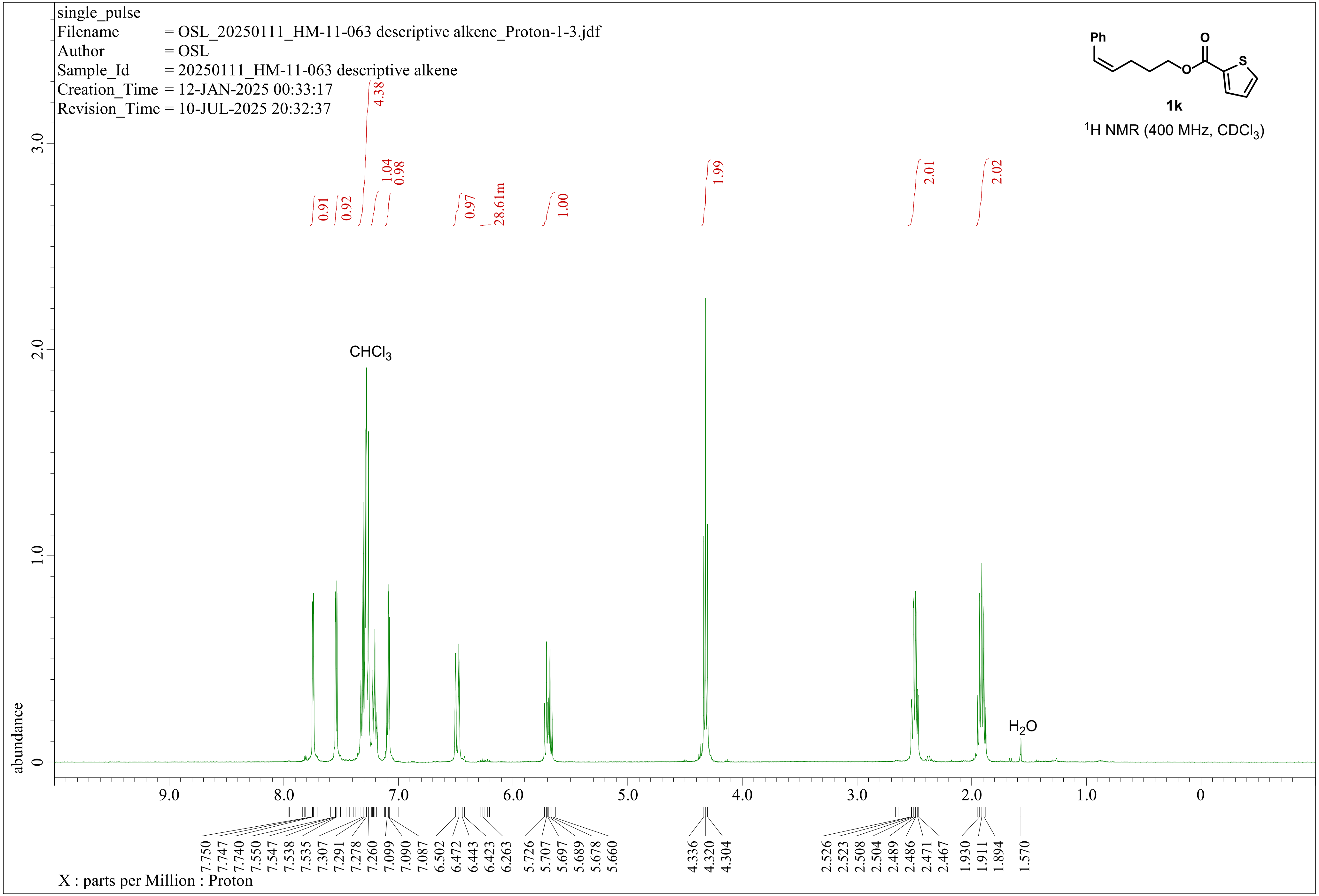
single_pulse
Filename = OSL_20250111_HM-11-063 descriptive alkene_Proton-1-3.jdf
Author = OSL
Sample_Id = 20250111_HM-11-063 descriptive alkene
Creation_Time = 12-JAN-2025 00:33:17
Revision_Time = 10-JUL-2025 20:32:37
Ph
O
S
O
1k
1H NMR (400 MHz, CDCl3)
CHCl3
H2O
0.91
0.92
4.38
1.04
0.98
0.97
28.61m
1.00
1.99
2.01
2.02
abundance
3.0
2.0
1.0
0
9.0
8.0
7.0
6.0
5.0
4.0
3.0
2.0
1.0
0
7.750
7.747
7.740
7.550
7.547
7.538
7.535
7.307
7.291
7.278
7.260
7.099
7.090
7.087
6.502
6.472
6.443
6.423
6.263
5.726
5.707
5.697
5.689
5.678
5.660
4.336
4.320
4.304
2.526
2.523
2.508
2.504
2.489
2.486
2.471
2.467
1.930
1.911
1.894
1.570
X : parts per Million : Proton

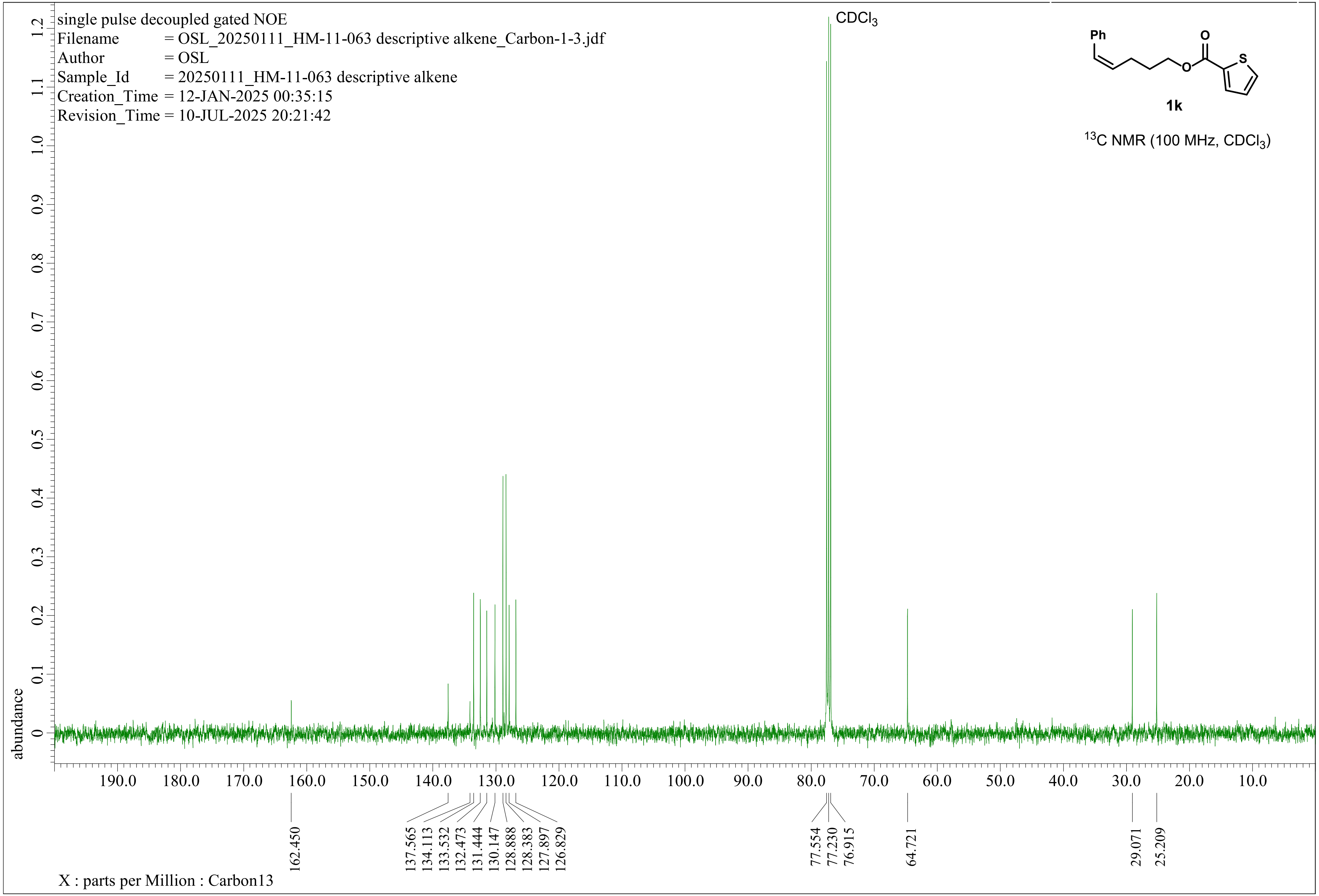

single pulse decoupled gated NOE
Filename = OSL_20250111_HM-11-063 descriptive alkene_Carbon-1-3.jdf
Author = OSL
Sample_Id = 20250111_HM-11-063 descriptive alkene
Creation_Time = 12-JAN-2025 00:35:15
Revision_Time = 10-JUL-2025 20:21:42
CDCl3
Ph
O
S
O
1k
13C NMR (100 MHz, CDCl3)
abundance
162.450
137.565
134.113
133.532
132.473
131.444
130.147
128.888
128.383
127.897
126.829
77.554
77.230
76.915
64.721
29.071
25.209
X : parts per Million : Carbon13

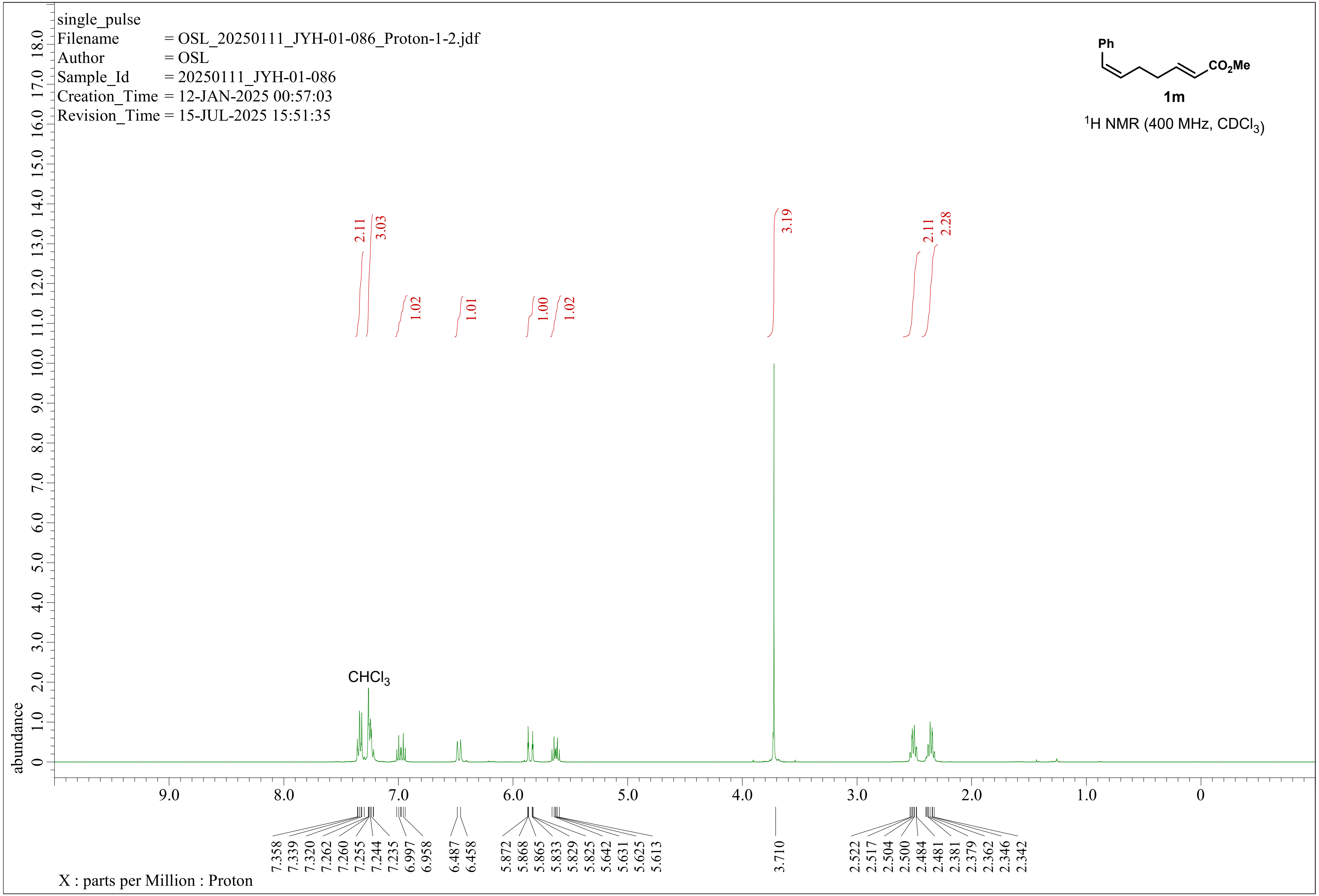
single_pulse
Filename = OSL_20250111_JYH-01-086_Proton-1-2.jdf
Author = OSL
Sample_Id = 20250111_JYH-01-086
Creation_Time = 12-JAN-2025 00:57:03
Revision_Time = 15-JUL-2025 15:51:35
Ph
CO2Me
1m
1H NMR (400 MHz, CDCl3)
CHCl3
2.11
3.03
1.02
1.01
1.00
1.02
3.19
2.11
2.28
7.358 7.339 7.320 7.262 7.260 7.255 7.244 7.235 6.997 6.958
6.487 6.458
5.872 5.868 5.865 5.833 5.829 5.825 5.642 5.631 5.625 5.613
3.710
2.522 2.517 2.504 2.500 2.484 2.481 2.381 2.379 2.362 2.346 2.342
abundance
X : parts per Million : Proton

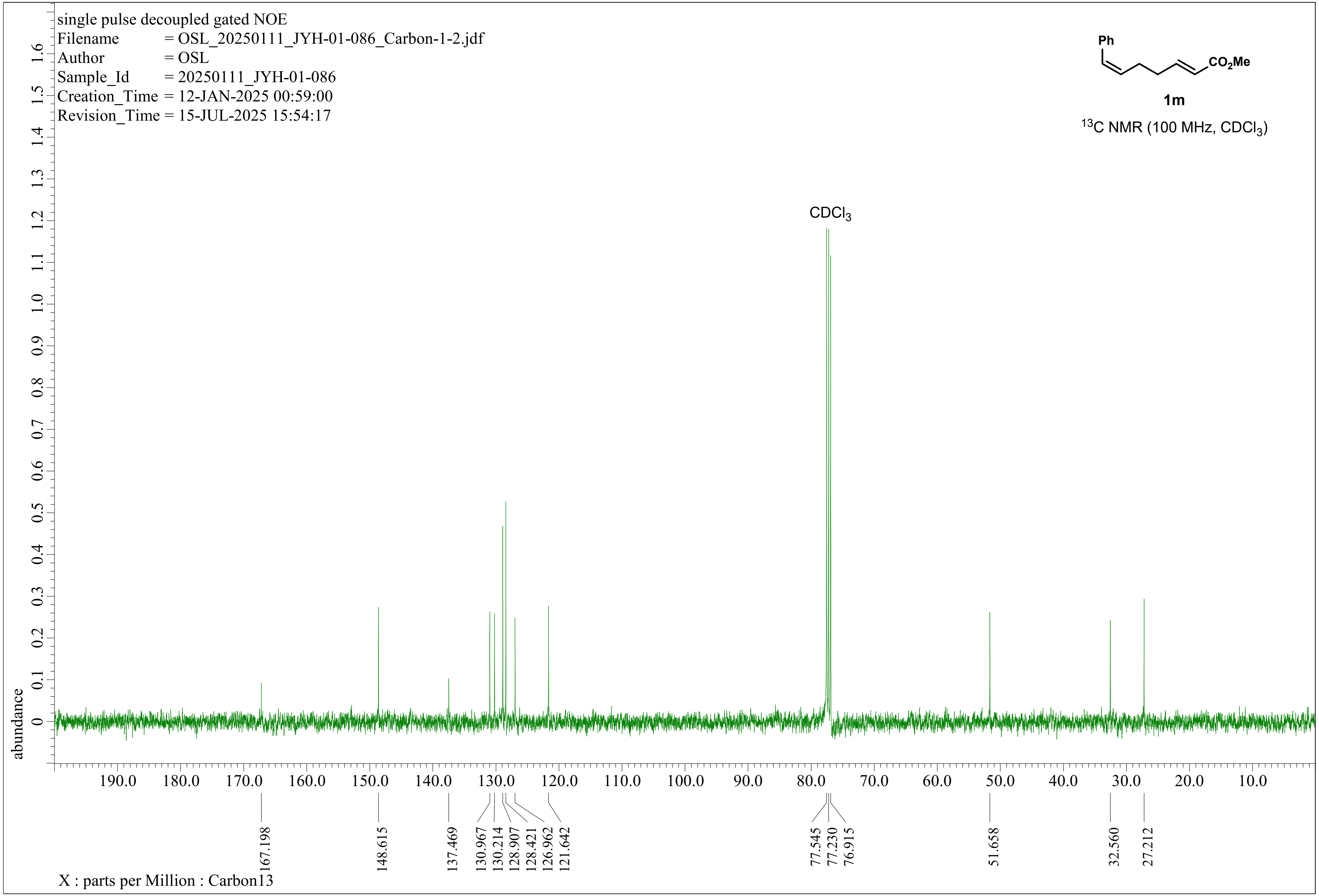
single pulse decoupled gated NOE
Filename = OSL_20250111_JYH-01-086_Carbon-1-2.jdf
Author = OSL
Sample_Id = 20250111_JYH-01-086
Creation_Time = 12-JAN-2025 00:59:00
Revision_Time = 15-JUL-2025 15:54:17
Ph
CO2Me
1m
13C NMR (100 MHz, CDCl3)
CDCl3
abundance
X : parts per Million : Carbon13
167.198
148.615
137.469
130.967
130.214
128.907
128.421
126.962
121.642
77.545
77.230
76.915
51.658
32.560
27.212

single_pulse
Filename = OSL_20241021_JYH-01-098 Ku_Proton-1-2.jdf
Author = OSL
Sample_Id = 20241021_JYH-01-098 Ku
Creation_Time = 21-OCT-2024 14:05:31
Revision_Time = 15-JUL-2025 16:15:56

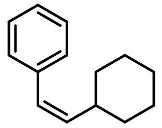

1n

^1H NMR (400 MHz, $CDCl_3$)

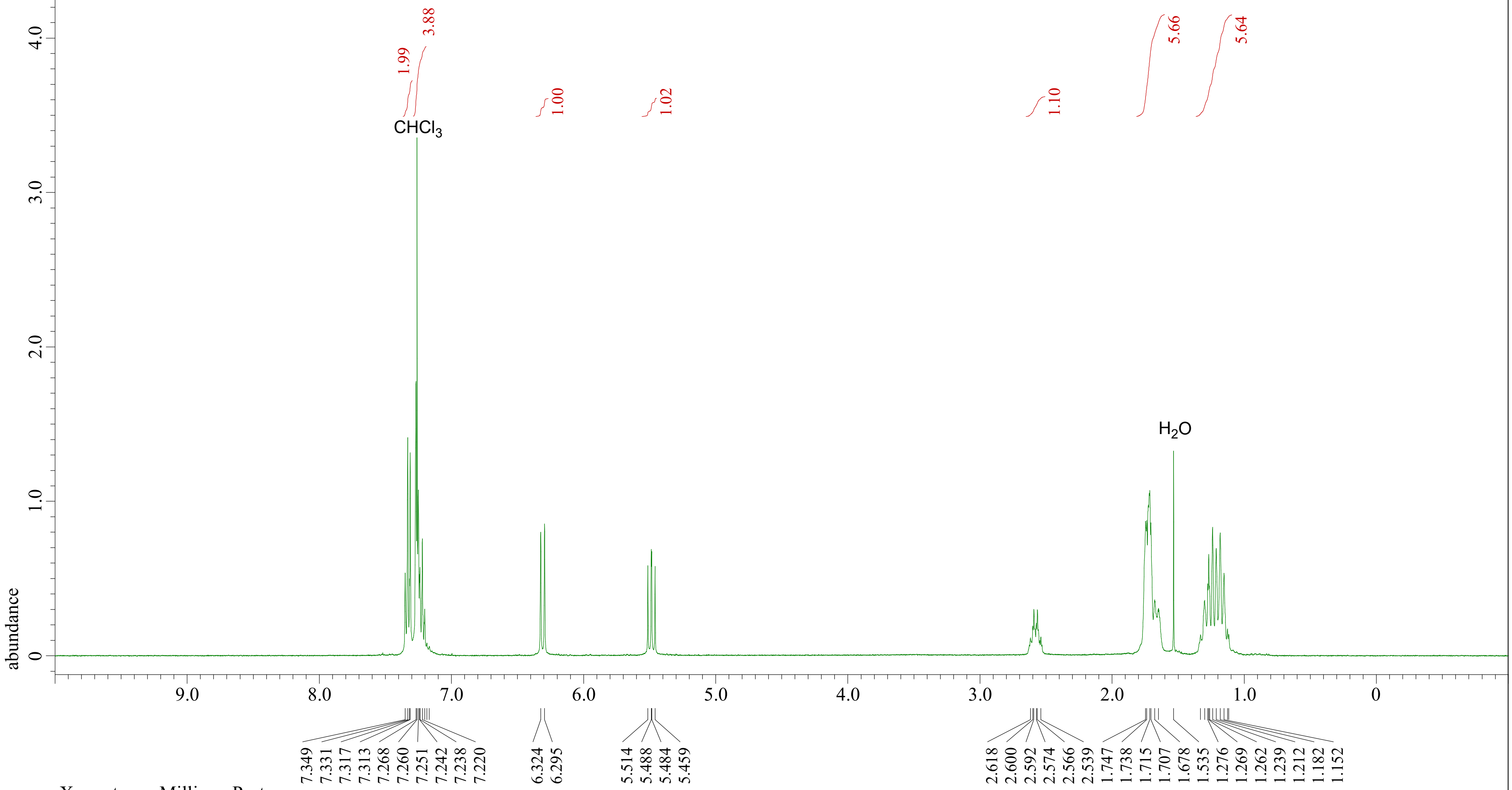

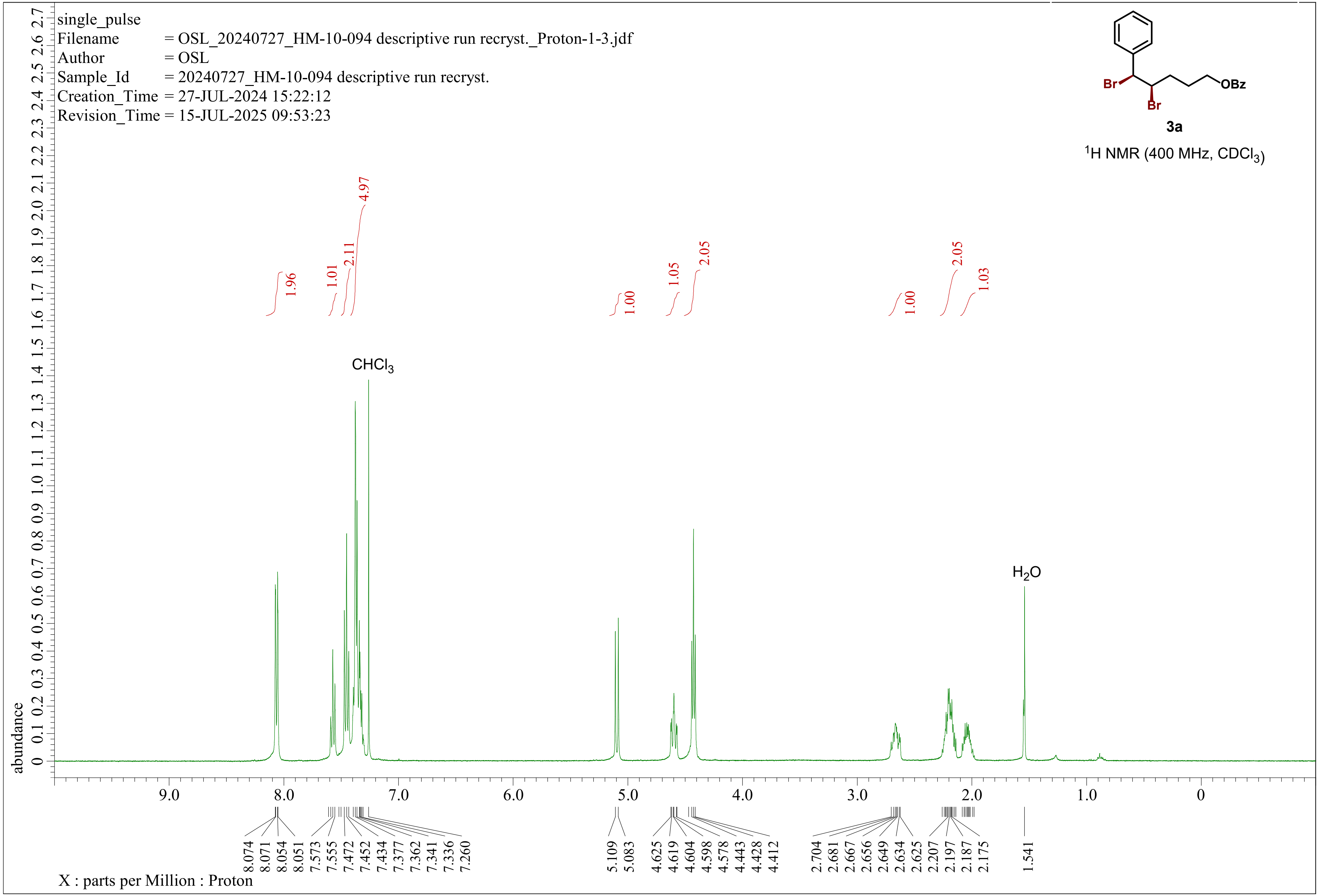
single_pulse
Filename = OSL_20240727_HM-10-094 descriptive run recryst._Proton-1-3.jdf
Author = OSL
Sample_Id = 20240727_HM-10-094 descriptive run recryst.
Creation_Time = 27-JUL-2024 15:22:12
Revision_Time = 15-JUL-2025 09:53:23
Br
Br
OBz
3a
1H NMR (400 MHz, CDCl3)
CHCl3
H2O
1.96
1.01
2.11
4.97
1.00
1.05
2.05
1.00
2.05
1.03
8.074
8.071
8.054
8.051
7.573
7.555
7.472
7.452
7.434
7.377
7.362
7.341
7.336
7.260
5.109
5.083
4.625
4.619
4.604
4.598
4.578
4.443
4.428
4.412
2.704
2.681
2.667
2.656
2.649
2.634
2.625
2.207
2.197
2.187
2.175
1.541
abundance
X : parts per Million : Proton

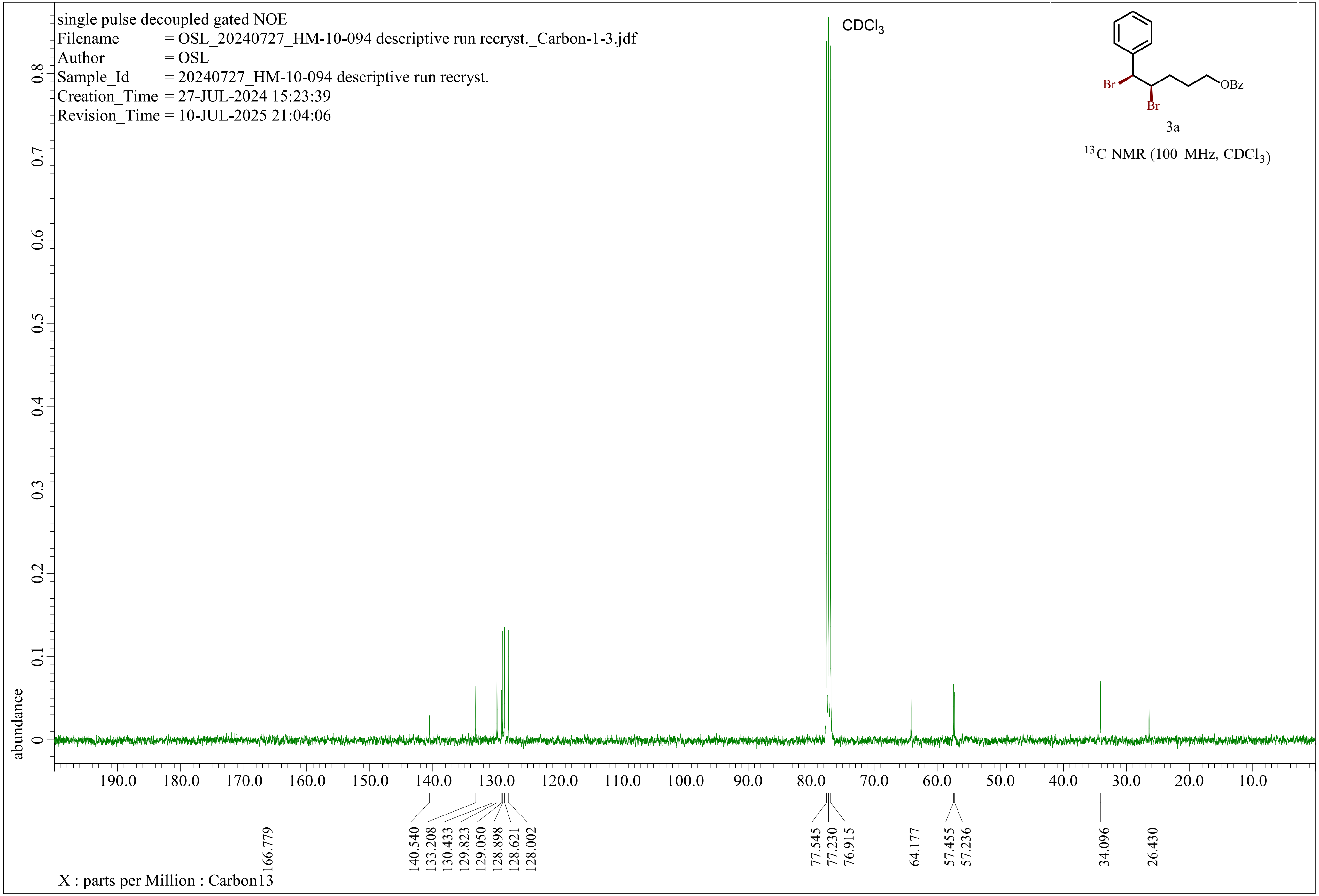
single pulse decoupled gated NOE
Filename = OSL_20240727_HM-10-094 descriptive run recryst._Carbon-1-3.jdf
Author = OSL
Sample_Id = 20240727_HM-10-094 descriptive run recryst.
Creation_Time = 27-JUL-2024 15:23:39
Revision_Time = 10-JUL-2025 21:04:06
CDCl3
Br
Br
OBz
3a
13C NMR (100 MHz, CDCl3)
abundance
166.779
140.540
133.208
130.433
129.823
129.050
128.898
128.621
128.002
77.545
77.230
76.915
64.177
57.455
57.236
34.096
26.430
X : parts per Million : Carbon13

single_pulse
Filename = OSL_20240730_HM-10-096 descriptive run_Proton-1-4.jdf
Author = OSL
Sample_Id = 20240730_HM-10-096 descriptive run
Creation_Time = 30-JUL-2024 19:03:59
Revision_Time = 15-JUL-2025 09:57:37

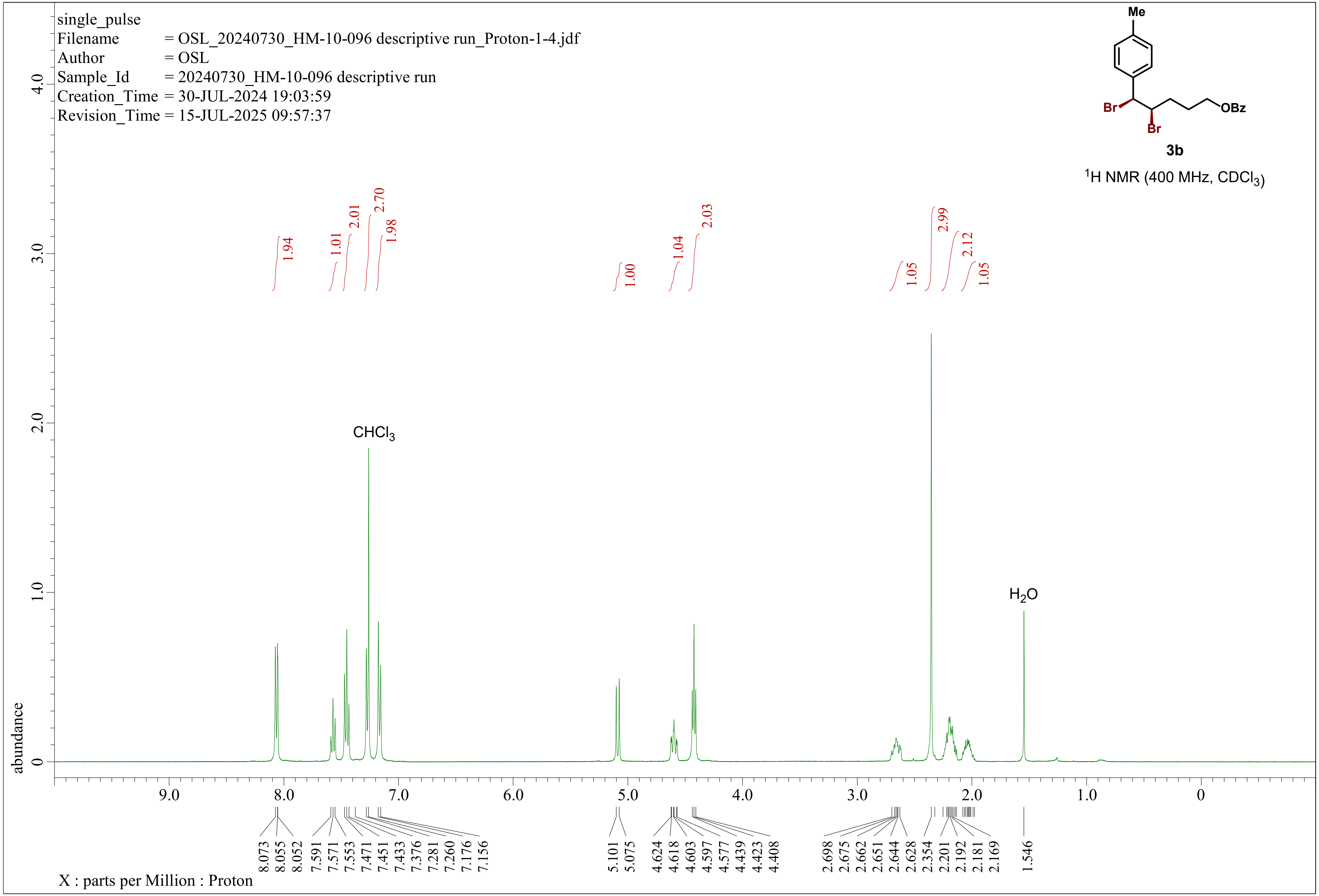

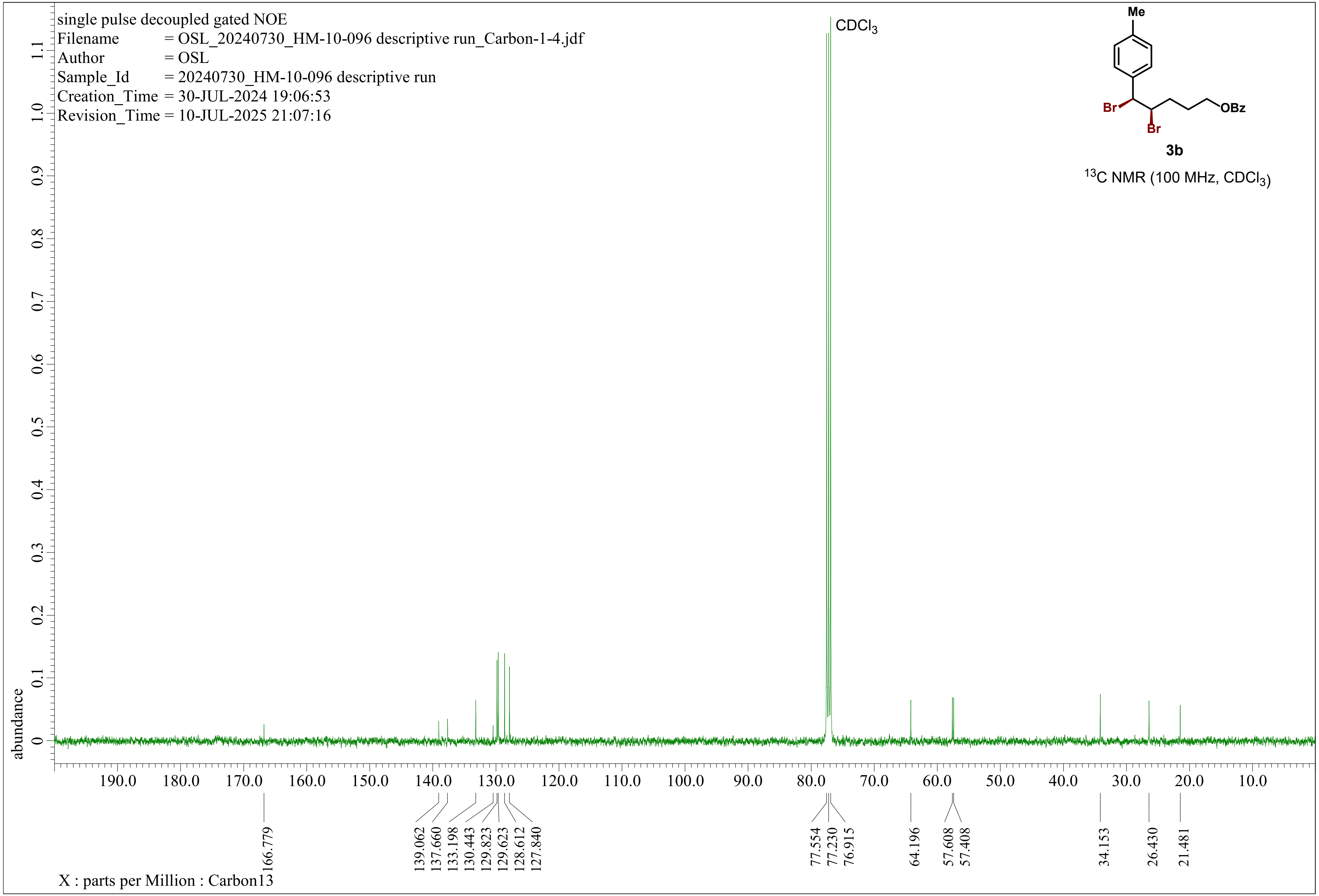
single pulse decoupled gated NOE
Filename = OSL_20240730_HM-10-096 descriptive run_Carbon-1-4.jdf
Author = OSL
Sample_Id = 20240730_HM-10-096 descriptive run
Creation_Time = 30-JUL-2024 19:06:53
Revision_Time = 10-JUL-2025 21:07:16
CDCl3
Me
Br
Br
OBz
3b
13C NMR (100 MHz, CDCl3)
abundance
X : parts per Million : Carbon13
166.779
139.062
137.660
133.198
130.443
129.823
129.623
128.612
127.840
77.554
77.230
76.915
64.196
57.608
57.408
34.153
26.430
21.481

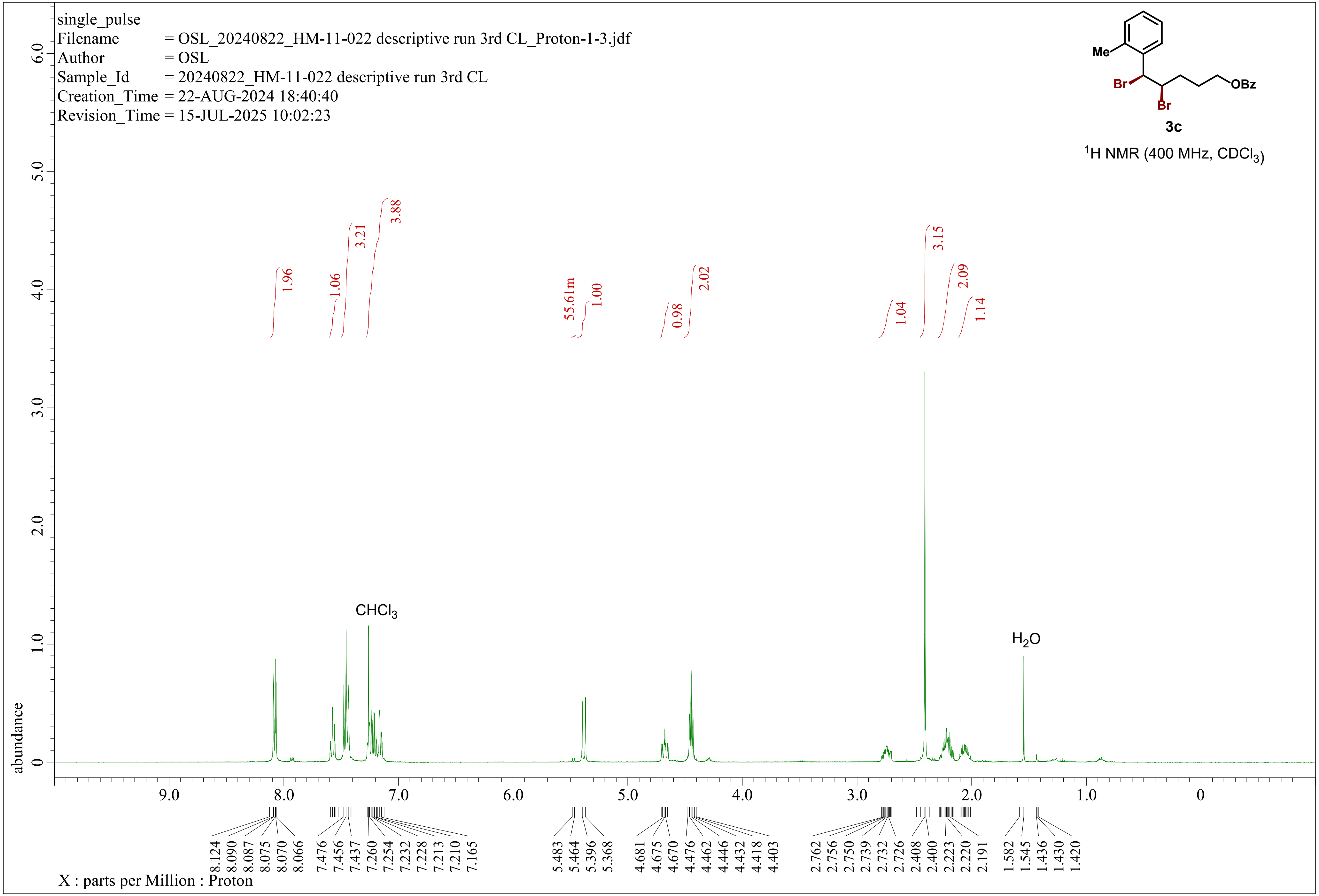
single_pulse
Filename = OSL_20240822_HM-11-022 descriptive run 3rd CL_Proton-1-3.jdf
Author = OSL
Sample_Id = 20240822_HM-11-022 descriptive run 3rd CL
Creation_Time = 22-AUG-2024 18:40:40
Revision_Time = 15-JUL-2025 10:02:23
Me
Br
Br
OBz
3c
1H NMR (400 MHz, CDCl3)
1.96
1.06
3.21
3.88
55.61m
1.00
0.98
2.02
1.04
3.15
2.09
1.14
CHCl3
H2O
abundance
6.0
5.0
4.0
3.0
2.0
1.0
0
9.0
8.0
7.0
6.0
5.0
4.0
3.0
2.0
1.0
0
8.124
8.090
8.087
8.075
8.070
8.066
7.476
7.456
7.437
7.260
7.254
7.232
7.228
7.213
7.210
7.165
5.483
5.464
5.396
5.368
4.681
4.675
4.670
4.476
4.462
4.446
4.432
4.418
4.403
2.762
2.756
2.750
2.739
2.732
2.726
2.408
2.400
2.223
2.220
2.191
1.582
1.545
1.436
1.430
1.420
X : parts per Million : Proton

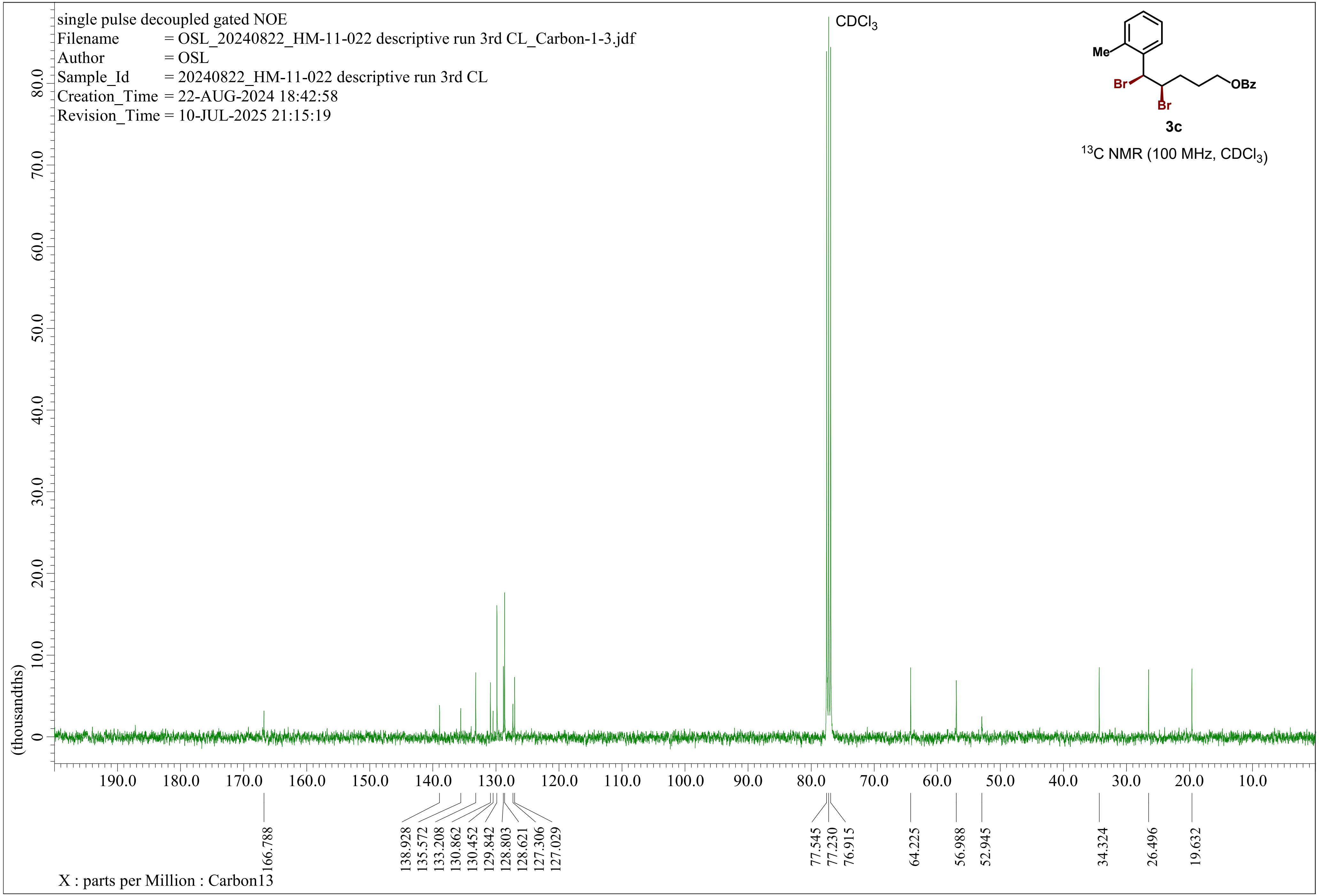
single pulse decoupled gated NOE
Filename = OSL_20240822_HM-11-022 descriptive run 3rd CL_Carbon-1-3.jdf
Author = OSL
Sample_Id = 20240822_HM-11-022 descriptive run 3rd CL
Creation_Time = 22-AUG-2024 18:42:58
Revision_Time = 10-JUL-2025 21:15:19
CDCl3
Me
Br
Br
OBz
3c
13C NMR (100 MHz, CDCl3)
(thousandths)
166.788
138.928
135.572
133.208
130.862
130.452
129.842
128.803
128.621
127.306
127.029
77.545
77.230
76.915
64.225
56.988
52.945
34.324
26.496
19.632
X : parts per Million : Carbon13

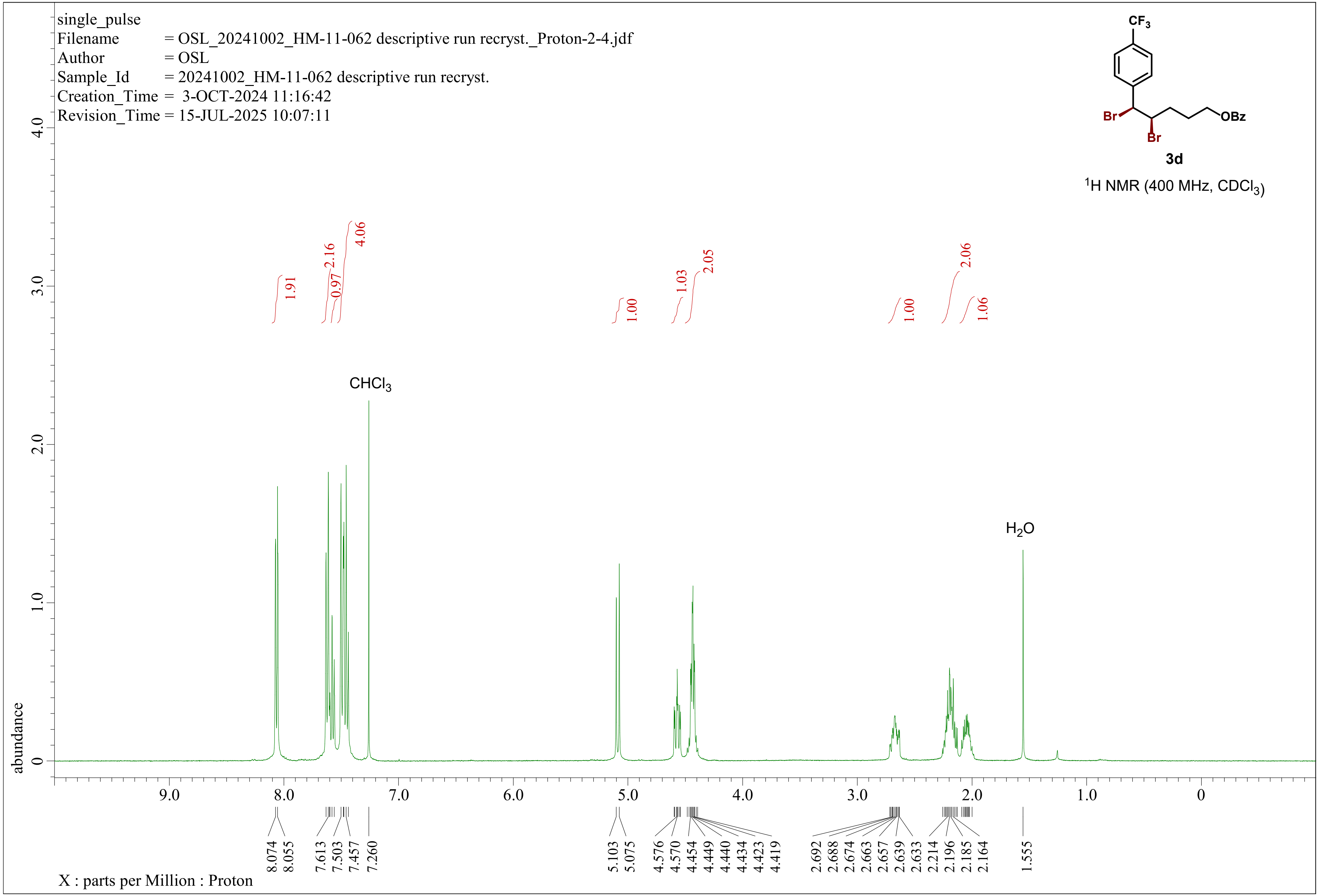

single_pulse
Filename = OSL_20241002_HM-11-062 descriptive run recryst._Proton-2-4.jdf
Author = OSL
Sample_Id = 20241002_HM-11-062 descriptive run recryst.
Creation_Time = 3-OCT-2024 11:16:42
Revision_Time = 15-JUL-2025 10:07:11
CF3
Br
Br
OBz
3d
1H NMR (400 MHz, CDCl3)
CHCl3
H2O
1.91
2.16
0.97
4.06
1.00
1.03
2.05
1.00
2.06
1.06
8.074
8.055
7.613
7.503
7.457
7.260
5.103
5.075
4.576
4.570
4.454
4.449
4.440
4.434
4.423
4.419
2.692
2.688
2.674
2.663
2.657
2.639
2.633
2.214
2.196
2.185
2.164
1.555
abundance
X : parts per Million : Proton

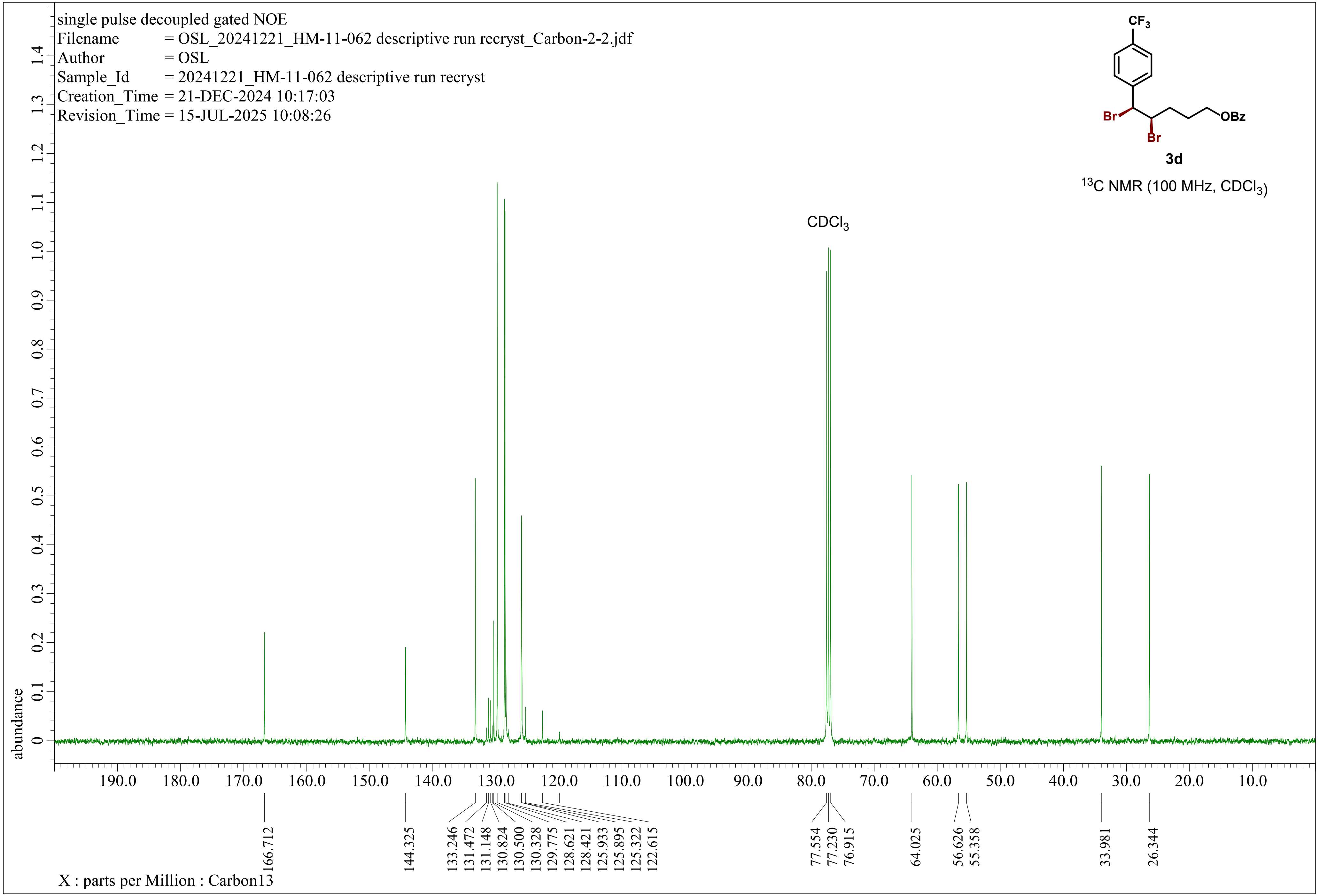
single pulse decoupled gated NOE
Filename = OSL_20241221_HM-11-062 descriptive run recryst_Carbon-2-2.jdf
Author = OSL
Sample_Id = 20241221_HM-11-062 descriptive run recryst
Creation_Time = 21-DEC-2024 10:17:03
Revision_Time = 15-JUL-2025 10:08:26
CF3
Br
Br
OBz
3d
13C NMR (100 MHz, CDCl3)
CDCl3
abundance
166.712
144.325
133.246
131.472
131.148
130.824
130.500
130.328
129.775
128.621
128.421
125.933
125.895
125.322
122.615
77.554
77.230
76.915
64.025
56.626
55.358
33.981
26.344
X : parts per Million : Carbon13

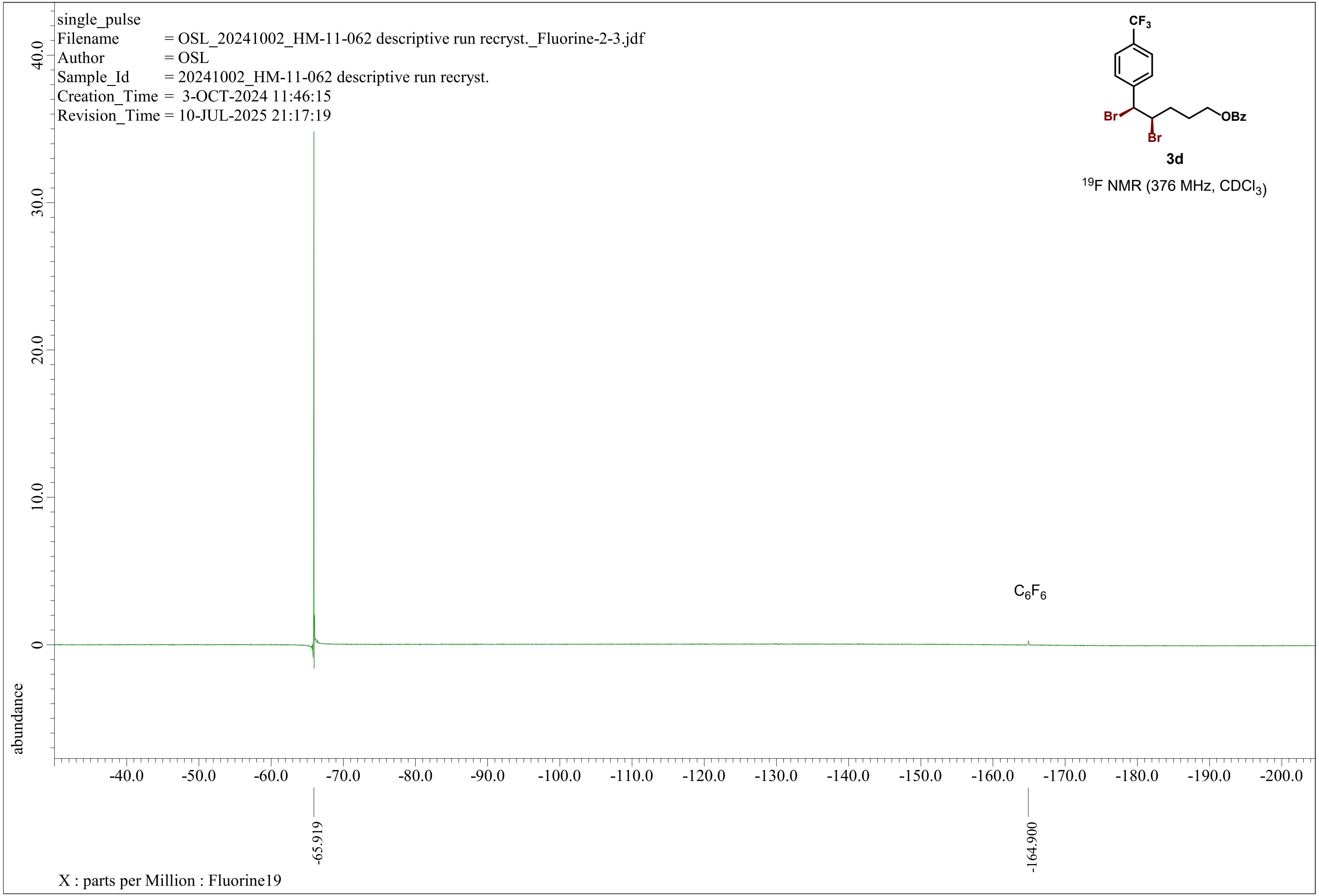

single_pulse
Filename = OSL_20241002_HM-11-062 descriptive run recryst._Fluorine-2-3.jdf
Author = OSL
Sample_Id = 20241002_HM-11-062 descriptive run recryst.
Creation_Time = 3-OCT-2024 11:46:15
Revision_Time = 10-JUL-2025 21:17:19
CF3
Br
Br
OBz
3d
19F NMR (376 MHz, CDCl3)
C6F6
abundance
40.0
30.0
20.0
10.0
0
-40.0
-50.0
-60.0
-70.0
-80.0
-90.0
-100.0
-110.0
-120.0
-130.0
-140.0
-150.0
-160.0
-170.0
-180.0
-190.0
-200.0
-65.919
-164.900
X : parts per Million : Fluorine19

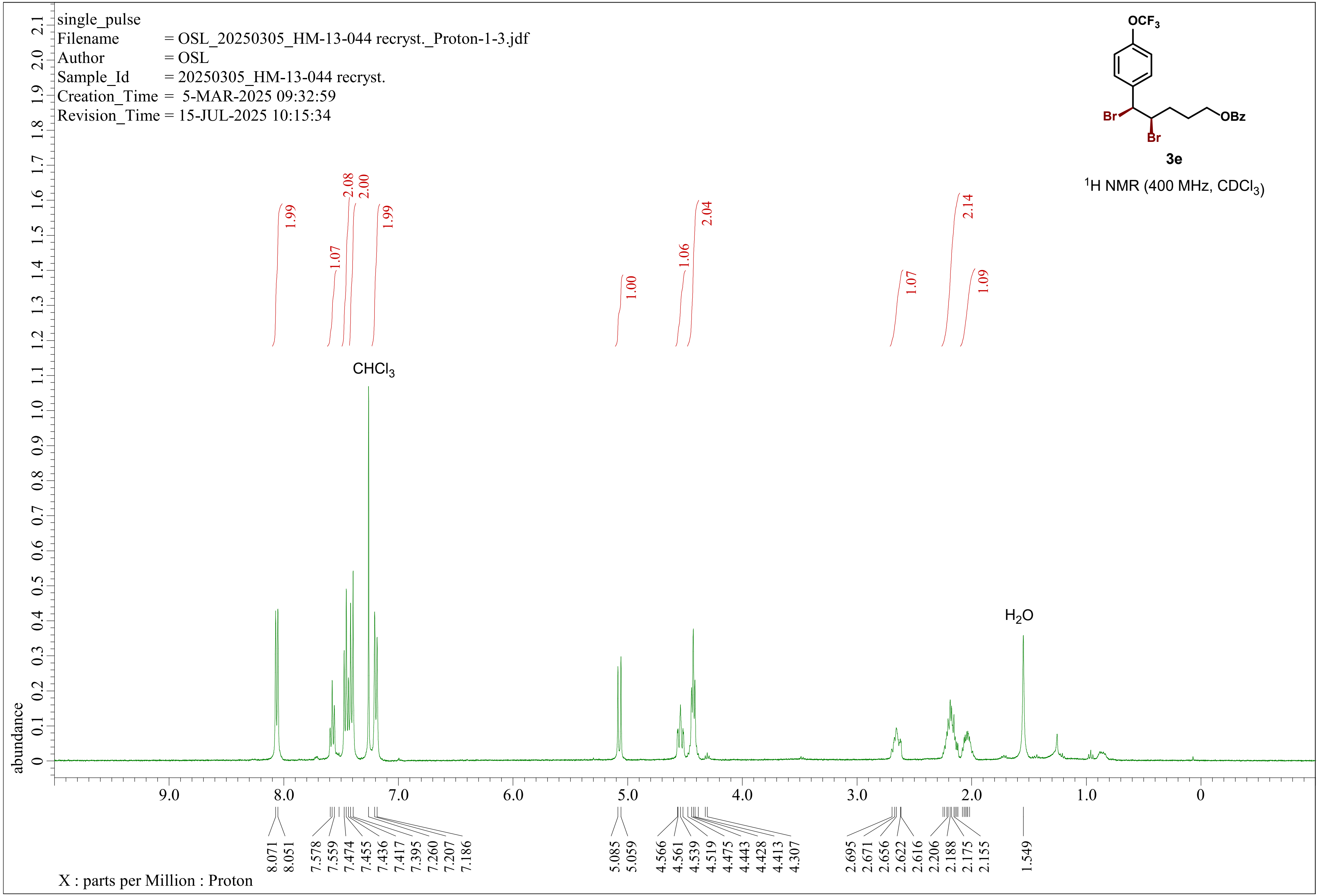

single_pulse
Filename = OSL_20250305_HM-13-044 recryst._Proton-1-3.jdf
Author = OSL
Sample_Id = 20250305_HM-13-044 recryst.
Creation_Time = 5-MAR-2025 09:32:59
Revision_Time = 15-JUL-2025 10:15:34
OCF3
Br
Br
OBz
3e
1H NMR (400 MHz, CDCl3)
CHCl3
H2O
abundance
X : parts per Million : Proton
8.071 8.051 7.578 7.559 7.474 7.455 7.436 7.417 7.395 7.260 7.207 7.186
5.085 5.059
4.566 4.561 4.539 4.519 4.475 4.443 4.428 4.413 4.307
2.695 2.671 2.656 2.622 2.616 2.206 2.188 2.175 2.155
1.549
1.99 1.07 2.08 2.00 1.99 1.00 1.06 2.04 1.07 2.14 1.09

single pulse decoupled gated NOE
Filename = OSL_20250305_HM-13-043 descriptive run recryst._Carbon-1-7.jdf
Author = OSL
Sample_Id = 20250305_HM-13-043 descriptive run recryst.
Creation_Time = 5-MAR-2025 23:39:23
Revision_Time = 10-JUL-2025 21:29:50

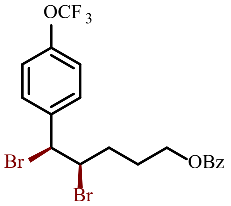

3e

^{13}C NMR (100 MHz, $CDCl_3$)

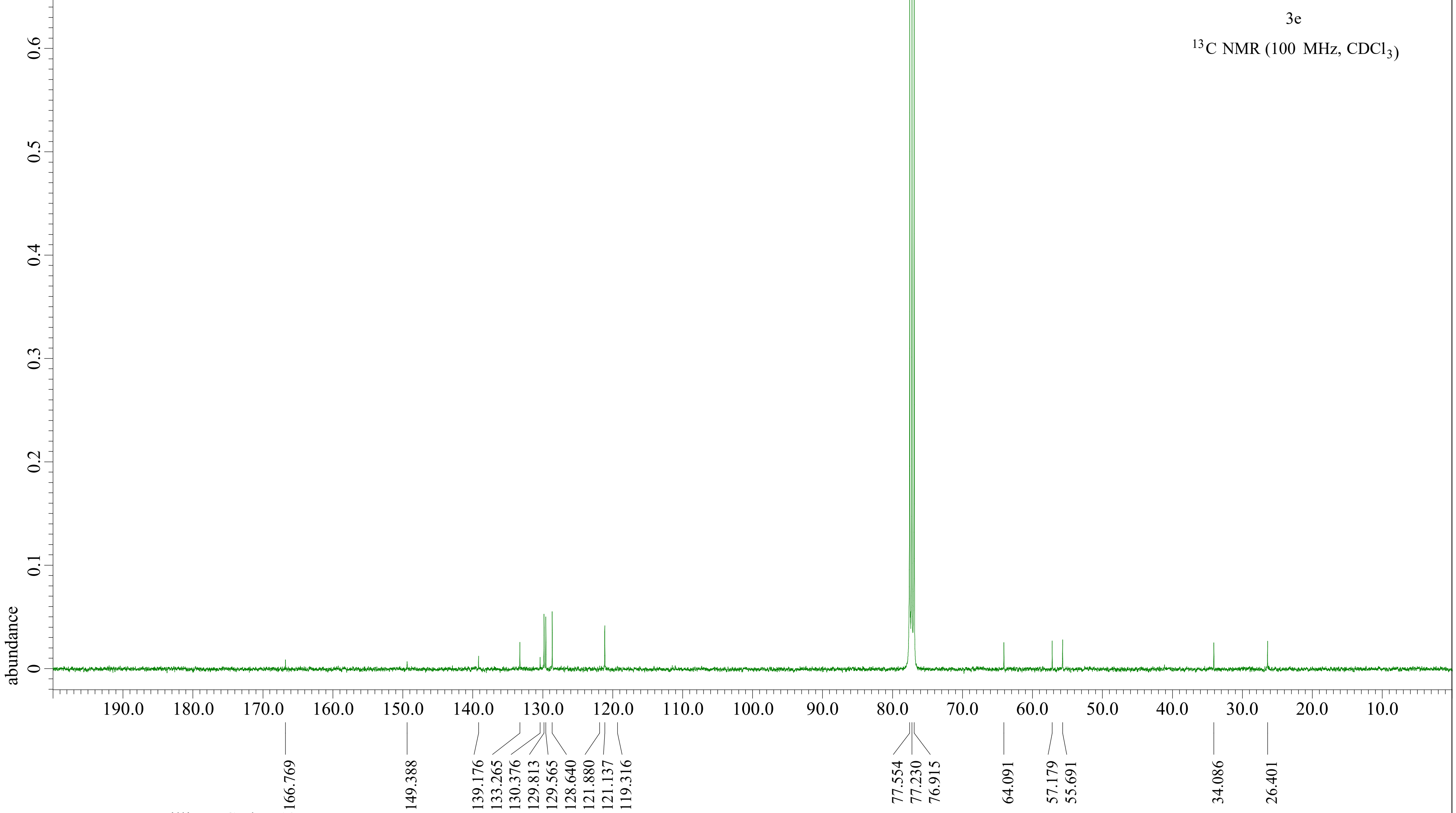

X : parts per Million : Carbon13

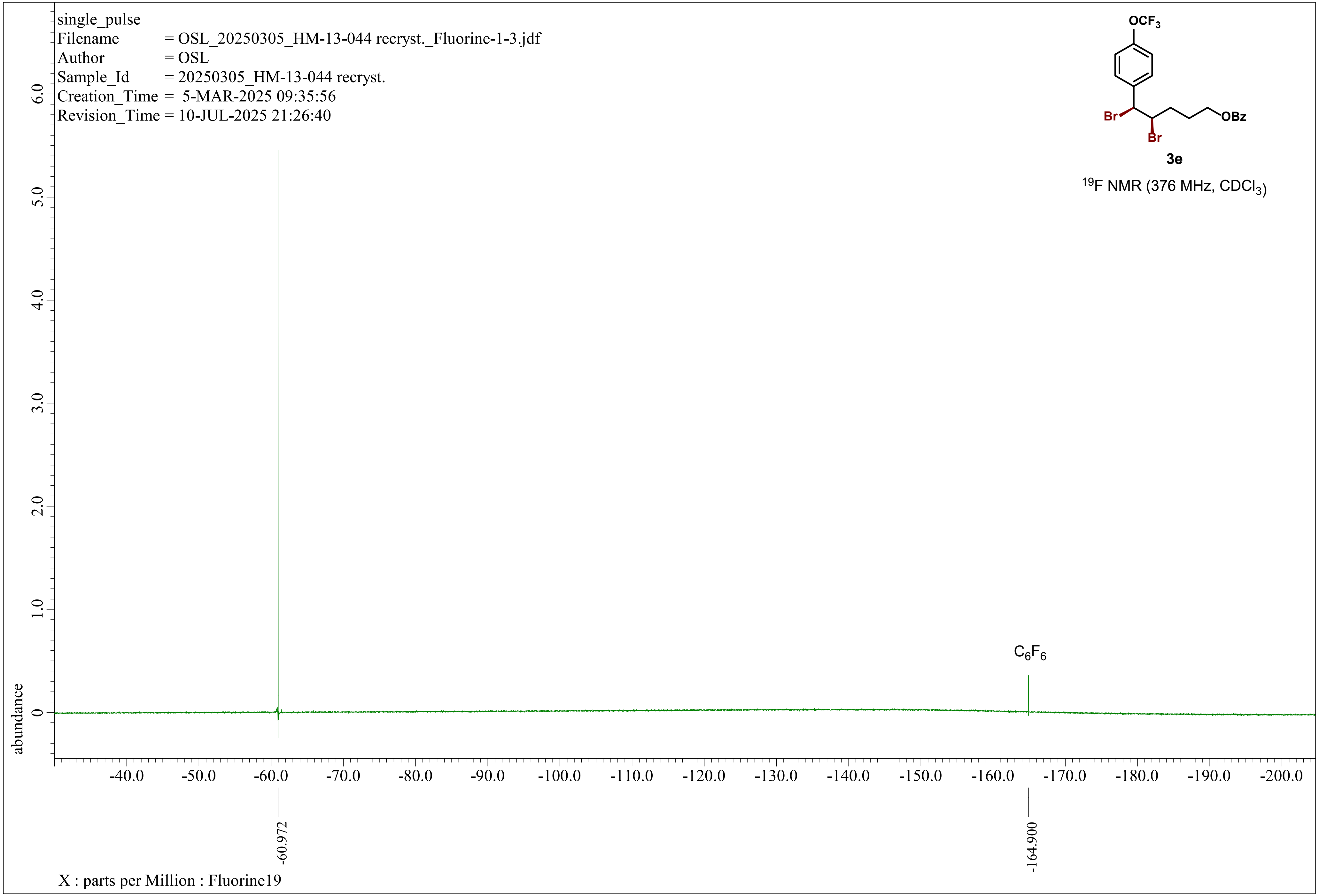
single_pulse
Filename = OSL_20250305_HM-13-044 recryst._Fluorine-1-3.jdf
Author = OSL
Sample_Id = 20250305_HM-13-044 recryst.
Creation_Time = 5-MAR-2025 09:35:56
Revision_Time = 10-JUL-2025 21:26:40
OCF3
Br
Br
OBz
3e
19F NMR (376 MHz, CDCl3)
abundance
6.0
5.0
4.0
3.0
2.0
1.0
0
C6F6
-40.0
-50.0
-60.0
-70.0
-80.0
-90.0
-100.0
-110.0
-120.0
-130.0
-140.0
-150.0
-160.0
-170.0
-180.0
-190.0
-200.0
-60.972
-164.900
X : parts per Million : Fluorine19

single_pulse
Filename = OSL_20241007_HM-11-067 descriptive run recryst._Proton-1-4.jdf
Author = OSL
Sample_Id = 20241007_HM-11-067 descriptive run recryst.
Creation_Time = 7-OCT-2024 14:01:34
Revision_Time = 15-JUL-2025 10:51:56

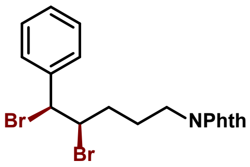

3f

^1H NMR (400 MHz, $CDCl_3$)

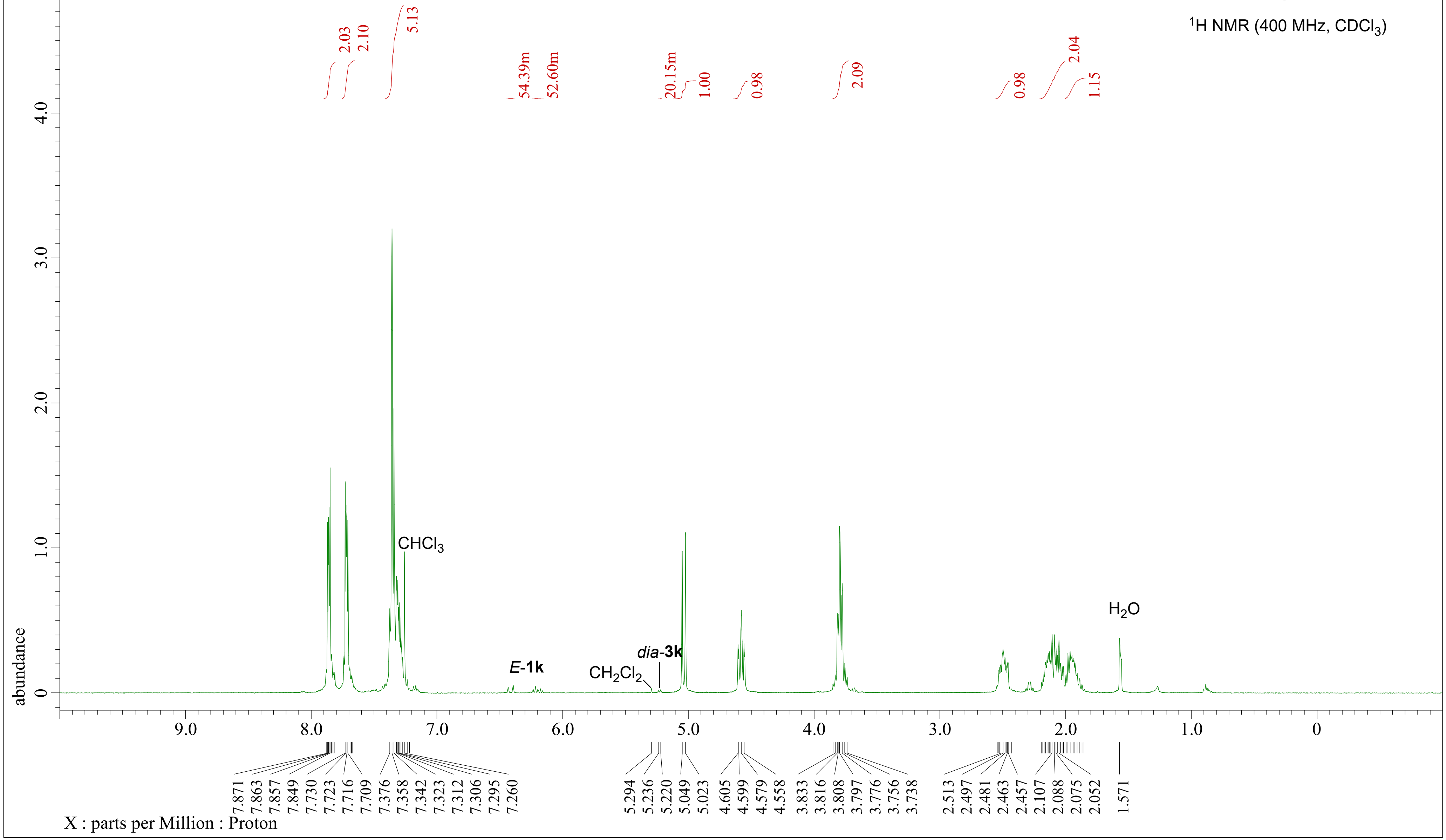

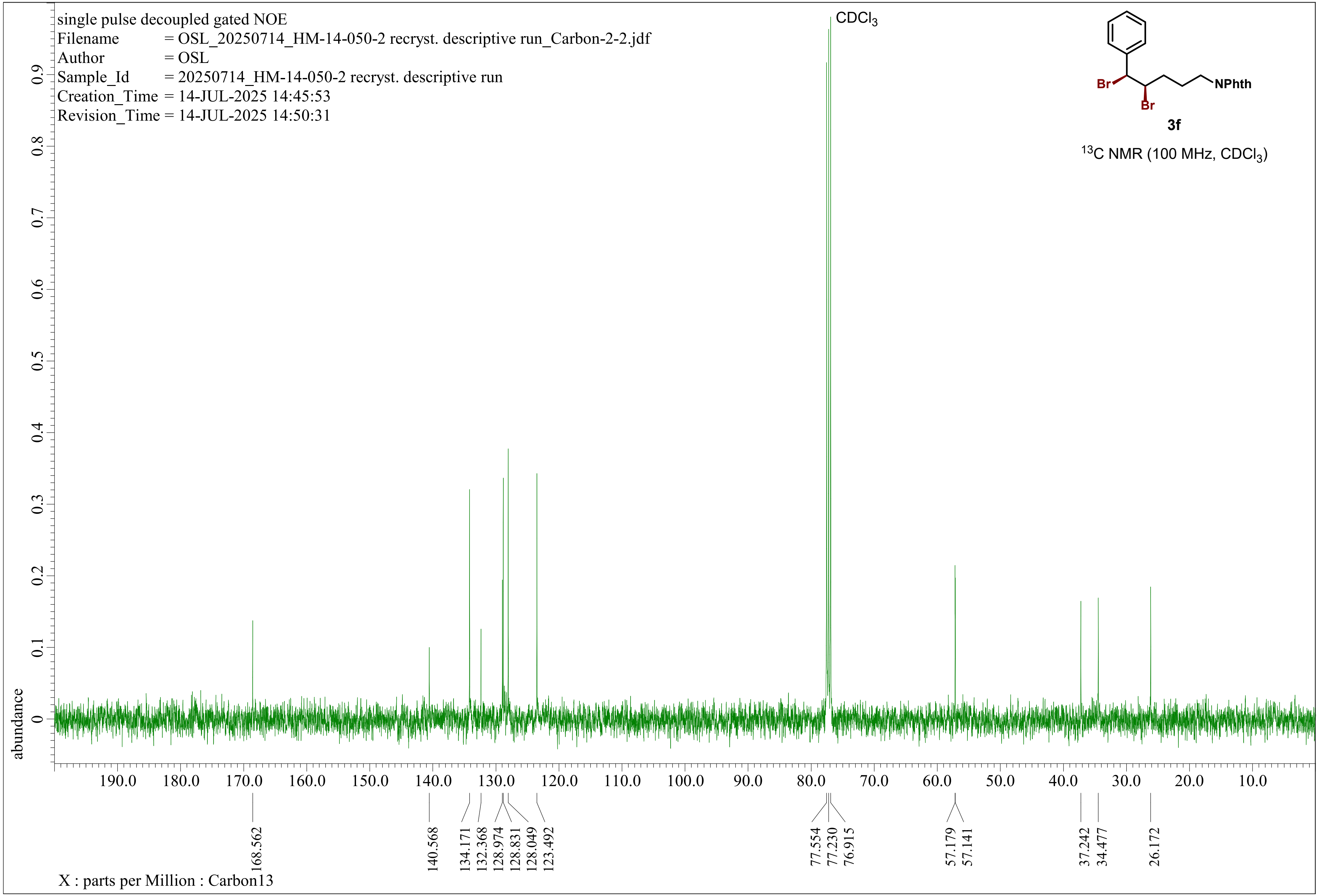
single pulse decoupled gated NOE
Filename = OSL_20250714_HM-14-050-2 recryst. descriptive run_Carbon-2-2.jdf
Author = OSL
Sample_Id = 20250714_HM-14-050-2 recryst. descriptive run
Creation_Time = 14-JUL-2025 14:45:53
Revision_Time = 14-JUL-2025 14:50:31
CDCl3
Br
Br
NPhth
3f
13C NMR (100 MHz, CDCl3)
abundance
168.562
140.568
134.171
132.368
128.974
128.831
128.049
123.492
77.554
77.230
76.915
57.179
57.141
37.242
34.477
26.172
X : parts per Million : Carbon13

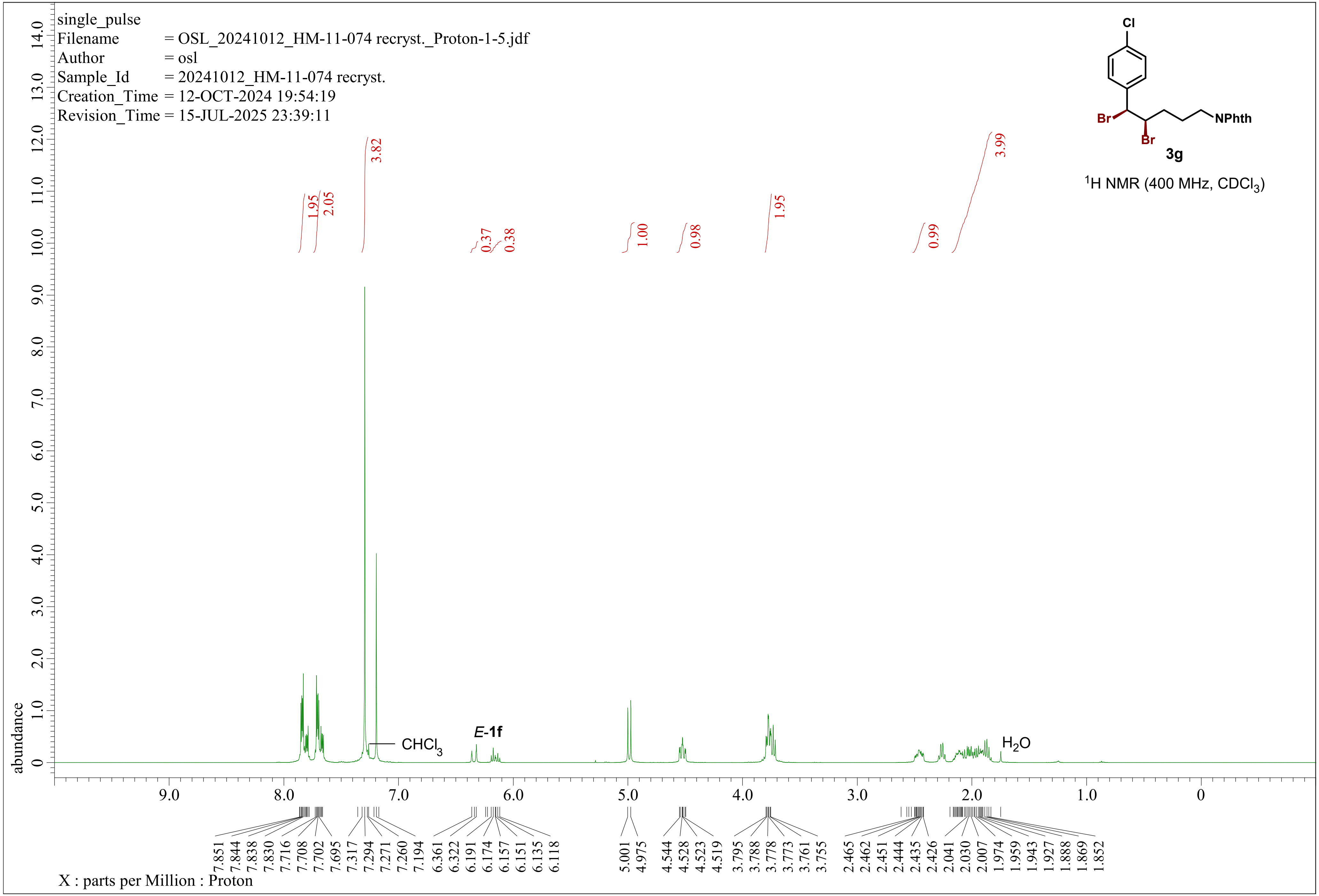

single_pulse
Filename = OSL_20241012_HM-11-074 recryst._Proton-1-5.jdf
Author = osl
Sample_Id = 20241012_HM-11-074 recryst.
Creation_Time = 12-OCT-2024 19:54:19
Revision_Time = 15-JUL-2025 23:39:11
Cl
Br
Br
NPhth
3g
1H NMR (400 MHz, CDCl3)
CHCl3
E-1f
H2O
abundance
X : parts per Million : Proton

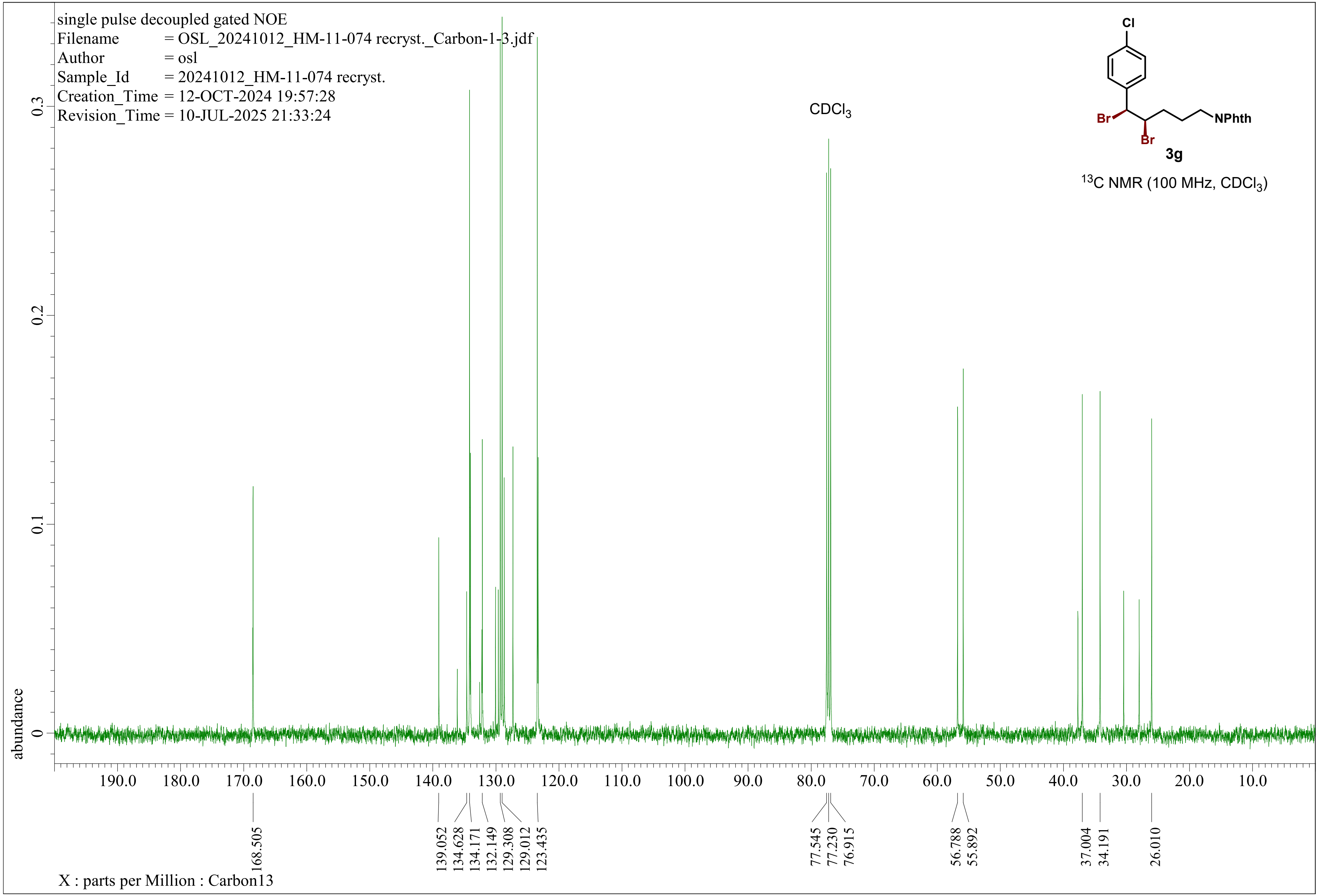
single pulse decoupled gated NOE
Filename = OSL_20241012_HM-11-074 recryst._Carbon-1-3.jdf
Author = osl
Sample_Id = 20241012_HM-11-074 recryst.
Creation_Time = 12-OCT-2024 19:57:28
Revision_Time = 10-JUL-2025 21:33:24
CDCl3
Cl
Br
Br
NPhth
3g
13C NMR (100 MHz, CDCl3)
abundance
0.3
0.2
0.1
0
190.0 180.0 170.0 160.0 150.0 140.0 130.0 120.0 110.0 100.0 90.0 80.0 70.0 60.0 50.0 40.0 30.0 20.0 10.0
168.505
139.052
134.628
134.171
132.149
129.308
129.012
123.435
77.545
77.230
76.915
56.788
55.892
37.004
34.191
26.010
X : parts per Million : Carbon13

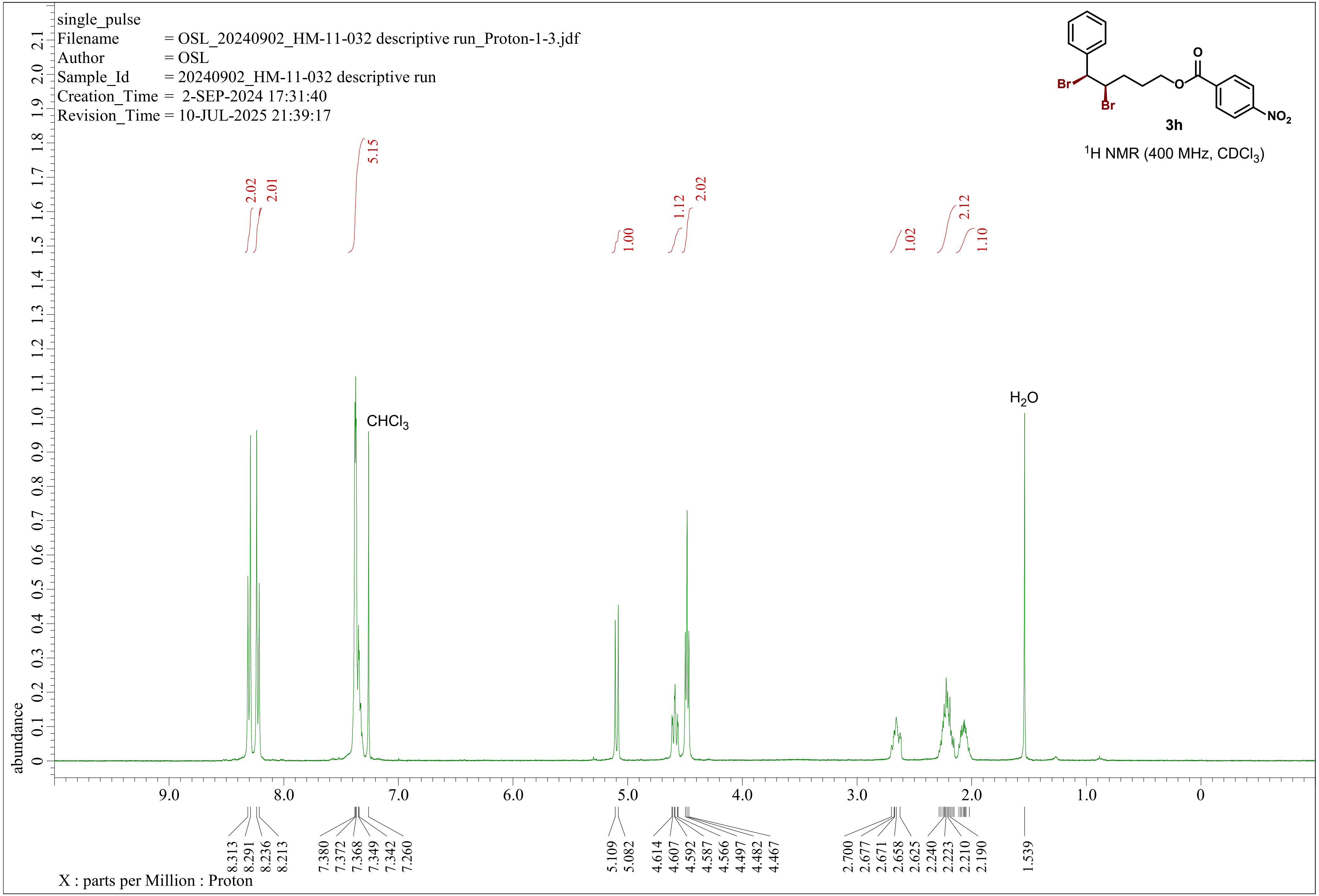

single_pulse
Filename = OSL_20240902_HM-11-032 descriptive run_Proton-1-3.jdf
Author = OSL
Sample_Id = 20240902_HM-11-032 descriptive run
Creation_Time = 2-SEP-2024 17:31:40
Revision_Time = 10-JUL-2025 21:39:17
3h
1H NMR (400 MHz, CDCl3)
CHCl3
H2O
abundance
X : parts per Million : Proton
2.02 2.01 5.15 1.00 1.12 2.02 1.02 2.12 1.10
8.313 8.291 8.236 8.213 7.380 7.372 7.368 7.349 7.342 7.260 5.109 5.082 4.614 4.607 4.592 4.587 4.566 4.497 4.482 4.467 2.700 2.677 2.671 2.658 2.625 2.240 2.223 2.210 2.190 1.539

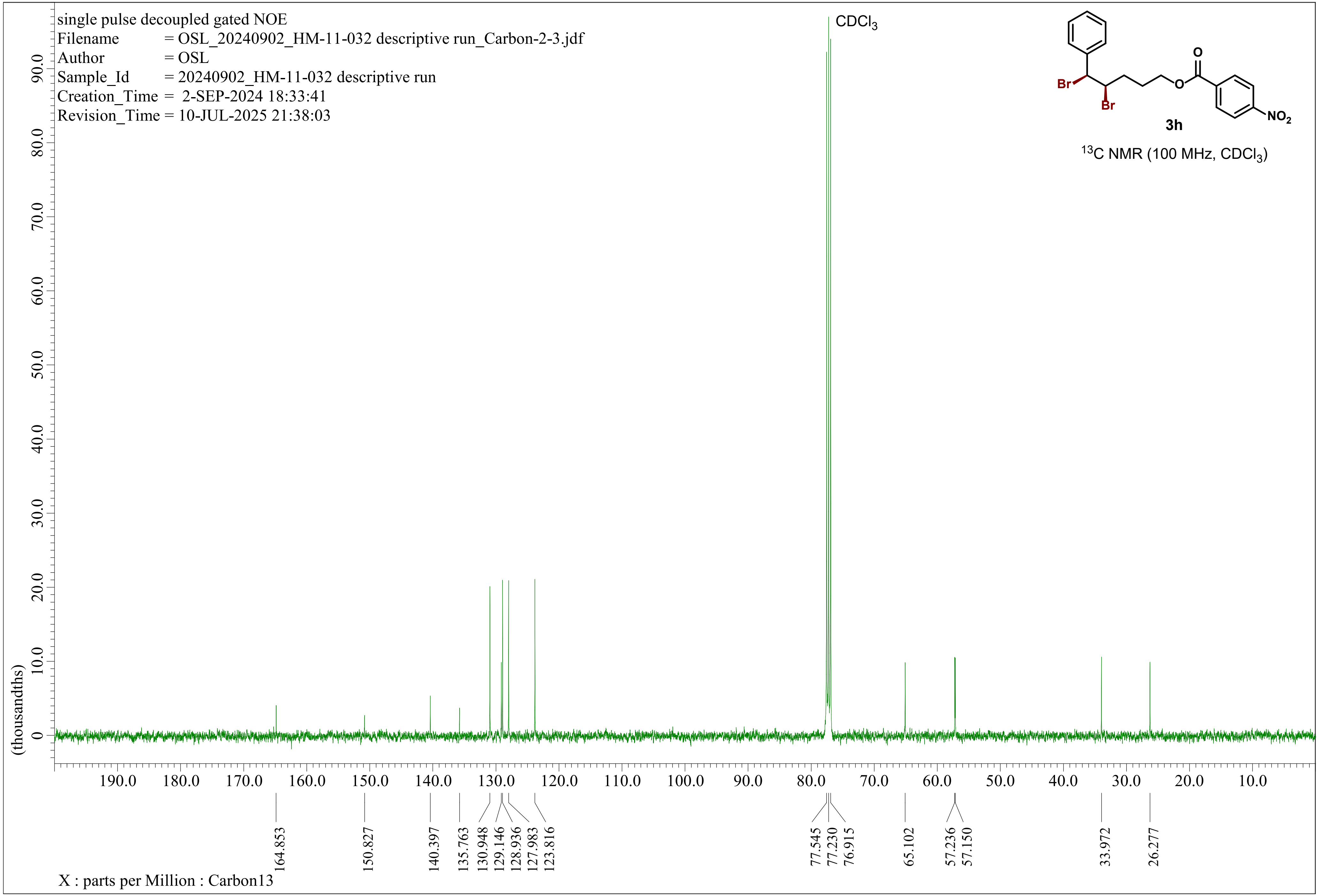
single pulse decoupled gated NOE
Filename = OSL_20240902_HM-11-032 descriptive run_Carbon-2-3.jdf
Author = OSL
Sample_Id = 20240902_HM-11-032 descriptive run
Creation_Time = 2-SEP-2024 18:33:41
Revision_Time = 10-JUL-2025 21:38:03
CDCl3
Br
Br
O
O
NO2
3h
13C NMR (100 MHz, CDCl3)
(thousandths)
164.853
150.827
140.397
135.763
130.948
129.146
128.936
127.983
123.816
77.545
77.230
76.915
65.102
57.236
57.150
33.972
26.277
X : parts per Million : Carbon13

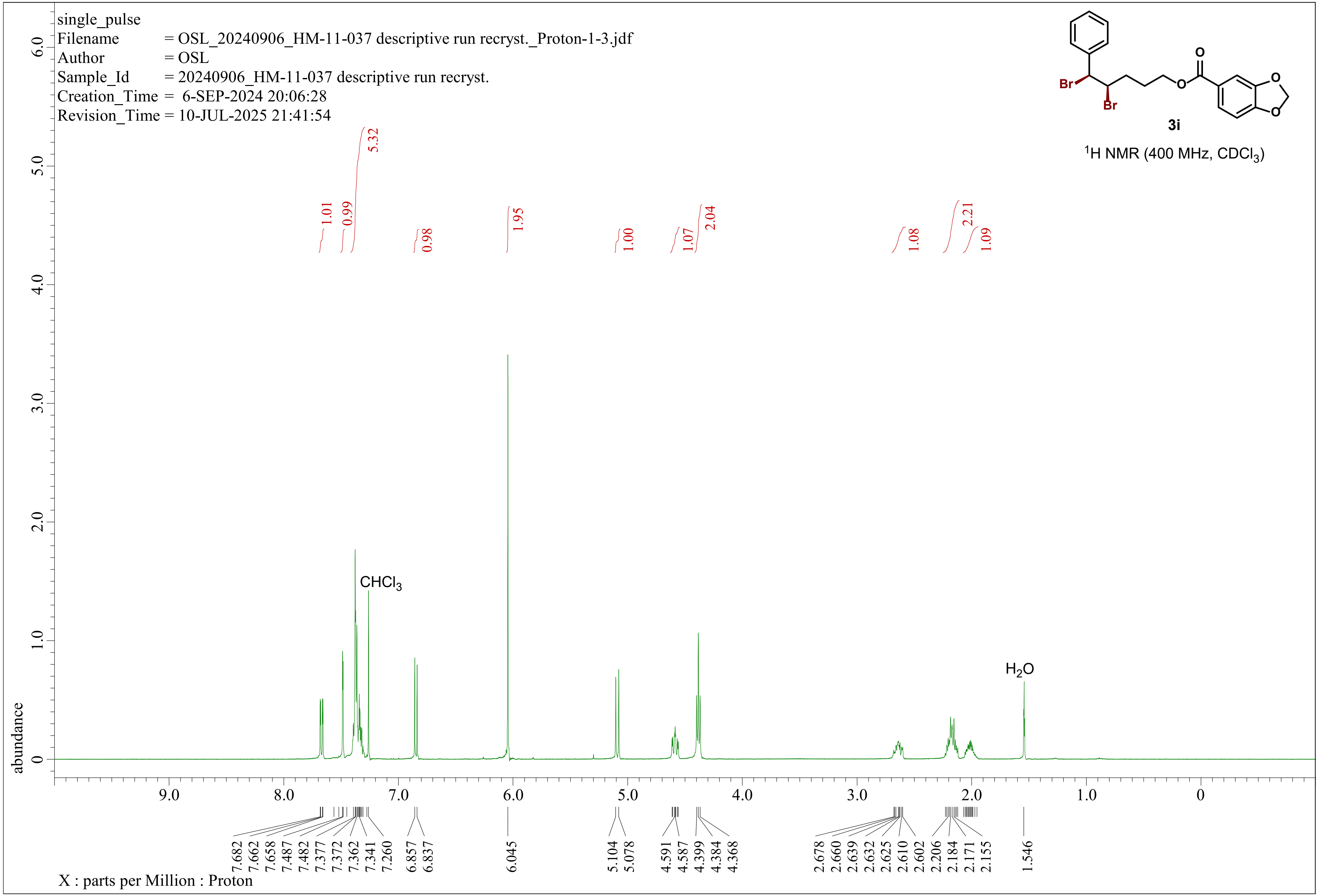
single_pulse
Filename = OSL_20240906_HM-11-037 descriptive run recryst._Proton-1-3.jdf
Author = OSL
Sample_Id = 20240906_HM-11-037 descriptive run recryst.
Creation_Time = 6-SEP-2024 20:06:28
Revision_Time = 10-JUL-2025 21:41:54
Br
Br
O
O
O
O
3i
1H NMR (400 MHz, CDCl3)
1.01
0.99
5.32
0.98
1.95
1.00
1.07
2.04
1.08
2.21
1.09
CHCl3
H2O
abundance
X : parts per Million : Proton
7.682
7.662
7.658
7.487
7.482
7.377
7.372
7.362
7.341
7.260
6.857
6.837
6.045
5.104
5.078
4.591
4.587
4.399
4.384
4.368
2.678
2.660
2.639
2.632
2.625
2.610
2.602
2.206
2.184
2.171
2.155
1.546

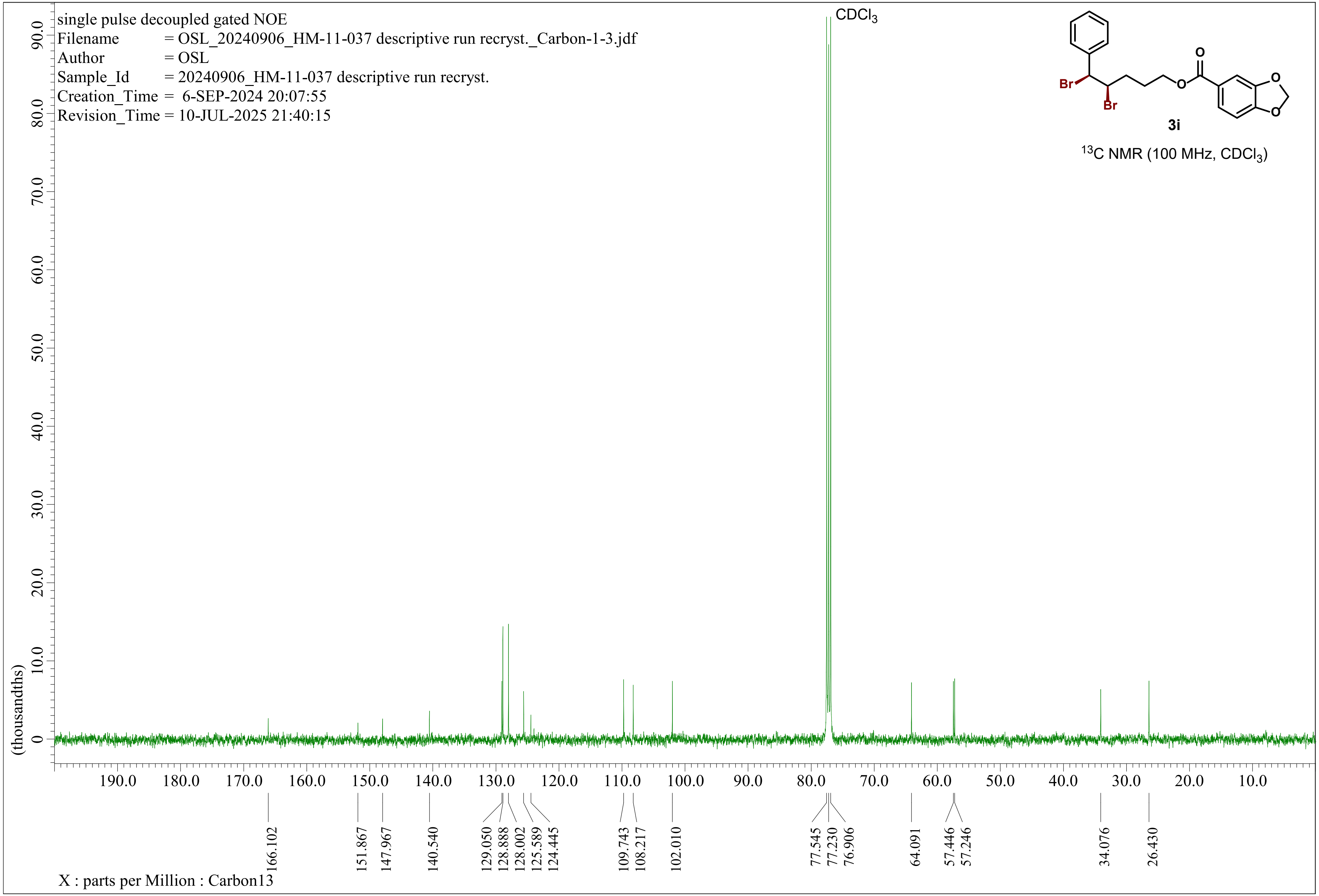
single pulse decoupled gated NOE
Filename = OSL_20240906_HM-11-037 descriptive run recryst._Carbon-1-3.jdf
Author = OSL
Sample_Id = 20240906_HM-11-037 descriptive run recryst.
Creation_Time = 6-SEP-2024 20:07:55
Revision_Time = 10-JUL-2025 21:40:15
CDCl3
Br
Br
O
O
O
O
3i
13C NMR (100 MHz, CDCl3)
(thousandths)
X : parts per Million : Carbon13
166.102
151.867
147.967
140.540
129.050
128.888
128.002
125.589
124.445
109.743
108.217
102.010
77.545
77.230
76.906
64.091
57.446
57.246
34.076
26.430

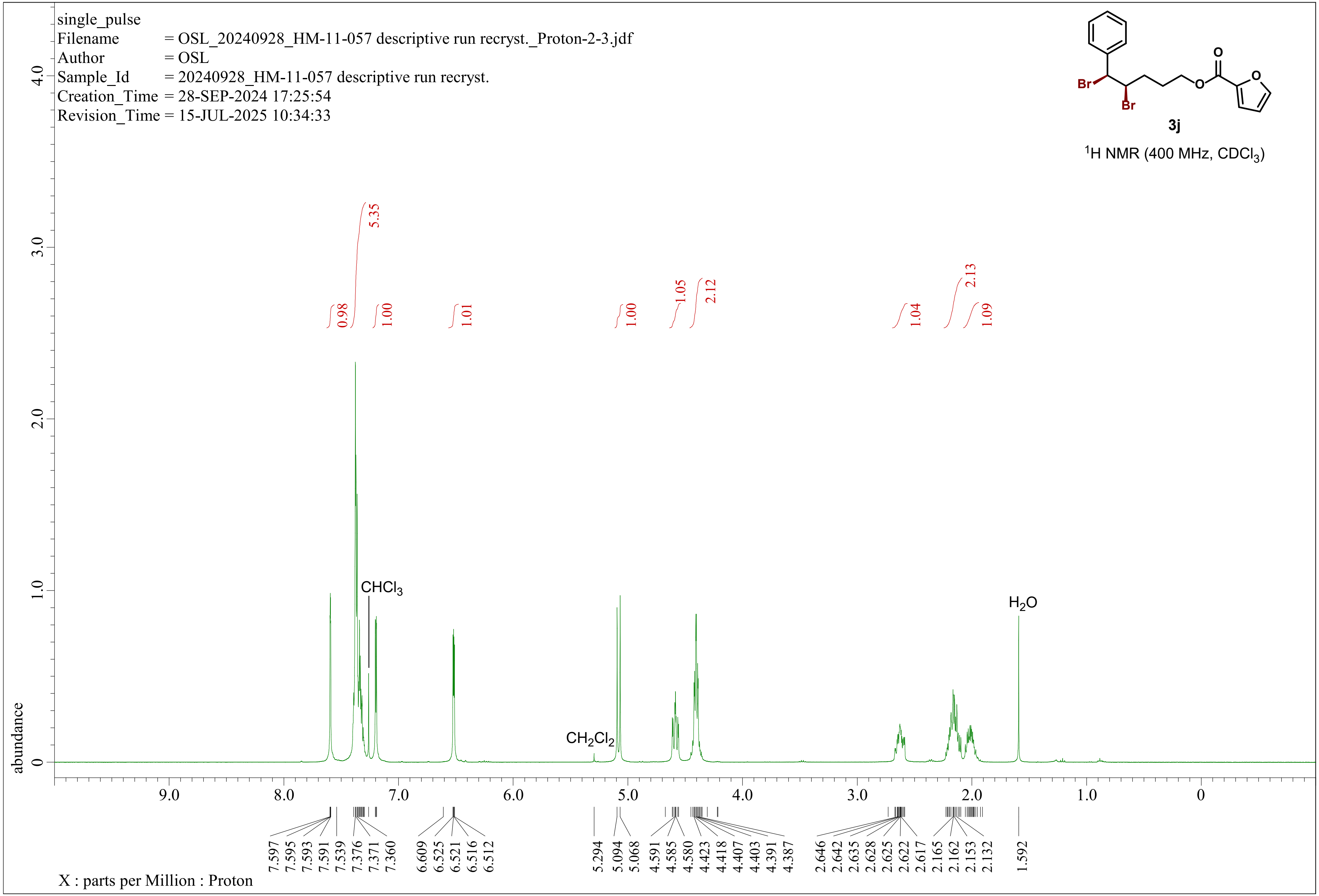
single_pulse
Filename = OSL_20240928_HM-11-057 descriptive run recryst._Proton-2-3.jdf
Author = OSL
Sample_Id = 20240928_HM-11-057 descriptive run recryst.
Creation_Time = 28-SEP-2024 17:25:54
Revision_Time = 15-JUL-2025 10:34:33
3j
1H NMR (400 MHz, CDCl3)
CHCl3
CH2Cl2
H2O
abundance
X : parts per Million : Proton
0.98
5.35
1.00
1.01
1.00
1.05
2.12
1.04
2.13
1.09
7.597 7.595 7.593 7.591 7.539 7.376 7.371 7.360
6.609 6.525 6.521 6.516 6.512
5.294 5.094 5.068
4.591 4.585 4.580 4.423 4.418 4.407 4.403 4.391 4.387
2.646 2.642 2.635 2.628 2.625 2.622 2.617 2.165 2.162 2.153 2.132
1.592

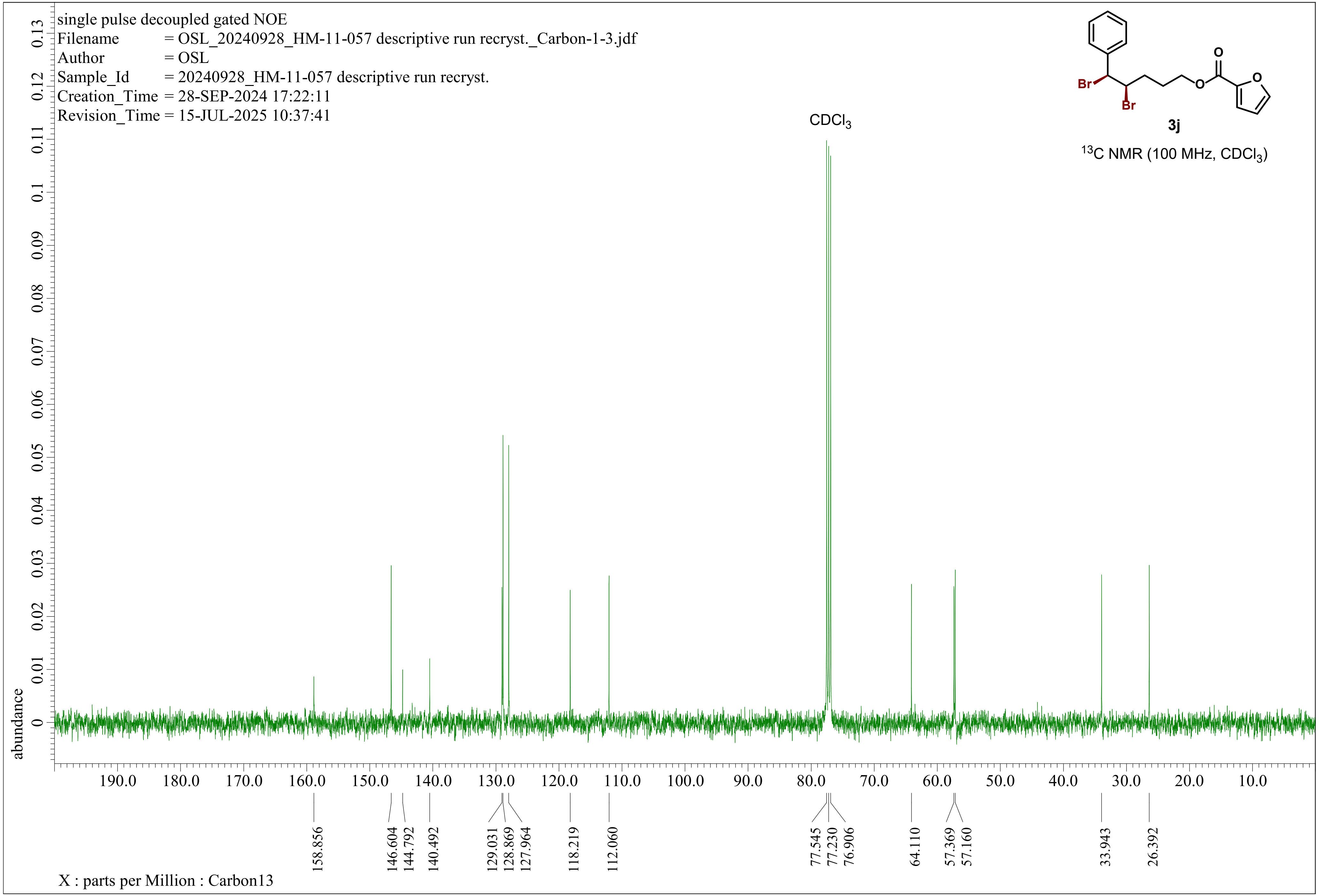
single pulse decoupled gated NOE
Filename = OSL_20240928_HM-11-057 descriptive run recryst._Carbon-1-3.jdf
Author = OSL
Sample_Id = 20240928_HM-11-057 descriptive run recryst.
Creation_Time = 28-SEP-2024 17:22:11
Revision_Time = 15-JUL-2025 10:37:41
CDCl3
3j
13C NMR (100 MHz, CDCl3)
Br
Br
O
O
O
abundance
158.856
146.604
144.792
140.492
129.031
128.869
127.964
118.219
112.060
77.545
77.230
76.906
64.110
57.369
57.160
33.943
26.392
X : parts per Million : Carbon13

single_pulse
Filename = OSL_20241004_HM-11-064 descriptive run recryst._Proton-1-2.jdf
Author = OSL
Sample_Id = 20241004_HM-11-064 descriptive run recryst.
Creation_Time = 4-OCT-2024 09:28:38
Revision_Time = 15-JUL-2025 10:44:11

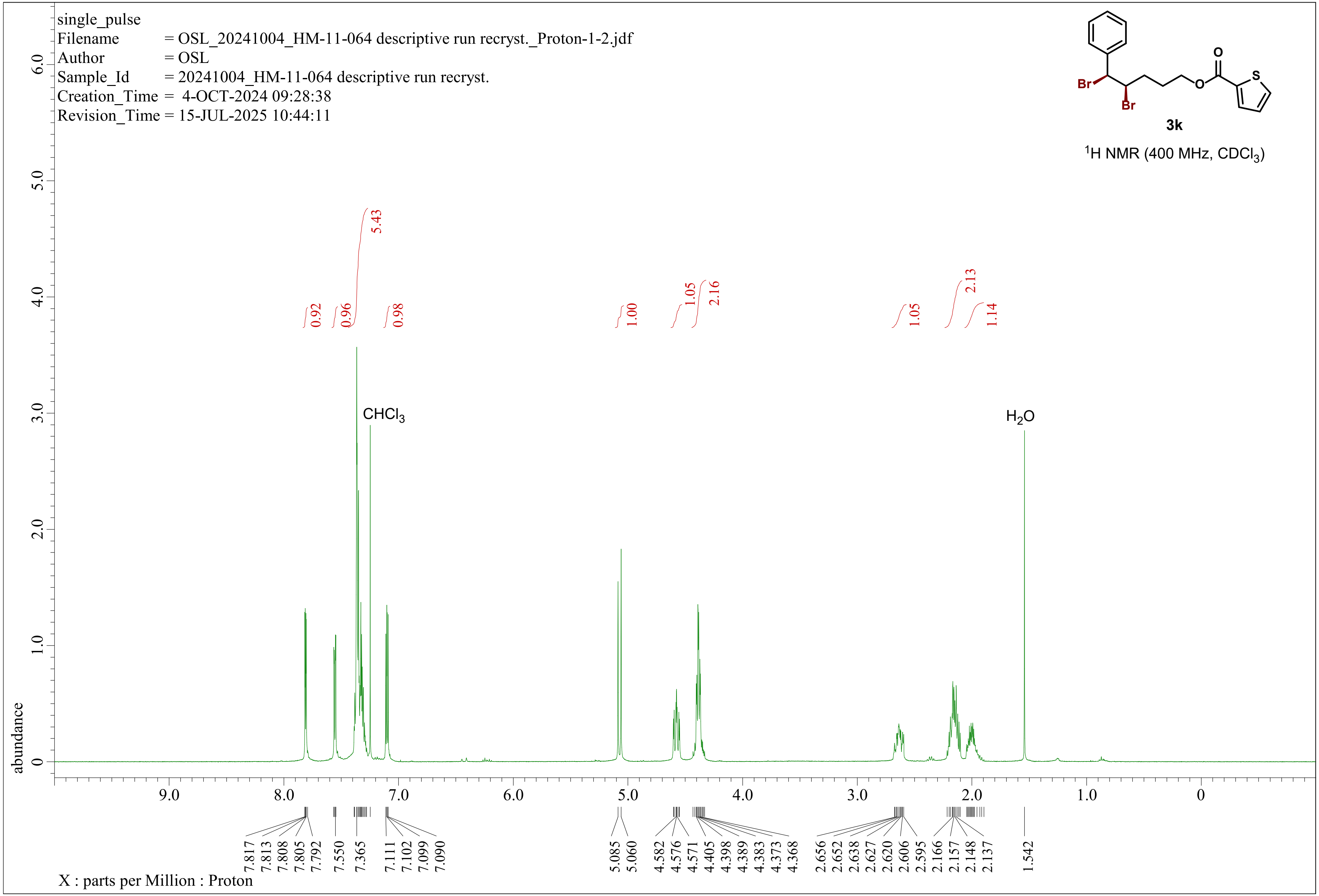

single pulse decoupled gated NOE
Filename = OSL_20241007_HM-11-064 descriptive run recryst._Carbon-1-3.jdf
Author = OSL
Sample_Id = 20241007_HM-11-064 descriptive run recryst.
Creation_Time = 7-OCT-2024 13:44:22
Revision_Time = 10-JUL-2025 21:45:42

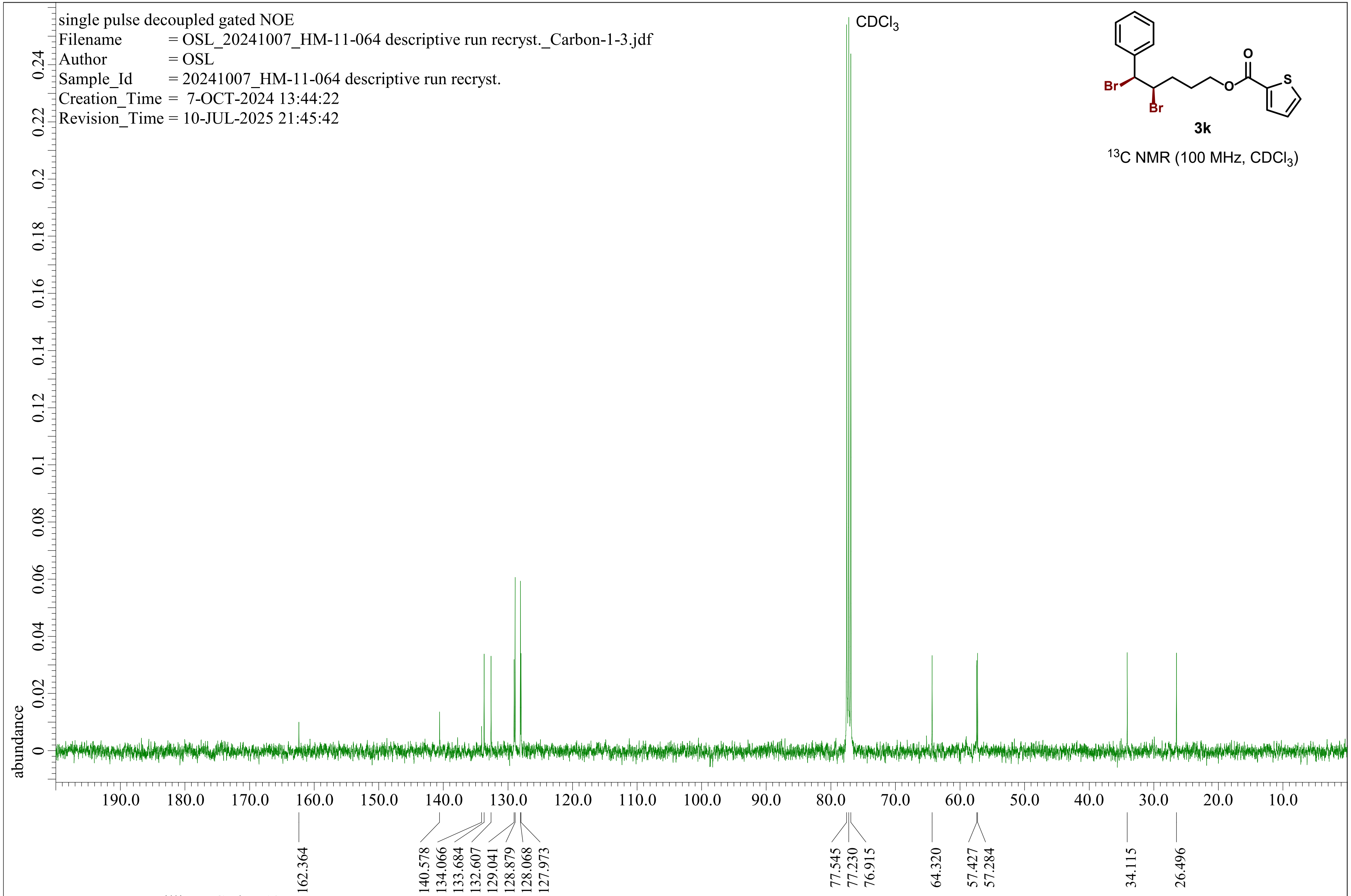

X : parts per Million : Carbon13

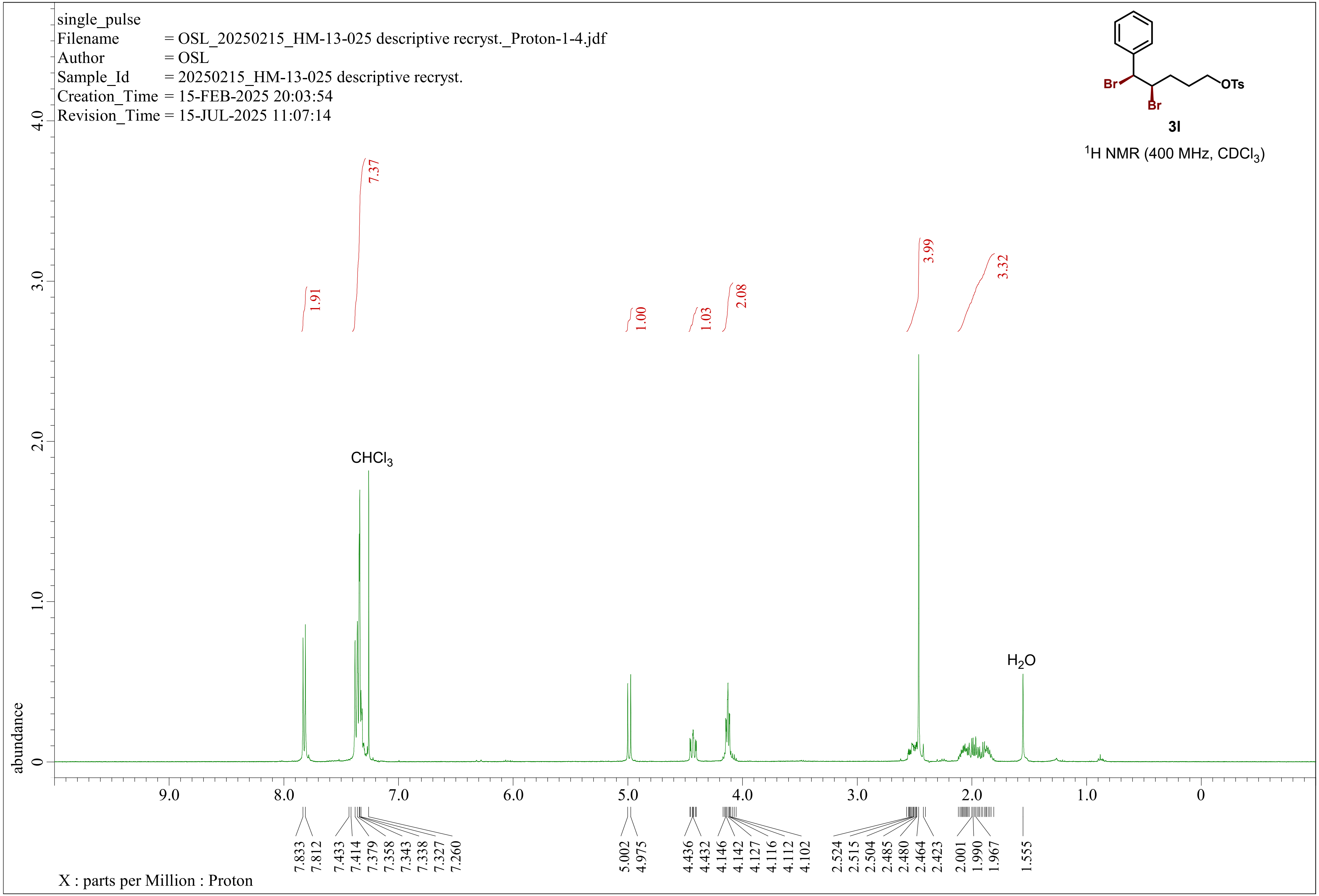

single_pulse
Filename = OSL_20250215_HM-13-025 descriptive recryst._Proton-1-4.jdf
Author = OSL
Sample_Id = 20250215_HM-13-025 descriptive recryst.
Creation_Time = 15-FEB-2025 20:03:54
Revision_Time = 15-JUL-2025 11:07:14
Br
Br
OTs
3l
1H NMR (400 MHz, CDCl3)
CHCl3
H2O
1.91
7.37
1.00
1.03
2.08
3.99
3.32
7.833
7.812
7.433
7.414
7.379
7.358
7.343
7.338
7.327
7.260
5.002
4.975
4.436
4.432
4.146
4.142
4.127
4.116
4.112
4.102
2.524
2.515
2.504
2.485
2.480
2.464
2.423
2.001
1.990
1.967
1.555
abundance
X : parts per Million : Proton

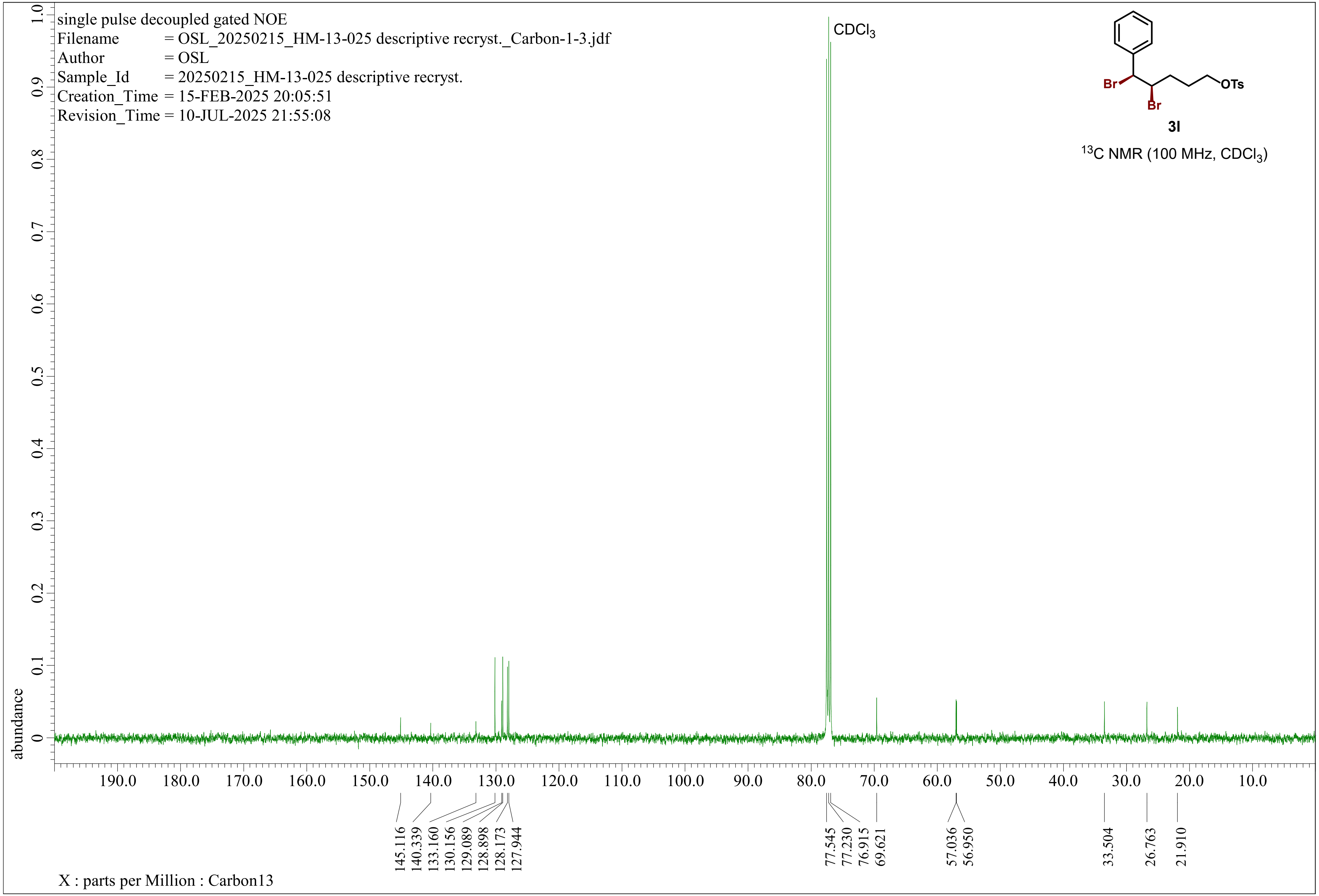
single pulse decoupled gated NOE
Filename = OSL_20250215_HM-13-025 descriptive recryst._Carbon-1-3.jdf
Author = OSL
Sample_Id = 20250215_HM-13-025 descriptive recryst.
Creation_Time = 15-FEB-2025 20:05:51
Revision_Time = 10-JUL-2025 21:55:08
CDCl3
Br
Br
OTs
3l
13C NMR (100 MHz, CDCl3)
abundance
145.116
140.339
133.160
130.156
129.089
128.898
128.173
127.944
77.545
77.230
76.915
69.621
57.036
56.950
33.504
26.763
21.910
X : parts per Million : Carbon13

single_pulse
Filename = OSL_20241014_HM-11-079 descriptive run recryst._Proton-1-4.jdf
Author = OSL
Sample_Id = 20241014_HM-11-079 descriptive run recryst.
Creation_Time = 14-OCT-2024 17:18:23
Revision_Time = 15-JUL-2025 11:11:31

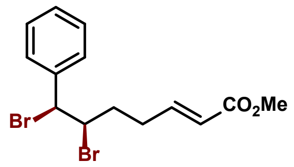

3m

^{1}H NMR (400 MHz, $CDCl_3$)

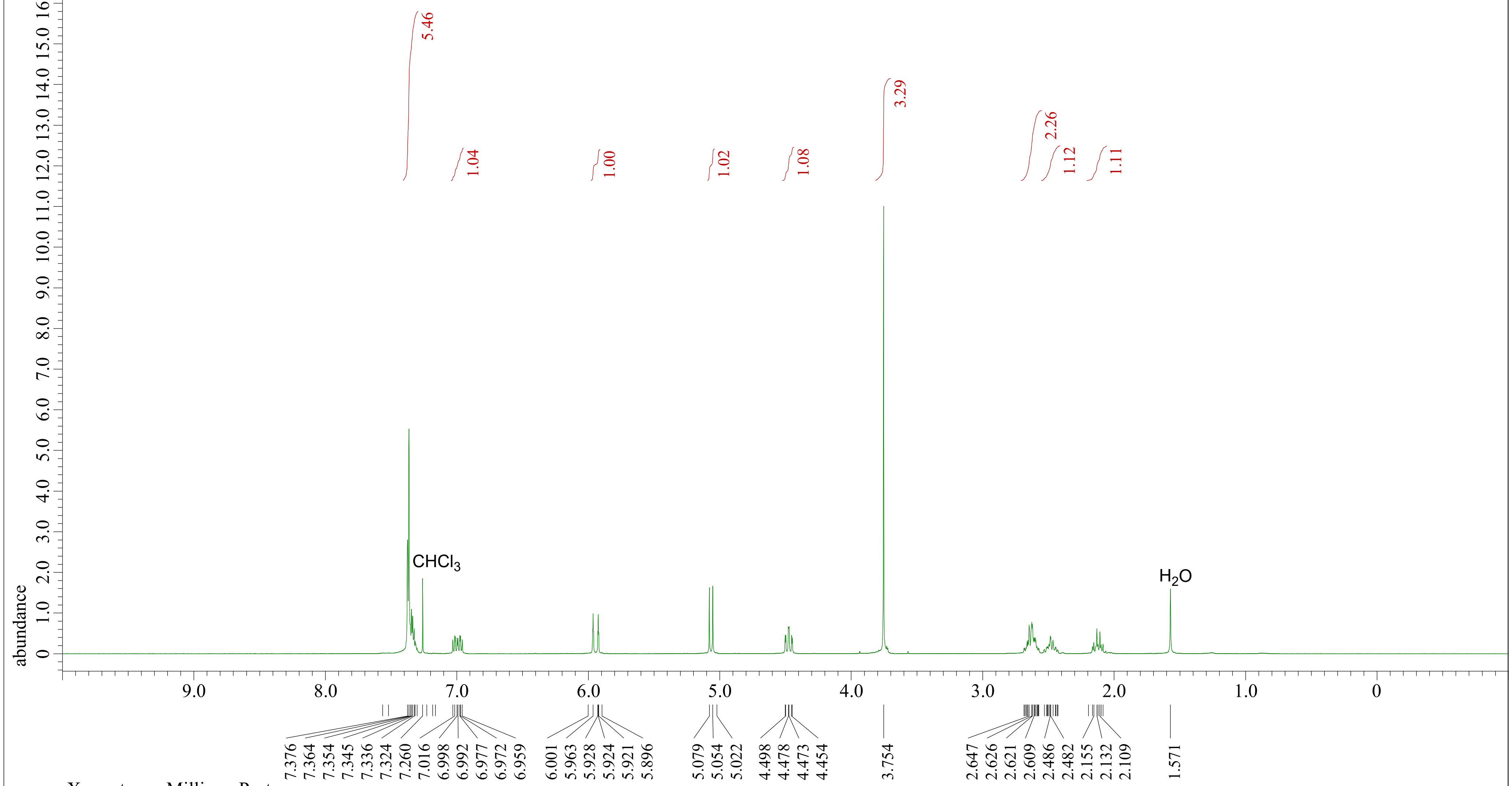

X : parts per Million : Proton

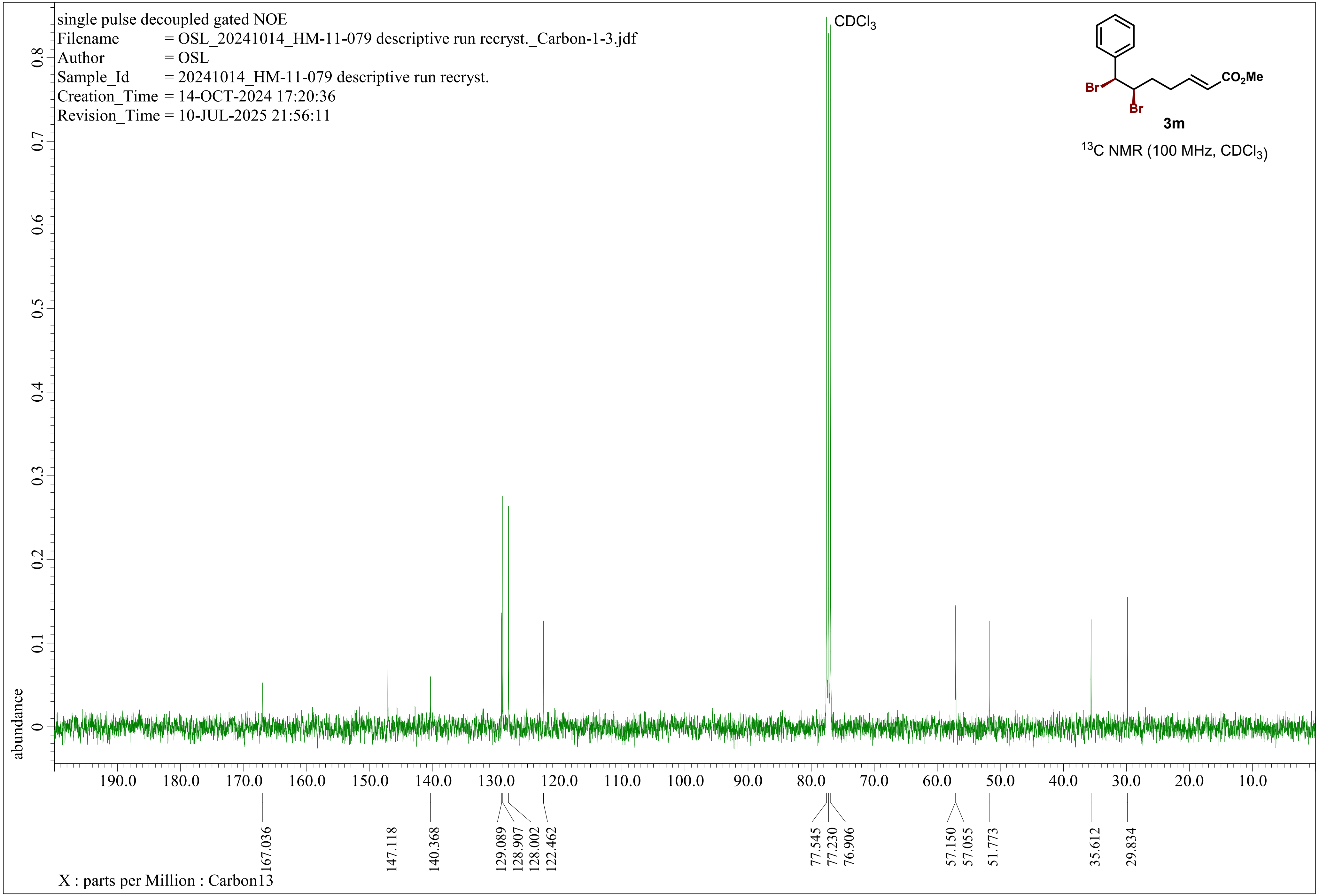
single pulse decoupled gated NOE
Filename = OSL_20241014_HM-11-079 descriptive run recryst._Carbon-1-3.jdf
Author = OSL
Sample_Id = 20241014_HM-11-079 descriptive run recryst.
Creation_Time = 14-OCT-2024 17:20:36
Revision_Time = 10-JUL-2025 21:56:11
CDCl3
Br
Br
CO2Me
3m
13C NMR (100 MHz, CDCl3)
abundance
0.8
0.7
0.6
0.5
0.4
0.3
0.2
0.1
0
190.0
180.0
170.0
160.0
150.0
140.0
130.0
120.0
110.0
100.0
90.0
80.0
70.0
60.0
50.0
40.0
30.0
20.0
10.0
167.036
147.118
140.368
129.089
128.907
128.002
122.462
77.545
77.230
76.906
57.150
57.055
51.773
35.612
29.834
X : parts per Million : Carbon13

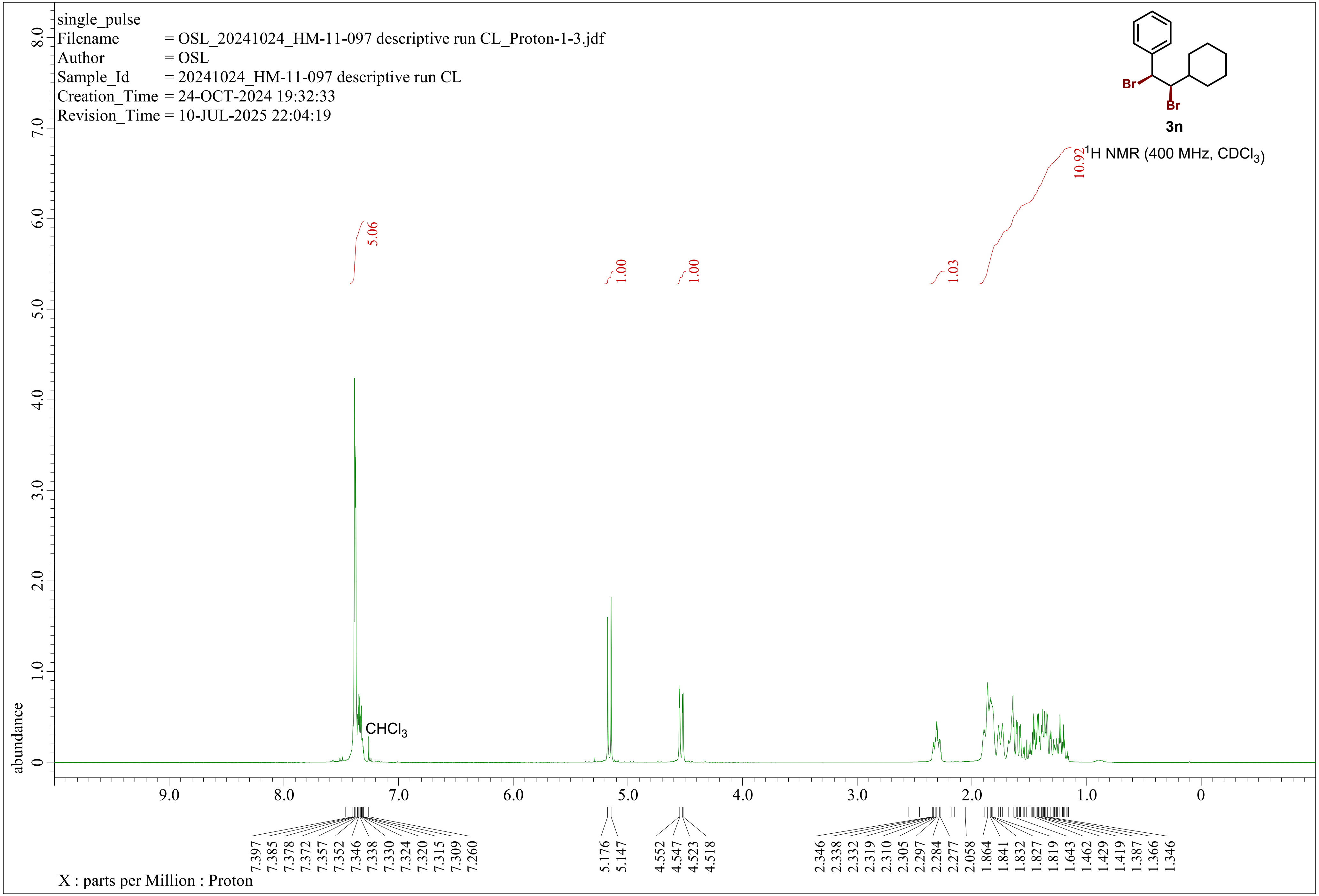

single_pulse
Filename = OSL_20241024_HM-11-097 descriptive run CL_Proton-1-3.jdf
Author = OSL
Sample_Id = 20241024_HM-11-097 descriptive run CL
Creation_Time = 24-OCT-2024 19:32:33
Revision_Time = 10-JUL-2025 22:04:19
Br
Br
3n
1H NMR (400 MHz, CDCl3)
5.06
1.00
1.00
1.03
10.92
CHCl3
abundance
X : parts per Million : Proton
7.397 7.385 7.378 7.372 7.357 7.352 7.346 7.338 7.330 7.324 7.320 7.315 7.309 7.260
5.176 5.147
4.552 4.547 4.523 4.518
2.346 2.338 2.332 2.319 2.310 2.305 2.297 2.284 2.277 2.058 1.864 1.841 1.832 1.827 1.819 1.643 1.462 1.429 1.419 1.387 1.366 1.346

single pulse decoupled gated NOE
Filename = OSL_20241025_HM-11-097 descriptive run Column, in benzene-d6_Carbon-1-4.jdf
Author = OSL
Sample_Id = 20241025_HM-11-097 descriptive run Column, in benzene-d6
Creation_Time = 25-OCT-2024 08:35:59
Revision_Time = 15-JUL-2025 11:14:53

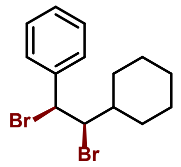

3n

^{13}C NMR (100 MHz, C_6D_6)

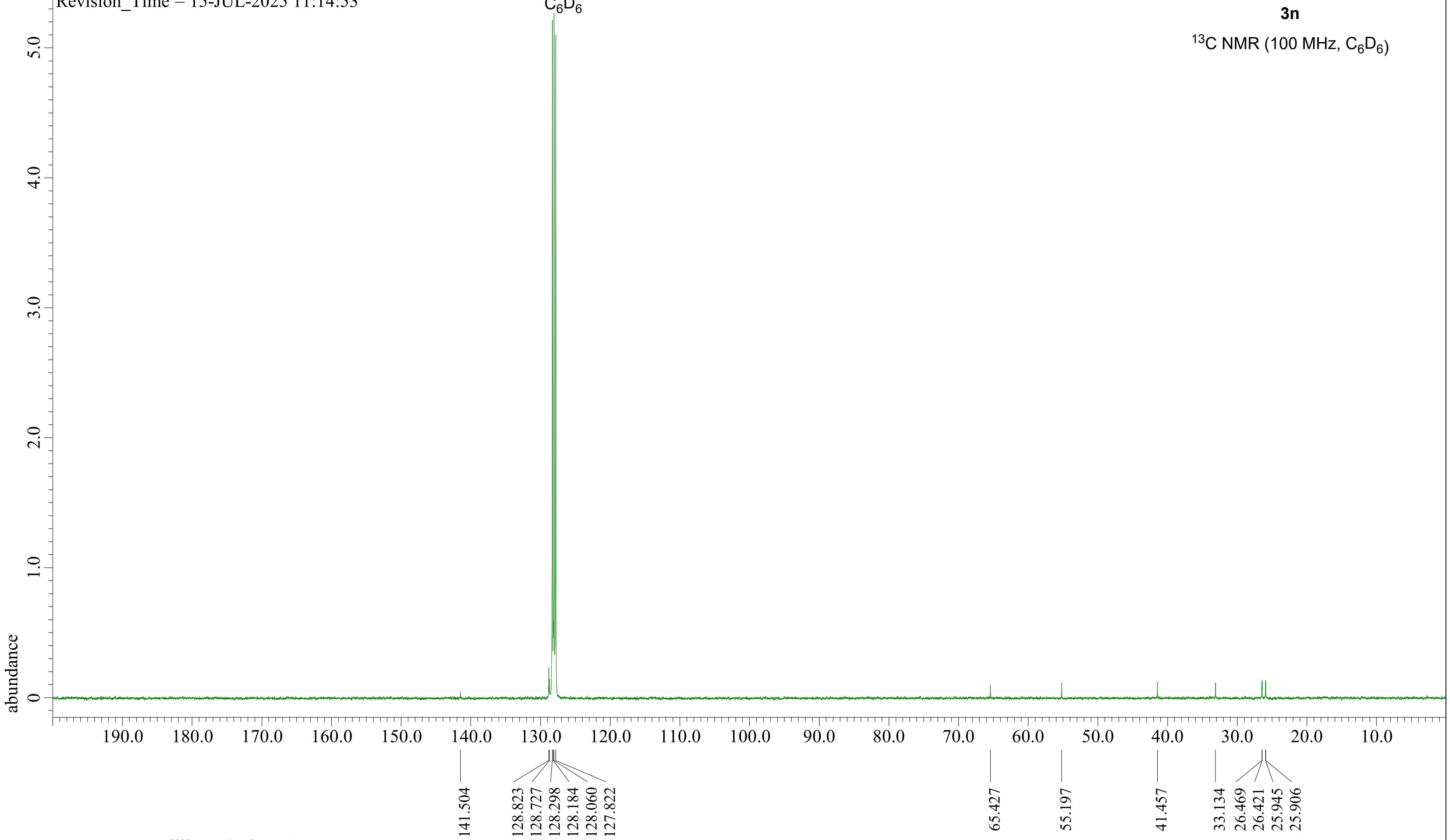

X : parts per Million : Carbon13

single_pulse
Filename = JYH-02-011 recryst._H-2.jdf
Author = OSL
Sample_Id = 20241101_JYH-02-011 descriptive run recryst.
Creation_Time = 1-NOV-2024 18:57:52
Revision_Time = 15-JUL-2025 20:58:49

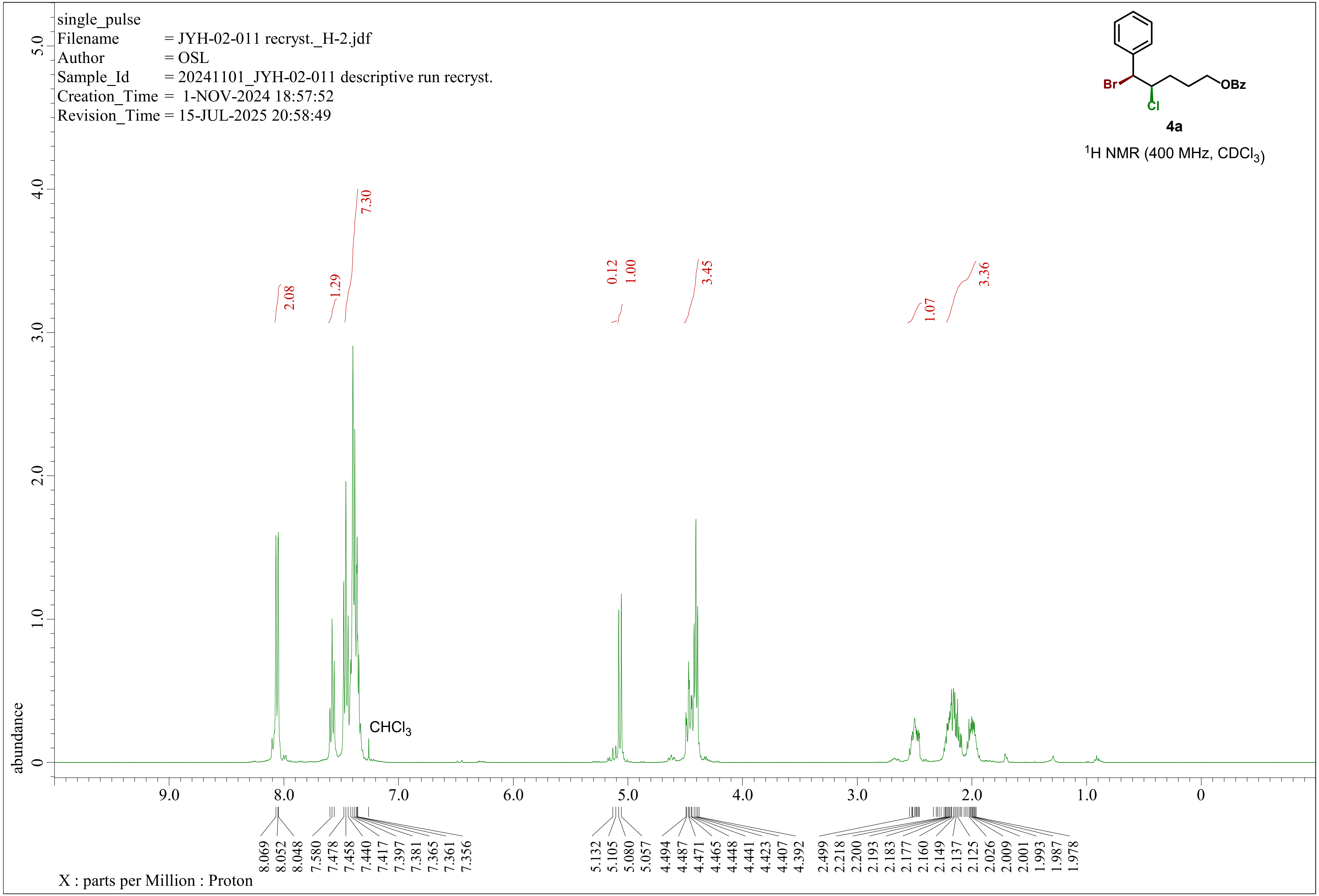

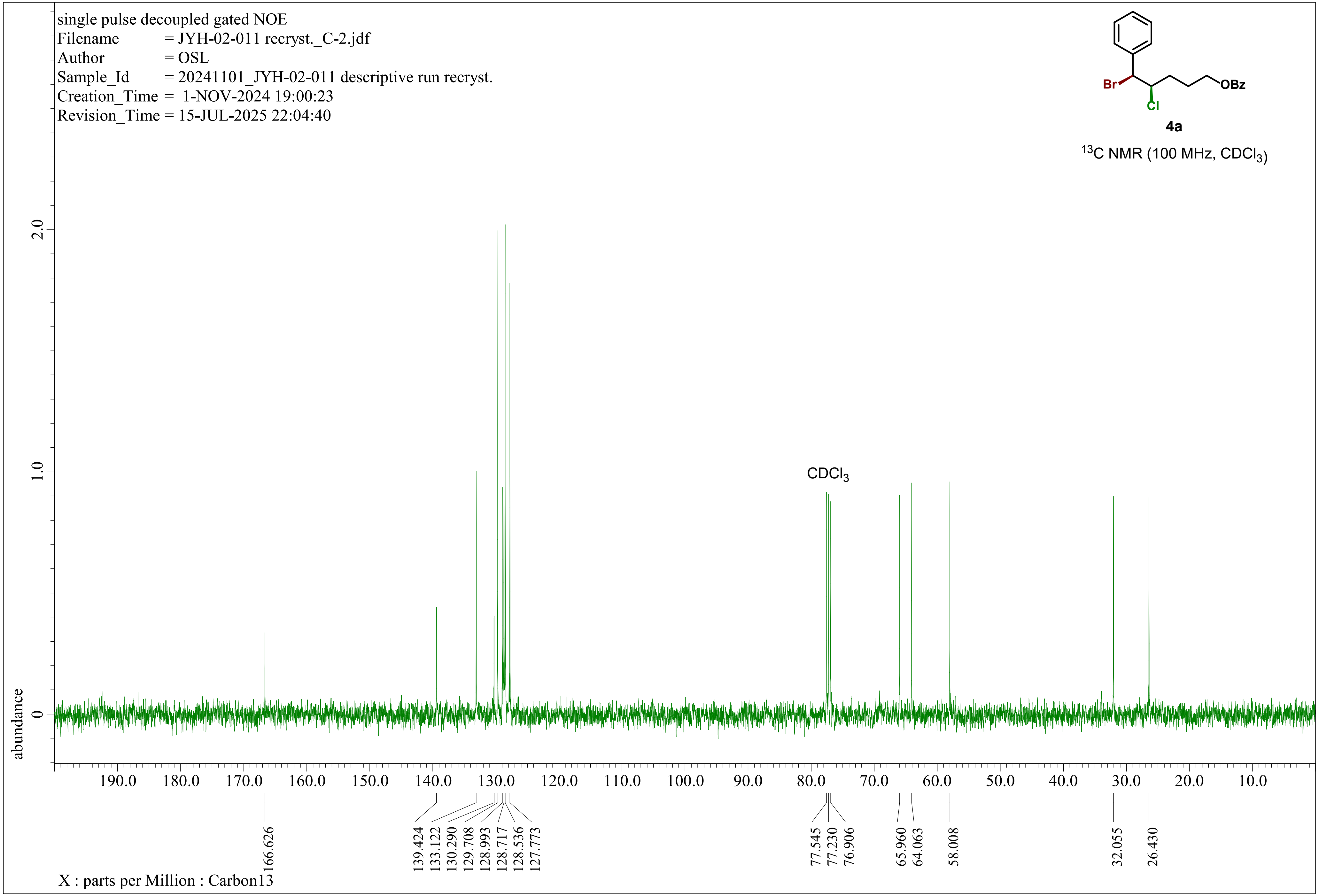

single pulse decoupled gated NOE
Filename = JYH-02-011 recryst._C-2.jdf
Author = OSL
Sample_Id = 20241101_JYH-02-011 descriptive run recryst.
Creation_Time = 1-NOV-2024 19:00:23
Revision_Time = 15-JUL-2025 22:04:40
Br
Cl
OBz
4a
13C NMR (100 MHz, CDCl3)
CDCl3
abundance
2.0
1.0
0
190.0
180.0
170.0
160.0
150.0
140.0
130.0
120.0
110.0
100.0
90.0
80.0
70.0
60.0
50.0
40.0
30.0
20.0
10.0
166.626
139.424
133.122
130.290
129.708
128.993
128.717
128.536
127.773
77.545
77.230
76.906
65.960
64.063
58.008
32.055
26.430
X : parts per Million : Carbon13

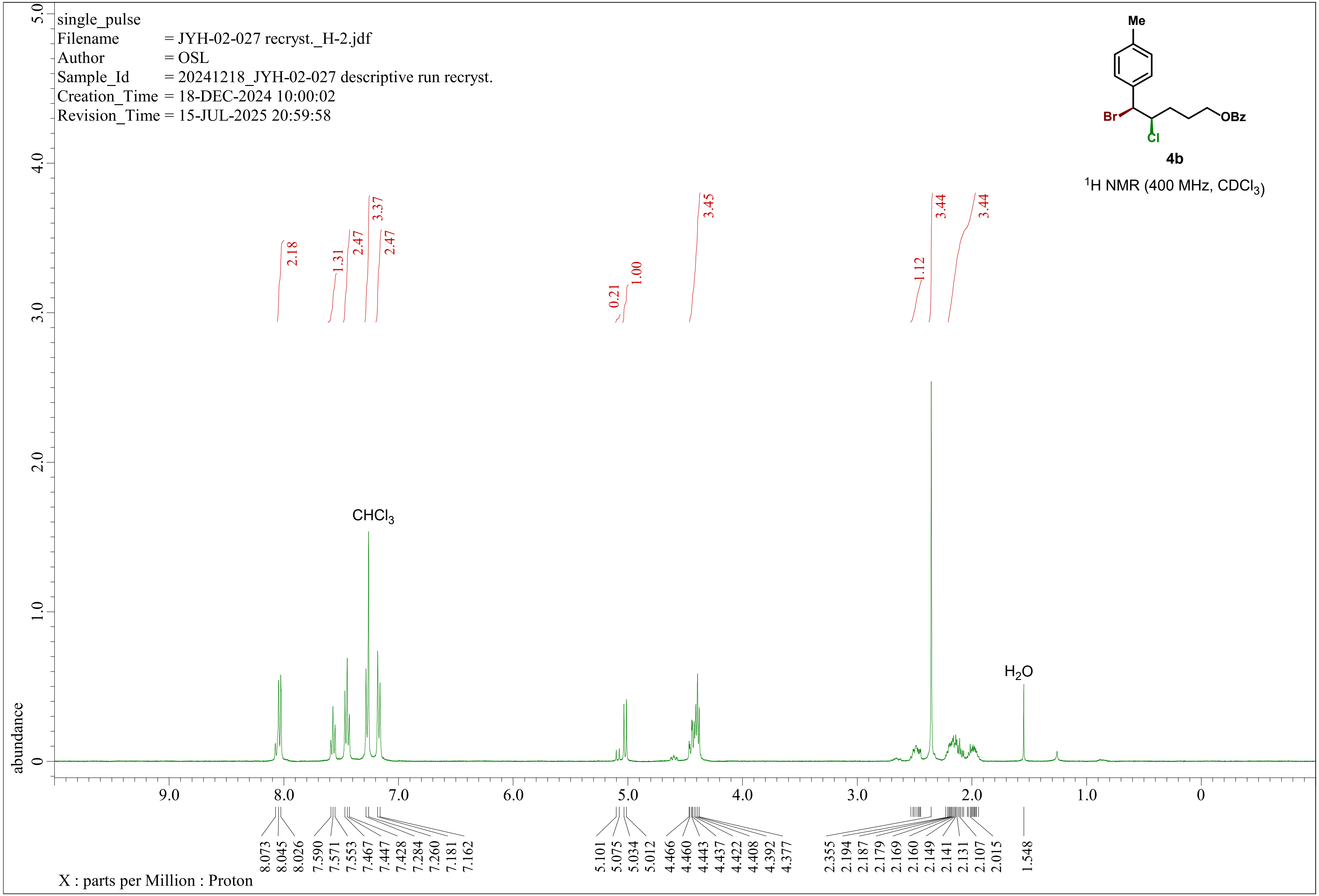
single_pulse
Filename = JYH-02-027 recryst._H-2.jdf
Author = OSL
Sample_Id = 20241218_JYH-02-027 descriptive run recryst.
Creation_Time = 18-DEC-2024 10:00:02
Revision_Time = 15-JUL-2025 20:59:58
Me
Br
Cl
OBz
4b
^{1}H NMR (400 MHz, $CDCl_3$)
2.18
1.31
2.47
3.37
2.47
0.21
1.00
3.45
1.12
3.44
3.44
$CHCl_3$
H_2O
abundance
5.0
4.0
3.0
2.0
1.0
0
9.0
8.0
7.0
6.0
5.0
4.0
3.0
2.0
1.0
0
8.073
8.045
8.026
7.590
7.571
7.553
7.467
7.447
7.428
7.284
7.260
7.181
7.162
5.101
5.075
5.034
5.012
4.466
4.460
4.443
4.437
4.422
4.408
4.392
4.377
2.355
2.194
2.187
2.179
2.169
2.160
2.149
2.141
2.131
2.107
2.015
1.548
X : parts per Million : Proton

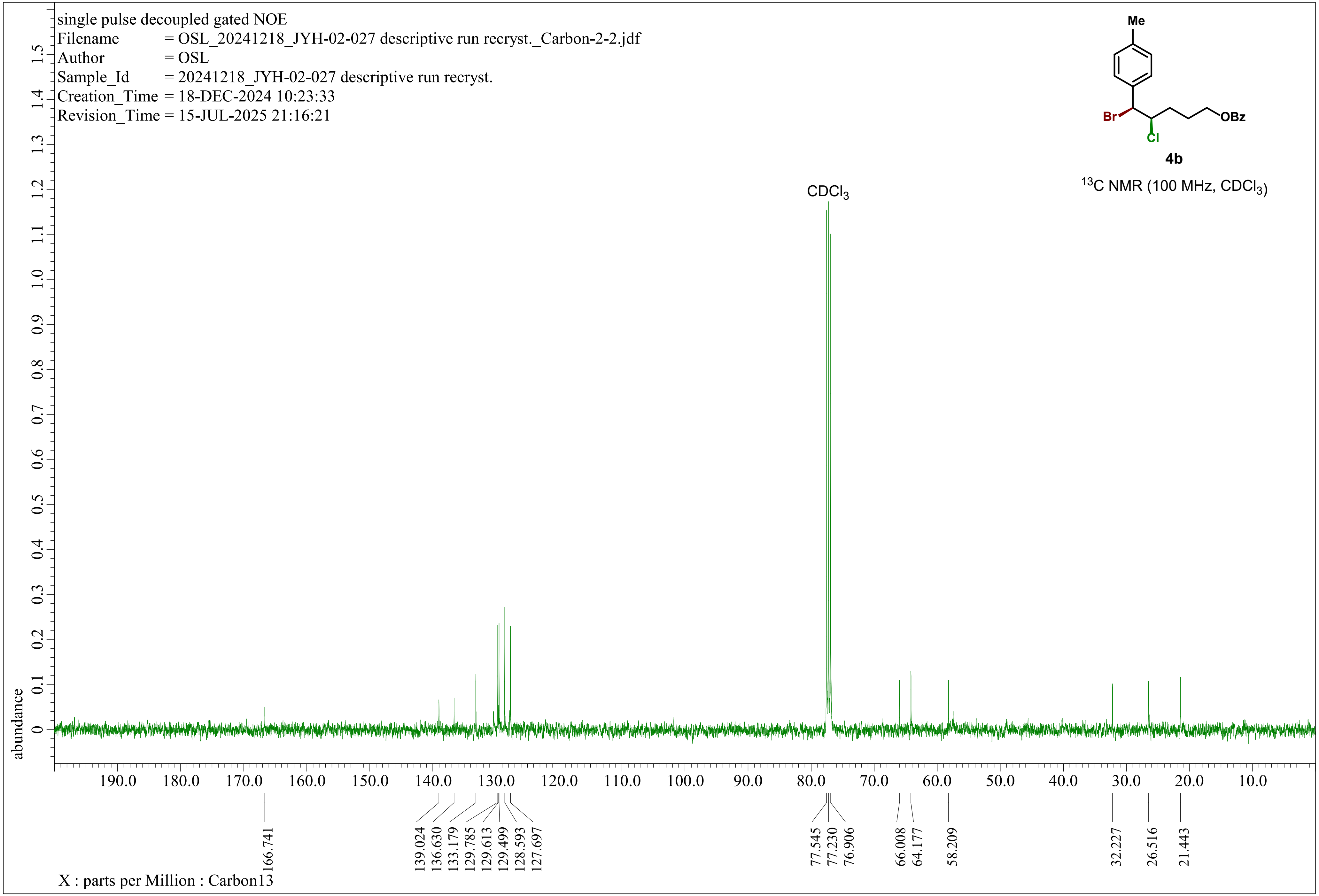

single pulse decoupled gated NOE
Filename = OSL_20241218_JYH-02-027 descriptive run recryst._Carbon-2-2.jdf
Author = OSL
Sample_Id = 20241218_JYH-02-027 descriptive run recryst.
Creation_Time = 18-DEC-2024 10:23:33
Revision_Time = 15-JUL-2025 21:16:21
Me
Br
Cl
OBz
4b
13C NMR (100 MHz, CDCl3)
CDCl3
abundance
166.741
139.024
136.630
133.179
129.785
129.613
129.499
128.593
127.697
77.545
77.230
76.906
66.008
64.177
58.209
32.227
26.516
21.443
X : parts per Million : Carbon13

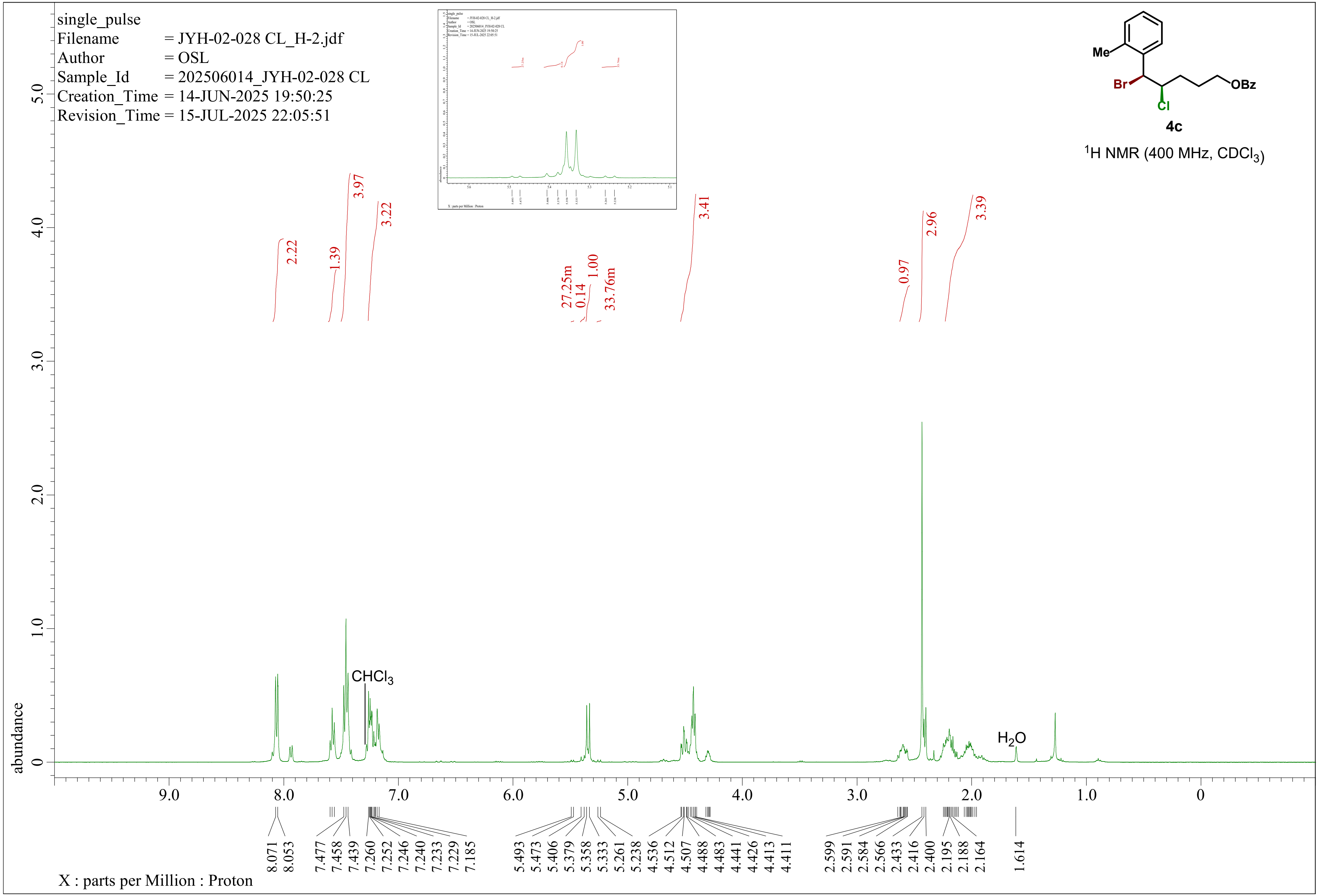
single_pulse
Filename = JYH-02-028 CL_H-2.jdf
Author = OSL
Sample_Id = 202506014_JYH-02-028 CL
Creation_Time = 14-JUN-2025 19:50:25
Revision_Time = 15-JUL-2025 22:05:51
Me
Br
Cl
OBz
4c
^{1}H NMR (400 MHz, CDCl$_3$)
2.22
1.39
3.97
3.22
27.25m
0.14
1.00
33.76m
3.41
0.97
2.96
3.39
CHCl$_3$
H$_2$O
abundance
X : parts per Million : Proton
8.071
8.053
7.477
7.458
7.439
7.260
7.252
7.246
7.240
7.233
7.229
7.185
5.493
5.473
5.406
5.379
5.358
5.333
5.261
5.238
4.536
4.512
4.507
4.488
4.483
4.441
4.426
4.413
4.411
2.599
2.591
2.584
2.566
2.433
2.416
2.400
2.195
2.188
2.164
1.614

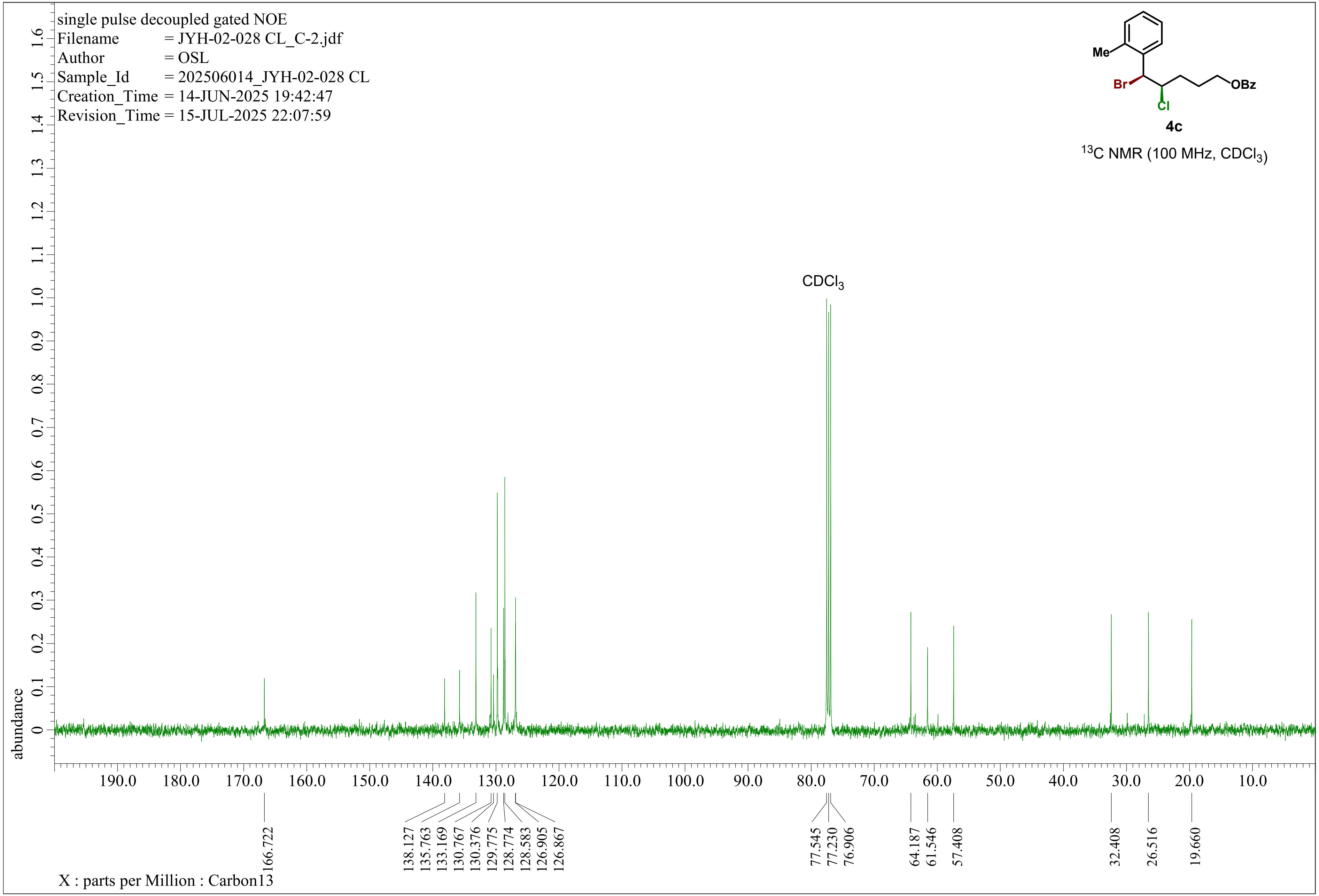
single pulse decoupled gated NOE
Filename = JYH-02-028 CL_C-2.jdf
Author = OSL
Sample_Id = 202506014_JYH-02-028 CL
Creation_Time = 14-JUN-2025 19:42:47
Revision_Time = 15-JUL-2025 22:07:59
Me
Br
Cl
OBz
4c
13C NMR (100 MHz, CDCl3)
CDCl3
abundance
X : parts per Million : Carbon13
166.722
138.127
135.763
133.169
130.767
130.376
129.775
128.774
128.583
126.905
126.867
77.545
77.230
76.906
64.187
61.546
57.408
32.408
26.516
19.660

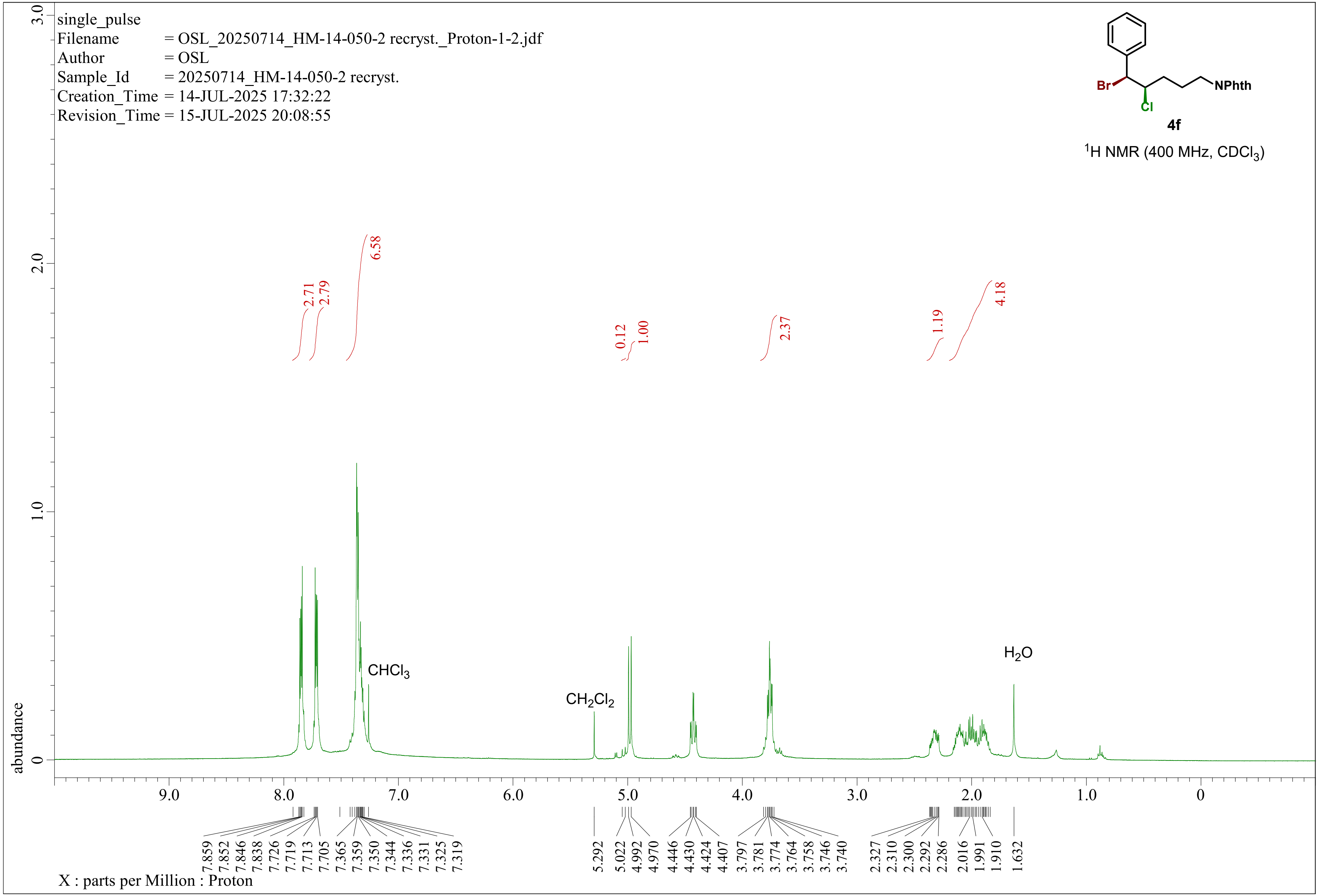
single_pulse
Filename = OSL_20250714_HM-14-050-2 recryst._Proton-1-2.jdf
Author = OSL
Sample_Id = 20250714_HM-14-050-2 recryst.
Creation_Time = 14-JUL-2025 17:32:22
Revision_Time = 15-JUL-2025 20:08:55
Br
Cl
NPhth
4f
1H NMR (400 MHz, CDCl3)
2.71
2.79
6.58
0.12
1.00
2.37
1.19
4.18
CHCl3
CH2Cl2
H2O
abundance
7.859 7.852 7.846 7.838 7.726 7.719 7.713 7.705 7.365 7.359 7.350 7.344 7.336 7.331 7.325 7.319
5.292 5.022 4.992 4.970 4.446 4.430 4.424 4.407 3.797 3.781 3.774 3.764 3.758 3.746 3.740
2.327 2.310 2.300 2.292 2.286 2.016 1.991 1.910 1.632
X : parts per Million : Proton

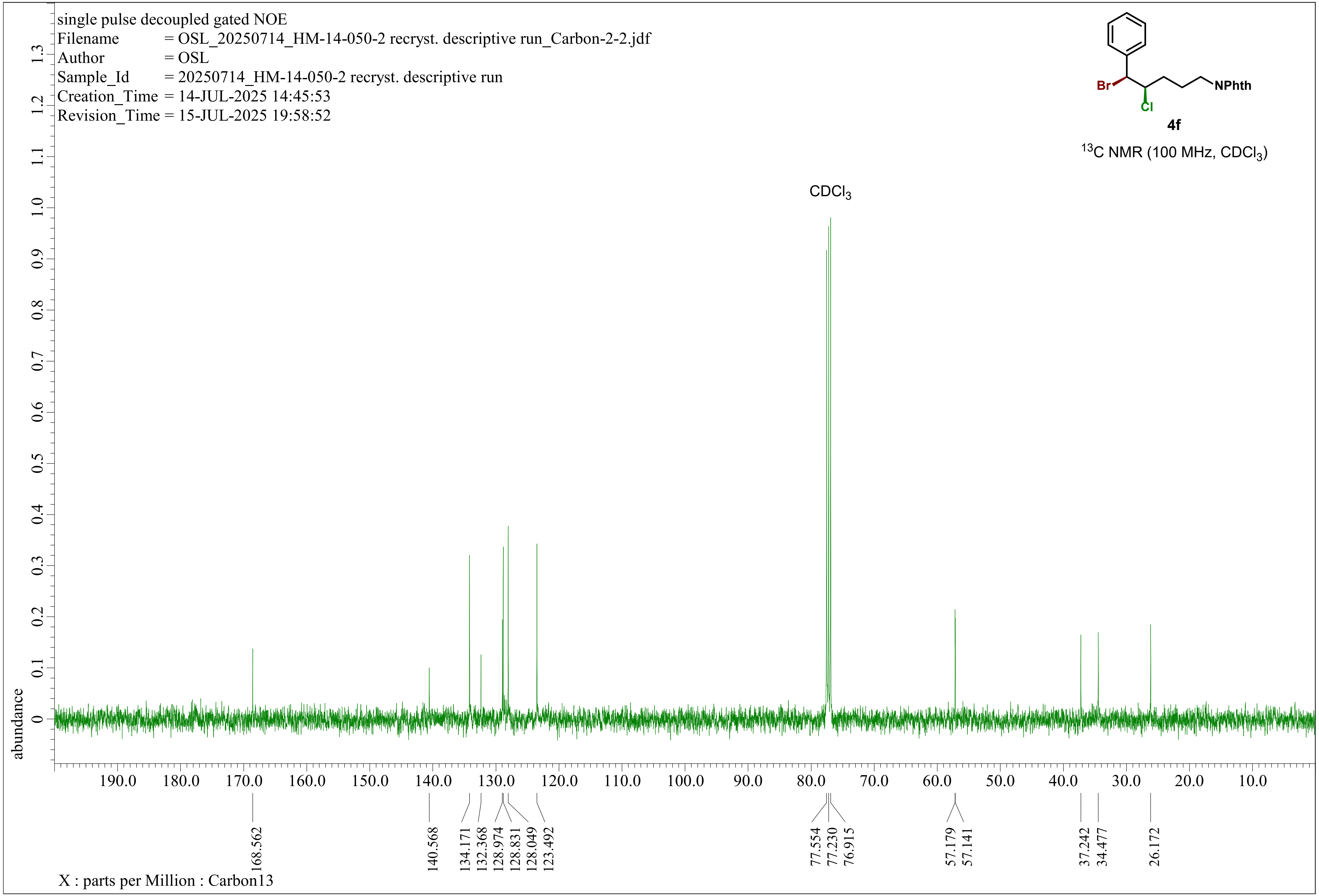

single pulse decoupled gated NOE
Filename = OSL_20250714_HM-14-050-2 recryst. descriptive run_Carbon-2-2.jdf
Author = OSL
Sample_Id = 20250714_HM-14-050-2 recryst. descriptive run
Creation_Time = 14-JUL-2025 14:45:53
Revision_Time = 15-JUL-2025 19:58:52
Br
Cl
NPhth
4f
^{13}C NMR (100 MHz, CDCl$_3$)
CDCl$_3$
abundance
168.562
140.568
134.171
132.368
128.974
128.831
128.049
123.492
77.554
77.230
76.915
57.179
57.141
37.242
34.477
26.172
X : parts per Million : Carbon13

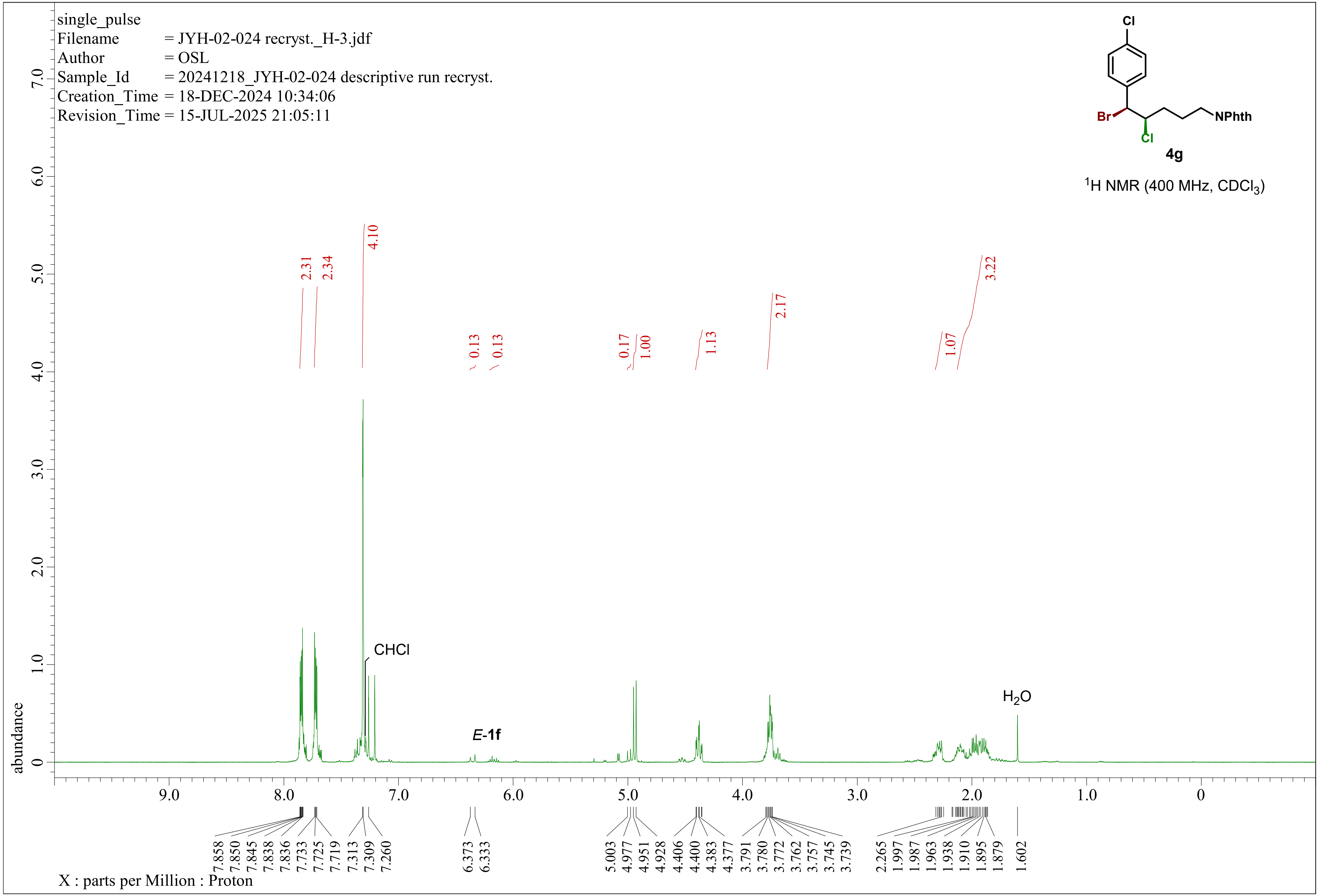
single_pulse
Filename = JYH-02-024 recryst._H-3.jdf
Author = OSL
Sample_Id = 20241218_JYH-02-024 descriptive run recryst.
Creation_Time = 18-DEC-2024 10:34:06
Revision_Time = 15-JUL-2025 21:05:11
Cl
Br
Cl
NPhth
4g
1H NMR (400 MHz, CDCl3)
2.31
2.34
4.10
0.13
0.13
0.17
1.00
1.13
2.17
1.07
3.22
CHCl
E-1f
H2O
abundance
X : parts per Million : Proton
7.858
7.850
7.845
7.838
7.836
7.733
7.725
7.719
7.313
7.309
7.260
6.373
6.333
5.003
4.977
4.951
4.928
4.406
4.400
4.383
4.377
3.791
3.780
3.772
3.762
3.757
3.745
3.739
2.265
1.997
1.987
1.963
1.938
1.910
1.895
1.879
1.602

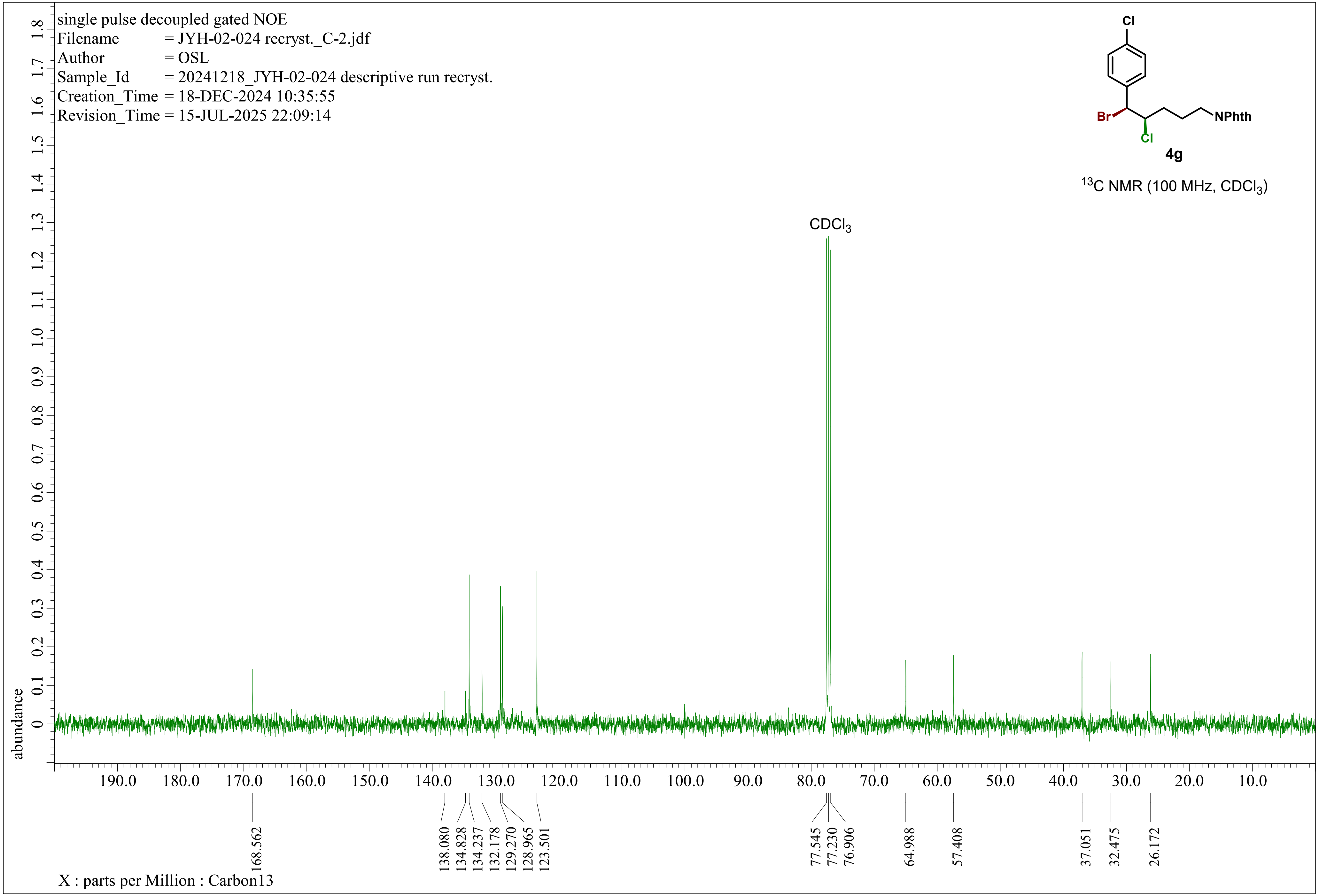
single pulse decoupled gated NOE
Filename = JYH-02-024 recryst._C-2.jdf
Author = OSL
Sample_Id = 20241218_JYH-02-024 descriptive run recryst.
Creation_Time = 18-DEC-2024 10:35:55
Revision_Time = 15-JUL-2025 22:09:14
Cl
Br
Cl
NPhth
4g
13C NMR (100 MHz, CDCl3)
CDCl3
abundance
168.562
138.080
134.828
134.237
132.178
129.270
128.965
123.501
77.545
77.230
76.906
64.988
57.408
37.051
32.475
26.172
X : parts per Million : Carbon13

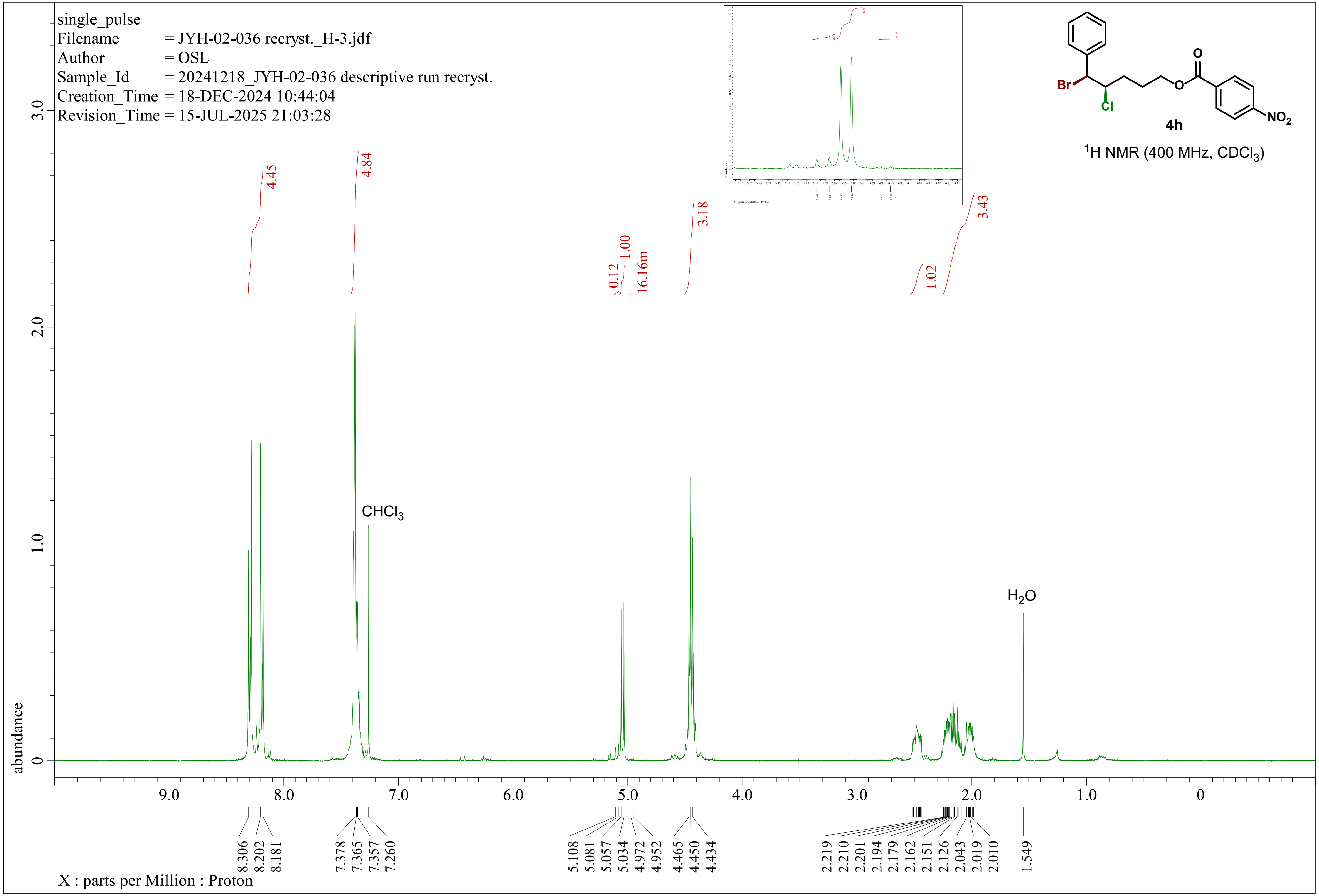
single_pulse
Filename = JYH-02-036 recryst._H-3.jdf
Author = OSL
Sample_Id = 20241218_JYH-02-036 descriptive run recryst.
Creation_Time = 18-DEC-2024 10:44:04
Revision_Time = 15-JUL-2025 21:03:28
4h
^{1}H NMR (400 MHz, $CDCl_3$)
4.45
4.84
0.12
1.00
16.16m
3.18
1.02
3.43
$CHCl_3$
H_2O
abundance
X : parts per Million : Proton
8.306
8.202
8.181
7.378
7.365
7.357
7.260
5.108
5.081
5.057
5.034
4.972
4.952
4.465
4.450
4.434
2.219
2.210
2.201
2.194
2.179
2.162
2.151
2.126
2.043
2.019
2.010
1.549

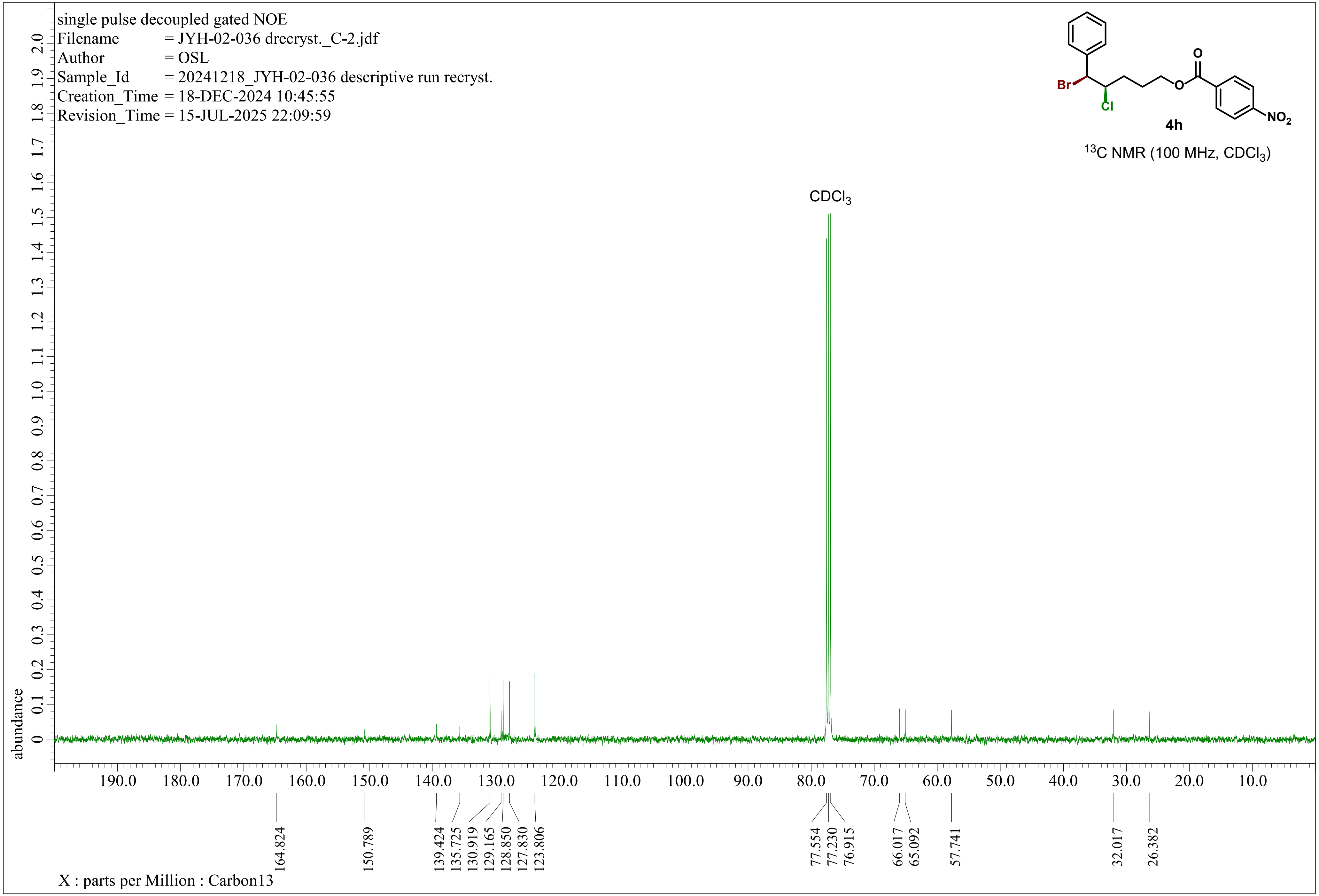
single pulse decoupled gated NOE
Filename = JYH-02-036 drecryst._C-2.jdf
Author = OSL
Sample_Id = 20241218_JYH-02-036 descriptive run recryst.
Creation_Time = 18-DEC-2024 10:45:55
Revision_Time = 15-JUL-2025 22:09:59
Br
Cl
O
O
NO2
4h
13C NMR (100 MHz, CDCl3)
CDCl3
abundance
164.824
150.789
139.424
135.725
130.919
129.165
128.850
127.830
123.806
77.554
77.230
76.915
66.017
65.092
57.741
32.017
26.382
X : parts per Million : Carbon13

single_pulse
Filename = OSL_20241116_HM-12-029 descriptive run Column_Proton-1-4.jdf
Author = OSL
Sample_Id = 20241116_HM-12-028 descriptive run Column
Creation_Time = 16-NOV-2024 18:57:55
Revision_Time = 15-JUL-2025 19:40:37

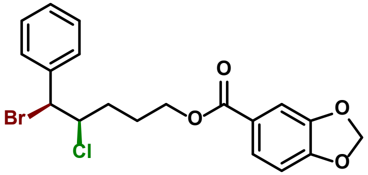

4i

^{1}H NMR (400 MHz, $CDCl_3$)

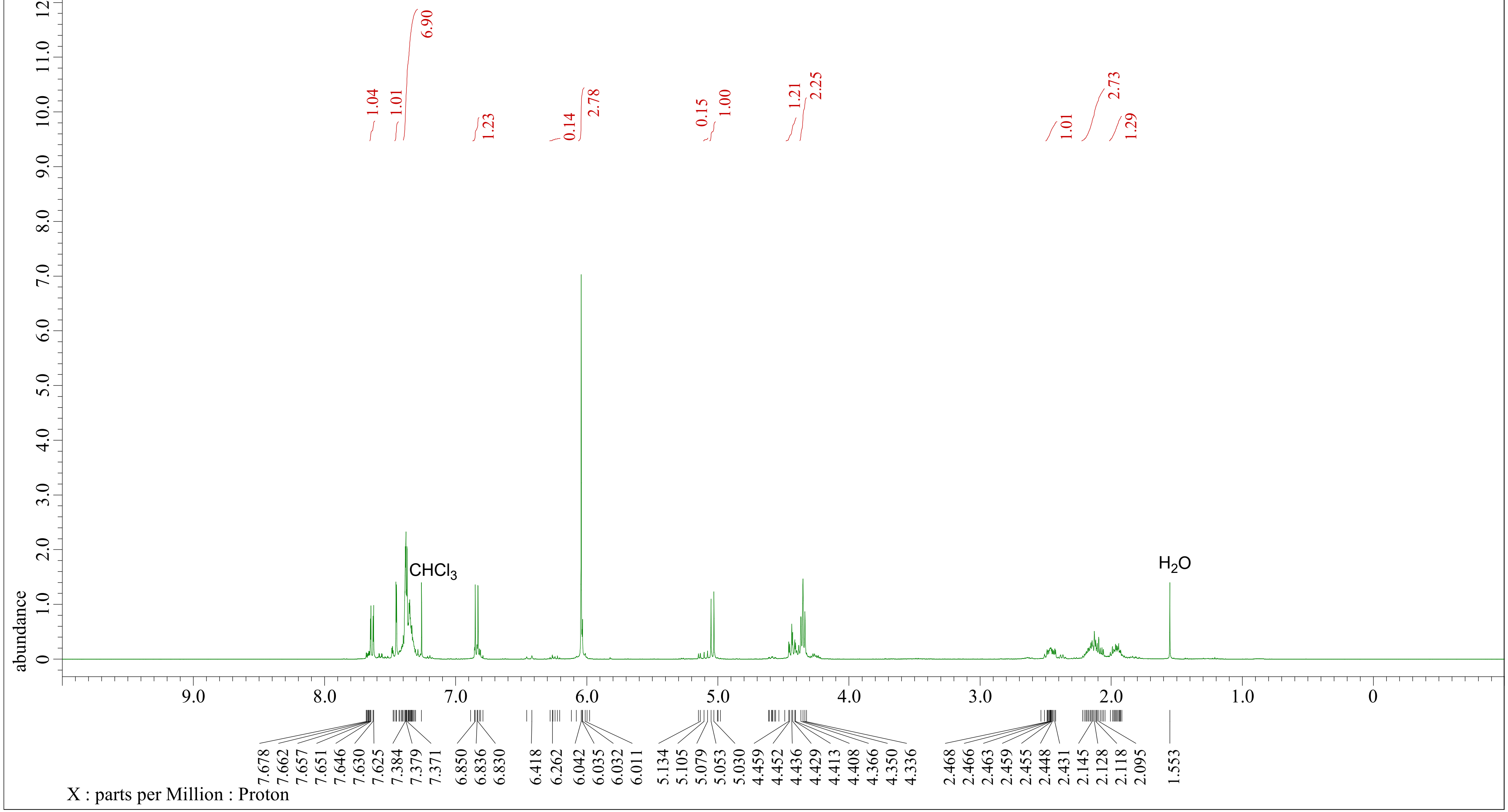

single pulse decoupled gated NOE
Filename = OSL_20241116_HM-12-029 descriptive run Column_Carbon-1-2.jdf
Author = OSL
Sample_Id = 20241116_HM-12-028 descriptive run Column
Creation_Time = 16-NOV-2024 18:59:34
Revision_Time = 15-JUL-2025 19:20:50

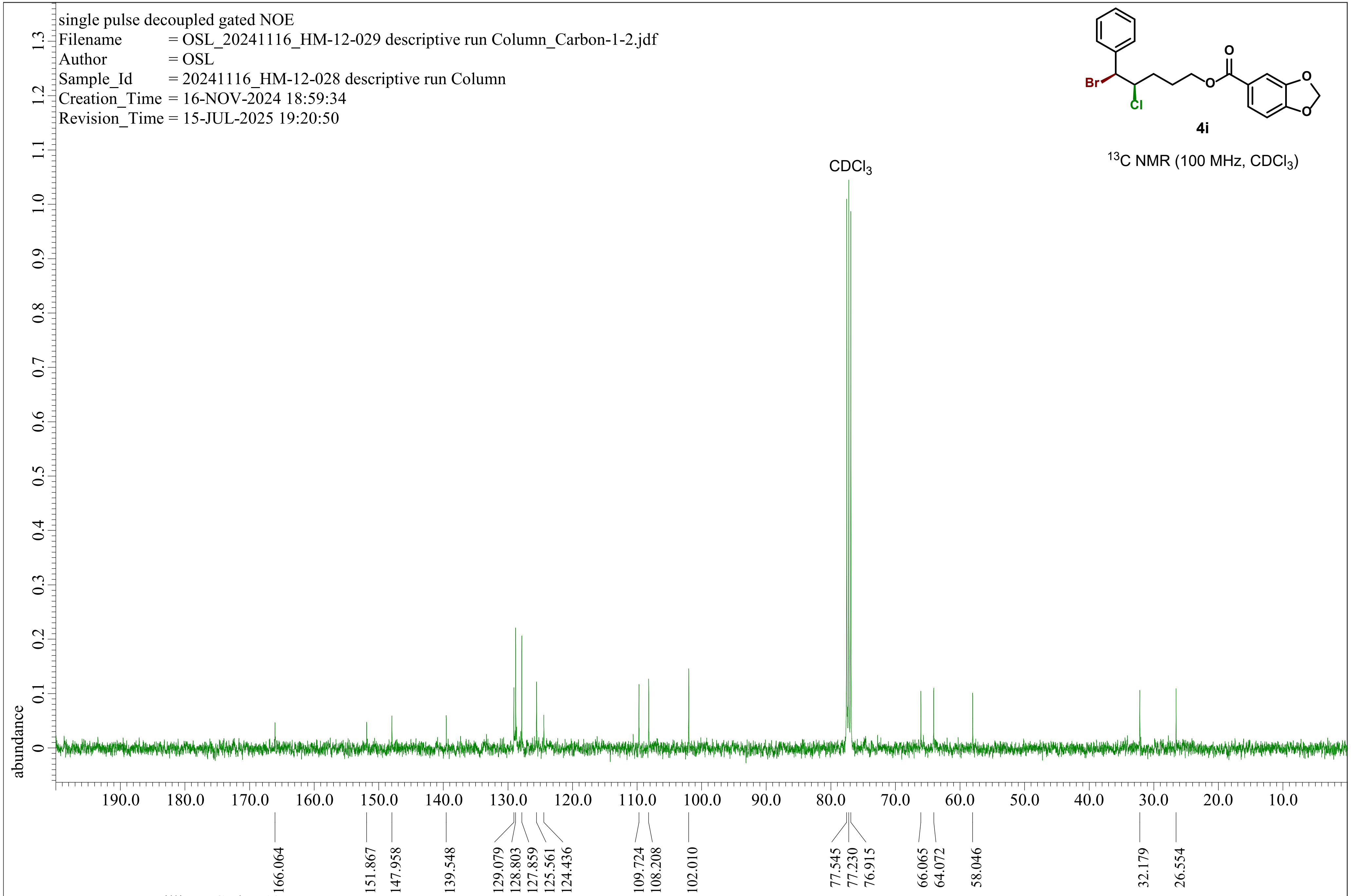

X : parts per Million : Carbon13

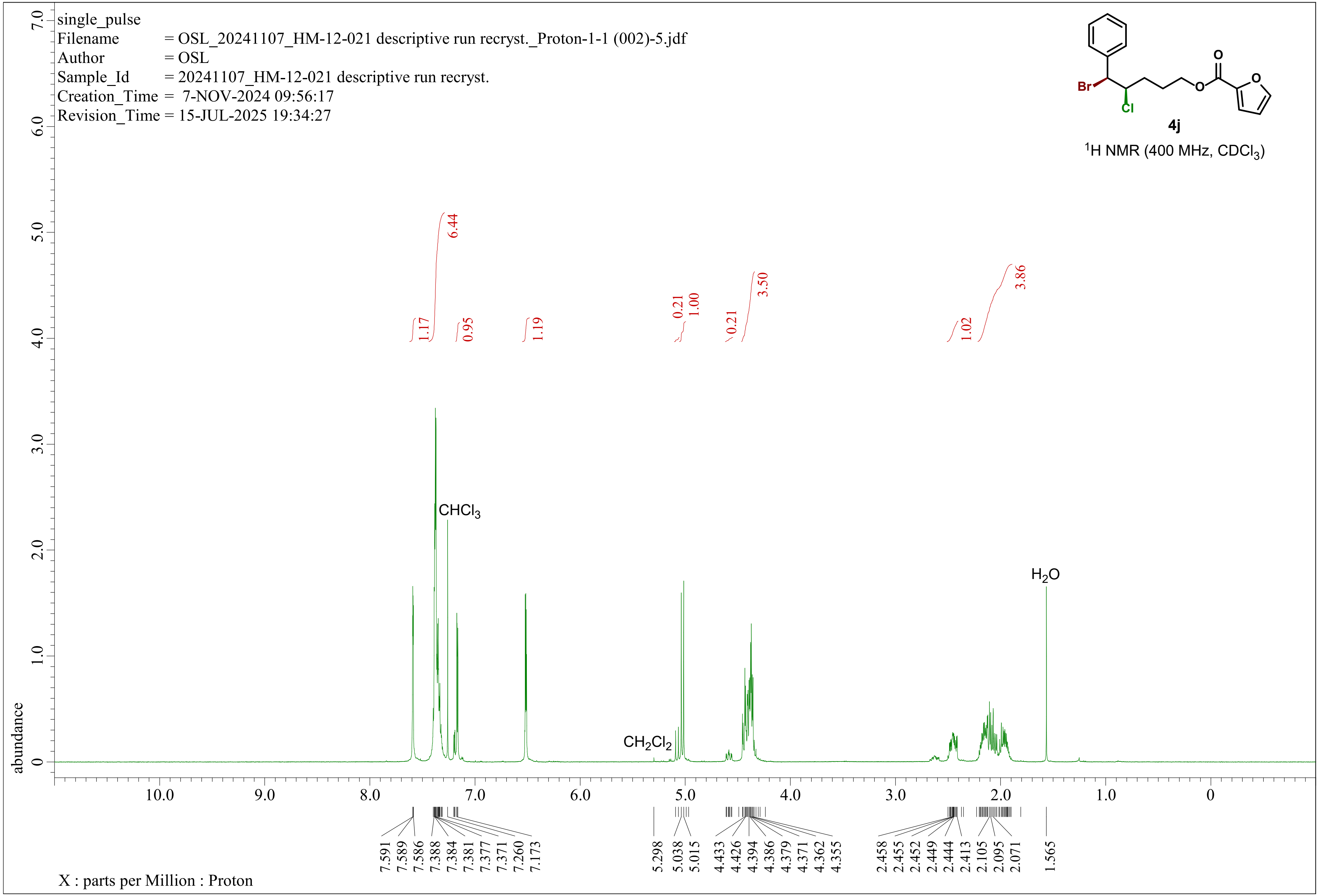
single_pulse
Filename = OSL_20241107_HM-12-021 descriptive run recryst._Proton-1-1 (002)-5.jdf
Author = OSL
Sample_Id = 20241107_HM-12-021 descriptive run recryst.
Creation_Time = 7-NOV-2024 09:56:17
Revision_Time = 15-JUL-2025 19:34:27
Br
Cl
O
4j
^{1}H NMR (400 MHz, $CDCl_3$)
1.17
6.44
0.95
1.19
0.21
1.00
0.21
3.50
1.02
3.86
$CHCl_3$
CH_2Cl_2
H_2O
abundance
X : parts per Million : Proton
7.591
7.589
7.586
7.388
7.384
7.381
7.377
7.371
7.260
7.173
5.298
5.038
5.015
4.433
4.426
4.394
4.386
4.379
4.371
4.362
4.355
2.458
2.455
2.452
2.449
2.444
2.413
2.105
2.095
2.071
1.565

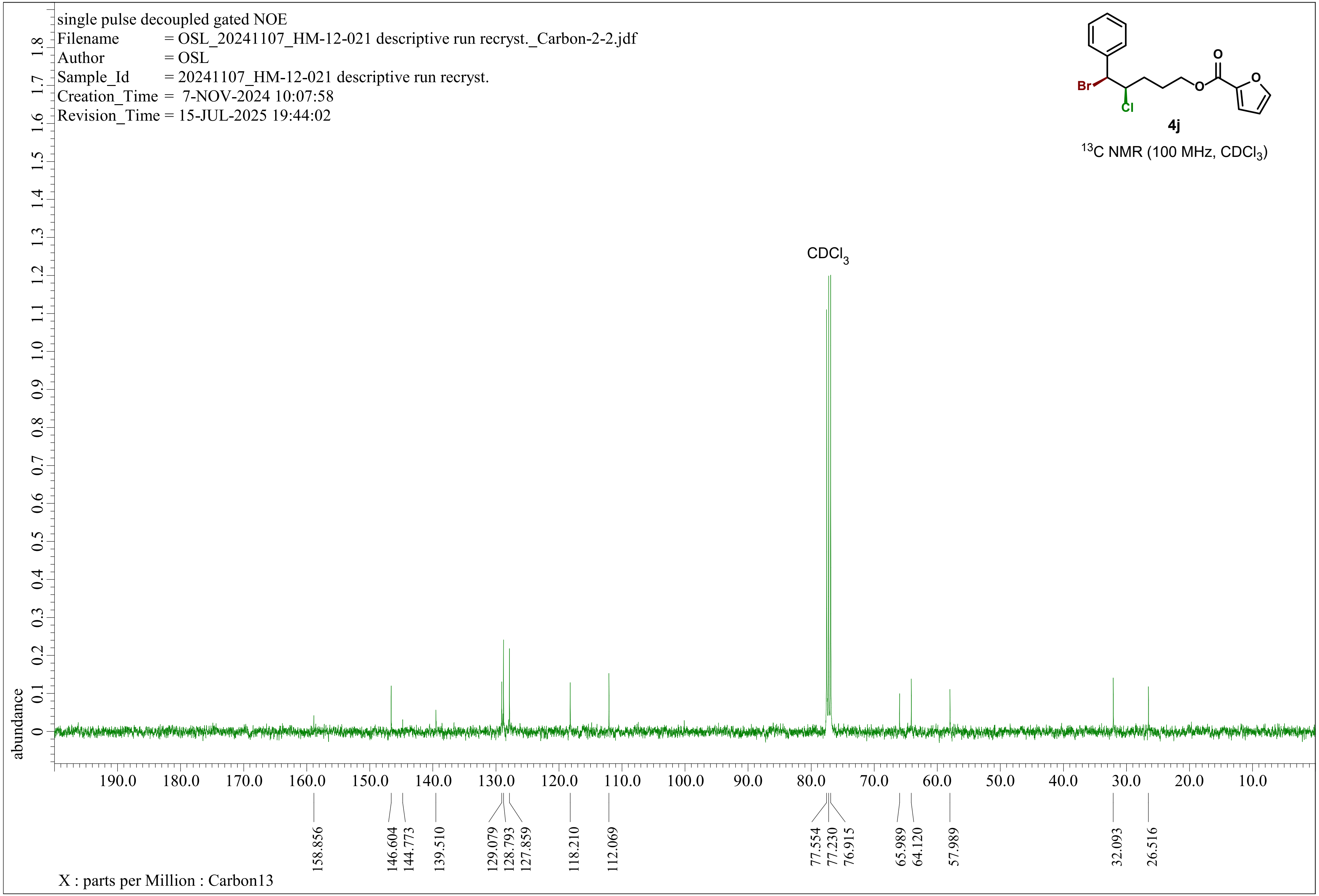

single pulse decoupled gated NOE
Filename = OSL_20241107_HM-12-021 descriptive run recryst._Carbon-2-2.jdf
Author = OSL
Sample_Id = 20241107_HM-12-021 descriptive run recryst.
Creation_Time = 7-NOV-2024 10:07:58
Revision_Time = 15-JUL-2025 19:44:02
Br
Cl
O
O
O
4j
13C NMR (100 MHz, CDCl3)
CDCl3
abundance
158.856
146.604
144.773
139.510
129.079
128.793
127.859
118.210
112.069
77.554
77.230
76.915
65.989
64.120
57.989
32.093
26.516
X : parts per Million : Carbon13

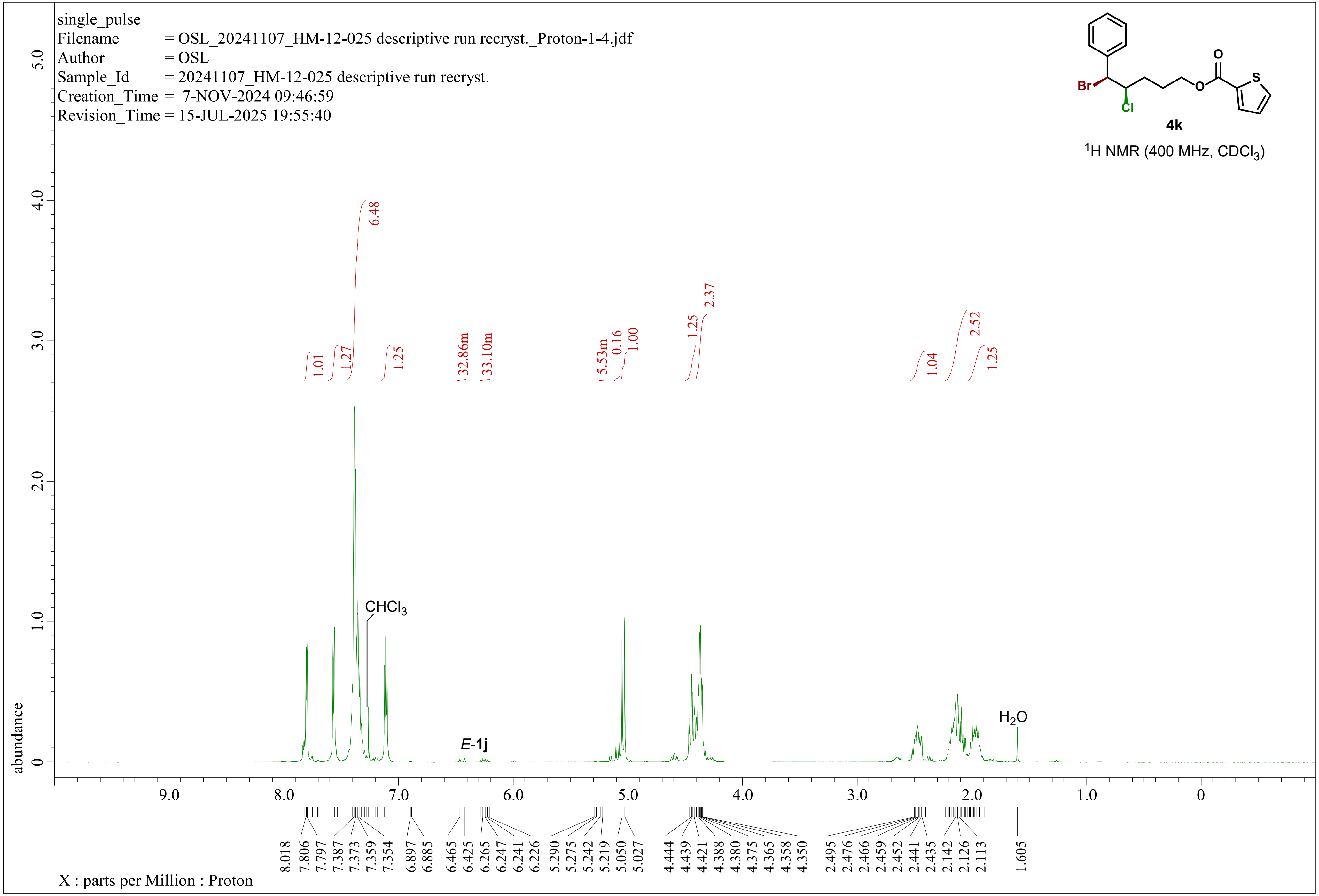
single_pulse
Filename = OSL_20241107_HM-12-025 descriptive run recryst._Proton-1-4.jdf
Author = OSL
Sample_Id = 20241107_HM-12-025 descriptive run recryst.
Creation_Time = 7-NOV-2024 09:46:59
Revision_Time = 15-JUL-2025 19:55:40
4k
1H NMR (400 MHz, CDCl3)
CHCl3
E-1j
H2O
abundance
X : parts per Million : Proton

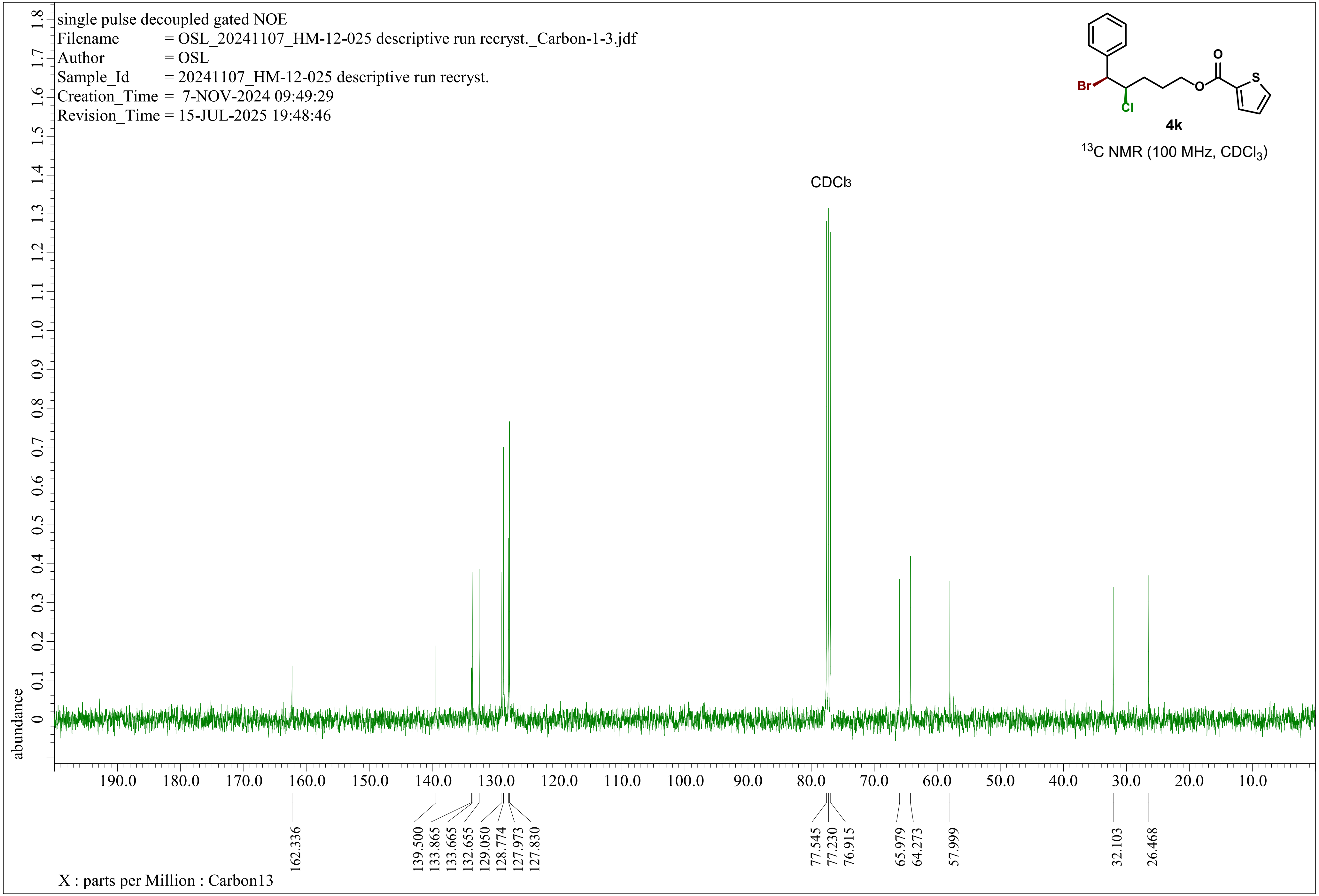
single pulse decoupled gated NOE
Filename = OSL_20241107_HM-12-025 descriptive run recryst._Carbon-1-3.jdf
Author = OSL
Sample_Id = 20241107_HM-12-025 descriptive run recryst.
Creation_Time = 7-NOV-2024 09:49:29
Revision_Time = 15-JUL-2025 19:48:46
Br
Cl
O
O
S
4k
13C NMR (100 MHz, CDCl3)
CDCl3
abundance
162.336
139.500
133.865
133.665
132.655
129.050
128.774
127.973
127.830
77.545
77.230
76.915
65.979
64.273
57.999
32.103
26.468
X : parts per Million : Carbon13

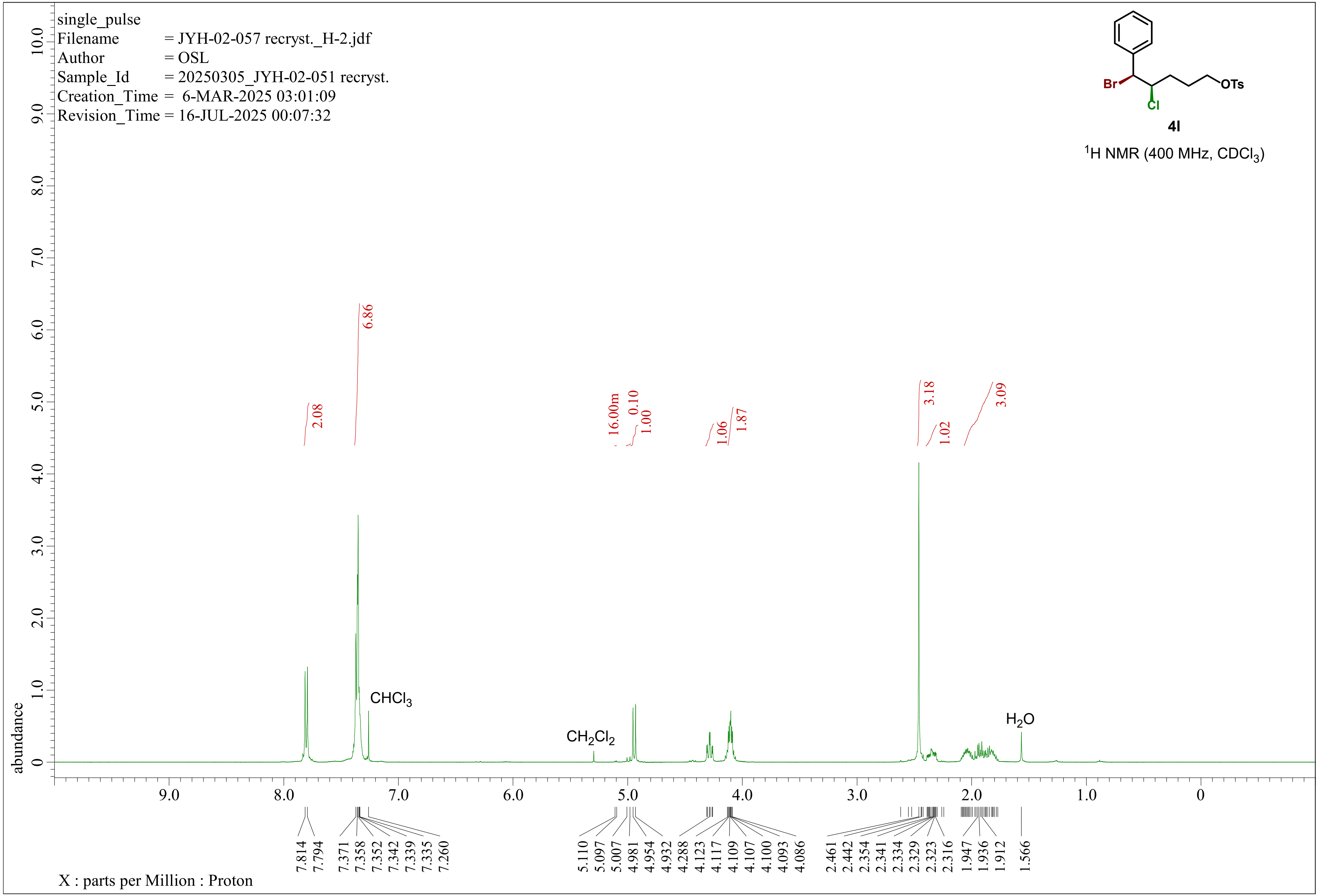
single_pulse
Filename = JYH-02-057 recryst._H-2.jdf
Author = OSL
Sample_Id = 20250305_JYH-02-051 recryst.
Creation_Time = 6-MAR-2025 03:01:09
Revision_Time = 16-JUL-2025 00:07:32
Br
Cl
OTs
4l
1H NMR (400 MHz, CDCl3)
2.08
6.86
16.00m
0.10
1.00
1.06
1.87
3.18
1.02
3.09
CHCl3
CH2Cl2
H2O
abundance
X : parts per Million : Proton
7.814
7.794
7.371
7.358
7.352
7.342
7.339
7.335
7.260
5.110
5.097
5.007
4.981
4.954
4.932
4.288
4.123
4.117
4.109
4.107
4.100
4.093
4.086
2.461
2.442
2.354
2.341
2.334
2.329
2.323
2.316
1.947
1.936
1.912
1.566

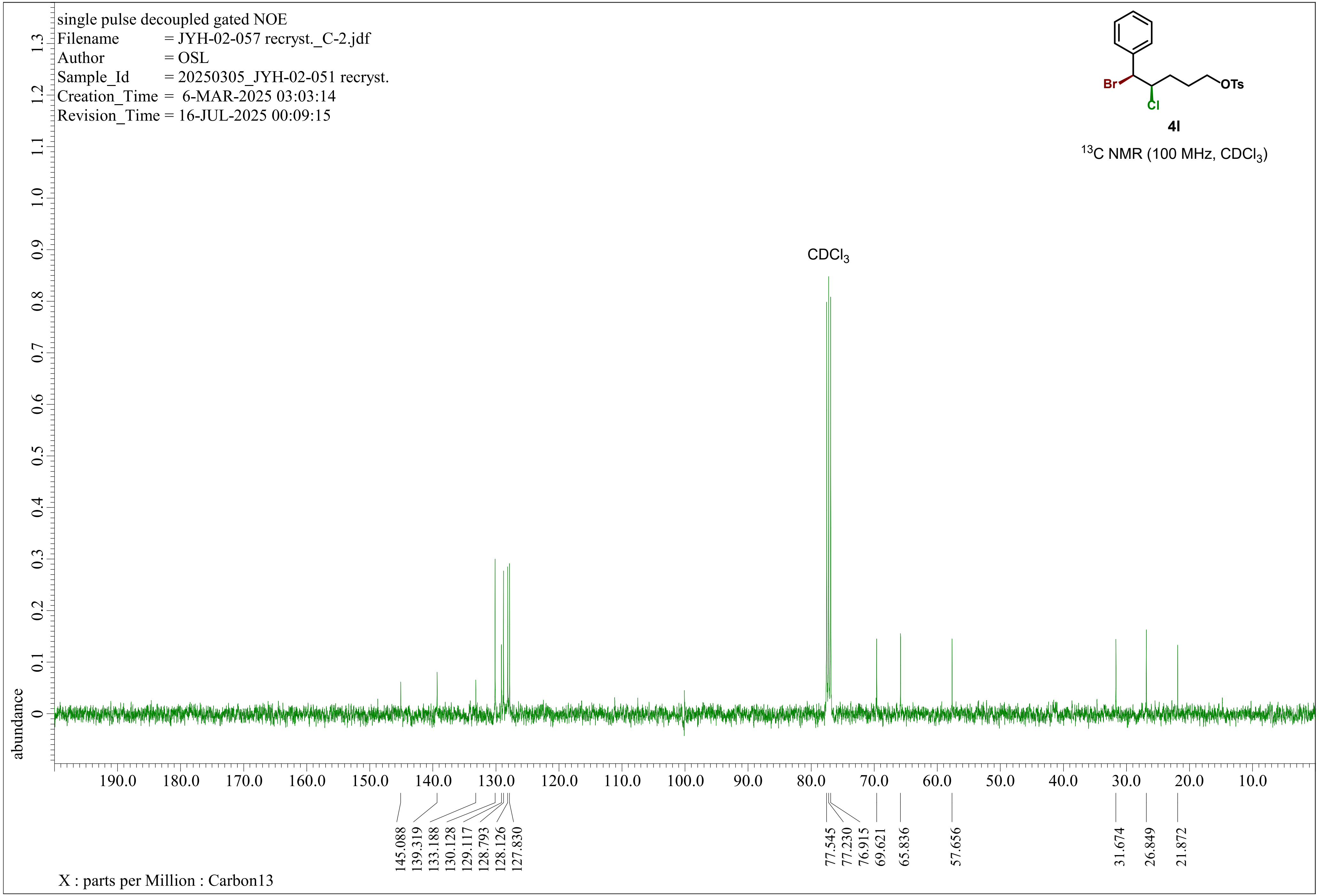

single pulse decoupled gated NOE
Filename = JYH-02-057 recryst._C-2.jdf
Author = OSL
Sample_Id = 20250305_JYH-02-051 recryst.
Creation_Time = 6-MAR-2025 03:03:14
Revision_Time = 16-JUL-2025 00:09:15
Br
Cl
OTs
4l
13C NMR (100 MHz, CDCl3)
CDCl3
abundance
145.088
139.319
133.188
130.128
129.117
128.793
128.126
127.830
77.545
77.230
76.915
69.621
65.836
57.656
31.674
26.849
21.872
X : parts per Million : Carbon13

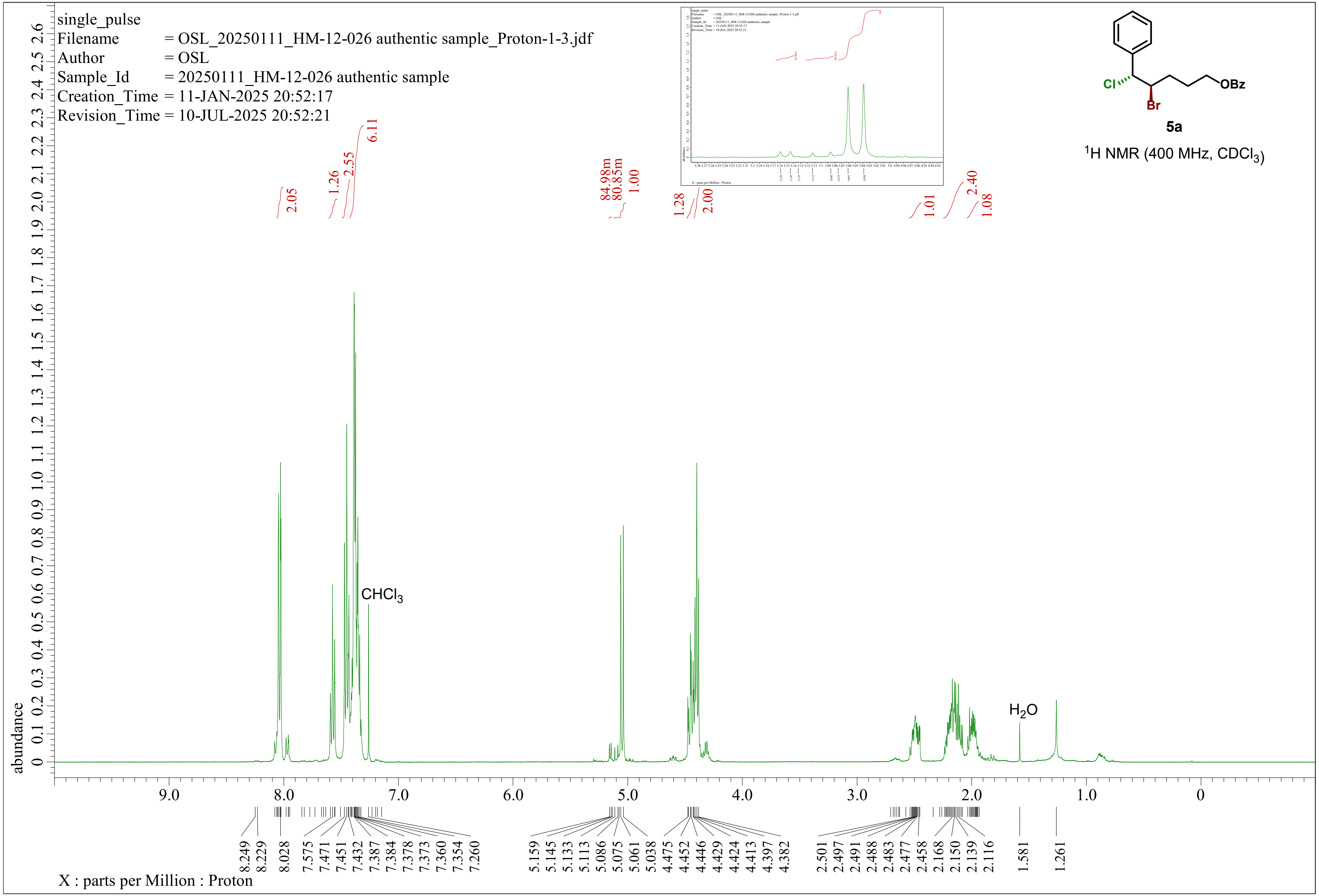
single_pulse
Filename = OSL_20250111_HM-12-026 authentic sample_Proton-1-3.jdf
Author = OSL
Sample_Id = 20250111_HM-12-026 authentic sample
Creation_Time = 11-JAN-2025 20:52:17
Revision_Time = 10-JUL-2025 20:52:21
Cl
Br
OBz
5a
1H NMR (400 MHz, CDCl3)
CHCl3
H2O
2.05
1.26
2.55
6.11
84.98m
80.85m
1.00
1.28
2.00
1.01
2.40
1.08
abundance
X : parts per Million : Proton
8.249 8.229 8.028 7.575 7.471 7.451 7.432 7.387 7.384 7.378 7.373 7.360 7.354 7.260
5.159 5.145 5.133 5.113 5.086 5.075 5.061 5.038 4.475 4.452 4.446 4.429 4.424 4.413 4.397 4.382
2.501 2.497 2.491 2.488 2.483 2.477 2.458 2.168 2.150 2.139 2.116 1.581 1.261

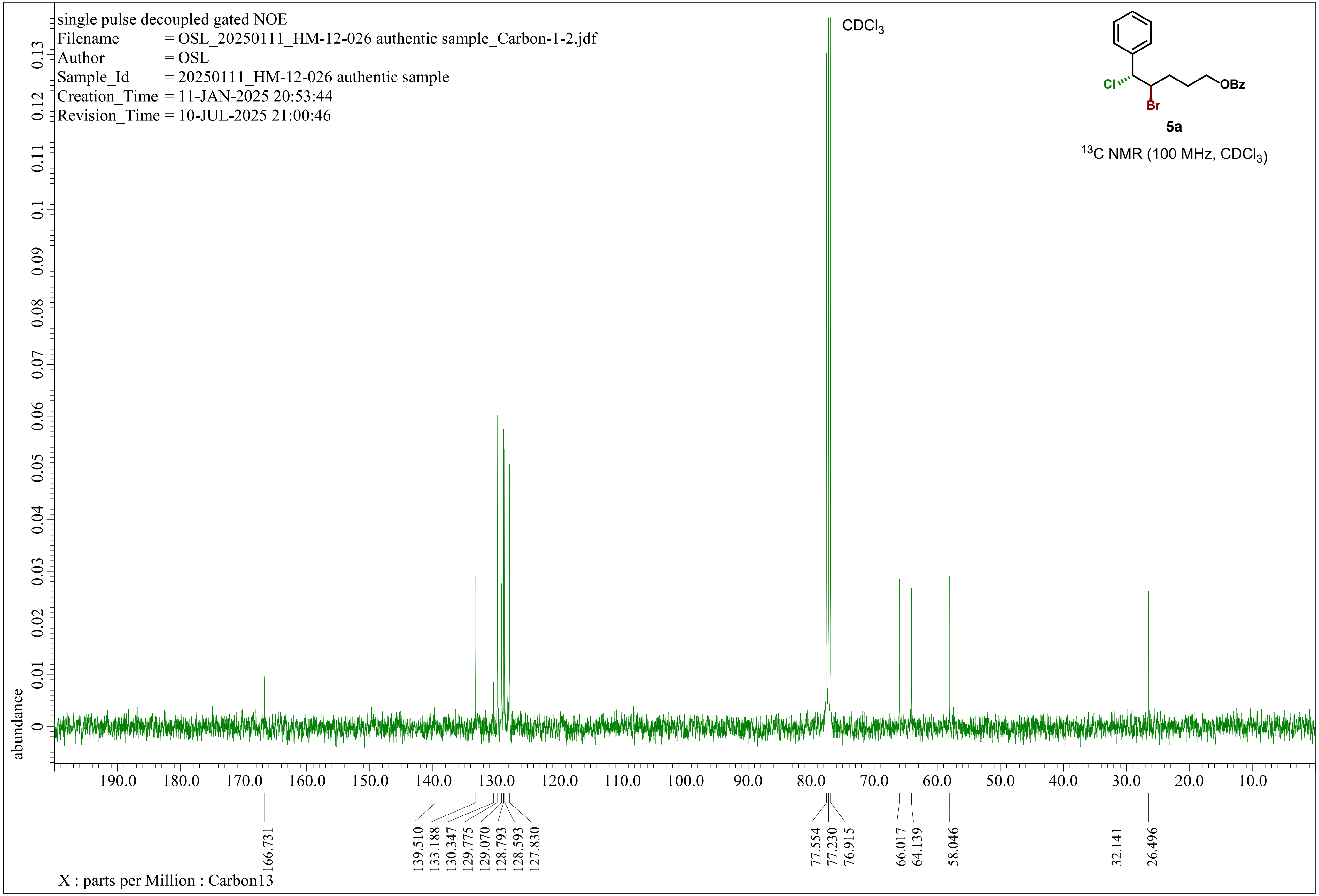
single pulse decoupled gated NOE
Filename = OSL_20250111_HM-12-026 authentic sample_Carbon-1-2.jdf
Author = OSL
Sample_Id = 20250111_HM-12-026 authentic sample
Creation_Time = 11-JAN-2025 20:53:44
Revision_Time = 10-JUL-2025 21:00:46
CDCl3
Cl
Br
OBz
5a
13C NMR (100 MHz, CDCl3)
abundance
X : parts per Million : Carbon13
166.731
139.510
133.188
130.347
129.775
129.070
128.793
128.593
127.830
77.554
77.230
76.915
66.017
64.139
58.046
32.141
26.496

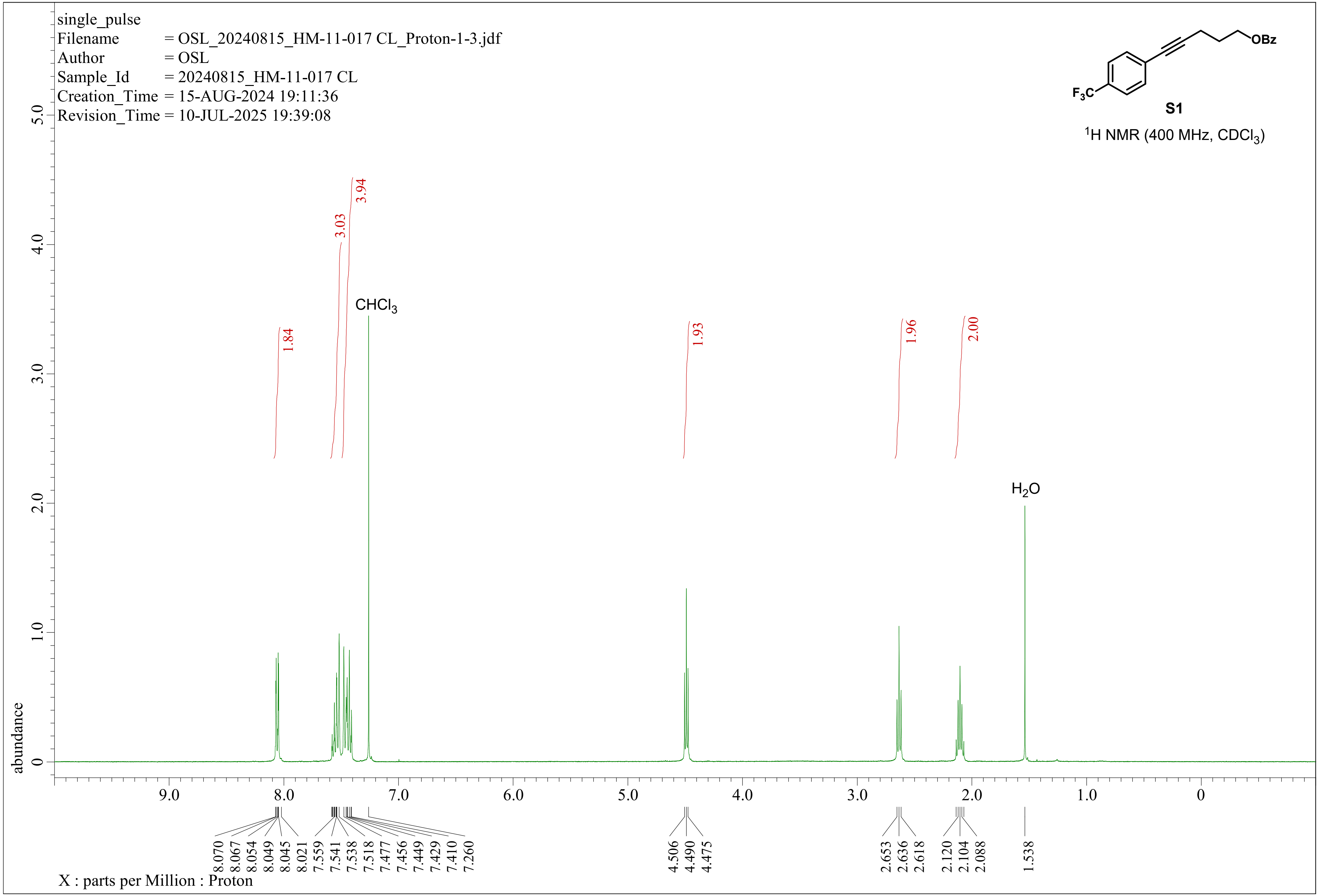
single_pulse
Filename = OSL_20240815_HM-11-017 CL_Proton-1-3.jdf
Author = OSL
Sample_Id = 20240815_HM-11-017 CL
Creation_Time = 15-AUG-2024 19:11:36
Revision_Time = 10-JUL-2025 19:39:08
F3C
OBz
S1
1H NMR (400 MHz, CDCl3)
CHCl3
H2O
1.84
3.03
3.94
1.93
1.96
2.00
abundance
X : parts per Million : Proton
8.070
8.067
8.054
8.049
8.045
8.021
7.559
7.541
7.538
7.518
7.477
7.456
7.449
7.429
7.410
7.260
4.506
4.490
4.475
2.653
2.636
2.618
2.120
2.104
2.088
1.538

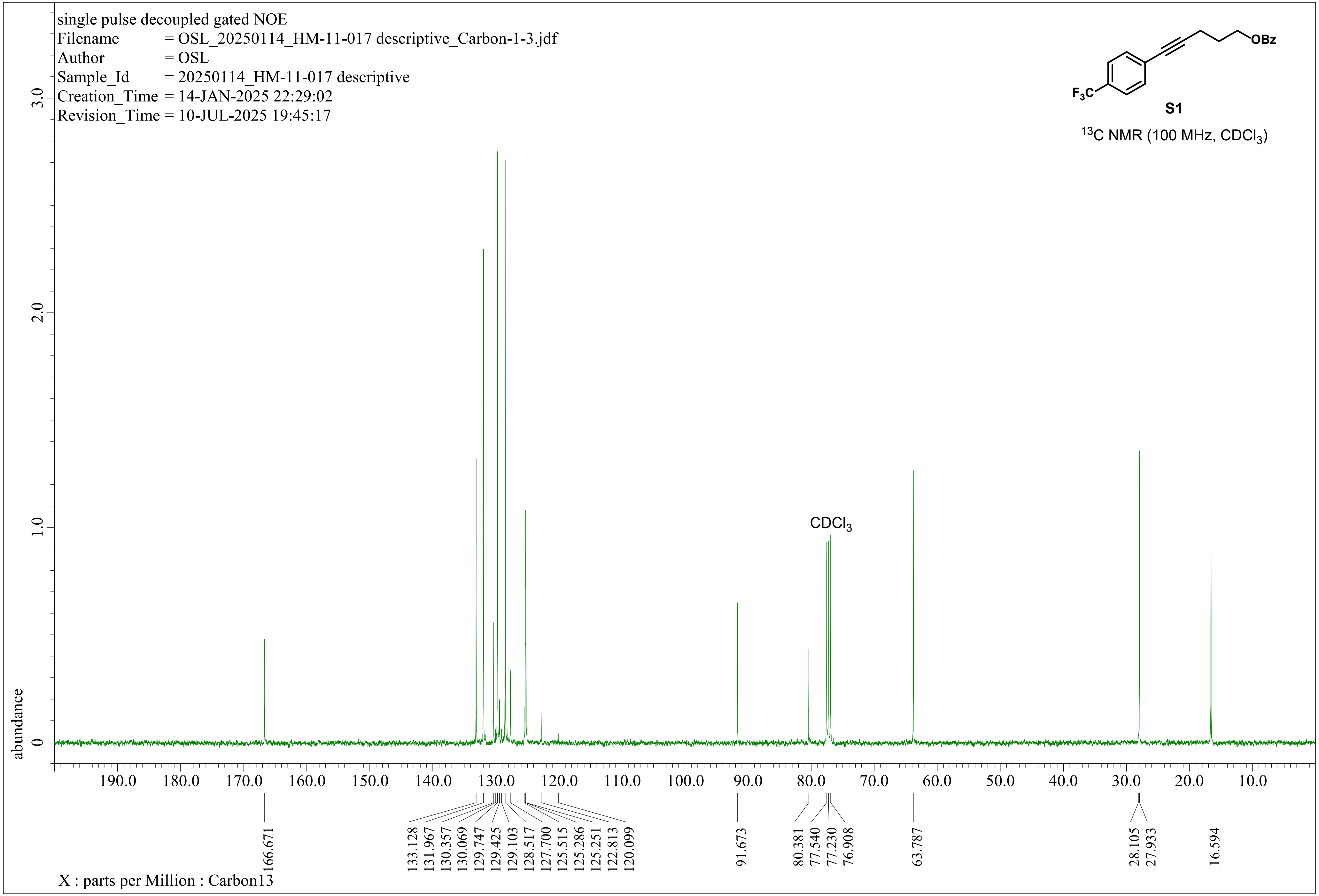
single pulse decoupled gated NOE
Filename = OSL_20250114_HM-11-017 descriptive_Carbon-1-3.jdf
Author = OSL
Sample_Id = 20250114_HM-11-017 descriptive
Creation_Time = 14-JAN-2025 22:29:02
Revision_Time = 10-JUL-2025 19:45:17
F3C
OBz
S1
13C NMR (100 MHz, CDCl3)
CDCl3
abundance
3.0
2.0
1.0
0
190.0 180.0 170.0 160.0 150.0 140.0 130.0 120.0 110.0 100.0 90.0 80.0 70.0 60.0 50.0 40.0 30.0 20.0 10.0
166.671
133.128 131.967 130.357 130.069 129.747 129.425 129.103 128.517 127.700 125.515 125.286 125.251 122.813 120.099
91.673
80.381 77.540 77.230 76.908
63.787
28.105 27.933
16.594
X : parts per Million : Carbon13

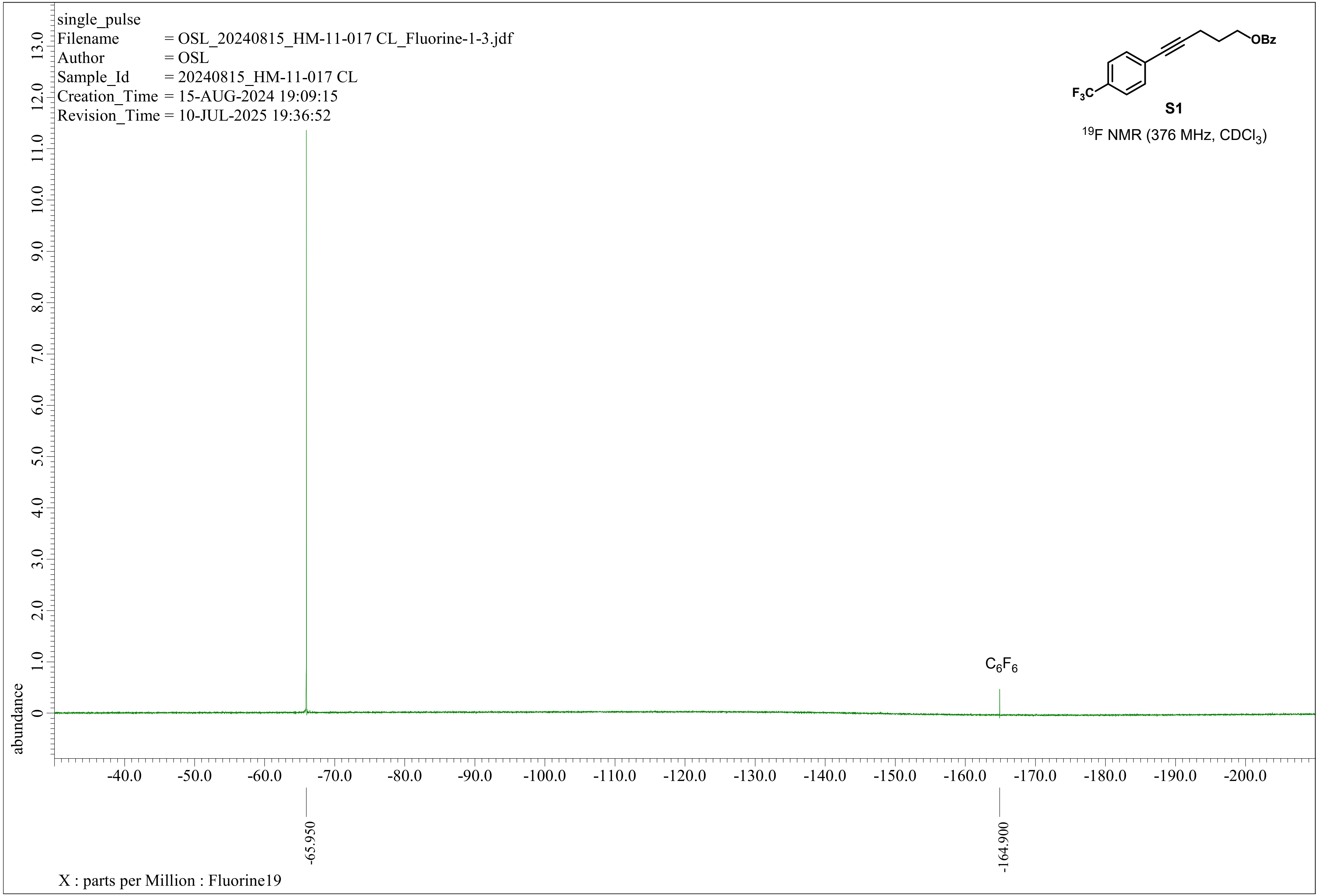
single_pulse
Filename = OSL_20240815_HM-11-017 CL_Fluorine-1-3.jdf
Author = OSL
Sample_Id = 20240815_HM-11-017 CL
Creation_Time = 15-AUG-2024 19:09:15
Revision_Time = 10-JUL-2025 19:36:52
F3C
OBz
S1
19F NMR (376 MHz, CDCl3)
C6F6
abundance
-65.950
-164.900
X : parts per Million : Fluorine19

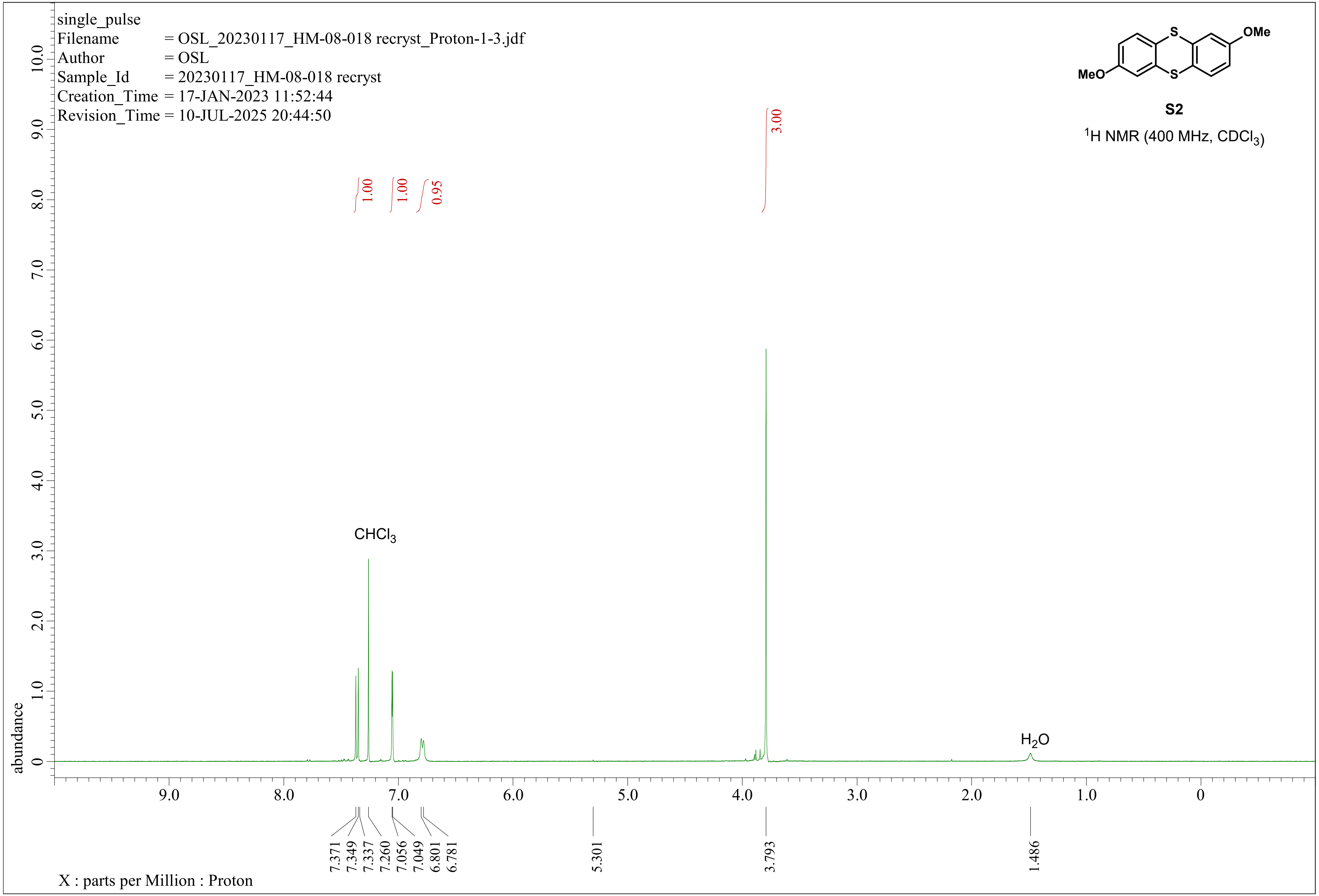
single_pulse
Filename = OSL_20230117_HM-08-018 recryst_Proton-1-3.jdf
Author = OSL
Sample_Id = 20230117_HM-08-018 recryst
Creation_Time = 17-JAN-2023 11:52:44
Revision_Time = 10-JUL-2025 20:44:50
S
OMe
MeO
S
S2
^1H NMR (400 MHz, $CDCl_3$)
1.00
1.00
0.95
3.00
$CHCl_3$
H_2O
abundance
7.371
7.349
7.337
7.260
7.056
7.049
6.801
6.781
5.301
3.793
1.486
X : parts per Million : Proton

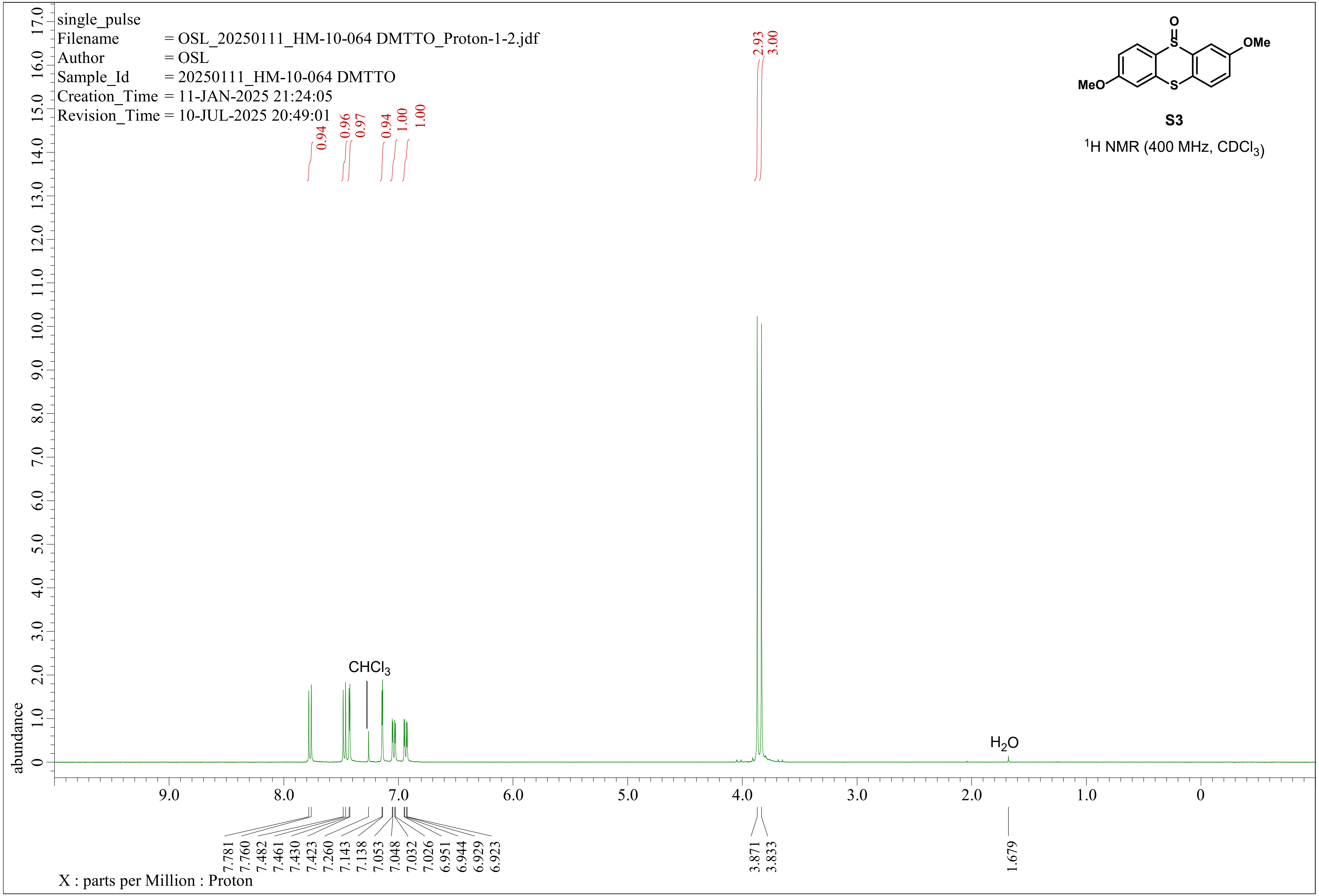

single_pulse
Filename = OSL_20250111_HM-10-064 DMTTO_Proton-1-2.jdf
Author = OSL
Sample_Id = 20250111_HM-10-064 DMTTO
Creation_Time = 11-JAN-2025 21:24:05
Revision_Time = 10-JUL-2025 20:49:01
MeO
OMe
S3
1H NMR (400 MHz, CDCl3)
CHCl3
H2O
0.94
0.96
0.97
0.94
1.00
1.00
2.93
3.00
7.781
7.760
7.482
7.461
7.430
7.423
7.260
7.143
7.138
7.053
7.048
7.032
7.026
6.951
6.944
6.929
6.923
3.871
3.833
1.679
abundance
X : parts per Million : Proton

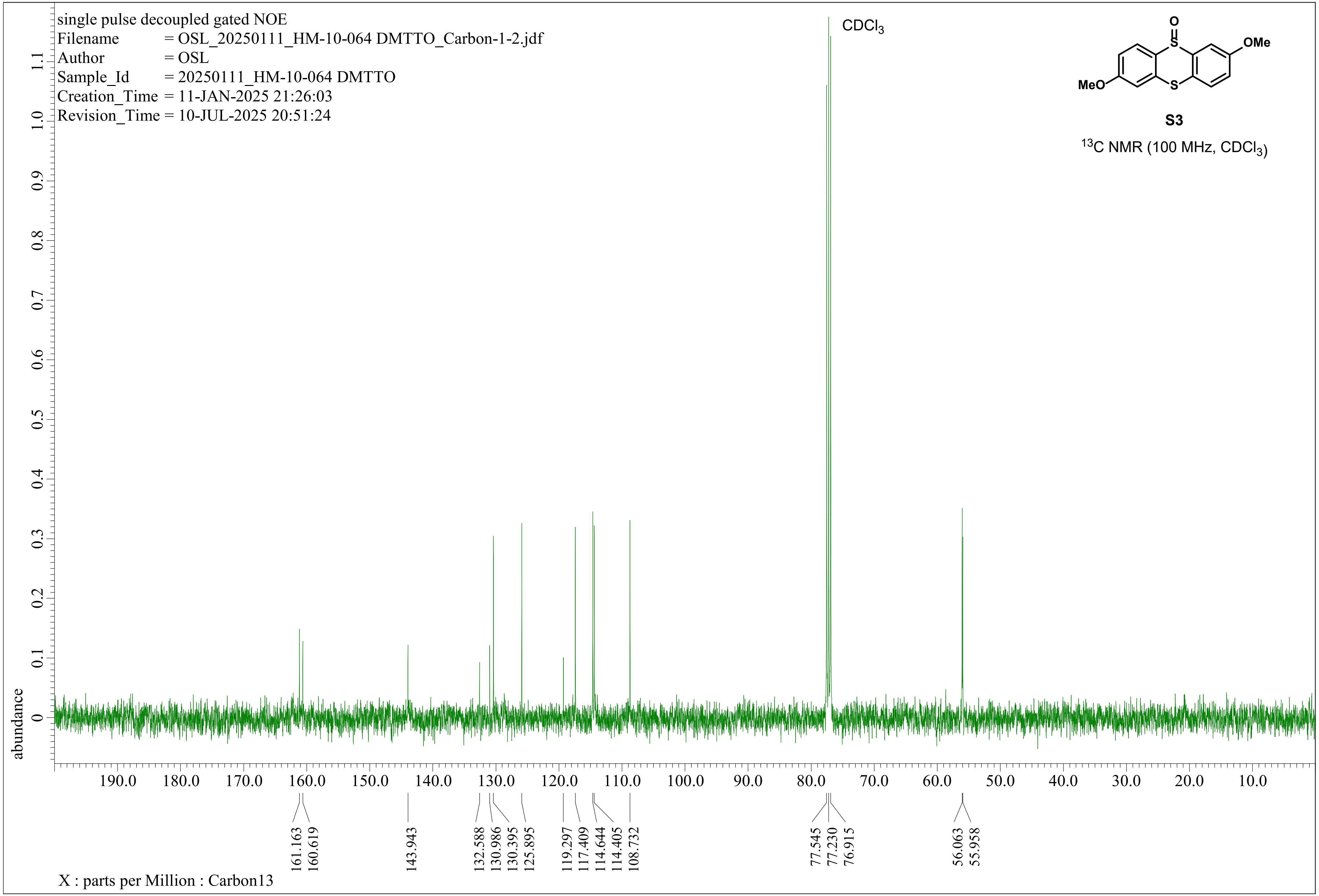
single pulse decoupled gated NOE
Filename = OSL_20250111_HM-10-064 DMTTO_Carbon-1-2.jdf
Author = OSL
Sample_Id = 20250111_HM-10-064 DMTTO
Creation_Time = 11-JAN-2025 21:26:03
Revision_Time = 10-JUL-2025 20:51:24
CDCl3
O
S
S
MeO
OMe
S3
13C NMR (100 MHz, CDCl3)
abundance
161.163
160.619
143.943
132.588
130.986
130.395
125.895
119.297
117.409
114.644
114.405
108.732
77.545
77.230
76.915
56.063
55.958
X : parts per Million : Carbon13

4. References

1. Fanourakis, A.; Hodson, N. J.; Lit, A. R.; Phipps, R. J. Substrate-Directed Enantioselective Aziridination of Alkenyl Alcohols Controlled by a Chiral Cation. *J. Am. Chem. Soc.* **2023**, *145*, 7516−7527.
2. Zhang, B.-S.; Wang, F.; Gou, X.-Y.; Yang, Y.-H.; Jia, W.-Y.; Liang, Y.-M.; Wang, X.-C.; Li, Y.; Quan, Z.-J. Palladium-Catalyzed Synthesis of Tricyclic Indoles via a N–S Bond Cleavage Strategy. *Org. Lett.* **2021**, *23*, 7518−7523.
3. Wearing, E. R.; Blackmun, D. E.; Becker, M. R.; Schindler, C. S. 1- and 2-Azetines via Visible Light-Mediated [2 + 2]-Cycloadditions of Alkynes and Oximes. *J. Am. Chem. Soc.* **2021**, *143*, 16235–16242.
4. Yue, Q.; Yang, T.; Yang, Y.; Zhang, C.; Zhang, Q.; Li, D. Divergent Reactivity of (Diacyloxyiodo)arenes under Palladium Catalysis: Controlled Allylic C–H Acyloxylation and Vinylic Arylation. *Asian J. Org. Chem.* **2017**, *6*, 936–942.
5. Moon, H.; Jung, J.; Choi, J.-H.; Chung, W.-j. Stereospecific *syn*-Dihalogenations and Regiodivergent *syn*-Interhalogenation of Alkenes via Vicinal Double Electrophilic Activation Strategy. *Nat. Commun.* **2024**, *15*, 3710.
6. Satoh, T.; Kondo, A.; Musashi, J. Generation of Magnesium Carbenoids from 1-Chloroalkyl Phenyl Sulfoxides with a Grignard Reagent and Applications to Alkylation and Olefin Synthesis. *Tetrahedron* **2004**, *60*, 5453−5460.
7. Sprug, A.; Schnakenburg, G.; Waldvogel, S. R. Oxidative Coupling of Diaryldisulfides by $MoCl_5$ to Thianthrenes. *Chem. Eur. J.* **2009**, *15*, 13313–13317.
8. Agilent **2014**. CrysAlis PRO. Agilent Technologies Ltd, Yarnton, Oxfordshire, England.
9. Dolomanov, O. V.; Bourhis, L. J.; Gildea, R. J.; Howard, J. A. K.; Puschmann, H. OLEX2: A Complete Structure Solution, Refinement and Analysis Program. *J. Appl. Crystallogr.* **2009**, *42*, 339–341.
